# Molecular Response to High Hydrostatic Pressure: Time-Series Transcriptomic Analysis of Shallow-Water Sea Cucumber *Apostichopus japonicus*

**DOI:** 10.3389/fgene.2020.00355

**Published:** 2020-04-30

**Authors:** Jiawei Chen, Linying Liang, Yanan Li, Haibin Zhang

**Affiliations:** ^1^Institute of Deep-sea Science and Engineering, Chinese Academy of Sciences, Sanya, China; ^2^College of Earth Sciences, University of Chinese Academy of Sciences, Beijing, China

**Keywords:** time series, transcriptomic, molecular response, hydrostatic pressure, sea cucumber

## Abstract

Hydrostatic pressure is a key environmental factor constraining the benthic migration of shallow-water invertebrates. Although many studies have examined the physiological effects of high hydrostatic pressure on shallow-water invertebrates, the molecular response to high pressure is not fully understood. This question has received increasing attention because ocean warming is forcing the bathymetric migrations of shallow-water invertebrates. Here, we applied time-series transcriptomic analysis to high-pressure incubated and atmospheric pressure-recovered shallow-water sea cucumber (*Apostichopus japonicus*) to address this question. A total of 44 samples from 15 experimental groups were sequenced. Our results showed that most genes responded to pressure stress at the beginning when pressure was changed, but significant differences of gene expression appeared after 4 to 6 h. Transcription was the most sensitive biological process responding to high-pressure exposure, which was enriched among up-regulated genes after 2 h, followed by ubiquitination (4 h), endocytosis (6 h), stress response (6 h), methylation regulation (24 h), and transmembrane transportation (24 h). After high-pressure incubation, all these biological processes remained up-regulated within 4–6 h at atmospheric pressure. Overall, our results revealed the dynamic transcriptional response of *A. japonicus* to high-pressure exposure. Additionally, few quantitative or functional responses related to *A. japonicus* on transcriptional level were introduced by hydrostatic pressure changes after 1 h, and main biological responses were introduced after 4 h, suggesting that, when hydrostatic pressure is the mainly changed environmental factor, it will be better to fix sea cucumber samples for transcriptomic analysis within 1 h, but 4 h will be also acceptable.

## Introduction

Hydrostatic pressure is one of the major environmental factors limiting the distribution of shallow-water invertebrates (Brown and Thatje, 2014, 2015). The question of how hydrostatic pressure affects shallow-water invertebrates has received increasing attention because ocean warming is forcing the bathymetric migrations of shallow-water marine invertebrates (Pinsky et al., 2013; Brown and Thatje, 2015). Many studies focused on the effects of high pressure on metabolic rates, behaviors, growth, and development status of marine benthic invertebrates, indicating that most shallow-water invertebrates can survive at the hydrostatic pressure (∼20 MPa), which is outside their known natural distributions for a period of time (Brown and Thatje, 2014). However, the molecular response to high pressure on transcriptional level was seldom studied.

The study of molecular response to high pressure can also provide clues to reveal evolutionary processes of extant deep-sea invertebrates. Most extant deep-sea invertebrates originated from shallow waters through isothermal water columns (Young et al., 1997; Thatje et al., 2005; Brown and Thatje, 2014). Given that expression variation may be facilitated by regulatory elements or epigenetic mechanisms that alter gene expression even before genetic variants arise in the population (West-Eberhard, 2003), population-level differential expression may reflect the early processes that underlie adaptive divergence (Derome et al., 2006; Jeukens et al., 2010).

Somero (1992) and Oger and Jebbar (2010) have reviewed the effects of hydrostatic pressure on shallow-water organisms. On the one hand, high pressure affects the equilibrium and reaction rates of cellular biochemical reaction. On the other hand, high pressure affects the intermolecular distances and weak chemical bonds, resulting in denaturing of proteins, stiffening of lipid bilayer, and stabilizing of double-stranded nucleic acids. Many biological processes were reported important for the adaptation of high-pressure environment, including genetic information processing (Lan et al., 2017), fatty acid metabolism (Wang et al., 2019), antioxidation (Xie et al., 2018), and immunity (Oliver et al., 2010). Studies on shallow-water amphipod indicated that genes responding to high-pressure stress are similar to those involving deep-sea adaptation (Chen et al., 2019).

One limitation of relevant studies on using high-pressure incubation is that most of them used observations from only a few single time points to describe organismal responses to high pressure, usually including one short or acute exposure (<∼6 h) and one sustained exposure (>∼24 h) (Thatje et al., 2010; Cottin et al., 2012; Smith and Thatje, 2012; Morris et al., 2015). This methodology condenses the temporal variation into a single time point and cannot represent the dynamic physiological regulation processes. To obtain a more nuanced understanding of molecular response to high pressure, we set a time series of high-pressure incubations and use a transcriptomic method to identify biological processes involving molecular response to high pressure.

Another limitation is that many studies focused on the responses of shallow-water invertebrates to high pressure, but only a few studied what happens to experimental individuals after high-pressure incubation. Brown et al. (2017) found that all experimental individuals survived ≥3 months after acute high-pressure incubation, and normal behaviors were observed after days of quiescence, whereas not all individuals could survive after sustained high-pressure incubation. To determine how long the effects on transcriptional level of high pressure would last, we also conducted time-series atmospheric pressure recovery experiments after 24 h high-pressure incubation.

Transcriptomic analysis is a powerful and increasingly popular approach for studying molecular response to the changes of environmental factors. To minimize interference, samples for transcriptomic analysis require consistent handling with minimal exposure to unwanted stimuli before and during collection (Alvarez et al., 2015). Therefore, each sample should be collected at approximately the same time and flash frozen or treated by RNA preservation additives immediately. However, deep-sea benthic invertebrates sometimes cannot be fixed *in situ* in field work, and it takes minutes to hours before they were fixed on board or on land. Thus, the relation between sampling time and differential expression caused by hydrostatic pressure changes needs to be determined. In this study, time-series high-pressure incubation and atmospheric pressure recovery experiments could provide data to address this question.

The sea cucumber *Apostichopus japonicus*, which belongs to the order Synallactida, is a temperate species mainly distributed along the coastal area of Eastern Asia (Han et al., 2016). It is a popular seafood and common aquaculture species in China. Additionally, Synallactida are not only ubiquitous in coastal areas but are also widespread at the abyssal depth (Miller et al., 2017). Given that deep-sea organisms do not require novel functions but apparently use gene sets homologous to their coastal relatives for stress response to adapt to deep-sea environments (Gunbin et al., 2009; Zheng et al., 2017), we predicted that *A. japonicus* can survive in high-pressure environment for a period of time and chose it as our experimental species.

In this study, we investigated time-series transcriptional response of *A. japonicus* to high-pressure incubations. Experimental individuals were incubated at atmospheric pressure (P0) and at high pressure (25 MPa) for 1 (P1), 2 (P2), 4 (P4), 6 (P6), 12 (P12), and 24 h (P24). In addition, we measured the experimental contexts before and after each incubation experiment to ascertain the stabilization of seawater qualities, and no significant difference was observed (Supplementary Table S1). We also investigated time-series transcriptional response to atmospheric pressure recoveries after high-pressure incubation. Experimental individuals were recovered for 0.5 (R0.5), 1 (R1), 2 (R2), 4 (R4), 6 (R6), 12 (R12), 24 (R24), and 48 h (R48). The specific questions revealed with these data were (i) how *A. japonicus* responds to hydrostatic pressure changes on transcriptional level in time series and (ii) how much variation the redundant sampling time would introduce to sea cucumber samples for transcriptomic analysis studies when hydrostatic pressure was the mainly changed environmental factor.

## Results

### Illumina Sequencing, De Novo Assembly, Gene Annotation, and RNA-Seq Validation

A total of 44 samples from 15 experimental groups were sequenced. More than ∼10-Gb clean data were generated for each sample after quality control (Supplementary Table S2). Paired reads of 21 high-pressure incubated samples from seven groups, including P0, P1, P2, P4, P6, P12, and P24, were assembled into 590,006 unigenes with a total length of 488,782,028 bp and an N50 length of 1,192 bp (Supplementary Table S3). To evaluate the status of recovery samples, P0 and P24 need to be assembled with recovery groups as control groups. Therefore, paired reads of 29 samples from 10 groups, including P0, P24, R0.5, R1, R2, R4, R6, R12, R24, and R48, were assembled into 699,913 unigenes with a total length of 542,758,628 bp and an N50 length of 1,085 bp (Supplementary Table S4).

BUSCO completeness of these two transcriptomes were 91.3% (single copy: 29.8%, duplicated: 61.5%, fragmented: 7.5%, and missing: 1.2%) and 91.5% (single copy: 31.6%, duplicated: 59.9%, fragmented: 7.2%, and missing: 1.3%), respectively. Two quantification methods, namely, RNA-seq and quantitative real-time reverse transcription–polymerase chain reaction (qPCR), produced largely consistent results, and their Pearson correlation coefficients ranged from 0.81 to 0.99 (Supplementary Table S5). The results of BUSCO and qPCR validated the reliability of the assembly and RNA-seq.

### Differential Expression Analysis Between Time-Series Experimental Groups

**FIGURE 1:**
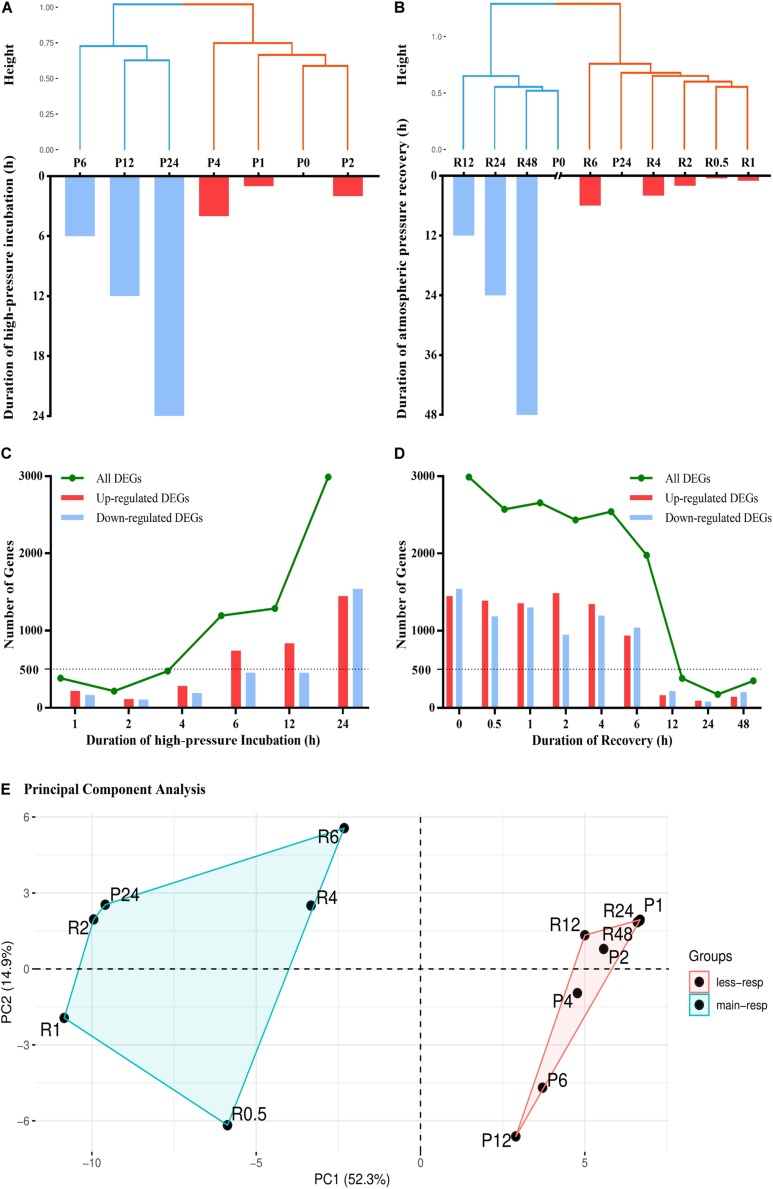
Results of hierarchical clustering (HCL), differential expression analysis, and principal component analysis (PCA). **(A)** Hierarchical clustering analysis of seven experimental groups, including P0, P1, P2, P4, P6, P12, and P24. The high-pressure incubated duration of each group is shown via bar chart. **(B)** Hierarchical clustering analysis of 10 experimental groups, including P0, P24, R0.5, R1, R2, R4, R6, R12, R24, and R48. The atmospheric pressure recovered duration of each group is shown via bar chart. **(C)** The number of differentially expressed genes (DEGs) in different high-pressure incubations. **(D)** The number of DEGs in different atmospheric pressure recoveries. **(E)** Principal component analysis of all experimental groups according to their enrichment analysis results. less-resp, less-responding period; main-resp, main-responding period.

To address the question how quickly the gene expression changes occur in response to pressure changes, we used hierarchical clustering analysis to visualize the similarity between different groups according to the expression levels of all unigenes. According to total within sum of square, the time-series high-pressure incubation and atmospheric pressure recovery groups should be divided into two clusters (Figures 1A,B), and the results of anosim analysis are *R* = 1, *p* = 0.035 and *R* = 0.98, *p* = 0.004, respectively.

We used DESeq2 R Package to detect differentially expressed genes (DEGs) in different experimental groups (Figures 1C,D). The number of DEGs within 4 h (P1, P2, and P4) was fewer than 500. After 4-h incubation, the number of DEGs increased sharply. A total of 1,194 and 1,287 DEGs were detected in P6 and P12, respectively. The numbers more than doubled in P24 compared with P12, and 2,988 DEGs were detected. After 24 h high-pressure incubation, the number of DEGs remained more than ∼2,000 within 6 h atmospheric pressure recovery (R0.5, R1, R2, R4, and R6). After 6 h recovery, the number of DEGs decreased sharply. The number decreased to fewer than 500 after 12 h recovery (R12, R24, and R48).

### Enrichment Analysis of DEGs

**FIGURE 2:**
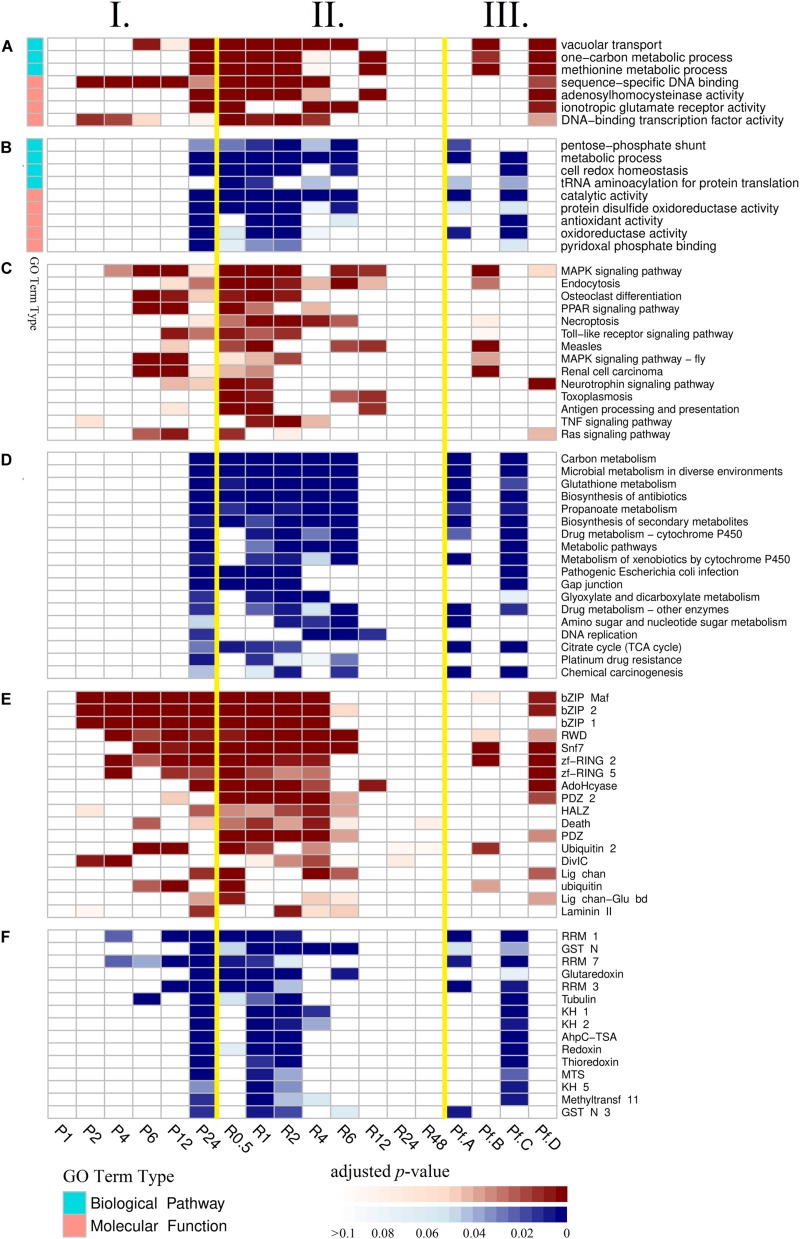
Results of enrichment analysis. **(A)** Gene Ontology enrichment analysis of up-regulated differentially expressed genes (DEGs). **(B)** Gene Ontology enrichment analysis of down-regulated DEGs. **(C)** Kyoto Encyclopedia of Genes and Genomes enrichment analysis of up-regulated DEGs. **(D)** Kyoto Encyclopedia of Genes and Genomes enrichment analysis of down-regulated DEGs. **(E)** Protein family enrichment analysis of up-regulated DEGs. **(F)** Protein family enrichment analysis of down-regulated DEGs. (I) Experimental groups of high-pressure incubations. (II) Experimental groups of atmospheric pressure recoveries. (III) Major expression profiles. Pf.A, profile A; Pf.B, profile B; Pf.C, profile C; Pf.D, profile D.

Gene Ontology (GO), Kyoto Encyclopedia of Genes and Genomes (KEGG), and protein family (Pfam) enrichment analyses of DEGs in time-series experimental groups were used to identify dynamic biological responses to high-pressure environment, and their results are shown via heatmap (Figure 2). Principal component analysis was used to visualize and evaluate the functional similarity between experimental groups according to results of enrichment analysis (Figure 1E, anosim analysis: *R* = 0.894, *p* = 0.002). Most enriched items were detected between the late stages of high-pressure incubation (after 24 h) and the early stages of atmospheric pressure recovery (within 6 h). No items were enriched within 1-h high-pressure incubation and after 24 h atmospheric pressure recovery.

According to GO enrichment analysis of up-regulated DEGs (Figures 2AI,II and Supplementary Table S6), three GO terms belonging to biological process (vacuolar transport, one-carbon metabolic process, and methionine metabolic process) and four GO terms belonging to molecular function (sequence-specific DNA binding, adenosylhomocysteinase activity, ionotropic glutamate receptor activity, and DNA-binding transcription factor activity) were enriched. According to GO enrichment analysis of down-regulated DEGs (Figures 2BI,II and Supplementary Table S7), four GO terms belonging to biological process (pentose phosphate shunt, metabolic process, cell redox homeostasis, and tRNA aminoacylation for protein translation) and five GO terms belonging to molecular function (catalytic activity, protein disulfide oxidoreductase activity, antioxidant activity, oxidoreductase activity, and pyridoxal phosphate binding) were enriched.

According to KEGG enrichment analysis of up-regulated DEGs (Figures 2CI,II and Supplementary Table S8), 14 KEGG pathways were enriched, including mitogen-activated protein kinase (MAPK) signaling pathway, endocytosis, osteoclast differentiation, peroxisome proliferator-activated receptor (PPAR) signaling pathway, necroptosis, and Toll-like receptor signaling pathway. According to KEGG enrichment analysis of down-regulated DEGs (Figures 2DI,II and Supplementary Table S9), 18 KEGG pathways were enriched, including carbon metabolism, microbial metabolism in diverse environments, glutathione metabolism, biosynthesis of antibiotics, propanoate metabolism, and biosynthesis of secondary metabolites.

According to Pfam enrichment analysis of up-regulated DEGs (Figures 2EI,II and Supplementary Table S10), 18 gene families were enriched, including bZIP Maf transcription factor (bZIP Maf), basic region leucine zipper (bZIP 2), bZIP transcription factor (bZIP 1), RWD domain (RWD), Snf7, ring finger domain (zf-RING 2), zinc-RING finger domain (zf-RING 5), and adenosylhomocysteinase (AdoHcyase). According to Pfam enrichment analysis of down-regulated DEGs (Figures 2FI,II and Supplementary Table S11), 15 gene families were enriched, including RNA recognition motif 1 (RRM 1), glutathione S-transferase, N-terminal domain (GST N), RNA recognition motif 7 (RRM 7), glutaredoxin, RNA-binding motif (RRM 3), and tubulin.

### Association Between DEGs and Significantly Enriched Items

**FIGURE 3:**
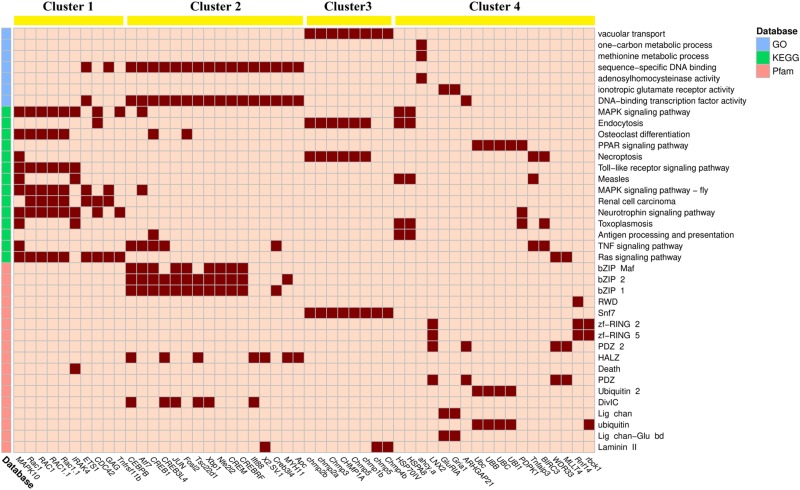
The linkage heatmap of differentially expressed genes (DEGs) and significantly up-regulated enriched items. The cells colored in dark red indicate that their corresponding DEGs are involved in their corresponding enriched items. Clustering results of k-means method are shown on the top of the heatmap.

**FIGURE 4:**
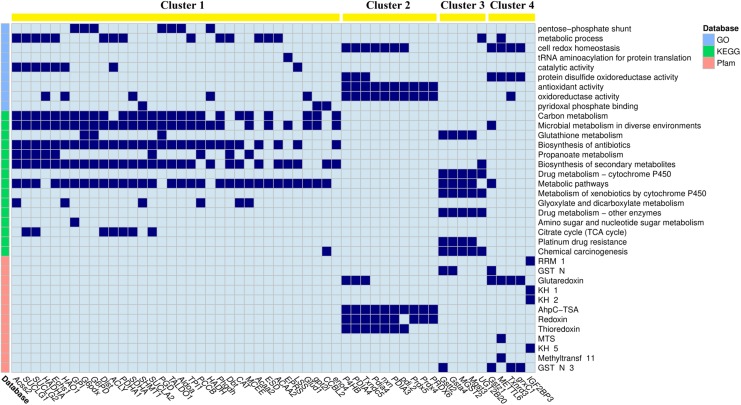
The linkage heatmap of differentially expressed genes (DEGs) and significantly down-regulated enriched items. The cells colored in dark blue indicate that their corresponding DEGs are involved in their corresponding enriched items. Clustering results of k-means method are shown on the top of the heatmap.

To visualize the association between DEGs and enriched items, their linkages are displayed via heatmap (Figures 3, 4). Based on k-means method, up-regulated DEGs and down-regulated DEGs should be divided into four clusters (anosim analysis of up-regulated DEGs clusters: *R* = 0.833, *p* = 0.001; anosim analysis of down-regulated DEGs clusters: *R* = 0.983, *p* = 0.001). The up-regulated DEGs grouped in cluster 1 mainly involved stress response, including mitogen-activated protein kinase 10 (*MAPK10*), ras-related C3 botulinum toxin substrate 1 (*RAC1*), and interleukin 1 receptor–associated kinase 4 (*IRAK4*). The enriched items involved in cluster 1 included MAPK signaling pathway, osteoclast differentiation, and Toll-like receptor signaling pathway. The DEGs in cluster 2 mainly involved transcription, including CCAAT/enhancer-binding protein beta (*CEBPB*), cyclic AMP-dependent transcription factor ATF-7 (*Atf7*), and cyclic AMP-responsive element-binding protein 1 (*CREB1*). The enriched items involved in cluster 2 included sequence-specific DNA binding, DNA-binding transcription factor activity, bZIP Maf, bZIP 1, and bZIP 2. The DEGs in cluster 3 mainly involved endocytosis, including different kinds of charged multivesicular body protein (*chmp*). The enriched items involved in cluster 3 included vacuolar transport, endocytosis, and snf7. The DEGs in cluster 4 mainly involved ubiquitination, including heat shock 70 kDa protein IV (*HSP70IV*), polyubiquitin-C (*UBC*), polyubiquitin-B (*UBB*), and polyubiquitin (*UBI1*). The enriched items involved in cluster 4 included PPAR signaling pathway, zf-RING 2, zf-RING 5, and ubiquitin.

The down-regulated DEGs grouped in cluster 1 mainly involved metabolic processes, especially fatty acid metabolisms and energy metabolisms, including acetyl-coenzyme A synthetase (*Acss2*), succinate-CoA ligase subunit alpha (*SUCLG1*), trifunctional enzyme subunit alpha (*HADHA*), and hydroxyacid oxidase 1 (*HAO1*). The enriched items involved in cluster 1 included pentose-phosphate shunt, metabolic process, carbon metabolism, and microbial metabolism in diverse environments. The DEGs grouped in cluster 2 mainly involved cellular homeostasis, including different kinds of protein disulfide-isomerase (*PDI*). The enriched items involved in cluster 2 included cell redox homeostasis, antioxidant activity, and AhpC-TSA. The DEGs grouped in clusters 3 and 4 mainly involved antioxidation, including different kinds of glutathione S-transferase (*GST*) in cluster 3 and different kinds of glutaredoxin in cluster 4. The enriched items involved in cluster 3 included glutathione metabolism, drug metabolism by cytochrome P450, and metabolism of xenobiotics by cytochrome P450, whereas the enriched items involved in cluster 4 included protein disulfide oxidoreductase activity and glutaredoxin.

### Gene Expression Pattern Analysis

**FIGURE 5:**
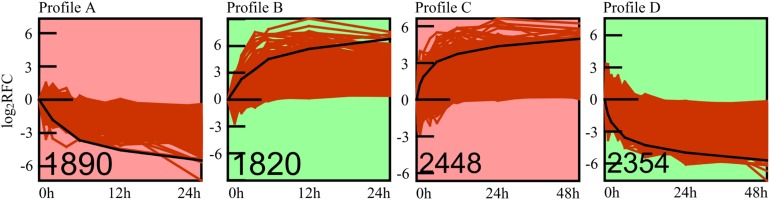
Significant gene expression profiles. The number of genes of each profile is shown at left bottom of each frame. The red curves represent the expression patterns of each gene. The black line represents the model expression pattern. The *y* axis represents log2 (RFC) of expression values, and the *x* axis represents the time points. RFC, relative fold change.

To address the expression patterns of genes responding to high pressure, we used Short Time-series Expression Miner (STEM) software in identifying their major expression profiles (Figure 5). Profiles A (1,890 genes, Supplementary Table S12) and B (1,820 genes, Supplementary Table S13) were the two most significant profiles (adjusted *p* < 0.01) of high-pressure incubation experiments, whereas profiles C (2,448 genes, Supplementary Table S14) and D (2,354 genes, Supplementary Table S15) were the two most significant profiles (adjusted *p* < 0.01) of atmospheric pressure recovery experiments. Additionally, the expression patterns of profiles A and D mirrored those of profiles B and C. The gene expression levels of profiles A and D were decreased rapidly at the beginning. The decreasing rate slowed down with the increase of time, and the expression levels were almost stable after ∼12 h. The gene expression levels of profiles B and C were increased rapidly at the beginning. The increasing rate slowed down with the increase of time, and the expression levels were almost stable after ∼12 h. Gene Ontology, KEGG, and Pfam enrichment analyses were also applied to these four major expression profiles: 62% (18 in 29) significantly enriched items of profile A, and 70% (28 in 40) of profile C was consistent with those significantly down-regulated enriched items, whereas 87.5% (seven in eight) significantly enriched items of profile B, and 75% (12 in 16) of profile D was consistent with those significantly up-regulated enriched items, suggesting that profiles A, B, C, and D could represent the major expression patterns of most DEGs involved in high-pressure response. The adjusted *p* values of these enriched items are displayed via heatmap (Figure 2III).

## Discussion

Compared with single-time-point design, the time-series design enables a better description of organismal dynamic regulation processes. The time-series designs in this study included two opposite processes: the high-pressure incubations used to understand how *A. japonicus* respond to high-pressure environment in a time series and the atmospheric recoveries used to reveal how long the molecular responses to high pressure would last. Moreover, these time-series designs were used to evaluate the relation between sampling time and differential expression caused by hydrostatic pressure changes, which can provide reference for the deep-sea benthic invertebrate sampling work.

Our results showed that major gene expression changes occurred during 4–6 h after hydrostatic pressure was changed. Hierarchical clustering showed that the expression patterns of groups within 4 h high-pressure incubation were similar, and groups within 6 h atmospheric pressure recovery were similar. The results of DESeq2 consistently showed that the number of DEGs increased sharply after 4 h high-pressure incubation and decreased sharply after 6 h atmospheric pressure recovery. However, STEM results showed that most of these genes did not respond to pressure changes with a time lag of 4–6 h but responded to pressure changes at the beginning of pressure changes. These results indicated that although molecular responses occur once hydrostatic pressure is changed, these responses cannot be detected by DESeq2 within 4 h. Compared with quantitative responses, functional responses were slower. Although biological processes started to respond after 2 h incubation, main functional responses occurred after 24 h. We assume that the hardest periods of high-pressure incubated individuals are the first 2–4 h because the effects of high pressure occur once the pressure is elevated, but the significant regulations on transcription level will occur after 2–4 h.

It is hypothesized that the invasions into bathyal and abyssal depths primarily occurred via isothermal water columns (Young et al., 1997; Thatje et al., 2005; Brown and Thatje, 2014). Therefore, although deep-sea environments are characterized by high pressure and low temperature, high pressure could be the first barrier preventing the migration of shallow-water benthic invertebrates to deep sea. The overall effect of pressure on biological systems is reduction but not complete inhibition of the activity. This decrease may be overcome by increasing the concentration of certain components. Fine tuning of gene expression will play an important role in pressure environments lower than 40 MPa (Oger and Jebbar, 2010). The up-regulated biological processes of *A. japonicus* at high-pressure condition included transcription, ubiquitination, endocytosis, stress response, methylation regulation, and transmembrane transportation. Some of them were also reported to be important in deep-sea adaptation, such as stress response (Zheng et al., 2017) and transmembrane transportation (Somero, 1998).

High pressure stabilizes DNA hydrogen bonds, impeding the double- to single-strand transition (Macgregor, 2002). In addition, the activities of shallow-water transmembrane proteins are strongly inhibited by high pressure (Chong et al., 1985). The up-regulation of transcription and transmembrane transportation could counteract these effects. High pressure also causes the denaturizing of protein structures (Balny et al., 2002; Northrop, 2002). Thus, stress response is induced by high pressure, and these responses usually result in several cellular changes to remit the environmental stress. In addition, ubiquitination and endocytosis could collaborate with each other and regulate the elimination of misfolded proteins. We also found genes annotated as adenosylhomocysteinase A (*ahcy-a*) significantly up-regulated in our results, which caused the significant enrichment of items involving methylation (Yang et al., 2003), such as one-carbon metabolic process, methionine metabolic process, and adenosylhomocysteinase activity. The up-regulation of methylation indicated that the modification of macromolecule may be important for *A. japonicus* to acclimatize to high-pressure condition.

The up-regulated biological processes did not respond to high pressure simultaneously. Transcription was the most sensitive biological process responding to pressure exposure. Transcription factors containing bZIP domains were significantly enriched among up-regulated genes after 2 h high-pressure incubation. The following biological process was ubiquitination. Genes contained zf-RING domains, including different kinds of E3 ubiquitin-protein ligase, were significantly up-regulated after 4 h incubation. Then, the biological processes including stress response and endocytosis were identified. Their related enriched items, including MAPK signaling pathway and snf7, were significantly enriched among up-regulated genes after 6 h incubation. The other up-regulated processes, including methylation regulation (enriched items: adenosylhomocysteinase activity) and transmembrane transportation (enriched items: ionotropic glutamate receptor activity), were significantly enriched after 24 h incubation. Transcription factors are proteins that control the rate of transcription of genetic information from DNA to mRNA via binding to specific binding sites (Latchman, 1997). Thus, the up-regulation of transcription factors may be the inducement of the following up-regulated processes.

The down-regulated enriched items mainly involved fatty acid metabolisms, energy metabolisms, antioxidation, and cellular homeostasis. However, several of these processes were reported important for acclimatization and adaptation to high-pressure environment in other studies (Lan et al., 2017; Zhang et al., 2017; Chen et al., 2019; Wang et al., 2019). In fact, many up-regulated DEGs involved in antioxidation and cellular homeostasis were also detected in our results, and several up-regulated biological processes, such as stress response and endocytosis, are also related to homeostasis maintenance. We assume that not all proteins involving antioxidation or cellular homeostasis function well at high pressure, and only genes encoding efficient proteins are to be expressed. This phenomenon was also described in *Photobacterium profundum* strain SS9 whose porin OmpL only expressed at atmospheric pressure, whereas the porin OmpH only expressed at high-pressure environment (Bartlett et al., 1989).

After high-pressure incubation, the transcriptional effects of pressure exposure remained for approximately 6 h, and most biological processes were no longer enriched after 6 h atmospheric pressure recovery. Ten samples that were high-pressure incubated for 24 h survived at atmospheric pressure until the end of this project (≥2 months), and normal behaviors were observed. Although no item was further significantly enriched, 177–350 DEGs were observed after 24 h recovery, indicating that slight differences between R48 and P0 remained.

It is a consensus that gene expression status is highly influenced by environments. Therefore, immediate RNA preservation treatments are required to minimize variation from unwanted stimuli during collection (Alvarez et al., 2015). However, the samples sometimes cannot be fixed immediately, especially during deep-sea benthic invertebrate sampling work. Thus, the relation between sampling time and differential expression caused by hydrostatic pressure changes needs to be determined. According to time-series atmospheric recovery environments, the total number of DEGs of P0, R0.5, R1, R2, and R4 was similar, decreasing from 2,988 to 2,541. If we regard high pressure as an unwanted stimulus, then this stimulus does not introduce much quantitative variation within 4 h. Additionally, enrichment analyses showed that the biological processes responding to high-pressure exposure started to appear after 2 h incubation and disappeared after 4 h recovery, but main biological responses were detected after 4–6 h. Collectively, treatments after 1 h introduced only few quantitative or functional changes into samples, suggesting that the sea cucumber samples for transcriptomic analysis should be fixed within 1 h if possible. However, only transcriptional factors were enriched among DEGs after 2 h, and all the other biological processes were enriched after 4 h. Therefore, it is also acceptable to fix sea cucumber samples within 4 h when hydrostatic pressure is the mainly changed environmental factor.

## Conclusion

Our results reveal the dynamic transcriptional response of *A. japonicus* to high-pressure environment via time-series designs. Most genes respond to pressure changes at the beginning, and their differentially expressed levels keep increasing within ∼12 h, but these changes are not significant enough to be detected by DESeq2 within 4 h. Transcription is the most sensitive biological process responding to high pressure, which is significantly enriched among up-regulated genes after high-pressure incubation for 2 h. The following biological processes are ubiquitination, endocytosis, and stress response, which are significantly enriched after incubation for 4–6 h. The other biological processes, including methylation regulation and transmembrane transportation, are significantly enriched after 24 h incubation. After 24 h high-pressure incubation, all these biological processes will last for 4–6 h, and most of them are no longer enriched after 6 h atmospheric pressure recovery. In addition to studying the molecular response to high pressure, our data are used to identify the relation between sampling time and differential expression caused by hydrostatic pressure changes, showing that only few quantitative or functional responses of *A. japonicus* on transcriptional level are introduced by hydrostatic pressure changes after 1 h, and main biological responses are introduced after 4 h. The results suggested that, when hydrostatic pressure is the mainly changed environmental factor, it will be better to fix sea cucumber samples for transcriptomic analysis within 1 h, but 4 h will be also acceptable.

## Materials and Methods

### Sample Collection and Maintenance

All samples used in this study were aquacultural individuals owned by us. Juvenile specimens of *A. japonicus* (length 5 ± 1 cm, weight 3.9 ± 0.5 g) were collected from an aquaculture farm in Shandong, China, on December 2017 and then maintained at an indoor closed recirculating aquacultural system (Zhongkehai, Qingdao, China) at the optimum temperature (15°C) of *A. japonicus* (Han et al., 2016) for 2 weeks to acclimatize to laboratory environments. The sea cucumber *A. japonicus* were reared in aerated filtered seawater (salinity: 34.7–35.3, 1 μm filtered, natural light cycle) and were fed with algae powder (Haijie, Qingdao, China) twice a week; unconsumed food was removed after 24 h via refreshing seawater. Experimental individuals were starved for 3 days prior to high-pressure incubation.

### Time-Series High-Pressure Incubations and Atmospheric Pressure Recoveries

The hydrostatic pressurization system was set to 15°C by using circulating water bath (Tianheng, Zhejiang, China) at least 6 h prior to each experiment. Three individuals were placed inside the pressure chamber (volume ∼20 L, internal diameter 20 cm, internal depth 65 cm) each time and maintained for 1 h to allow acclimatization and recovery from handling stress. Then, the pressure vessel was pressurized at 1 MPa per minute by using hydraulic pump (Ailipu, Zhejiang, China). After the pressure reached 25 MPa (the highest pressure at which *A. japonicus* can 100% survive for 24 h), the individuals were incubated for 1, 2, 4, 6, 12, and 24 h in different experiments. The pressure chamber was sealed and isolated during high-pressure incubation. Once incubation was finished, the pressure was released instantaneously. Then, the individuals were removed from the pressure chamber and snap frozen in liquid nitrogen. The maximum time elapsed between departure from experimental pressure and flash freezing was 3 min. The flash-frozen individuals were stored at −80°C for further use.

In time-series atmospheric pressure recovery experiments, individuals were incubated at 25 MPa for 24 h at first. Then, the individuals were removed from the pressure chamber to the aquacultural system and recovered at atmospheric pressure for 0.5, 1, 2, 4, 6, 12, 24, and 48 h. Once recovered duration was reached, the individuals were snap frozen in liquid nitrogen and stored at −80°C for further use.

The experimental contexts before and after experiments were measured to ascertain the stabilization of seawater qualities. Dissolved oxygen, salinity, and pH value were measured by using YSI Professional Plus (YSI, Yellow Springs, OH, United States). Concentrations of nitrite nitrogen (NO_2_-N), ammoniacal nitrogen (NH_3_-N) and nitric nitrogen (NO_3_-N) were measured by using HACH DR 1900 (Hach, Loveland, CO, United States).

### RNA Extraction and Quality Control

Approximately 50 mg body wall tissue from each individual was used for RNA extraction. The tissue was dissected before melted and immediately transferred into 1 mL of QIAzol (from RNeasy Plus Universal Kit) and homogenized by T10 basic ULTRA-TURRAX (IKA, Staufen, Germany). Total RNA was extracted by RNeasy Plus Universal Kit (Qiagen, Maryland, United States) according to the manufacturer’s protocol. RNA quality and integrity were tested by NanoDrop spectrophotometer (Thermo Fisher Scientific, Shanghai, China) and RNA Nano 6000 Assay Kit of the Agilent Bioanalyzer 2100 system (Agilent Technologies, California, United States), respectively. All samples must meet the following requirements: the 260/280 ratio between 1.8 and 2.1, the 260/230 ratio between 2.0 and 2.4, and the RIN value higher than 6.8.

### Sequencing, Assembly, and Annotation

A total of 15 experimental groups were sequenced. Each of them had three biological duplications except R48, which had two duplications (R48 should have three duplications, but one RNA sample did not meet the quality requirements). A total of 1.5 μg RNA per sample was used for the RNA sample preparations. Sequencing libraries were generated by using NEBNext Ultra RNA Library Prep Kit for Illumina (NEB, Massachusetts, United States), and index codes were added to attribute sequences to each sample. TruSeq PE Cluster Kit v3-cBot-HS (Illumina, California, United States) was used for the clustering of the index-coded samples performed on a cBot Cluster Generation System. Then, the library preparations were sequenced on Illumina Hiseq X, and 150-bp paired-end reads were generated. Clean data were obtained by removing reads containing adapter, reads containing ploy-N, and low-quality reads from raw data.

Two transcriptomes were assembled by using Trinity (Grabherr et al., 2011) with min_kmer_cov set to 2 by default, and all other parameters set default. One assembly based on the clean data of seven groups, including P0, P1, P2, P4, P6, P12, and P24; the other one based on the clean data of 10 groups, including P0, P24, R0.5, R1, R2, R4, R6, R12, R24, and R48. BUSCO (v.3.0.2) was used to estimate the completeness of the final assembly with default settings and using the metazoan orthologs (Simao et al., 2015). All unigenes were annotated in four databases, including Swiss-Prot, GO, KEGG, and Pfam by using diamond (Buchfink et al., 2015), Blast2GO (Conesa et al., 2005), KAAS (Moriya et al., 2007), and HMMER (Eddy and Wheeler, 2019).

### Differential Expression Analysis

Fragments per kilobase per million mapped fragments (FPKMs) and read count were calculated by using RSEM (Li and Dewey, 2011), and FPKMs were converted to transcripts per million (TPMs). Then, the TPMs were normalized by using calcNormFactors function with the TMM method of edgeR R Package (Robinson et al., 2010). The TMM-normalized TPMs were used for further clustering and gene expression pattern analyses. DESeq2 R Package (Love et al., 2014) was used to detect DEGs taking P0 as control group. Only genes with an adjusted *p* < 0.01 and | log2 (fold change)| > 1 were regarded as DEGs. Bar charts and line graphs were used to visualize the number of DEGs and were drawn via GraphPad Prism 7 (GraphPad Software, California, United States).

### Clustering, Enrichment, and Principal Component Analyses

Hierarchical clustering analysis was used to visualize the similarity among experimental groups. Hierarchical clustering analysis was implemented by using hclust function with the ward.D2 method of R software (R Core Team, 2018). The differences among experimental groups were calculated according to scaled normalized TPMs by using vegdist function with the canberra method of vegan R Package (Oksanen et al., 2018). To ascertain the optimum cluster number (k-means), total within sum of square was calculated by using fviz_nbclust function with the wss method of factoextra R Package (Kassambara and Mundt, 2017). Anosim analysis was used to test whether we can reject the null hypothesis that the similarity between groups is greater than or equal to the similarity within the groups, and it was implemented by using vegan R Package (Oksanen et al., 2018).

Gene Ontology, KEGG, and Pfam enrichment analyses (based on Fisher exact test) were implemented to identify enriched biological processes in each experimental group by using enricher function of clusterProfiler R Package (Yu et al., 2012). Up- and down-regulated DEGs were analyzed separately, and only enriched items with an adjusted *p* < 0.01 were regarded as significantly enriched items. The adjusted *p*-values of significantly enriched items detected in at least two experimental groups were visualized via heatmap by using pheatmap R Package (Kolde, 2015). Results of up-regulated DEGs were shown through heatmap colored in red, whereas results of down-regulated were colored in blue. Principal component analysis was implemented by using fviz_pca_ind function of factoextra R Package (Kassambara and Mundt, 2017). The functional differences among experimental groups were calculated according to adjusted *p* values of all significantly enriched items. In addition, the linkages of DEGs and enriched items were also visualized via heatmap. DEGs involved in more than three up-regulated enriched items were shown in red heatmap, whereas DEGs involved in more than four down-regulated enriched items were shown via blue heatmap. The k-means method was also used to cluster these DEGs based on whether they were involved in each significantly enriched item.

### Gene Expression Pattern Analysis

STEM software (Ernst and Bar-Joseph, 2006) was used to identify the major expression patterns of time-series high-pressure incubations and atmospheric pressure recoveries. STEM could use an algorithm that takes advantage of the number of genes being large and the number of time points being few to identify statistically significant temporal expression profiles and the genes associated with these profiles (Ernst et al., 2005). Input gene expression data were TMM normalized TPMs. P0 and P24 were taken as control groups of time-series high-pressure incubations and atmospheric pressure recoveries, respectively. Only expression profiles with an adjusted *p* < 0.01 were regarded as significant profiles. In addition, enrichment analysis was also implemented to major expression profiles.

### RNA-Seq Validation by qPCR

A total of 14 DEGs were employed for qPCR by StepOnePlus Real-Time PCR System (Applied Biosystems, California, United States) to validate the RNA-seq results. Each 25 μL reaction contained 12.5 μL of FastStart Universal SYBR Green Master (Rox) (Roche, Shanghai, China) and 2.5 μL of template cDNA. The primer sequences were designed by Primer Premier 5.0 software (Premier Biosoft International, California, United States). The cDNA library was established by PrimeScript II 1st Strand cDNA Synthesis Kit (Takara, Beijing, China) according to the manufacturer’s standard protocol. The melting curve analysis was performed at the end of each PCR to confirm that only one PCR product was amplified. Relative standard curve method was used for expression level analysis with *cytb* and β*-actin* as internal controls. At last, Pearson correlation coefficients between RNA-seq and qPCR results were calculated by using cor function of R software (R Core Team, 2018).

## Data Availability Statement

The clean sequence data are available from the Sequence Read Archive database of National Center for Biotechnology Information (Bioproject accessions: PRJNA532806 and PRJNA532988).

## Author Contributions

JC and HZ designed this study. JC analyzed data and wrote the manuscript. JC, LL, and YL conducted the experiments. HZ revised the manuscript and supervised the work.

## Conflict of Interest

The authors declare that the research was conducted in the absence of any commercial or financial relationships that could be construed as a potential conflict of interest.

## References

[B1] Alvarez M. Schrey A. W. Richards C. L. (2015). Ten years of transcriptomics in wild populations: what have we learned about their ecology and evolution? *Mol. Ecol.* 24 710–725. 10.1111/mec.13055 25604587

[B2] Balny C. Masson P. Heremans K. (2002). High pressure effects on biological macromolecules: from structural changes to alteration of cellular processes. *Biochim. Biophys. Acta* 1595 3–10. 10.1016/s0167-4838(01)00331-4 11983383

[B3] Bartlett D. Wright M. Yayanos A. A. Silverman M. (1989). Isolation of a gene regulated by hydrostatic pressure in a deep-sea bacterium. *Nature* 342 572–574. 10.1038/342572a0 2479840

[B4] Brown A. Thatje S. (2014). Explaining bathymetric diversity patterns in marine benthic invertebrates and demersal fishes: physiological contributions to adaptation of life at depth. *Biol. Rev.* 89 406–426. 10.1111/brv.12061 24118851 PMC4158864

[B5] Brown A. Thatje S. (2015). The effects of changing climate on faunal depth distributions determine winners and losers. *Glob. Change Biol.* 21 173–180. 10.1111/gcb.12680 25044552 PMC4310292

[B6] Brown A. Thatje S. Morris J. P. Oliphant A. Morgan E. A. Hauton C. (2017). Metabolic costs imposed by hydrostatic pressure constrain bathymetric range in the lithodid crab *Lithodes maja*. *J. Exp. Biol.* 220 3916–3926. 10.1242/jeb.158543 29093188

[B7] Buchfink B. Xie C. Huson D. H. (2015). Fast and sensitive protein alignment using DIAMOND. *Nat. Methods* 12 59–60. 10.1038/nmeth.3176 25402007

[B8] Chen J. Liu H. Cai S. Zhang H. (2019). Comparative transcriptome analysis of *Eogammarus possjeticus* at different hydrostatic pressure and temperature exposures. *Sci. Rep.* 9:3456. 10.1038/s41598-019-39716-y PMC6401005 30837550

[B9] Chong P. L. Fortes P. A. Jameson D. M. (1985). Mechanisms of inhibition of (Na,K)-ATPase by hydrostatic pressure studied with fluorescent probes. *J. Biol. Chem.* 260 14484–14490. 2997210

[B10] Conesa A. Gotz S. Garcia-Gomez J. M. Terol J. Talon M. Robles M. (2005). Blast2GO: a universal tool for annotation, visualization and analysis in functional genomics research. *Bioinformatics* 21 3674–3676. 10.1093/bioinformatics/bti610 16081474

[B11] Cottin D. Brown A. Oliphant A. Mestre N. C. Ravaux J. Shillito B. (2012). Sustained hydrostatic pressure tolerance of the shallow water shrimp *Palaemonetes varians* at different temperatures: insights into the colonisation of the deep sea. *Comp. Biochem. Physiol. A Mol. Integr. Physiol.* 162 357–363. 10.1016/j.cbpa.2012.04.005 22537881

[B12] Derome N. Duchesne P. Bernatchez L. (2006). Parallelism in gene transcription among sympatric lake whitefish (*Coregonus clupeaformis* Mitchill) ecotypes. *Mol. Ecol.* 15 1239–1249. 10.1111/j.1365-294x.2005.02968.x 16626451

[B13] Eddy S. R. Wheeler T. J. (2019). *The HMMER User’s Guide.* Available online at: http://hmmer.org/

[B14] Ernst J. Bar-Joseph Z. (2006). STEM: a tool for the analysis of short time series gene expression data. *BMC Bioinformatics* 7:191. 10.1186/1471-2105-7-191 16597342 PMC1456994

[B15] Ernst J. Nau G. J. Bar-Joseph Z. (2005). Clustering short time series gene expression data. *Bioinformatics* 21(Suppl. 1), i159–i168. 10.1093/bioinformatics/bti1022 15961453

[B16] Grabherr M. G. Haas B. J. Yassour M. Levin J. Z. Thompson D. A. Amit I. (2011). Full-length transcriptome assembly from RNA-Seq data without a reference genome. *Nat. Biotechnol.* 29 644–U130. 10.1038/nbt.1883 21572440 PMC3571712

[B17] Gunbin K. V. Afonnikov D. A. Kolchanov N. A. (2009). Molecular evolution of the hyperthermophilic archaea of the *Pyrococcus* genus: analysis of adaptation to different environmental conditions. *BMC Genomics* 10:639. 10.1186/1471-2164-10-639 20042074 PMC2816203

[B18] Han Q. Keesing J. K. Liu D. (2016). A review of sea cucumber aquaculture, ranching, and stock enhancement in China. *Rev. Fish. Sci. Aquac.* 24 326–341. 10.1080/23308249.2016.1193472

[B19] Jeukens J. Renaut S. St-Cyr J. Nolte A. W. Bernatchez L. (2010). The transcriptomics of sympatric dwarf and normal lake whitefish (*Coregonus clupeaformis* spp., Salmonidae) divergence as revealed by next-generation sequencing. *Mol. Ecol.* 19 5389–5403. 10.1111/j.1365-294X.2010.04934.x 21087448

[B20] Kassambara A. Mundt F. (2017). *factoextra: Extract and Visualize the Results of Multivariate Data Analyses Version 1.0.6.*

[B21] Kolde R. (2015). *pheatmap: Pretty Heatmaps. R package version 1.0.10.*

[B22] Lan Y. Sun J. Tian R. Bartlett D. H. Li R. Wong Y. H. (2017). Molecular adaptation in the world’s deepest-living animal: insights from transcriptome sequencing of the hadal amphipod *Hirondellea gigas*. *Mol. Ecol.* 26 3732–3743. 10.1111/mec.14149 28429829

[B23] Latchman D. S. (1997). Transcription factors: an overview. *Int. J. Biochem. Cell Biol.* 29 1305–1312. 9570129 10.1016/s1357-2725(97)00085-x

[B24] Li B. Dewey C. N. (2011). RSEM: accurate transcript quantification from RNA-Seq data with or without a reference genome. *BMC Bioinformatics* 12:323. 10.1186/1471-2105-12-323 21816040 PMC3163565

[B25] Love M. I. Huber W. Anders S. (2014). Moderated estimation of fold change and dispersion for RNA-seq data with DESeq2. *Genome Biol.* 15:550. 25516281 10.1186/s13059-014-0550-8 PMC4302049

[B26] Macgregor R. B. (2002). The interactions of nucleic acids at elevated hydrostatic pressure. *Biochim. Biophys. Acta* 1595 266–276. 10.1016/s0167-4838(01)00349-1 11983401

[B27] Miller A. K. Kerr A. M. Paulay G. Reich M. Wilson N. G. Carvajal J. I. (2017). Molecular phylogeny of extant *Holothuroidea* (Echinodermata). *Mol. Phylogenet. Evol.* 111 110–131. 10.1016/j.ympev.2017.02.014 28263876

[B28] Moriya Y. Itoh M. Okuda S. Yoshizawa A. C. Kanehisa M. (2007). KAAS: an automatic genome annotation and pathway reconstruction server. *Nucleic Acids Res.* 35 W182–W185. 17526522 10.1093/nar/gkm321 PMC1933193

[B29] Morris J. P. Thatje S. Ravaux J. Shillito B. Fernando D. Hauton C. (2015). Acute combined pressure and temperature exposures on a shallow-water crustacean: novel insights into the stress response and high pressure neurological syndrome. *Comp. Biochem. Physiol. A Mol. Integr. Physiol.* 181 9–17. 10.1016/j.cbpa.2014.10.028 25433335

[B30] Northrop D. B. (2002). Effects of high pressure on enzymatic activity. *Biochim. Biophys. Acta* 1595 71–79. 10.1016/s0167-4838(01)00335-1 11983387

[B31] Oger P. M. Jebbar M. (2010). The many ways of coping with pressure. *Res. Microbiol.* 161 799–809. 10.1016/j.resmic.2010.09.017 21035541

[B32] Oksanen J. Blanchet G. Kindt R. Legendre P. Minchin P. R. O’Hara R. B. (2018). *vegan: Community Ecology Package. R package version 2.5–2.*

[B33] Oliver T. A. Garfield D. A. Manier M. K. Haygood R. Wray G. A. Palumbi S. R. (2010). Whole-genome positive selection and habitat-driven evolution in a shallow and a deep-sea urchin. *Genome Biol. Evol.* 2 800–814. 10.1093/gbe/evq063 20935062 PMC2975446

[B34] Pinsky M. L. Worm B. Fogarty M. J. Sarmiento J. L. Levin S. A. (2013). Marine taxa track local climate velocities. *Science* 341 1239–1242. 10.1126/science.1239352 24031017

[B35] R Core Team (2018). *R: A Language and Environment for Statistical Computing.* Vienna: R Foundation for Statistical Computing.

[B36] Robinson M. D. McCarthy D. J. Smyth G. K. (2010). edgeR: a Bioconductor package for differential expression analysis of digital gene expression data. *Bioinformatics* 26 139–140. 10.1093/bioinformatics/btp616 19910308 PMC2796818

[B37] Simao F. A. Waterhouse R. M. Ioannidis P. Kriventseva E. V. Zdobnov E. M. (2015). BUSCO: assessing genome assembly and annotation completeness with single-copy orthologs. *Bioinformatics* 31 3210–3212. 10.1093/bioinformatics/btv351 26059717

[B38] Smith K. E. Thatje S. (2012). The secret to successful deep-sea invasion: does low temperature hold the key? *PLoS One* 7:e51219. 10.1371/journal.pone.0051219 23227254 PMC3515517

[B39] Somero G. N. (1992). Adaptations to high hydrostatic pressure. *Annu. Rev. Physiol.* 54 557–577. 10.1146/annurev.ph.54.030192.003013 1314046

[B40] Somero G. N. (1998). *Adaptation to Cold and Depth: Contrasts Between Polar and Deep-Sea Animals.* Cambridge, MA: Cambridge University Press, 33–57.

[B41] Thatje S. Casburn L. Calcagno J. A. (2010). Behavioural and respiratory response of the shallow-water hermit crab Pagurus cuanensis to hydrostatic pressure and temperature. *J. Exp. Mar. Biol. Ecol.* 390 22–30. 10.1016/j.jembe.2010.04.028

[B42] Thatje S. Hillenbrand C. D. Larter R. (2005). On the origin of Antarctic marine benthic community structure. *Trends Ecol. Evol.* 20 534–540. 10.1016/j.tree.2005.07.010 16701431

[B43] Wang K. Shen Y. Yang Y. Gan X. Liu G. Hu K. (2019). Morphology and genome of a snailfish from the Mariana Trench provide insights into deep-sea adaptation. *Nat. Ecol. Evol.* 3 823–833. 10.1038/s41559-019-0864-8 30988486

[B44] West-Eberhard M. J. (2003). *Developmental Plasticity and Evolution.* New York, NY: Oxford University Press.

[B45] Xie Z. Jian H. Jin Z. Xiao X. (2018). Enhancing the adaptability of the Deep-Sea Bacterium Shewanella piezotolerans WP3 to high pressure and low temperature by experimental evolution under H2O2 stress. *Appl. Environ. Microbiol.* 84:e02342-17. 10.1128/AEM.02342-17 PMC5812942 29269502

[B46] Yang X. Hu Y. Yin D. H. Turner M. A. Wang M. Borchardt R. T. (2003). Catalytic strategy of S-adenosyl-L-homocysteine hydrolase: transition-state stabilization and the avoidance of abortive reactions. *Biochemistry* 42 1900–1909. 10.1021/bi0262350 12590576

[B47] Young C. M. Tyler P. A. Fenaux L. (1997). Potential for deep sea invasion by Mediterranean shallow water echinoids:pressure and temperature as stage-specific dispersal barriers. *Mar. Ecol. Prog. Ser.* 154 197–209. 10.3354/meps154197

[B48] Yu G. Wang L.-G. Han Y. He Q.-Y. (2012). clusterProfiler: an R package for comparing biological themes among gene clusters. *OMICS J. Integr. Biol.* 16 284–287. 10.1089/omi.2011.0118 22455463 PMC3339379

[B49] Zhang J. Sun Q. L. Luan Z. D. Lian C. Sun L. (2017). Comparative transcriptome analysis of *Rimicaris* sp. reveals novel molecular features associated with survival in deep-sea hydrothermal vent. *Sci. Rep.* 7:2000. 10.1038/s41598-017-02073-9 28515421 PMC5435735

[B50] Zheng P. Wang M. Li C. Sun X. Wang X. Sun Y. (2017). Insights into deep-sea adaptations and host-symbiont interactions: a comparative transcriptome study on *Bathymodiolus mussels* and their coastal relatives. *Mol. Ecol.* 26 5133–5148. 10.1111/mec.14160 28437568

