# Supplementary material for: Molecular Response to High Hydrostatic Pressure: Time-Series Transcriptomic Analysis of Shallow-Water Sea Cucumber Apostichopus japonicus
Source: Front Genet. 2020 Apr 30;11:355. doi: 10.3389/fgene.2020.00355 (PMC7203883; doi:10.3389/fgene.2020.00355)
Supplement: Supplementary file 1 [file Data_Sheet_1.PDF]

Table S1. Water quality before and after high-pressure incubations.

	Dissolved oxygen (mg/L)	salinity	pH value	c(NO_2-N) (mg/L)	c(NH_3-N) (mg/L)	c(NO_3-N) (mg/L)
Before	5.76 ± 0.3	35.0 ± 0.3	8.1 ± 0.1	0.0055 ± 0.0005	0.015 ±0.005	0.015 ± 0.005
After	5.50 ± 0.4	35.0 ± 0.3	8.1 ± 0.1	0.007 ± 0.0005	0.03 ± 0.005	0.015 ± 0.005

Table S2. Quality of sequencing.

Sample	Raw Reads	Clean reads	Clean bases	Error(%)	Q20(%)	Q30(%)	GC(%)
P0_1	76,725,374	75,238,044	11.29G	0.03	95.77	89.66	41.79
P0_2	71,297,964	69,635,202	10.45G	0.03	96.87	91.95	40.76
P0_3	83,571,800	81,695,626	12.25G	0.03	96.84	91.90	41.43
P1_1	107,613,844	105,566,418	15.83G	0.03	96.57	91.38	40.25
P1_2	101,356,504	99,019,518	14.85G	0.03	96.89	91.98	41.12
P1_3	78,233,052	76,934,984	11.54G	0.03	96.93	92.05	40.59
P2_1	75,639,214	74,006,732	11.1G	0.03	96.85	91.91	41.33
P2_2	84,324,228	82,730,392	12.41G	0.03	96.64	91.55	41.79
P2_3	89,909,426	87,951,456	13.19G	0.03	96.98	92.10	41.24
P4_1	69,809,174	68,509,648	10.28G	0.03	96.80	91.75	41.13
P4_2	87,857,686	86,008,116	12.9G	0.03	96.77	91.73	39.61
P4_3	96,318,662	94,808,122	14.22G	0.03	97.23	92.62	42.13
P6_1	88,761,966	86,907,166	13.04G	0.03	96.95	92.12	40.93
P6_2	93,973,122	92,698,276	13.9G	0.03	96.97	92.17	41.70
P6_3	100,253,142	98,754,170	14.81G	0.03	96.70	91.56	41.34
P12_1	89,647,982	88,360,380	13.25G	0.03	96.60	91.41	40.03
P12_2	101,191,822	99,891,628	14.98G	0.03	97.00	92.26	43.84
P12_3	91,919,942	90,574,420	13.59G	0.03	96.76	91.68	41.59
P24_1	114,691,988	112,273,438	16.84G	0.03	96.76	91.74	39.65
P24_2	73,376,304	71,858,404	10.78G	0.03	96.76	91.69	40.22
P24_3	93,598,656	92,010,102	13.8G	0.03	96.80	91.87	41.33
R0.5_1	88,539,592	87,259,396	13.09G	0.03	95.85	89.53	42.29
R0.5_2	93,965,228	92,413,622	13.86G	0.03	96.84	91.88	41.65
R0.5_3	92,975,836	91,171,856	13.68G	0.03	96.87	91.96	41.52
R1_1	81,778,064	80,169,008	12.03G	0.03	96.77	91.76	41.17
R1_2	78,876,692	77,400,992	11.61G	0.03	96.83	91.89	41.21
R1_3	66,858,510	65,781,830	9.87G	0.03	96.87	91.95	41.42
R2_1	99,958,928	98,052,700	14.71G	0.03	96.64	91.45	41.24
R2_2	71,310,262	70,037,818	10.51G	0.03	96.94	92.07	41.32
R2_3	89,357,608	87,788,818	13.17G	0.03	96.97	92.14	41.15
R4_1	99,222,438	97,290,504	14.59G	0.03	96.90	92.00	42.20
R4_2	93,267,674	89,584,446	13.44G	0.03	96.93	92.09	40.31
R4_3	97,465,812	95,067,638	14.26G	0.03	97.01	92.24	43.30
R6_1	93,913,940	91,835,200	13.78G	0.03	97.01	92.26	41.37
R6_2	70,556,116	67,759,334	10.16G	0.03	96.70	91.56	41.04
R6_3	78,308,462	76,752,744	11.51G	0.03	96.98	92.17	40.50
R12_1	84,402,328	82,411,268	12.36G	0.03	96.75	91.73	40.10

R12_2	92,092,436	90,286,908	13.54G	0.03	96.98	92.17	42.92
R12_3	84,999,196	83,246,534	12.49G	0.03	96.72	91.64	40.20
R24_1	93,414,500	91,212,230	13.68G	0.03	96.87	91.96	41.73
R24_2	90,504,268	88,475,142	13.27G	0.03	96.62	91.44	39.83
R24_3	67,394,402	65,896,830	9.88G	0.03	96.88	91.94	41.15
R48_1	84,133,950	82,204,812	12.33G	0.03	97.02	92.24	42.71
R48_2	97,905,230	95,374,488	14.31G	0.03	96.98	92.20	41.13

Table S3. Quality of the assembly based on 7 groups, including P0, P1, P2, P4, P6, P12 and P24.

	Min Length (bp)	Mean Length (bp)	Median Length (bp)	Max Length (bp)	N50 (bp)	N90 (bp)	Total Nucleotides (bp)
Transcripts	201	716	416	25, 415	1, 080	291	527, 254, 626
Genes	201	828	515	25, 415	1, 192	359	488, 782, 028

Table S4. Quality of the assembly based on 10 groups, including P0, P24, R0.5, R1, R2, R4, R6, R12, R24 and R48.

	Min Length (bp)	Mean Length (bp)	Median Length (bp)	Max Length (bp)	N50 (bp)	N90 (bp)	Total Nucleotides (bp)
Transcripts	201	664	391	58, 334	963	276	592, 656, 478
Genes	201	775	487	58, 334	1, 085	341	542, 758, 628

Table S5. Information of the primers used in qPCR analysis and the Pearson correlation coefficients (PCC) between RNA-seq and qPCR results (with cytb and ß-actin as internal control).

Gene name	PCC	5’- 3’ forward primer	5’- 3’ reverse primer
cytb		TTCCCTAGCTTTCTCGTCAGTA	ACATTTGACCTCAAGGCAATAC
ß-actin		ATGAAGGATGGTTGGAAGAGGGTC	TCAAGGAGAAACTGTGTTACGTCGC
AP	0.95	AATGTGGTTGCCAAGTTATGCT	TCGGCATTCAACATTCTTCTTCC
MVP	0.81	GACTCCAGCGATGATGAATAGG	CAAGGACACCAAAACAAAAGAGAAT
CCAAT	0.98	ATGAACACTGATGACCAATCCCTC	TATTGCGGTTAGTTGGGACGAT
E3RN	0.99	GGGGTTGTTATTCAGGGATTGC	CCAAACGAGAGATGATAGAACGGAT
IST1	0.97	AATCATCCCAAACCTTGCCAGTAAC	TCTGGAGGGTATCGTTCCCAAAAGT
S1	0.98	TTCACAGACGAAGGTGGTTGGC	TTAGTTCTGAGGGAGGTTAGGACGC
GR53	0.98	TGAGGAAACCTTGCCGATTGTA	CACCCTGTAAGTCGTGAAGGATGAAC
ZF36	0.99	GGGGTCCGATTCCGTATTCAGT	CGATAGTGTGATATGTACGGCAGAG
MRP	0.95	CGGGTAGGAGACAAGGGAACAC	GGCTTGGACAACCCTTTCACTT
HT	0.97	CCTAAGAAGTCATTATGTCGCTGGT	AAAGCTAGTACCTGGTCATCCTCGT
N1	0.97	CAGATGTAGATGCTGCCGAGGTATT	CGAGCAGCGGTGATTCCAAGTA
NRC	0.93	CAGTGCTGATGTAGAGTGGACGGTA	AGCCACTGCTTATGACCGACCT
CMB	0.99	ACCTCGTCCGTCATTTCCAACA	GGAGCAAGCCAACTACGCCACA
UC	0.99	TGCCAATGTGACAAAGAACCGAGAT	CGAACACGGAAGACTCAATCAACAG

Table S6. Results of GO enrichment analysis of up-regulated differentially expressed genes.

GO ID	GO Term	Swiss-port ID	Gene Name	Gene Description
GO:0007034	vacuolar transport	Q6NVL7	*chmp2b*	Charged multivesicular body protein 2b
GO:0007034	vacuolar transport	Q7T339	*chmp5*	Charged multivesicular body protein 5
GO:0007034	vacuolar transport	Q7ZW25	*chmp2a*	Charged multivesicular body protein 2a
GO:0007034	vacuolar transport	Q8CGS4	*Chmp3*	Charged multivesicular body protein 3
GO:0007034	vacuolar transport	Q9HD42	*CHMP1A*	Charged multivesicular body protein 1a
GO:0007034	vacuolar transport	Q9D8B3	*Chmp4b*	Charged multivesicular body protein 4b
GO:0007034	vacuolar transport	Q9D7S9	*Chmp5*	Charged multivesicular body protein 5
GO:0007034	vacuolar transport	Q6DF27	*chmp1b*	Charged multivesicular body protein 1b
GO:0006730	one-carbon metabolic process	P51893	*ahcy-a*	Adenosylhomocysteinase A
GO:0006555	methionine metabolic process	Q8N972	*ZNF709*	Zinc finger protein 709
GO:0006555	methionine metabolic process	P51893	*ahcy-a*	Adenosylhomocysteinase A
GO:0043565	sequence-specific DNA binding	O95238	*SPDEF*	SAM pointed domain-containing Ets transcription factor
GO:0043565	sequence-specific DNA binding	P05412	*JUN*	Transcription factor AP-1
GO:0043565	sequence-specific DNA binding	O02755	*CEBPB*	CCAAT/enhancer-binding protein beta
GO:0043565	sequence-specific DNA binding	P51145	*Fosl2*	Fos-related antigen 2
GO:0043565	sequence-specific DNA binding	Q9WTP3	*Spdef*	SAM pointed domain-containing Ets transcription factor
GO:0043565	sequence-specific DNA binding	Q60795	*Nfe2l2*	Nuclear factor erythroid 2-related factor 2
GO:0043565	sequence-specific DNA binding	Q32N90	*mbtd1*	MBT domain-containing protein 1
GO:0043565	sequence-specific DNA binding	Q8R0S1	*Atf7*	Cyclic AMP-dependent transcription factor ATF-7
GO:0043565	sequence-specific DNA binding	P79145	*CREM*	cAMP-responsive element modulator
GO:0043565	sequence-specific DNA binding	Q99PI5	*Lpin2*	Phosphatidate phosphatase LPIN2
GO:0043565	sequence-specific DNA binding	P01105	*GAG*	p135Gag-Myb-Ets-transforming protein (Fragment)
GO:0043565	sequence-specific DNA binding	P14921	*ETS1*	Protein C-ets-1

GO:0043565	sequence-specific DNA binding	P27925	*CREB1*	Cyclic AMP-responsive element-binding protein 1
GO:0043565	sequence-specific DNA binding	P08775	*Polr2a*	DNA-directed RNA polymerase II subunit RPB1
GO:0043565	sequence-specific DNA binding	P62501	*Tsc22d1*	TSC22 domain family protein 1
GO:0043565	sequence-specific DNA binding	Q8IUR6	*CREBRF*	CREB3 regulatory factor
GO:0043565	sequence-specific DNA binding	Q61371	*Ift88*	Intraflagellar transport protein 88 homolog
GO:0043565	sequence-specific DNA binding	P39922		Myosin heavy chain, clone 203 (Fragment)
GO:0043565	sequence-specific DNA binding	P41971	*Elk3*	ETS domain-containing protein Elk-3
GO:0043565	sequence-specific DNA binding	P25210	*NFYB*	Nuclear transcription factor Y subunit beta
GO:0043565	sequence-specific DNA binding	P31503	*Pou2f1*	POU domain, class 2, transcription factor 1 (Fragment)
GO:0043565	sequence-specific DNA binding	P70478	*Apc*	Adenomatous polyposis coli protein
GO:0043565	sequence-specific DNA binding	Q5UEM7	*Creb3l4*	Cyclic AMP-responsive element-binding protein 3-like protein 4
GO:0043565	sequence-specific DNA binding	O35426	*Xbp1*	X-box-binding protein 1
GO:0043565	sequence-specific DNA binding	P37234	*pparg*	Peroxisome proliferator-activated receptor gamma
GO:0043565	sequence-specific DNA binding	Q8TEY5	*CREB3L4*	Cyclic AMP-responsive element-binding protein 3-like protein 4
GO:0043565	sequence-specific DNA binding	P35749	*MYH11*	Myosin-11
GO:0043565	sequence-specific DNA binding	Q921Y2	*Imp3*	U3 small nucleolar ribonucleoprotein protein IMP3
GO:0004013	adenosylhomocysteinase activity	P51893	*ahcy-a*	Adenosylhomocysteinase A
GO:0004970	ionotropic glutamate receptor activity	P19439	*KBP*	Probable glutamate receptor
GO:0004970	ionotropic glutamate receptor activity	Q03445	*GluRIA*	Glutamate receptor 1
GO:0004970	ionotropic glutamate receptor activity	Q10914	*glr-2*	Glutamate receptor 2
GO:0004970	ionotropic glutamate receptor activity	P23818	*Gria1*	Glutamate receptor 1
GO:0004970	ionotropic glutamate receptor activity	P26591		Glutamate receptor
GO:0003700	DNA-binding transcription factor activity	P05412	*JUN*	Transcription factor AP-1
GO:0003700	DNA-binding transcription factor activity	O02755	*CEBPB*	CCAAT/enhancer-binding protein beta
GO:0003700	DNA-binding transcription factor activity	P51145	*Fosl2*	Fos-related antigen 2
GO:0003700	DNA-binding transcription factor activity	Q9WTP3	*Spdef*	SAM pointed domain-containing Ets transcription factor

GO:0003700	DNA-binding transcription factor activity	Q60795	*Nfe2l2*	Nuclear factor erythroid 2-related factor 2
GO:0003700	DNA-binding transcription factor activity	Q6F3J0	*NFKB1*	Nuclear factor NF-kappa-B p105 subunit
GO:0003700	DNA-binding transcription factor activity	Q8R0S1	*Atf7*	Cyclic AMP-dependent transcription factor ATF-7
GO:0003700	DNA-binding transcription factor activity	P79145	*CREM*	cAMP-responsive element modulator
GO:0003700	DNA-binding transcription factor activity	P98156	*Vldlr*	Very low-density lipoprotein receptor
GO:0003700	DNA-binding transcription factor activity	Q99PI5	*Lpin2*	Phosphatidate phosphatase LPIN2
GO:0003700	DNA-binding transcription factor activity	P14921	*ETS1*	Protein C-ets-1
GO:0003700	DNA-binding transcription factor activity	P27925	*CREB1*	Cyclic AMP-responsive element-binding protein 1
GO:0003700	DNA-binding transcription factor activity	Q8MUF6		Paramyosin
GO:0003700	DNA-binding transcription factor activity	P62501	*Tsc22d1*	TSC22 domain family protein 1
GO:0003700	DNA-binding transcription factor activity	Q8IUR6	*CREBRF*	CREB3 regulatory factor
GO:0003700	DNA-binding transcription factor activity	Q5T5U3	*ARHGAP21*	Rho GTPase-activating protein 21
GO:0003700	DNA-binding transcription factor activity	Q9WV30	*Nfat5*	Nuclear factor of activated T-cells 5
GO:0003700	DNA-binding transcription factor activity	Q91ZZ5	*Rxfp2*	Relaxin receptor 2
GO:0003700	DNA-binding transcription factor activity	A0JMV4	*rbm5-a*	RNA-binding protein 5-A
GO:0003700	DNA-binding transcription factor activity	Q61371	*Ift88*	Intraflagellar transport protein 88 homolog
GO:0003700	DNA-binding transcription factor activity	P39922		Myosin heavy chain, clone 203 (Fragment)
GO:0003700	DNA-binding transcription factor activity	Q6ZMP0	*THSD4*	Thrombospondin type-1 domain-containing protein 4
GO:0003700	DNA-binding transcription factor activity	P41971	*Elk3*	ETS domain-containing protein Elk-3
GO:0003700	DNA-binding transcription factor activity	P31503	*Pou2f1*	POU domain, class 2, transcription factor 1 (Fragment)
GO:0003700	DNA-binding transcription factor activity	P70478	*Apc*	Adenomatous polyposis coli protein
GO:0003700	DNA-binding transcription factor activity	Q1JQA2	*SMAD1*	Mothers against decapentaplegic homolog 1
GO:0003700	DNA-binding transcription factor activity	P49133	*TPT*	Triose phosphate/phosphate translocator, chloroplastic
GO:0003700	DNA-binding transcription factor activity	Q5UEM7	*Creb3l4*	Cyclic AMP-responsive element-binding protein 3-like protein 4
GO:0003700	DNA-binding transcription factor activity	O35426	*Xbp1*	X-box-binding protein 1
GO:0003700	DNA-binding transcription factor activity	P37234	*pparg*	Peroxisome proliferator-activated receptor gamma

GO:0003700	DNA-binding transcription factor activity	Q8TEY5	*CREB3L4*	Cyclic AMP-responsive element-binding protein 3-like protein 4
GO:0003700	DNA-binding transcription factor activity	P35749	*MYH11*	Myosin-11
GO:0003700	DNA-binding transcription factor activity	P12755	*SKI*	Ski oncogene
GO:0003700	DNA-binding transcription factor activity	Q921Y2	*Imp3*	U3 small nucleolar ribonucleoprotein protein IMP3

Table S7. Results of GO enrichment analysis of down-regulated differentially expressed genes.

GO ID	GO Term	Swiss-port ID	Gene Name	Gene Description
GO:0006098	pentose-phosphate shunt	Q16836	*HADH*	Hydroxyacyl-coenzyme A dehydrogenase, mitochondrial
GO:0006098	pentose-phosphate shunt	Q99L13	*Hibadh*	3-hydroxyisobutyrate dehydrogenase, mitochondrial
GO:0006098	pentose-phosphate shunt	P00349	*PGD*	6-phosphogluconate dehydrogenase, decarboxylating
GO:0006098	pentose-phosphate shunt	Q4R591	*GPI*	Glucose-6-phosphate isomerase
GO:0006098	pentose-phosphate shunt	P00637	*FBP1*	Fructose-1,6-bisphosphatase 1
GO:0006098	pentose-phosphate shunt	Q2TBL6	*TALDO1*	Transaldolase
GO:0006098	pentose-phosphate shunt	P05370	*G6pdx*	Glucose-6-phosphate 1-dehydrogenase
GO:0006098	pentose-phosphate shunt	Q9EQS0	*Taldo1*	Transaldolase
GO:0006098	pentose-phosphate shunt	O55044	*G6PD*	Glucose-6-phosphate 1-dehydrogenase
GO:0006098	pentose-phosphate shunt	Q9Z2F5	*Ctbp1*	C-terminal-binding protein 1
GO:0006098	pentose-phosphate shunt	A2TLM1	*RPIA*	Ribose-5-phosphate isomerase
GO:0006098	pentose-phosphate shunt	Q29492	*G6PD*	Glucose-6-phosphate 1-dehydrogenase
GO:0006098	pentose-phosphate shunt	P05065	*Aldoa*	Fructose-bisphosphate aldolase A
GO:0008152	metabolic process	P42125	*Eci1*	Enoyl-CoA delta isomerase 1, mitochondrial
GO:0008152	metabolic process	P16930	*FAH*	Fumarylacetoacetase
GO:0008152	metabolic process	O77649	*UGT2B20*	UDP-glucuronosyltransferase 2B20
GO:0008152	metabolic process	A6H791	*TRMT61A*	tRNA (adenine(58)-N(1))-methyltransferase catalytic subunit TRMT61A
GO:0008152	metabolic process	O75521	*ECI2*	Enoyl-CoA delta isomerase 2, mitochondrial
GO:0008152	metabolic process	P13437	*Acaa2*	3-ketoacyl-CoA thiolase, mitochondrial
GO:0008152	metabolic process	Q8N6F8	*WBSCR27*	Williams-Beuren syndrome chromosomal region 27 protein
GO:0008152	metabolic process	Q01205	*Dlst*	Dihydrolipoyllysine-residue succinyltransferase component of 2-oxoglutarate dehydrogenase complex, mitochondrial
GO:0008152	metabolic process	Q91YN5	*Uap1*	UDP-N-acetylhexosamine pyrophosphorylase
GO:0008152	metabolic process	F1NB38	*ECHDC1*	Ethylmalonyl-CoA decarboxylase

GO:0008152	metabolic process	Q27128		Bifunctional 3'-phosphoadenosine 5'-phosphosulfate synthase
GO:0008152	metabolic process	Q29554	*HADHA*	Trifunctional enzyme subunit alpha, mitochondrial
GO:0008152	metabolic process	O35083	*Agpat1*	1-acyl-sn-glycerol-3-phosphate acyltransferase alpha
GO:0008152	metabolic process	Q9V0D5	*mdh*	Malate dehydrogenase
GO:0008152	metabolic process	Q5RFD6	*CPM*	Carboxypeptidase M
GO:0008152	metabolic process	P33124	*Acsl6*	Long-chain-fatty-acid--CoA ligase 6
GO:0008152	metabolic process	Q9BWD1	*ACAT2*	Acetyl-CoA acetyltransferase, cytosolic
GO:0008152	metabolic process	Q9WYG0	*TM_0325*	Uncharacterized oxidoreductase TM_0325
GO:0008152	metabolic process	Q8BMF4	*Dlat*	Dihydrolipoyllysine-residue acetyltransferase component of pyruvate dehydrogenase complex, mitochondrial
GO:0008152	metabolic process	Q54ED7	*pks40*	Probable polyketide synthase 40
GO:0008152	metabolic process	O08651	*Phgdh*	D-3-phosphoglycerate dehydrogenase
GO:0008152	metabolic process	Q5E956	*TPI1*	Triosephosphate isomerase
GO:0008152	metabolic process	O24144	*CCOAOMT1*	Caffeoyl-CoA O-methyltransferase 1
GO:0008152	metabolic process	P21953	*BCKDHB*	2-oxoisovalerate dehydrogenase subunit beta, mitochondrial
GO:0008152	metabolic process	A0AK43	*menG*	Demethylmenaquinone methyltransferase
GO:0008152	metabolic process	Q0V9P1	*hnmt*	Histamine N-methyltransferase
GO:0008152	metabolic process	P14604	*Echs1*	Enoyl-CoA hydratase, mitochondrial
GO:0008152	metabolic process	C1C4R8	*bdh2*	3-hydroxybutyrate dehydrogenase type 2
GO:0008152	metabolic process	Q8TCB7	*METTL6*	Methyltransferase-like protein 6
GO:0008152	metabolic process	Q8MJY8	*HPGD*	15-hydroxyprostaglandin dehydrogenase [NAD(+)]
GO:0008152	metabolic process	Q9CZR2	*Naalad2*	N-acetylated-alpha-linked acidic dipeptidase 2
GO:0008152	metabolic process	Q5FYB0	*ARSJ*	Arylsulfatase J
GO:0008152	metabolic process	Q91XL3	*Uxs1*	UDP-glucuronic acid decarboxylase 1
GO:0008152	metabolic process	Q9QXG4	*Acss2*	Acetyl-coenzyme A synthetase, cytoplasmic
GO:0008152	metabolic process	Q9N126	*RDH8*	Retinol dehydrogenase 8

GO:0008152	metabolic process	Q2KHZ9	*GCDH*	Glutaryl-CoA dehydrogenase, mitochondrial
GO:0008152	metabolic process	Q8K354	*Cbr3*	Carbonyl reductase [NADPH] 3
GO:0008152	metabolic process	Q6AZA0	*acat1*	Acetyl-CoA acetyltransferase, mitochondrial
GO:0008152	metabolic process	Q566I1	*ino80d*	INO80 complex subunit D
GO:0008152	metabolic process	P10768	*ESD*	S-formylglutathione hydrolase
GO:0008152	metabolic process	Q04609	*FOLH1*	Glutamate carboxypeptidase 2
GO:0008152	metabolic process	Q9Z2F5	*Ctbp1*	C-terminal-binding protein 1
GO:0008152	metabolic process	P49432	*Pdhb*	Pyruvate dehydrogenase E1 component subunit beta, mitochondrial
GO:0008152	metabolic process	B0UXP9	*impdh2*	Inosine-5'-monophosphate dehydrogenase 2
GO:0008152	metabolic process	Q9Z1G4	*Atp6v0a1*	V-type proton ATPase 116 kDa subunit a isoform 1
GO:0008152	metabolic process	Q9X248	*fabG*	3-oxoacyl-[acyl-carrier-protein] reductase FabG
GO:0008152	metabolic process	P11536	*Eip74EF*	Ecdysone-induced protein 74EF isoform B
GO:0008152	metabolic process	O46629	*HADHB*	Trifunctional enzyme subunit beta, mitochondrial
GO:0008152	metabolic process	Q55E72	*stlA*	Probable polyketide synthase 1
GO:0008152	metabolic process	Q6B855	*TKT*	Transketolase
GO:0008152	metabolic process	Q9YGD2	*SUCLG1*	Succinyl-CoA ligase subunit alpha, mitochondrial (Fragment)
GO:0008152	metabolic process	P53590	*SUCLG2*	Succinyl-CoA ligase [GDP-forming] subunit beta, mitochondrial (Fragment)
GO:0008152	metabolic process	Q55EX9	*DDB_G0268948*	Putative methyltransferase DDB_G0268948
GO:0008152	metabolic process	Q5RF00	*ALDH2*	Aldehyde dehydrogenase, mitochondrial
GO:0008152	metabolic process	P41216	*Acsl1*	Long-chain-fatty-acid--CoA ligase 1
GO:0008152	metabolic process	Q9CY21	*Wbscr22*	Probable 18S rRNA (guanine-N(7))-methyltransferase
GO:0008152	metabolic process	P53396	*ACLY*	ATP-citrate synthase
GO:0008152	metabolic process	A7MB35	*PDHA1*	Pyruvate dehydrogenase E1 component subunit alpha, somatic form, mitochondrial
GO:0008152	metabolic process	P51688	*SGSH*	N-sulphoglucosamine sulphohydrolase
GO:0008152	metabolic process	P33121	*ACSL1*	Long-chain-fatty-acid--CoA ligase 1
GO:0008152	metabolic process	Q2KJC9	*ALDH7A1*	Alpha-aminoadipic semialdehyde dehydrogenase

GO:0008152	metabolic process	Q62901	*Rere*	Arginine-glutamic acid dipeptide repeats protein
GO:0008152	metabolic process	P11046	*LanB1*	Laminin subunit beta-1
GO:0008152	metabolic process	Q6GQ29	*cpq*	Carboxypeptidase Q
GO:0008152	metabolic process	Q9Z244	*Gmpr*	GMP reductase 1
GO:0008152	metabolic process	C4A0D9	*BAP1*	Ubiquitin carboxyl-terminal hydrolase BAP1
GO:0008152	metabolic process	Q6DC37	*hnmt*	Histamine N-methyltransferase
GO:0008152	metabolic process	P53395	*Dbt*	Lipoamide acyltransferase component of branched-chain alpha-keto acid dehydrogenase complex, mitochondrial
GO:0008152	metabolic process	Q8HYL8	*ACOX1*	Peroxisomal acyl-coenzyme A oxidase 1
GO:0008152	metabolic process	O35156	*UGP2*	UTP--glucose-1-phosphate uridylyltransferase
GO:0008152	metabolic process	Q9D0L8	*Rnmt*	mRNA cap guanine-N7 methyltransferase
GO:0008152	metabolic process	Q5RES5	*ACAA2*	3-ketoacyl-CoA thiolase, mitochondrial
GO:0008152	metabolic process	Q9UMZ3	*PTPRQ*	Phosphatidylinositol phosphatase PTPRQ
GO:0008152	metabolic process	P30839	*Aldh3a2*	Fatty aldehyde dehydrogenase
GO:0008152	metabolic process	Q63ZT8	*aldh1l1*	Cytosolic 10-formyltetrahydrofolate dehydrogenase
GO:0008152	metabolic process	Q92058	*ALPL*	Alkaline phosphatase, tissue-nonspecific isozyme
GO:0045454	cell redox homeostasis	P83876	*TXNL4A*	Thioredoxin-like protein 4A
GO:0045454	cell redox homeostasis	P57113	*Gstz1*	Maleylacetoacetate isomerase
GO:0045454	cell redox homeostasis	Q99MD6	*Txnrd3*	Thioredoxin reductase 3
GO:0045454	cell redox homeostasis	Q5RC61	*SH3BGRL3*	SH3 domain-binding glutamic acid-rich-like protein 3
GO:0045454	cell redox homeostasis	Q92J02	*grxC1*	Glutaredoxin-1
GO:0045454	cell redox homeostasis	O18883	*TXNDC9*	Thioredoxin domain-containing protein 9
GO:0045454	cell redox homeostasis	Q9H299	*SH3BGRL3*	SH3 domain-binding glutamic acid-rich-like protein 3
GO:0045454	cell redox homeostasis	Q8R4U2	*P4HB*	Protein disulfide-isomerase
GO:0045454	cell redox homeostasis	Q29RV1	*PDIA4*	Protein disulfide-isomerase A4
GO:0045454	cell redox homeostasis	Q8TCB7	*METTL6*	Methyltransferase-like protein 6

GO:0045454	cell redox homeostasis	P38659	*Pdia4*	Protein disulfide-isomerase A4
GO:0045454	cell redox homeostasis	Q13454	*TUSC3*	Tumor suppressor candidate 3
GO:0045454	cell redox homeostasis	Q6PBM1	*glrx5*	Glutaredoxin-related protein 5, mitochondrial
GO:0045454	cell redox homeostasis	Q91W90	*Txndc5*	Thioredoxin domain-containing protein 5
GO:0045454	cell redox homeostasis	Q9CQM5	*Txndc17*	Thioredoxin domain-containing protein 17
GO:0045454	cell redox homeostasis	Q503L9	*nxn*	Nucleoredoxin
GO:0045454	cell redox homeostasis	Q17770	*pdi-2*	Protein disulfide-isomerase 2
GO:0045454	cell redox homeostasis	Q8JG64	*PDIA3*	Protein disulfide-isomerase A3
GO:0045454	cell redox homeostasis	P55822	*SH3BGR*	SH3 domain-binding glutamic acid-rich protein
GO:0045454	cell redox homeostasis	Q8K2Q9	*Kiaa1598*	Shootin-1
GO:0006418	tRNA aminoacylation for protein translation	P28491	*CALR*	Calreticulin
GO:0006418	tRNA aminoacylation for protein translation	Q1JPX3	*farsa*	Phenylalanine--tRNA ligase alpha subunit
GO:0006418	tRNA aminoacylation for protein translation	P41252	*IARS*	Isoleucine--tRNA ligase, cytoplasmic
GO:0006418	tRNA aminoacylation for protein translation	P47897	*QARS*	Glutamine--tRNA ligase
GO:0006418	tRNA aminoacylation for protein translation	Q9P2J5	*LARS*	Leucine--tRNA ligase, cytoplasmic
GO:0006418	tRNA aminoacylation for protein translation	Q5F408	*CARS*	Cysteine--tRNA ligase, cytoplasmic
GO:0006418	tRNA aminoacylation for protein translation	Q5RBL1	*GARS*	Glycine--tRNA ligase
GO:0006418	tRNA aminoacylation for protein translation	Q04462	*Vars*	Valine--tRNA ligase

GO:0006418	tRNA aminoacylation for protein translation	Q9D0R2	*Tars*	Threonine--tRNA ligase, cytoplasmic
GO:0006418	tRNA aminoacylation for protein translation	Q6PF21	*mars*	Methionine--tRNA ligase, cytoplasmic
GO:0006418	tRNA aminoacylation for protein translation	P07814	*EPRS*	Bifunctional glutamate/proline--tRNA ligase
GO:0006418	tRNA aminoacylation for protein translation	Q3T0B9	*CEBPG*	CCAAT/enhancer-binding protein gamma
GO:0003824	catalytic activity	Q00685	*C3*	Complement C3 (Fragment)
GO:0003824	catalytic activity	P42125	*Eci1*	Enoyl-CoA delta isomerase 1, mitochondrial
GO:0003824	catalytic activity	P16930	*FAH*	Fumarylacetoacetase
GO:0003824	catalytic activity	P00688	*Amy2*	Pancreatic alpha-amylase
GO:0003824	catalytic activity	O75521	*ECI2*	Enoyl-CoA delta isomerase 2, mitochondrial
GO:0003824	catalytic activity	P14618	*PKM*	Pyruvate kinase PKM
GO:0003824	catalytic activity	F6RQL9	*MTAP*	S-methyl-5'-thioadenosine phosphorylase
GO:0003824	catalytic activity	Q02293	*Fntb*	Protein farnesyltransferase subunit beta
GO:0003824	catalytic activity	F1NB38	*ECHDC1*	Ethylmalonyl-CoA decarboxylase
GO:0003824	catalytic activity	P34943	*NDUFA9*	NADH dehydrogenase [ubiquinone] 1 alpha subcomplex subunit 9, mitochondrial
GO:0003824	catalytic activity	Q29554	*HADHA*	Trifunctional enzyme subunit alpha, mitochondrial
GO:0003824	catalytic activity	Q4R4V2	*PPM1G*	Protein phosphatase 1G
GO:0003824	catalytic activity	P33124	*Acsl6*	Long-chain-fatty-acid--CoA ligase 6
GO:0003824	catalytic activity	Q32KX5	*LRRC28*	Leucine-rich repeat-containing protein 28
GO:0003824	catalytic activity	Q9Y289	*SLC5A6*	Sodium-dependent multivitamin transporter
GO:0003824	catalytic activity	Q92122	*pkm*	Pyruvate kinase PKM
GO:0003824	catalytic activity	P21953	*BCKDHB*	2-oxoisovalerate dehydrogenase subunit beta, mitochondrial
GO:0003824	catalytic activity	P14604	*Echs1*	Enoyl-CoA hydratase, mitochondrial

GO:0003824	catalytic activity	Q8NBP0	*TTC13*	Tetratricopeptide repeat protein 13
GO:0003824	catalytic activity	Q8MJY8	*HPGD*	15-hydroxyprostaglandin dehydrogenase [NAD(+)]
GO:0003824	catalytic activity	Q9D4H9	*Phf14*	PHD finger protein 14
GO:0003824	catalytic activity	Q5FYB0	*ARSJ*	Arylsulfatase J
GO:0003824	catalytic activity	Q91XL3	*Uxs1*	UDP-glucuronic acid decarboxylase 1
GO:0003824	catalytic activity	Q13126	*MTAP*	S-methyl-5'-thioadenosine phosphorylase
GO:0003824	catalytic activity	Q9QXG4	*Acss2*	Acetyl-coenzyme A synthetase, cytoplasmic
GO:0003824	catalytic activity	Q99497	*PARK7*	Protein deglycase DJ-1
GO:0003824	catalytic activity	Q8K354	*Cbr3*	Carbonyl reductase [NADPH] 3
GO:0003824	catalytic activity	P49432	*Pdhb*	Pyruvate dehydrogenase E1 component subunit beta, mitochondrial
GO:0003824	catalytic activity	B0UXP9	*impdh2*	Inosine-5'-monophosphate dehydrogenase 2
GO:0003824	catalytic activity	Q9UJM8	*HAO1*	Hydroxyacid oxidase 1
GO:0003824	catalytic activity	Q9X248	*fabG*	3-oxoacyl-[acyl-carrier-protein] reductase FabG
GO:0003824	catalytic activity	Q28GH3	*uba2*	SUMO-activating enzyme subunit 2
GO:0003824	catalytic activity	Q6B855	*TKT*	Transketolase
GO:0003824	catalytic activity	Q9YGD2	*SUCLG1*	Succinyl-CoA ligase subunit alpha, mitochondrial (Fragment)
GO:0003824	catalytic activity	Q8BLN5	*Lss*	Lanosterol synthase
GO:0003824	catalytic activity	P48449	*LSS*	Lanosterol synthase
GO:0003824	catalytic activity	P53590	*SUCLG2*	Succinyl-CoA ligase [GDP-forming] subunit beta, mitochondrial (Fragment)
GO:0003824	catalytic activity	P41216	*Acsl1*	Long-chain-fatty-acid--CoA ligase 1
GO:0003824	catalytic activity	P53396	*ACLY*	ATP-citrate synthase
GO:0003824	catalytic activity	A7Z035	*CLINT1*	Clathrin interactor 1
GO:0003824	catalytic activity	P51688	*SGSH*	N-sulphoglucosamine sulphohydrolase
GO:0003824	catalytic activity	P33121	*ACSL1*	Long-chain-fatty-acid--CoA ligase 1
GO:0003824	catalytic activity	Q8NBX0	*SCCPDH*	Saccharopine dehydrogenase-like oxidoreductase
GO:0003824	catalytic activity	Q9Z244	*Gmpr*	GMP reductase 1

GO:0003824	catalytic activity	Q8K3X2	*TSTA3*	GDP-L-fucose synthase
GO:0003824	catalytic activity	Q92058	*ALPL*	Alkaline phosphatase, tissue-nonspecific isozyme
GO:0015035	protein disulfide oxidoreductase activity	P57113	*Gstz1*	Maleylacetoacetate isomerase
GO:0015035	protein disulfide oxidoreductase activity	Q99MD6	*Txnrd3*	Thioredoxin reductase 3
GO:0015035	protein disulfide oxidoreductase activity	Q5RC61	*SH3BGRL3*	SH3 domain-binding glutamic acid-rich-like protein 3
GO:0015035	protein disulfide oxidoreductase activity	Q92J02	*grxC1*	Glutaredoxin-1
GO:0015035	protein disulfide oxidoreductase activity	Q9H299	*SH3BGRL3*	SH3 domain-binding glutamic acid-rich-like protein 3
GO:0015035	protein disulfide oxidoreductase activity	Q29RV1	*PDIA4*	Protein disulfide-isomerase A4
GO:0015035	protein disulfide oxidoreductase activity	Q8TCB7	*METTL6*	Methyltransferase-like protein 6
GO:0015035	protein disulfide oxidoreductase activity	Q6PBM1	*glrx5*	Glutaredoxin-related protein 5, mitochondrial
GO:0015035	protein disulfide oxidoreductase activity	Q91W90	*Txndc5*	Thioredoxin domain-containing protein 5
GO:0015035	protein disulfide oxidoreductase activity	Q8R4U2	*P4HB*	Protein disulfide-isomerase
GO:0015035	protein disulfide oxidoreductase activity	P55822	*SH3BGR*	SH3 domain-binding glutamic acid-rich protein
GO:0016209	antioxidant activity	Q8R4U2	*P4HB*	Protein disulfide-isomerase
GO:0016209	antioxidant activity	P99029	*Prdx5*	Peroxiredoxin-5, mitochondrial

GO:0016209	antioxidant activity	Q29RV1	*PDIA4*	Protein disulfide-isomerase A4
GO:0016209	antioxidant activity	O08807	*Prdx4*	Peroxiredoxin-4
GO:0016209	antioxidant activity	Q5ZJF4	*PRDX6*	Peroxiredoxin-6
GO:0016209	antioxidant activity	P38659	*Pdia4*	Protein disulfide-isomerase A4
GO:0016209	antioxidant activity	Q91W90	*Txndc5*	Thioredoxin domain-containing protein 5
GO:0016209	antioxidant activity	Q503L9	*nxn*	Nucleoredoxin
GO:0016209	antioxidant activity	Q17770	*pdi-2*	Protein disulfide-isomerase 2
GO:0016209	antioxidant activity	Q8JG64	*PDIA3*	Protein disulfide-isomerase A3
GO:0016491	oxidoreductase activity	O97490	*Mfi2*	Melanotransferrin
GO:0016491	oxidoreductase activity	Q16836	*HADH*	Hydroxyacyl-coenzyme A dehydrogenase, mitochondrial
GO:0016491	oxidoreductase activity	Q9QZU7	*Bbox1*	Gamma-butyrobetaine dioxygenase
GO:0016491	oxidoreductase activity	B1AZI6	*Thoc2*	THO complex subunit 2
GO:0016491	oxidoreductase activity	Q28GH3	*uba2*	SUMO-activating enzyme subunit 2
GO:0016491	oxidoreductase activity	A8E657	*AASS*	Alpha-aminoadipic semialdehyde synthase, mitochondrial
GO:0016491	oxidoreductase activity	Q99L13	*Hibadh*	3-hydroxyisobutyrate dehydrogenase, mitochondrial
GO:0016491	oxidoreductase activity	Q9Z1S0	*Bub1b*	Mitotic checkpoint serine/threonine-protein kinase BUB1 beta
GO:0016491	oxidoreductase activity	P0DMQ6	*SORD*	Sorbitol dehydrogenase
GO:0016491	oxidoreductase activity	Q9NRD9	*DUOX1*	Dual oxidase 1
GO:0016491	oxidoreductase activity	Q29554	*HADHA*	Trifunctional enzyme subunit alpha, mitochondrial
GO:0016491	oxidoreductase activity	Q99MD6	*Txnrd3*	Thioredoxin reductase 3
GO:0016491	oxidoreductase activity	Q9V0D5	*mdh*	Malate dehydrogenase
GO:0016491	oxidoreductase activity	Q5U249	*ahctf1*	Protein ELYS
GO:0016491	oxidoreductase activity	Q6NYG8	*dpyd*	Dihydropyrimidine dehydrogenase [NADP(+)]
GO:0016491	oxidoreductase activity	Q9YHT1	*SDHA*	Succinate dehydrogenase [ubiquinone] flavoprotein subunit, mitochondrial
GO:0016491	oxidoreductase activity	Q9WYG0	*TM_0325*	Uncharacterized oxidoreductase TM_0325
GO:0016491	oxidoreductase activity	P51175	*Ppox*	Protoporphyrinogen oxidase

GO:0016491	oxidoreductase activity	Q02081	*LCC4*	Laccase-4
GO:0016491	oxidoreductase activity	Q6IRI9	*Fmo2*	Dimethylaniline monooxygenase [N-oxide-forming] 2
GO:0016491	oxidoreductase activity	Q8R4U2	*P4HB*	Protein disulfide-isomerase
GO:0016491	oxidoreductase activity	P99029	*Prdx5*	Peroxiredoxin-5, mitochondrial
GO:0016491	oxidoreductase activity	Q0MQG2	*NDUFS1*	NADH-ubiquinone oxidoreductase 75 kDa subunit, mitochondrial
GO:0016491	oxidoreductase activity	Q29RV1	*PDIA4*	Protein disulfide-isomerase A4
GO:0016491	oxidoreductase activity	A6QQU6	*TMEM144*	Transmembrane protein 144
GO:0016491	oxidoreductase activity	O08807	*Prdx4*	Peroxiredoxin-4
GO:0016491	oxidoreductase activity	C1C4R8	*bdh2*	3-hydroxybutyrate dehydrogenase type 2
GO:0016491	oxidoreductase activity	Q28CZ9	*cyb5r4*	Cytochrome b5 reductase 4
GO:0016491	oxidoreductase activity	P26443	*Glud1*	Glutamate dehydrogenase 1, mitochondrial
GO:0016491	oxidoreductase activity	Q8MJY8	*HPGD*	15-hydroxyprostaglandin dehydrogenase [NAD(+)]
GO:0016491	oxidoreductase activity	Q5ZJF4	*PRDX6*	Peroxiredoxin-6
GO:0016491	oxidoreductase activity	Q95LA2	*FMO1*	Dimethylaniline monooxygenase [N-oxide-forming] 1
GO:0016491	oxidoreductase activity	Q91XL3	*Uxs1*	UDP-glucuronic acid decarboxylase 1
GO:0016491	oxidoreductase activity	P38659	*Pdia4*	Protein disulfide-isomerase A4
GO:0016491	oxidoreductase activity	Q9N126	*RDH8*	Retinol dehydrogenase 8
GO:0016491	oxidoreductase activity	A4YGN0	*Msed_1424*	Succinate-semialdehyde dehydrogenase (acetylating)
GO:0016491	oxidoreductase activity	Q8K354	*Cbr3*	Carbonyl reductase [NADPH] 3
GO:0016491	oxidoreductase activity	P15547	*MT-CYB*	Cytochrome b
GO:0016491	oxidoreductase activity	P57080	*Usp25*	Ubiquitin carboxyl-terminal hydrolase 25
GO:0016491	oxidoreductase activity	Q566I1	*ino80d*	INO80 complex subunit D
GO:0016491	oxidoreductase activity	O93257	*XRCC6*	X-ray repair cross-complementing protein 5
GO:0016491	oxidoreductase activity	Q02650	*Pax5*	Paired box protein Pax-5
GO:0016491	oxidoreductase activity	Q91W90	*Txndc5*	Thioredoxin domain-containing protein 5
GO:0016491	oxidoreductase activity	B0UXP9	*impdh2*	Inosine-5'-monophosphate dehydrogenase 2

GO:0016491	oxidoreductase activity	Q9UJM8	*HAO1*	Hydroxyacid oxidase 1
GO:0016491	oxidoreductase activity	Q66HF1	*Ndufs1*	NADH-ubiquinone oxidoreductase 75 kDa subunit, mitochondrial
GO:0016491	oxidoreductase activity	Q9X248	*fabG*	3-oxoacyl-[acyl-carrier-protein] reductase FabG
GO:0016491	oxidoreductase activity	Q503L9	*nxn*	Nucleoredoxin
GO:0016491	oxidoreductase activity	Q5RF00	*ALDH2*	Aldehyde dehydrogenase, mitochondrial
GO:0016491	oxidoreductase activity	Q5REK0	*FMO2*	Dimethylaniline monooxygenase [N-oxide-forming] 2
GO:0016491	oxidoreductase activity	Q8CIY2	*Duox1*	Dual oxidase 1
GO:0016491	oxidoreductase activity	Q17770	*pdi-2*	Protein disulfide-isomerase 2
GO:0016491	oxidoreductase activity	Q8JG64	*PDIA3*	Protein disulfide-isomerase A3
GO:0016491	oxidoreductase activity	Q9R112	*Sqrdl*	Sulfide:quinone oxidoreductase, mitochondrial
GO:0016491	oxidoreductase activity	Q8NBX0	*SCCPDH*	Saccharopine dehydrogenase-like oxidoreductase
GO:0016491	oxidoreductase activity	Q672K1	*Nox3*	NADPH oxidase 3
GO:0016491	oxidoreductase activity	Q2KJC9	*ALDH7A1*	Alpha-aminoadipic semialdehyde dehydrogenase
GO:0016491	oxidoreductase activity	Q9Z244	*Gmpr*	GMP reductase 1
GO:0016491	oxidoreductase activity	C4A0D9	*BAP1*	Ubiquitin carboxyl-terminal hydrolase BAP1
GO:0016491	oxidoreductase activity	Q8BUE4	*Aifm2*	Apoptosis-inducing factor 2
GO:0016491	oxidoreductase activity	P42577		Soma ferritin
GO:0016491	oxidoreductase activity	P30839	*Aldh3a2*	Fatty aldehyde dehydrogenase
GO:0016491	oxidoreductase activity	Q63ZT8	*aldh1l1*	Cytosolic 10-formyltetrahydrofolate dehydrogenase
GO:0030170	pyridoxal phosphate binding	Q3E6S9	*CpNIFS3*	Probable L-cysteine desulfhydrase, chloroplastic
GO:0030170	pyridoxal phosphate binding	Q9Y289	*SLC5A6*	Sodium-dependent multivitamin transporter
GO:0030170	pyridoxal phosphate binding	Q9Z1J3	*Nfs1*	Cysteine desulfurase, mitochondrial
GO:0030170	pyridoxal phosphate binding	Q6NYL5	*gpt2l*	Alanine aminotransferase 2-like
GO:0030170	pyridoxal phosphate binding	P07511	*SHMT1*	Serine hydroxymethyltransferase, cytosolic
GO:0030170	pyridoxal phosphate binding	P32929	*CTH*	Cystathionine gamma-lyase
GO:0030170	pyridoxal phosphate binding	E1BB03	*ZRANB3*	DNA annealing helicase and endonuclease ZRANB3

GO:0030170	pyridoxal phosphate binding	Q80WP8	*Gadl1*	Acidic amino acid decarboxylase GADL1
GO:0030170	pyridoxal phosphate binding	Q6DEB1	*etnppl*	Ethanolamine-phosphate phospho-lyase
GO:0030170	pyridoxal phosphate binding	Q6YP21	*CCBL2*	Kynurenine--oxoglutarate transaminase 3

Table S8. Results of KEGG enrichment analysis of up-regulated differentially expressed genes.

Pathway ID	Pathway Name	Swiss-port ID	Gene Name	Gene Description
ko04010	MAPK signaling pathway	O08727	*Tnfrsf11b*	Tumor necrosis factor receptor superfamily member 11B
ko04010	MAPK signaling pathway	P24523	*GADD45A*	Growth arrest and DNA damage-inducible protein GADD45 alpha
ko04010	MAPK signaling pathway	Q06248	*HSP70IV*	Heat shock 70 kDa protein IV
ko04010	MAPK signaling pathway	Q02789	*Cacna1s*	Voltage-dependent L-type calcium channel subunit alpha-1S
ko04010	MAPK signaling pathway	P49137	*MAPKAPK2*	MAP kinase-activated protein kinase 2
ko04010	MAPK signaling pathway	Q9BY84	*DUSP16*	Dual specificity protein phosphatase 16
ko04010	MAPK signaling pathway	Q8R0S1	*Atf7*	Cyclic AMP-dependent transcription factor ATF-7
ko04010	MAPK signaling pathway	Q6RUV5	*Rac1*	Ras-related C3 botulinum toxin substrate 1
ko04010	MAPK signaling pathway	Q75J93	*cpras1*	Circularly permutated Ras protein 1
ko04010	MAPK signaling pathway	P62999	*RAC1*	Ras-related C3 botulinum toxin substrate 1
ko04010	MAPK signaling pathway	Q06807	*TEK*	Angiopoietin-1 receptor
ko04010	MAPK signaling pathway	Q9NWZ3	*IRAK4*	Interleukin-1 receptor-associated kinase 4
ko04010	MAPK signaling pathway	P62998	*RAC1*	Ras-related C3 botulinum toxin substrate 1
ko04010	MAPK signaling pathway	Q13163	*MAP2K5*	Dual specificity mitogen-activated protein kinase kinase 5
ko04010	MAPK signaling pathway	P63001	*Rac1*	Ras-related C3 botulinum toxin substrate 1
ko04010	MAPK signaling pathway	P48317	*Gadd45a*	Growth arrest and DNA damage-inducible protein GADD45 alpha
ko04010	MAPK signaling pathway	P53779	*MAPK10*	Mitogen-activated protein kinase 10
ko04010	MAPK signaling pathway	O13224	*hspb1*	Heat shock protein beta-1
ko04010	MAPK signaling pathway	Q9Y6W6	*DUSP10*	Dual specificity protein phosphatase 10
ko04010	MAPK signaling pathway	Q05922	*Dusp2*	Dual specificity protein phosphatase 2
ko04010	MAPK signaling pathway	Q90694	*CDC42*	Cell division control protein 42 homolog
ko04010	MAPK signaling pathway	P19120	*HSPA8*	Heat shock cognate 71 kDa protein
ko04010	MAPK signaling pathway	P36993	*Ppm1b*	Protein phosphatase 1B
ko04144	Endocytosis	Q06248	*HSP70IV*	Heat shock 70 kDa protein IV

ko04144	Endocytosis	O14964	*HGS*	Hepatocyte growth factor-regulated tyrosine kinase substrate
ko04144	Endocytosis	Q6NVL7	*chmp2b*	Charged multivesicular body protein 2b
ko04144	Endocytosis	Q8CGS4	*Chmp3*	Charged multivesicular body protein 3
ko04144	Endocytosis	Q8NEZ2	*VPS37A*	Vacuolar protein sorting-associated protein 37A
ko04144	Endocytosis	O95405	*ZFYVE9*	Zinc finger FYVE domain-containing protein 9
ko04144	Endocytosis	Q3T178	*VPS28*	Vacuolar protein sorting-associated protein 28 homolog
ko04144	Endocytosis	Q7ZW25	*chmp2a*	Charged multivesicular body protein 2a
ko04144	Endocytosis	Q9HD42	*CHMP1A*	Charged multivesicular body protein 1a
ko04144	Endocytosis	Q8CFI0	*Nedd4l*	E3 ubiquitin-protein ligase NEDD4-like
ko04144	Endocytosis	Q99LI8	*Hgs*	Hepatocyte growth factor-regulated tyrosine kinase substrate
ko04144	Endocytosis	Q9D7S9	*Chmp5*	Charged multivesicular body protein 5
ko04144	Endocytosis	Q6DF27	*chmp1b*	Charged multivesicular body protein 1b
ko04144	Endocytosis	Q92783	*STAM*	Signal transducing adapter molecule 1
ko04144	Endocytosis	Q9R1D7	*GBF1*	Golgi-specific brefeldin A-resistance guanine nucleotide exchange factor 1
ko04144	Endocytosis	Q9W6C5	*pdcd6ip*	Programmed cell death 6-interacting protein
ko04144	Endocytosis	Q90694	*CDC42*	Cell division control protein 42 homolog
ko04144	Endocytosis	Q9H9H4	*VPS37B*	Vacuolar protein sorting-associated protein 37B
ko04144	Endocytosis	P19120	*HSPA8*	Heat shock cognate 71 kDa protein
ko04144	Endocytosis	Q07113	*Igf2r*	Cation-independent mannose-6-phosphate receptor
ko04380	Osteoclast differentiation	P51145	*Fosl2*	Fos-related antigen 2
ko04380	Osteoclast differentiation	Q6RUV5	*Rac1*	Ras-related C3 botulinum toxin substrate 1
ko04380	Osteoclast differentiation	Q9Y5S8	*NOX1*	NADPH oxidase 1
ko04380	Osteoclast differentiation	O08623	*Sqstm1*	Sequestosome-1
ko04380	Osteoclast differentiation	F1N9Y5	*SYK*	Tyrosine-protein kinase SYK
ko04380	Osteoclast differentiation	P62999	*RAC1*	Ras-related C3 botulinum toxin substrate 1
ko04380	Osteoclast differentiation	P27925	*CREB1*	Cyclic AMP-responsive element-binding protein 1

ko04380	Osteoclast differentiation	P62998	*RAC1*	Ras-related C3 botulinum toxin substrate 1
ko04380	Osteoclast differentiation	Q9HBY0	*NOX3*	NADPH oxidase 3
ko04380	Osteoclast differentiation	P63001	*Rac1*	Ras-related C3 botulinum toxin substrate 1
ko04380	Osteoclast differentiation	P53779	*MAPK10*	Mitogen-activated protein kinase 10
ko04380	Osteoclast differentiation	P13686	*ACP5*	Tartrate-resistant acid phosphatase type 5
ko03320	PPAR signaling pathway	Q63429	*Ubc*	Polyubiquitin-C
ko03320	PPAR signaling pathway	Q5R668	*ACSL3*	Long-chain-fatty-acid--CoA ligase 3
ko03320	PPAR signaling pathway	Q8MKD1	*UBB*	Polyubiquitin-B
ko03320	PPAR signaling pathway	P0CG68	*UBC*	Polyubiquitin-C
ko03320	PPAR signaling pathway	Q92038		Acyl-CoA desaturase
ko03320	PPAR signaling pathway	P0CG73	*UBI1*	Polyubiquitin
ko03320	PPAR signaling pathway	O15530	*PDPK1*	3-phosphoinositide-dependent protein kinase 1
ko03320	PPAR signaling pathway	Q99541	*PLIN2*	Perilipin-2
ko03320	PPAR signaling pathway	O60488	*ACSL4*	Long-chain-fatty-acid--CoA ligase 4
ko04217	Necroptosis	Q6NVL7	*chmp2b*	Charged multivesicular body protein 2b
ko04217	Necroptosis	Q8CGS4	*Chmp3*	Charged multivesicular body protein 3
ko04217	Necroptosis	O08623	*Sqstm1*	Sequestosome-1
ko04217	Necroptosis	Q0PV50	*TLR3*	Toll-like receptor 3
ko04217	Necroptosis	Q7ZW25	*chmp2a*	Charged multivesicular body protein 2a
ko04217	Necroptosis	Q9HD42	*CHMP1A*	Charged multivesicular body protein 1a
ko04217	Necroptosis	Q924T7	*Rnf31*	E3 ubiquitin-protein ligase RNF31
ko04217	Necroptosis	Q9D7S9	*Chmp5*	Charged multivesicular body protein 5
ko04217	Necroptosis	Q6DF27	*chmp1b*	Charged multivesicular body protein 1b
ko04217	Necroptosis	Q96EP0	*RNF31*	E3 ubiquitin-protein ligase RNF31
ko04217	Necroptosis	P53779	*MAPK10*	Mitogen-activated protein kinase 10
ko04217	Necroptosis	Q9Y6F1	*PARP3*	Poly [ADP-ribose] polymerase 3

ko04217	Necroptosis	O46373	*SLC25A4*	ADP/ATP translocase 1
ko04217	Necroptosis	P48962	*Slc25a4*	ADP/ATP translocase 1
ko04217	Necroptosis	P02722	*SLC25A4*	ADP/ATP translocase 1
ko04217	Necroptosis	Q60769	*Tnfaip3*	Tumor necrosis factor alpha-induced protein 3
ko04217	Necroptosis	Q13489	*BIRC3*	Baculoviral IAP repeat-containing protein 3
ko04620	Toll-like receptor signaling pathway	Q6RUV5	*Rac1*	Ras-related C3 botulinum toxin substrate 1
ko04620	Toll-like receptor signaling pathway	P62999	*RAC1*	Ras-related C3 botulinum toxin substrate 1
ko04620	Toll-like receptor signaling pathway	Q0PV50	*TLR3*	Toll-like receptor 3
ko04620	Toll-like receptor signaling pathway	Q9NWZ3	*IRAK4*	Interleukin-1 receptor-associated kinase 4
ko04620	Toll-like receptor signaling pathway	P62998	*RAC1*	Ras-related C3 botulinum toxin substrate 1
ko04620	Toll-like receptor signaling pathway	P63001	*Rac1*	Ras-related C3 botulinum toxin substrate 1
ko04620	Toll-like receptor signaling pathway	P53779	*MAPK10*	Mitogen-activated protein kinase 10
ko05162	Measles	Q06248	*HSP70IV*	Heat shock 70 kDa protein IV
ko05162	Measles	Q9Z1Z1	*Eif2ak3*	Eukaryotic translation initiation factor 2-alpha kinase 3
ko05162	Measles	Q9NWZ3	*IRAK4*	Interleukin-1 receptor-associated kinase 4
ko05162	Measles	Q16611	*BAK1*	Bcl-2 homologous antagonist/killer
ko05162	Measles	P53779	*MAPK10*	Mitogen-activated protein kinase 10
ko05162	Measles	Q60769	*Tnfaip3*	Tumor necrosis factor alpha-induced protein 3
ko05162	Measles	P19120	*HSPA8*	Heat shock cognate 71 kDa protein
ko05162	Measles	O95786	*DDX58*	Probable ATP-dependent RNA helicase DDX58
ko04013	MAPK signaling pathway - fly	Q80U96	*Xpo1*	Exportin-1
ko04013	MAPK signaling pathway - fly	Q8R0S1	*Atf7*	Cyclic AMP-dependent transcription factor ATF-7
ko04013	MAPK signaling pathway - fly	Q6RUV5	*Rac1*	Ras-related C3 botulinum toxin substrate 1
ko04013	MAPK signaling pathway - fly	Q6VAB6	*KSR2*	Kinase suppressor of Ras 2
ko04013	MAPK signaling pathway - fly	P01105	*GAG*	p135Gag-Myb-Ets-transforming protein (Fragment)
ko04013	MAPK signaling pathway - fly	P62999	*RAC1*	Ras-related C3 botulinum toxin substrate 1

ko04013	MAPK signaling pathway - fly	P14921	*ETS1*	Protein C-ets-1
ko04013	MAPK signaling pathway - fly	P62998	*RAC1*	Ras-related C3 botulinum toxin substrate 1
ko04013	MAPK signaling pathway - fly	P63001	*Rac1*	Ras-related C3 botulinum toxin substrate 1
ko04013	MAPK signaling pathway - fly	P53779	*MAPK10*	Mitogen-activated protein kinase 10
ko04013	MAPK signaling pathway - fly	P36993	*Ppm1b*	Protein phosphatase 1B
ko05211	Renal cell carcinoma	Q3B7L5	*FLCN*	Folliculin
ko05211	Renal cell carcinoma	Q6RUV5	*Rac1*	Ras-related C3 botulinum toxin substrate 1
ko05211	Renal cell carcinoma	P01105	*GAG*	p135Gag-Myb-Ets-transforming protein (Fragment)
ko05211	Renal cell carcinoma	P62999	*RAC1*	Ras-related C3 botulinum toxin substrate 1
ko05211	Renal cell carcinoma	P14921	*ETS1*	Protein C-ets-1
ko05211	Renal cell carcinoma	P62998	*RAC1*	Ras-related C3 botulinum toxin substrate 1
ko05211	Renal cell carcinoma	Q2KII4	*TCEB1*	Transcription elongation factor B polypeptide 1
ko05211	Renal cell carcinoma	P63001	*Rac1*	Ras-related C3 botulinum toxin substrate 1
ko05211	Renal cell carcinoma	Q90694	*CDC42*	Cell division control protein 42 homolog
ko04722	Neurotrophin signaling pathway	O08727	*Tnfrsf11b*	Tumor necrosis factor receptor superfamily member 11B
ko04722	Neurotrophin signaling pathway	P62153		Calmodulin-A
ko04722	Neurotrophin signaling pathway	P49137	*MAPKAPK2*	MAP kinase-activated protein kinase 2
ko04722	Neurotrophin signaling pathway	Q6RUV5	*Rac1*	Ras-related C3 botulinum toxin substrate 1
ko04722	Neurotrophin signaling pathway	P62999	*RAC1*	Ras-related C3 botulinum toxin substrate 1
ko04722	Neurotrophin signaling pathway	Q9NWZ3	*IRAK4*	Interleukin-1 receptor-associated kinase 4
ko04722	Neurotrophin signaling pathway	P62998	*RAC1*	Ras-related C3 botulinum toxin substrate 1
ko04722	Neurotrophin signaling pathway	Q13163	*MAP2K5*	Dual specificity mitogen-activated protein kinase kinase 5
ko04722	Neurotrophin signaling pathway	P63001	*Rac1*	Ras-related C3 botulinum toxin substrate 1
ko04722	Neurotrophin signaling pathway	P53779	*MAPK10*	Mitogen-activated protein kinase 10
ko04722	Neurotrophin signaling pathway	O15530	*PDPK1*	3-phosphoinositide-dependent protein kinase 1
ko04722	Neurotrophin signaling pathway	Q90694	*CDC42*	Cell division control protein 42 homolog

ko05145	Toxoplasmosis	Q06248	*HSP70IV*	Heat shock 70 kDa protein IV
ko05145	Toxoplasmosis	Q9NWZ3	*IRAK4*	Interleukin-1 receptor-associated kinase 4
ko05145	Toxoplasmosis	Q62210	*Birc2*	Baculoviral IAP repeat-containing protein 2
ko05145	Toxoplasmosis	P09917	*ALOX5*	Arachidonate 5-lipoxygenase
ko05145	Toxoplasmosis	P53779	*MAPK10*	Mitogen-activated protein kinase 10
ko05145	Toxoplasmosis	O15530	*PDPK1*	3-phosphoinositide-dependent protein kinase 1
ko05145	Toxoplasmosis	P19120	*HSPA8*	Heat shock cognate 71 kDa protein
ko05145	Toxoplasmosis	Q13489	*BIRC3*	Baculoviral IAP repeat-containing protein 3
ko04612	Antigen processing and presentation	Q06248	*HSP70IV*	Heat shock 70 kDa protein IV
ko04612	Antigen processing and presentation	P27925	*CREB1*	Cyclic AMP-responsive element-binding protein 1
ko04612	Antigen processing and presentation	Q9R0J8	*Lgmn*	Legumain
ko04612	Antigen processing and presentation	A6QPN6	*IFI30*	Gamma-interferon-inducible lysosomal thiol reductase
ko04612	Antigen processing and presentation	Q95029	*Cp1*	Cathepsin L
ko04612	Antigen processing and presentation	Q26636		Cathepsin L
ko04612	Antigen processing and presentation	P25210	*NFYB*	Nuclear transcription factor Y subunit beta
ko04612	Antigen processing and presentation	Q8HY81	*CTSS*	Cathepsin S
ko04612	Antigen processing and presentation	Q5E968	*CTSK*	Cathepsin K
ko04612	Antigen processing and presentation	P19120	*HSPA8*	Heat shock cognate 71 kDa protein
ko04668	TNF signaling pathway	O02755	*CEBPB*	CCAAT/enhancer-binding protein beta
ko04668	TNF signaling pathway	Q5RES1	*MMP14*	Matrix metalloproteinase-14
ko04668	TNF signaling pathway	Q8R0S1	*Atf7*	Cyclic AMP-dependent transcription factor ATF-7
ko04668	TNF signaling pathway	P27925	*CREB1*	Cyclic AMP-responsive element-binding protein 1
ko04668	TNF signaling pathway	P20749	*BCL3*	B-cell lymphoma 3 protein
ko04668	TNF signaling pathway	P53779	*MAPK10*	Mitogen-activated protein kinase 10
ko04668	TNF signaling pathway	Q5UEM7	*Creb3l4*	Cyclic AMP-responsive element-binding protein 3-like protein 4
ko04668	TNF signaling pathway	Q60769	*Tnfaip3*	Tumor necrosis factor alpha-induced protein 3

ko04668	TNF signaling pathway	Q8TEY5	*CREB3L4*	Cyclic AMP-responsive element-binding protein 3-like protein 4
ko04668	TNF signaling pathway	Q13489	*BIRC3*	Baculoviral IAP repeat-containing protein 3
ko04014	Ras signaling pathway	O08727	*Tnfrsf11b*	Tumor necrosis factor receptor superfamily member 11B
ko04014	Ras signaling pathway	P62153		Calmodulin-A
ko04014	Ras signaling pathway	Q6RUV5	*Rac1*	Ras-related C3 botulinum toxin substrate 1
ko04014	Ras signaling pathway	Q6VAB6	*KSR2*	Kinase suppressor of Ras 2
ko04014	Ras signaling pathway	P01105	*GAG*	p135Gag-Myb-Ets-transforming protein (Fragment)
ko04014	Ras signaling pathway	Q9C0J8	*WDR33*	pre-mRNA 3' end processing protein WDR33
ko04014	Ras signaling pathway	Q75J93	*cpras1*	Circularly permutated Ras protein 1
ko04014	Ras signaling pathway	P62999	*RAC1*	Ras-related C3 botulinum toxin substrate 1
ko04014	Ras signaling pathway	P14921	*ETS1*	Protein C-ets-1
ko04014	Ras signaling pathway	Q06807	*TEK*	Angiopoietin-1 receptor
ko04014	Ras signaling pathway	P62998	*RAC1*	Ras-related C3 botulinum toxin substrate 1
ko04014	Ras signaling pathway	P63001	*Rac1*	Ras-related C3 botulinum toxin substrate 1
ko04014	Ras signaling pathway	P55196	*MLLT4*	Afadin
ko04014	Ras signaling pathway	P53779	*MAPK10*	Mitogen-activated protein kinase 10
ko04014	Ras signaling pathway	Q9NZL6	*RGL1*	Ral guanine nucleotide dissociation stimulator-like 1
ko04014	Ras signaling pathway	Q90694	*CDC42*	Cell division control protein 42 homolog

Table S9. Results of KEGG enrichment analysis of down-regulated differentially expressed genes.

Pathway ID	Pathway Name	Swiss-port ID	Gene Name	Gene Description
ko01200	Carbon metabolism	P00349	*PGD*	6-phosphogluconate dehydrogenase, decarboxylating
ko01200	Carbon metabolism	Q01205	*Dlst*	Dihydrolipoyllysine-residue succinyltransferase component of 2-oxoglutarate dehydrogenase complex, mitochondrial
ko01200	Carbon metabolism	Q29554	*HADHA*	Trifunctional enzyme subunit alpha, mitochondrial
ko01200	Carbon metabolism	A9UMP7	*eno4*	Enolase-like protein ENO4
ko01200	Carbon metabolism	Q4R591	*GPI*	Glucose-6-phosphate isomerase
ko01200	Carbon metabolism	Q9YHT1	*SDHA*	Succinate dehydrogenase [ubiquinone] flavoprotein subunit, mitochondrial
ko01200	Carbon metabolism	P40927	*ME1*	NADP-dependent malic enzyme
ko01200	Carbon metabolism	Q2TBR0	*PCCB*	Propionyl-CoA carboxylase beta chain, mitochondrial
ko01200	Carbon metabolism	O08651	*Phgdh*	D-3-phosphoglycerate dehydrogenase
ko01200	Carbon metabolism	Q5E956	*TPI1*	Triosephosphate isomerase
ko01200	Carbon metabolism	Q6NYL5	*gpt2l*	Alanine aminotransferase 2-like
ko01200	Carbon metabolism	P07511	*SHMT1*	Serine hydroxymethyltransferase, cytosolic
ko01200	Carbon metabolism	P14604	*Echs1*	Enoyl-CoA hydratase, mitochondrial
ko01200	Carbon metabolism	P26443	*Glud1*	Glutamate dehydrogenase 1, mitochondrial
ko01200	Carbon metabolism	Q2TBL6	*TALDO1*	Transaldolase
ko01200	Carbon metabolism	P05370	*G6pdx*	Glucose-6-phosphate 1-dehydrogenase
ko01200	Carbon metabolism	O97580	*SUCLA2*	Succinyl-CoA ligase [ADP-forming] subunit beta, mitochondrial (Fragment)
ko01200	Carbon metabolism	Q9QXG4	*Acss2*	Acetyl-coenzyme A synthetase, cytoplasmic
ko01200	Carbon metabolism	P04040	*CAT*	Catalase
ko01200	Carbon metabolism	O55044	*G6PD*	Glucose-6-phosphate 1-dehydrogenase
ko01200	Carbon metabolism	P10768	*ESD*	S-formylglutathione hydrolase

ko01200	Carbon metabolism	Q96PE7	*MCEE*	Methylmalonyl-CoA epimerase, mitochondrial
ko01200	Carbon metabolism	Q9UJM8	*HAO1*	Hydroxyacid oxidase 1
ko01200	Carbon metabolism	Q9YGD2	*SUCLG1*	Succinyl-CoA ligase subunit alpha, mitochondrial (Fragment)
ko01200	Carbon metabolism	P53590	*SUCLG2*	Succinyl-CoA ligase [GDP-forming] subunit beta, mitochondrial (Fragment)
ko01200	Carbon metabolism	A7MB35	*PDHA1*	Pyruvate dehydrogenase E1 component subunit alpha, somatic form, mitochondrial
ko01200	Carbon metabolism	P05065	*Aldoa*	Fructose-bisphosphate aldolase A
ko01120	Microbial metabolism in diverse environments	Q16836	*HADH*	Hydroxyacyl-coenzyme A dehydrogenase, mitochondrial
ko01120	Microbial metabolism in diverse environments	P16930	*FAH*	Fumarylacetoacetase
ko01120	Microbial metabolism in diverse environments	P00349	*PGD*	6-phosphogluconate dehydrogenase, decarboxylating
ko01120	Microbial metabolism in diverse environments	Q01205	*Dlst*	Dihydrolipoyllysine-residue succinyltransferase component of 2-oxoglutarate dehydrogenase complex, mitochondrial
ko01120	Microbial metabolism in diverse environments	Q27128		Bifunctional 3'-phosphoadenosine 5'-phosphosulfate synthase
ko01120	Microbial metabolism in diverse environments	Q29554	*HADHA*	Trifunctional enzyme subunit alpha, mitochondrial
ko01120	Microbial metabolism in diverse environments	A9UMP7	*eno4*	Enolase-like protein ENO4
ko01120	Microbial metabolism in diverse environments	Q4R591	*GPI*	Glucose-6-phosphate isomerase
ko01120	Microbial metabolism in diverse environments	Q9YHT1	*SDHA*	Succinate dehydrogenase [ubiquinone] flavoprotein subunit, mitochondrial

ko01120	Microbial metabolism in diverse environments	P40927	*ME1*	NADP-dependent malic enzyme
ko01120	Microbial metabolism in diverse environments	Q2TBR0	*PCCB*	Propionyl-CoA carboxylase beta chain, mitochondrial
ko01120	Microbial metabolism in diverse environments	O08651	*Phgdh*	D-3-phosphoglycerate dehydrogenase
ko01120	Microbial metabolism in diverse environments	Q5E956	*TPI1*	Triosephosphate isomerase
ko01120	Microbial metabolism in diverse environments	Q6NYL5	*gpt2l*	Alanine aminotransferase 2-like
ko01120	Microbial metabolism in diverse environments	P07511	*SHMT1*	Serine hydroxymethyltransferase, cytosolic
ko01120	Microbial metabolism in diverse environments	P14604	*Echs1*	Enoyl-CoA hydratase, mitochondrial
ko01120	Microbial metabolism in diverse environments	P26443	*Glud1*	Glutamate dehydrogenase 1, mitochondrial
ko01120	Microbial metabolism in diverse environments	P57113	*Gstz1*	Maleylacetoacetate isomerase
ko01120	Microbial metabolism in diverse environments	Q2TBL6	*TALDO1*	Transaldolase
ko01120	Microbial metabolism in diverse environments	P05370	*G6pdx*	Glucose-6-phosphate 1-dehydrogenase
ko01120	Microbial metabolism in diverse environments	O97580	*SUCLA2*	Succinyl-CoA ligase [ADP-forming] subunit beta, mitochondrial (Fragment)
ko01120	Microbial metabolism in diverse environments	Q9QXG4	*Acss2*	Acetyl-coenzyme A synthetase, cytoplasmic

ko01120	Microbial metabolism in diverse environments	O55044	*G6PD*	Glucose-6-phosphate 1-dehydrogenase
ko01120	Microbial metabolism in diverse environments	P10768	*ESD*	S-formylglutathione hydrolase
ko01120	Microbial metabolism in diverse environments	Q96PE7	*MCEE*	Methylmalonyl-CoA epimerase, mitochondrial
ko01120	Microbial metabolism in diverse environments	Q9UJM8	*HAO1*	Hydroxyacid oxidase 1
ko01120	Microbial metabolism in diverse environments	Q9YGD2	*SUCLG1*	Succinyl-CoA ligase subunit alpha, mitochondrial (Fragment)
ko01120	Microbial metabolism in diverse environments	P53590	*SUCLG2*	Succinyl-CoA ligase [GDP-forming] subunit beta, mitochondrial (Fragment)
ko01120	Microbial metabolism in diverse environments	P53396	*ACLY*	ATP-citrate synthase
ko01120	Microbial metabolism in diverse environments	A7MB35	*PDHA1*	Pyruvate dehydrogenase E1 component subunit alpha, somatic form, mitochondrial
ko01120	Microbial metabolism in diverse environments	P05065	*Aldoa*	Fructose-bisphosphate aldolase A
ko01120	Microbial metabolism in diverse environments	P07814	*EPRS*	Bifunctional glutamate/proline--tRNA ligase
ko00480	Glutathione metabolism	Q4KLN6	*Rrm2*	Ribonucleoside-diphosphate reductase subunit M2
ko00480	Glutathione metabolism	P00349	*PGD*	6-phosphogluconate dehydrogenase, decarboxylating
ko00480	Glutathione metabolism	P07742	*Rrm1*	Ribonucleoside-diphosphate reductase large subunit
ko00480	Glutathione metabolism	Q61133	*Gstt2*	Glutathione S-transferase theta-2
ko00480	Glutathione metabolism	Q2KJG4	*MGST2*	Microsomal glutathione S-transferase 2
ko00480	Glutathione metabolism	P05370	*G6pdx*	Glucose-6-phosphate 1-dehydrogenase

ko00480	Glutathione metabolism	O55044	*G6PD*	Glucose-6-phosphate 1-dehydrogenase
ko00480	Glutathione metabolism	Q9CPU4	*Mgst3*	Microsomal glutathione S-transferase 3
ko00480	Glutathione metabolism	Q5XGB9	*lap3*	Cytosol aminopeptidase
ko00480	Glutathione metabolism	P24472	*Gsta4*	Glutathione S-transferase A4
ko01130	Biosynthesis of antibiotics	Q16836	*HADH*	Hydroxyacyl-coenzyme A dehydrogenase, mitochondrial
ko01130	Biosynthesis of antibiotics	P21872	*GART*	Trifunctional purine biosynthetic protein adenosine-3
ko01130	Biosynthesis of antibiotics	A8E657	*AASS*	Alpha-aminoadipic semialdehyde synthase, mitochondrial
ko01130	Biosynthesis of antibiotics	P00349	*PGD*	6-phosphogluconate dehydrogenase, decarboxylating
ko01130	Biosynthesis of antibiotics	P13437	*Acaa2*	3-ketoacyl-CoA thiolase, mitochondrial
ko01130	Biosynthesis of antibiotics	Q9WTP6	*Ak2*	Adenylate kinase 2, mitochondrial
ko01130	Biosynthesis of antibiotics	Q02293	*Fntb*	Protein farnesyltransferase subunit beta
ko01130	Biosynthesis of antibiotics	Q01205	*Dlst*	Dihydrolipoyllysine-residue succinyltransferase component of 2-oxoglutarate dehydrogenase complex, mitochondrial
ko01130	Biosynthesis of antibiotics	Q91YN5	*Uap1*	UDP-N-acetylhexosamine pyrophosphorylase
ko01130	Biosynthesis of antibiotics	Q27128		Bifunctional 3'-phosphoadenosine 5'-phosphosulfate synthase
ko01130	Biosynthesis of antibiotics	Q29554	*HADHA*	Trifunctional enzyme subunit alpha, mitochondrial
ko01130	Biosynthesis of antibiotics	A9UMP7	*eno4*	Enolase-like protein ENO4
ko01130	Biosynthesis of antibiotics	Q4R591	*GPI*	Glucose-6-phosphate isomerase
ko01130	Biosynthesis of antibiotics	Q9YHT1	*SDHA*	Succinate dehydrogenase [ubiquinone] flavoprotein subunit, mitochondrial
ko01130	Biosynthesis of antibiotics	Q2TBR0	*PCCB*	Propionyl-CoA carboxylase beta chain, mitochondrial
ko01130	Biosynthesis of antibiotics	O08651	*Phgdh*	D-3-phosphoglycerate dehydrogenase
ko01130	Biosynthesis of antibiotics	Q5E956	*TPI1*	Triosephosphate isomerase
ko01130	Biosynthesis of antibiotics	P07511	*SHMT1*	Serine hydroxymethyltransferase, cytosolic
ko01130	Biosynthesis of antibiotics	P14604	*Echs1*	Enoyl-CoA hydratase, mitochondrial
ko01130	Biosynthesis of antibiotics	Q2TBL6	*TALDO1*	Transaldolase

ko01130	Biosynthesis of antibiotics	P05370	*G6pdx*	Glucose-6-phosphate 1-dehydrogenase
ko01130	Biosynthesis of antibiotics	O97580	*SUCLA2*	Succinyl-CoA ligase [ADP-forming] subunit beta, mitochondrial (Fragment)
ko01130	Biosynthesis of antibiotics	Q9QXG4	*Acss2*	Acetyl-coenzyme A synthetase, cytoplasmic
ko01130	Biosynthesis of antibiotics	P04040	*CAT*	Catalase
ko01130	Biosynthesis of antibiotics	P32929	*CTH*	Cystathionine gamma-lyase
ko01130	Biosynthesis of antibiotics	O55044	*G6PD*	Glucose-6-phosphate 1-dehydrogenase
ko01130	Biosynthesis of antibiotics	O55023	*Impa1*	Inositol monophosphatase 1
ko01130	Biosynthesis of antibiotics	Q9UJM8	*HAO1*	Hydroxyacid oxidase 1
ko01130	Biosynthesis of antibiotics	Q9YGD2	*SUCLG1*	Succinyl-CoA ligase subunit alpha, mitochondrial (Fragment)
ko01130	Biosynthesis of antibiotics	P48449	*LSS*	Lanosterol synthase
ko01130	Biosynthesis of antibiotics	P53590	*SUCLG2*	Succinyl-CoA ligase [GDP-forming] subunit beta, mitochondrial (Fragment)
ko01130	Biosynthesis of antibiotics	P53396	*ACLY*	ATP-citrate synthase
ko01130	Biosynthesis of antibiotics	A7MB35	*PDHA1*	Pyruvate dehydrogenase E1 component subunit alpha, somatic form, mitochondrial
ko01130	Biosynthesis of antibiotics	P53395	*Dbt*	Lipoamide acyltransferase component of branched-chain alpha-keto acid dehydrogenase complex, mitochondrial
ko01130	Biosynthesis of antibiotics	P05065	*Aldoa*	Fructose-bisphosphate aldolase A
ko01130	Biosynthesis of antibiotics	Q5RES5	*ACAA2*	3-ketoacyl-CoA thiolase, mitochondrial
ko00640	Propanoate metabolism	F1NB38	*ECHDC1*	Ethylmalonyl-CoA decarboxylase
ko00640	Propanoate metabolism	Q29554	*HADHA*	Trifunctional enzyme subunit alpha, mitochondrial
ko00640	Propanoate metabolism	Q2TBR0	*PCCB*	Propionyl-CoA carboxylase beta chain, mitochondrial
ko00640	Propanoate metabolism	P14604	*Echs1*	Enoyl-CoA hydratase, mitochondrial
ko00640	Propanoate metabolism	O97580	*SUCLA2*	Succinyl-CoA ligase [ADP-forming] subunit beta, mitochondrial (Fragment)

ko00640	Propanoate metabolism	Q9QXG4	*Acss2*	Acetyl-coenzyme A synthetase, cytoplasmic
ko00640	Propanoate metabolism	Q96PE7	*MCEE*	Methylmalonyl-CoA epimerase, mitochondrial
ko00640	Propanoate metabolism	Q9YGD2	*SUCLG1*	Succinyl-CoA ligase subunit alpha, mitochondrial (Fragment)
ko00640	Propanoate metabolism	P53590	*SUCLG2*	Succinyl-CoA ligase [GDP-forming] subunit beta, mitochondrial (Fragment)
ko00640	Propanoate metabolism	P53395	*Dbt*	Lipoamide acyltransferase component of branched-chain alpha-keto acid dehydrogenase complex, mitochondrial
ko01110	Biosynthesis of secondary metabolites	Q16836	*HADH*	Hydroxyacyl-coenzyme A dehydrogenase, mitochondrial
ko01110	Biosynthesis of secondary metabolites	O77649	*UGT2B20*	UDP-glucuronosyltransferase 2B20
ko01110	Biosynthesis of secondary metabolites	P21872	*GART*	Trifunctional purine biosynthetic protein adenosine-3
ko01110	Biosynthesis of secondary metabolites	A8E657	*AASS*	Alpha-aminoadipic semialdehyde synthase, mitochondrial
ko01110	Biosynthesis of secondary metabolites	P00349	*PGD*	6-phosphogluconate dehydrogenase, decarboxylating
ko01110	Biosynthesis of secondary metabolites	P13437	*Acaa2*	3-ketoacyl-CoA thiolase, mitochondrial
ko01110	Biosynthesis of secondary metabolites	Q9WTP6	*Ak2*	Adenylate kinase 2, mitochondrial
ko01110	Biosynthesis of secondary metabolites	Q01205	*Dlst*	Dihydrolipoyllysine-residue succinyltransferase component of 2-oxoglutarate dehydrogenase complex, mitochondrial
ko01110	Biosynthesis of secondary metabolites	Q29554	*HADHA*	Trifunctional enzyme subunit alpha, mitochondrial
ko01110	Biosynthesis of secondary metabolites	O35083	*Agpat1*	1-acyl-sn-glycerol-3-phosphate acyltransferase alpha
ko01110	Biosynthesis of secondary metabolites	A9UMP7	*eno4*	Enolase-like protein ENO4
ko01110	Biosynthesis of secondary metabolites	Q4R591	*GPI*	Glucose-6-phosphate isomerase
ko01110	Biosynthesis of secondary metabolites	Q9YHT1	*SDHA*	Succinate dehydrogenase [ubiquinone] flavoprotein subunit, mitochondrial
ko01110	Biosynthesis of secondary metabolites	Q6GMK8	*gmppaa*	Mannose-1-phosphate guanyltransferase alpha-A
ko01110	Biosynthesis of secondary metabolites	Q5E956	*TPI1*	Triosephosphate isomerase
ko01110	Biosynthesis of secondary metabolites	O35077	*Gpd1*	Glycerol-3-phosphate dehydrogenase [NAD(+)], cytoplasmic
ko01110	Biosynthesis of secondary metabolites	P07511	*SHMT1*	Serine hydroxymethyltransferase, cytosolic
ko01110	Biosynthesis of secondary metabolites	P14604	*Echs1*	Enoyl-CoA hydratase, mitochondrial

ko01110	Biosynthesis of secondary metabolites	Q8BTZ7	*Gmppb*	Mannose-1-phosphate guanyltransferase beta
ko01110	Biosynthesis of secondary metabolites	O57478	*fech*	Ferrochelatase, mitochondrial
ko01110	Biosynthesis of secondary metabolites	Q2TBL6	*TALDO1*	Transaldolase
ko01110	Biosynthesis of secondary metabolites	P05370	*G6pdx*	Glucose-6-phosphate 1-dehydrogenase
ko01110	Biosynthesis of secondary metabolites	O97580	*SUCLA2*	Succinyl-CoA ligase [ADP-forming] subunit beta, mitochondrial (Fragment)
ko01110	Biosynthesis of secondary metabolites	Q9QXG4	*Acss2*	Acetyl-coenzyme A synthetase, cytoplasmic
ko01110	Biosynthesis of secondary metabolites	P04040	*CAT*	Catalase
ko01110	Biosynthesis of secondary metabolites	O55044	*G6PD*	Glucose-6-phosphate 1-dehydrogenase
ko01110	Biosynthesis of secondary metabolites	Q9UJM8	*HAO1*	Hydroxyacid oxidase 1
ko01110	Biosynthesis of secondary metabolites	Q9YGD2	*SUCLG1*	Succinyl-CoA ligase subunit alpha, mitochondrial (Fragment)
ko01110	Biosynthesis of secondary metabolites	P48449	*LSS*	Lanosterol synthase
ko01110	Biosynthesis of secondary metabolites	P53590	*SUCLG2*	Succinyl-CoA ligase [GDP-forming] subunit beta, mitochondrial (Fragment)
ko01110	Biosynthesis of secondary metabolites	P53396	*ACLY*	ATP-citrate synthase
ko01110	Biosynthesis of secondary metabolites	A7MB35	*PDHA1*	Pyruvate dehydrogenase E1 component subunit alpha, somatic form, mitochondrial
ko01110	Biosynthesis of secondary metabolites	P53395	*Dbt*	Lipoamide acyltransferase component of branched-chain alpha-keto acid dehydrogenase complex, mitochondrial
ko01110	Biosynthesis of secondary metabolites	Q8HYL8	*ACOX1*	Peroxisomal acyl-coenzyme A oxidase 1
ko01110	Biosynthesis of secondary metabolites	P05065	*Aldoa*	Fructose-bisphosphate aldolase A
ko01110	Biosynthesis of secondary metabolites	Q6YP21	*CCBL2*	Kynurenine--oxoglutarate transaminase 3
ko01110	Biosynthesis of secondary metabolites	P07814	*EPRS*	Bifunctional glutamate/proline--tRNA ligase
ko01110	Biosynthesis of secondary metabolites	Q5RES5	*ACAA2*	3-ketoacyl-CoA thiolase, mitochondrial
ko00982	Drug metabolism - cytochrome P450	O77649	*UGT2B20*	UDP-glucuronosyltransferase 2B20
ko00982	Drug metabolism - cytochrome P450	Q61133	*Gstt2*	Glutathione S-transferase theta-2

ko00982	Drug metabolism - cytochrome P450	Q6IRI9	*Fmo2*	Dimethylaniline monooxygenase [N-oxide-forming] 2
ko00982	Drug metabolism - cytochrome P450	Q2KJG4	*MGST2*	Microsomal glutathione S-transferase 2
ko00982	Drug metabolism - cytochrome P450	Q9CPU4	*Mgst3*	Microsomal glutathione S-transferase 3
ko00982	Drug metabolism - cytochrome P450	P24472	*Gsta4*	Glutathione S-transferase A4
ko01100	Metabolic pathways	Q01205	*Dlst*	Dihydrolipoyllysine-residue succinyltransferase component of 2-oxoglutarate dehydrogenase complex, mitochondrial
ko01100	Metabolic pathways	Q9Y673	*ALG5*	Dolichyl-phosphate beta-glucosyltransferase
ko01100	Metabolic pathways	P33124	*Acsl6*	Long-chain-fatty-acid--CoA ligase 6
ko01100	Metabolic pathways	Q9YHT1	*SDHA*	Succinate dehydrogenase [ubiquinone] flavoprotein subunit, mitochondrial
ko01100	Metabolic pathways	P07686	*HEXB*	Beta-hexosaminidase subunit beta
ko01100	Metabolic pathways	Q6GMK8	*gmppaa*	Mannose-1-phosphate guanyltransferase alpha-A
ko01100	Metabolic pathways	P40927	*ME1*	NADP-dependent malic enzyme
ko01100	Metabolic pathways	Q924L9	*FPGS*	Folylpolyglutamate synthase, mitochondrial
ko01100	Metabolic pathways	Q2TBR0	*PCCB*	Propionyl-CoA carboxylase beta chain, mitochondrial
ko01100	Metabolic pathways	Q9Z1J3	*Nfs1*	Cysteine desulfurase, mitochondrial
ko01100	Metabolic pathways	O08651	*Phgdh*	D-3-phosphoglycerate dehydrogenase
ko01100	Metabolic pathways	Q5E956	*TPI1*	Triosephosphate isomerase
ko01100	Metabolic pathways	Q2KJG4	*MGST2*	Microsomal glutathione S-transferase 2
ko01100	Metabolic pathways	Q24439	*Oscp*	ATP synthase subunit O, mitochondrial
ko01100	Metabolic pathways	Q6NYL5	*gpt2l*	Alanine aminotransferase 2-like
ko01100	Metabolic pathways	P07511	*SHMT1*	Serine hydroxymethyltransferase, cytosolic
ko01100	Metabolic pathways	P14604	*Echs1*	Enoyl-CoA hydratase, mitochondrial
ko01100	Metabolic pathways	P26443	*Glud1*	Glutamate dehydrogenase 1, mitochondrial
ko01100	Metabolic pathways	Q90744	*NAGA*	Alpha-N-acetylgalactosaminidase
ko01100	Metabolic pathways	Q8BTZ7	*Gmppb*	Mannose-1-phosphate guanyltransferase beta
ko01100	Metabolic pathways	O57478	*fech*	Ferrochelatase, mitochondrial

ko01100	Metabolic pathways	P57113	*Gstz1*	Maleylacetoacetate isomerase
ko01100	Metabolic pathways	Q14117	*DPYS*	Dihydropyrimidinase
ko01100	Metabolic pathways	Q2TBL6	*TALDO1*	Transaldolase
ko01100	Metabolic pathways	P05370	*G6pdx*	Glucose-6-phosphate 1-dehydrogenase
ko01100	Metabolic pathways	O97580	*SUCLA2*	Succinyl-CoA ligase [ADP-forming] subunit beta, mitochondrial (Fragment)
ko01100	Metabolic pathways	Q9QXG4	*Acss2*	Acetyl-coenzyme A synthetase, cytoplasmic
ko01100	Metabolic pathways	P04040	*CAT*	Catalase
ko01100	Metabolic pathways	P32929	*CTH*	Cystathionine gamma-lyase
ko01100	Metabolic pathways	Q61133	*Gstt2*	Glutathione S-transferase theta-2
ko01100	Metabolic pathways	Q02368	*NDUFB7*	NADH dehydrogenase [ubiquinone] 1 beta subcomplex subunit 7
ko01100	Metabolic pathways	P13437	*Acaa2*	3-ketoacyl-CoA thiolase, mitochondrial
ko01100	Metabolic pathways	Q5R6R5	*CMAS*	N-acylneuraminate cytidylyltransferase
ko01100	Metabolic pathways	P17276	*Hn*	Protein henna
ko01100	Metabolic pathways	Q8TDX6	*CSGALNACT1*	Chondroitin sulfate N-acetylgalactosaminyltransferase 1
ko01100	Metabolic pathways	O55044	*G6PD*	Glucose-6-phosphate 1-dehydrogenase
ko01100	Metabolic pathways	O60760	*HPGDS*	Hematopoietic prostaglandin D synthase
ko01100	Metabolic pathways	Q71S46	*Atp5g3*	ATP synthase F(0) complex subunit C3, mitochondrial
ko01100	Metabolic pathways	P15547	*MT-CYB*	Cytochrome b
ko01100	Metabolic pathways	P10768	*ESD*	S-formylglutathione hydrolase
ko01100	Metabolic pathways	Q9ERS2	*Ndufa13*	NADH dehydrogenase [ubiquinone] 1 alpha subcomplex subunit 13
ko01100	Metabolic pathways	Q96PE7	*MCEE*	Methylmalonyl-CoA epimerase, mitochondrial
ko01100	Metabolic pathways	O55023	*Impa1*	Inositol monophosphatase 1
ko01100	Metabolic pathways	Q25117		ATP synthase subunit beta, mitochondrial
ko01100	Metabolic pathways	Q9UJM8	*HAO1*	Hydroxyacid oxidase 1
ko01100	Metabolic pathways	Q9Z1G4	*Atp6v0a1*	V-type proton ATPase 116 kDa subunit a isoform 1

ko01100	Metabolic pathways	Q9CPU4	*Mgst3*	Microsomal glutathione S-transferase 3
ko01100	Metabolic pathways	P54363	*Aprt*	Adenine phosphoribosyltransferase
ko01100	Metabolic pathways	Q9YGD2	*SUCLG1*	Succinyl-CoA ligase subunit alpha, mitochondrial (Fragment)
ko01100	Metabolic pathways	P48449	*LSS*	Lanosterol synthase
ko01100	Metabolic pathways	Q5XGB9	*lap3*	Cytosol aminopeptidase
ko01100	Metabolic pathways	P53590	*SUCLG2*	Succinyl-CoA ligase [GDP-forming] subunit beta, mitochondrial (Fragment)
ko01100	Metabolic pathways	Q6AZN6	*pik3c3*	Phosphatidylinositol 3-kinase catalytic subunit type 3
ko01100	Metabolic pathways	P41216	*Acsl1*	Long-chain-fatty-acid--CoA ligase 1
ko01100	Metabolic pathways	Q91WD5	*Ndufs2*	NADH dehydrogenase [ubiquinone] iron-sulfur protein 2, mitochondrial
ko01100	Metabolic pathways	P24472	*Gsta4*	Glutathione S-transferase A4
ko01100	Metabolic pathways	Q6DEB1	*etnppl*	Ethanolamine-phosphate phospho-lyase
ko01100	Metabolic pathways	P53396	*ACLY*	ATP-citrate synthase
ko01100	Metabolic pathways	A7MB35	*PDHA1*	Pyruvate dehydrogenase E1 component subunit alpha, somatic form, mitochondrial
ko01100	Metabolic pathways	Q0MQD0	*NDUFB3*	NADH dehydrogenase [ubiquinone] 1 beta subcomplex subunit 3
ko01100	Metabolic pathways	Q02380	*NDUFB5*	NADH dehydrogenase [ubiquinone] 1 beta subcomplex subunit 5, mitochondrial
ko01100	Metabolic pathways	P21872	*GART*	Trifunctional purine biosynthetic protein adenosine-3
ko01100	Metabolic pathways	Q6PF21	*mars*	Methionine--tRNA ligase, cytoplasmic
ko01100	Metabolic pathways	Q5RAP9	*ATP5G2*	ATP synthase F(0) complex subunit C2, mitochondrial
ko01100	Metabolic pathways	P05496	*ATP5G1*	ATP synthase F(0) complex subunit C1, mitochondrial
ko01100	Metabolic pathways	P13619	*ATP5F1*	ATP synthase F(0) complex subunit B1, mitochondrial
ko01100	Metabolic pathways	P18293	*Npr1*	Atrial natriuretic peptide receptor 1
ko01100	Metabolic pathways	P53395	*Dbt*	Lipoamide acyltransferase component of branched-chain alpha-keto acid dehydrogenase complex, mitochondrial

ko01100	Metabolic pathways	Q8HYL8	*ACOX1*	Peroxisomal acyl-coenzyme A oxidase 1
ko01100	Metabolic pathways	Q8K3X2	*TSTA3*	GDP-L-fucose synthase
ko01100	Metabolic pathways	P51688	*SGSH*	N-sulphoglucosamine sulphohydrolase
ko01100	Metabolic pathways	P05065	*Aldoa*	Fructose-bisphosphate aldolase A
ko01100	Metabolic pathways	Q6YP21	*CCBL2*	Kynurenine--oxoglutarate transaminase 3
ko01100	Metabolic pathways	P07814	*EPRS*	Bifunctional glutamate/proline--tRNA ligase
ko01100	Metabolic pathways	P21571	*Atp5j*	ATP synthase-coupling factor 6, mitochondrial
ko01100	Metabolic pathways	Q2I6J0	*inppl1b*	Phosphatidylinositol 3,4,5-trisphosphate 5-phosphatase 2B
ko01100	Metabolic pathways	Q5RES5	*ACAA2*	3-ketoacyl-CoA thiolase, mitochondrial
ko01100	Metabolic pathways	Q4R591	*GPI*	Glucose-6-phosphate isomerase
ko01100	Metabolic pathways	Q28852	*ATP5L*	ATP synthase subunit g, mitochondrial
ko00980	Metabolism of xenobiotics by cytochrome P450	O77649	*UGT2B20*	UDP-glucuronosyltransferase 2B20
ko00980	Metabolism of xenobiotics by cytochrome P450	Q61133	*Gstt2*	Glutathione S-transferase theta-2
ko00980	Metabolism of xenobiotics by cytochrome P450	Q2KJG4	*MGST2*	Microsomal glutathione S-transferase 2
ko00980	Metabolism of xenobiotics by cytochrome P450	Q9CPU4	*Mgst3*	Microsomal glutathione S-transferase 3
ko00980	Metabolism of xenobiotics by cytochrome P450	P24472	*Gsta4*	Glutathione S-transferase A4
ko05130	Pathogenic Escherichia coli infection	P08537	*tuba*	Tubulin alpha chain
ko05130	Pathogenic Escherichia coli infection	P11833		Tubulin beta chain
ko05130	Pathogenic Escherichia coli infection	Q9YHC3		Tubulin beta-1 chain
ko05130	Pathogenic Escherichia coli infection	Q91060		Tubulin alpha chain
ko05130	Pathogenic Escherichia coli infection	Q1JP79	*ARPC1A*	Actin-related protein 2/3 complex subunit 1A

ko05130	Pathogenic Escherichia coli infection	Q9UVX4	*ACT1*	Actin
ko05130	Pathogenic Escherichia coli infection	O17320		Actin
ko05130	Pathogenic Escherichia coli infection	Q25378	*PKC1*	Protein kinase C
ko05130	Pathogenic Escherichia coli infection	P18258		Tubulin alpha-1 chain
ko05130	Pathogenic Escherichia coli infection	Q00215		Actin, cytoplasmic
ko05130	Pathogenic Escherichia coli infection	Q4R5B3	*TUBB2A*	Tubulin beta-2A chain
ko05130	Pathogenic Escherichia coli infection	P41383	*TUB2*	Tubulin alpha-2/alpha-4 chain
ko05130	Pathogenic Escherichia coli infection	P52273		Tubulin alpha chain
ko05130	Pathogenic Escherichia coli infection	P12716		Actin, cytoplasmic
ko05130	Pathogenic Escherichia coli infection	P30883	*tubb4*	Tubulin beta-4 chain
ko05130	Pathogenic Escherichia coli infection	P61858	*betaTub85D*	Tubulin beta-2 chain
ko05130	Pathogenic Escherichia coli infection	P68373	*Tuba1c*	Tubulin alpha-1C chain
ko05130	Pathogenic Escherichia coli infection	P32882		Tubulin beta-2 chain
ko05130	Pathogenic Escherichia coli infection	P17126		Actin, non-muscle 6.2
ko05130	Pathogenic Escherichia coli infection	Q3MHR7	*ARPC2*	Actin-related protein 2/3 complex subunit 2
ko04540	Gap junction	Q07890	*SOS2*	Son of sevenless homolog 2
ko04540	Gap junction	P08537	*tuba*	Tubulin alpha chain
ko04540	Gap junction	P11833		Tubulin beta chain
ko04540	Gap junction	Q9YHC3		Tubulin beta-1 chain
ko04540	Gap junction	Q91060		Tubulin alpha chain
ko04540	Gap junction	Q25378	*PKC1*	Protein kinase C
ko04540	Gap junction	P18258		Tubulin alpha-1 chain
ko04540	Gap junction	Q4R5B3	*TUBB2A*	Tubulin beta-2A chain
ko04540	Gap junction	P41383	*TUB2*	Tubulin alpha-2/alpha-4 chain
ko04540	Gap junction	Q9Y2U5	*MAP3K2*	Mitogen-activated protein kinase kinase kinase 2
ko04540	Gap junction	P52273		Tubulin alpha chain

ko04540	Gap junction	P30883	*tubb4*	Tubulin beta-4 chain
ko04540	Gap junction	P61858	*betaTub85D*	Tubulin beta-2 chain
ko04540	Gap junction	P68373	*Tuba1c*	Tubulin alpha-1C chain
ko04540	Gap junction	P32882		Tubulin beta-2 chain
ko00630	Glyoxylate and dicarboxylate metabolism	Q2TBR0	*PCCB*	Propionyl-CoA carboxylase beta chain, mitochondrial
ko00630	Glyoxylate and dicarboxylate metabolism	P07511	*SHMT1*	Serine hydroxymethyltransferase, cytosolic
ko00630	Glyoxylate and dicarboxylate metabolism	Q9QXG4	*Acss2*	Acetyl-coenzyme A synthetase, cytoplasmic
ko00630	Glyoxylate and dicarboxylate metabolism	P04040	*CAT*	Catalase
ko00630	Glyoxylate and dicarboxylate metabolism	Q96PE7	*MCEE*	Methylmalonyl-CoA epimerase, mitochondrial
ko00630	Glyoxylate and dicarboxylate metabolism	Q9UJM8	*HAO1*	Hydroxyacid oxidase 1
ko00983	Drug metabolism - other enzymes	Q4KLN6	*Rrm2*	Ribonucleoside-diphosphate reductase subunit M2
ko00983	Drug metabolism - other enzymes	O77649	*UGT2B20*	UDP-glucuronosyltransferase 2B20
ko00983	Drug metabolism - other enzymes	P07742	*Rrm1*	Ribonucleoside-diphosphate reductase large subunit
ko00983	Drug metabolism - other enzymes	Q61133	*Gstt2*	Glutathione S-transferase theta-2
ko00983	Drug metabolism - other enzymes	Q2KJG4	*MGST2*	Microsomal glutathione S-transferase 2
ko00983	Drug metabolism - other enzymes	Q14117	*DPYS*	Dihydropyrimidinase
ko00983	Drug metabolism - other enzymes	Q9CPU4	*Mgst3*	Microsomal glutathione S-transferase 3
ko00983	Drug metabolism - other enzymes	P24472	*Gsta4*	Glutathione S-transferase A4
ko00520	Amino sugar and nucleotide sugar metabolism	Q91YN5	*Uap1*	UDP-N-acetylhexosamine pyrophosphorylase

ko00520	Amino sugar and nucleotide sugar metabolism	Q5R6R5	*CMAS*	N-acylneuraminate cytidylyltransferase
ko00520	Amino sugar and nucleotide sugar metabolism	Q4R591	*GPI*	Glucose-6-phosphate isomerase
ko00520	Amino sugar and nucleotide sugar metabolism	P07686	*HEXB*	Beta-hexosaminidase subunit beta
ko00520	Amino sugar and nucleotide sugar metabolism	Q6GMK8	*gmppaa*	Mannose-1-phosphate guanyltransferase alpha-A
ko00520	Amino sugar and nucleotide sugar metabolism	Q28CZ9	*cyb5r4*	Cytochrome b5 reductase 4
ko00520	Amino sugar and nucleotide sugar metabolism	Q8BTZ7	*Gmppb*	Mannose-1-phosphate guanyltransferase beta
ko00520	Amino sugar and nucleotide sugar metabolism	Q8K3X2	*TSTA3*	GDP-L-fucose synthase
ko03030	DNA replication	Q9DEA3	*PCNA*	Proliferating cell nuclear antigen
ko03030	DNA replication	Q5FW17	*rpa1*	Replication protein A 70 kDa DNA-binding subunit
ko03030	DNA replication	Q6DIH3	*mcm2*	DNA replication licensing factor mcm2
ko03030	DNA replication	Q6PCI7	*mcm5-b*	DNA replication licensing factor mcm5-B
ko03030	DNA replication	P35251	*RFC1*	Replication factor C subunit 1
ko03030	DNA replication	Q43124		Proliferating cell nuclear antigen
ko03030	DNA replication	Q99J62	*Rfc4*	Replication factor C subunit 4
ko03030	DNA replication	Q2TBV1	*RFC3*	Replication factor C subunit 3
ko03030	DNA replication	P33992	*MCM5*	DNA replication licensing factor MCM5
ko03030	DNA replication	Q91876	*mcm7-a*	DNA replication licensing factor mcm7-A
ko00020	Citrate cycle (TCA cycle)	Q01205	*Dlst*	Dihydrolipoyllysine-residue succinyltransferase component of 2-oxoglutarate dehydrogenase complex, mitochondrial

ko00020	Citrate cycle (TCA cycle)	Q9YHT1	*SDHA*	Succinate dehydrogenase [ubiquinone] flavoprotein subunit, mitochondrial
ko00020	Citrate cycle (TCA cycle)	O97580	*SUCLA2*	Succinyl-CoA ligase [ADP-forming] subunit beta, mitochondrial (Fragment)
ko00020	Citrate cycle (TCA cycle)	Q9YGD2	*SUCLG1*	Succinyl-CoA ligase subunit alpha, mitochondrial (Fragment)
ko00020	Citrate cycle (TCA cycle)	P53590	*SUCLG2*	Succinyl-CoA ligase [GDP-forming] subunit beta, mitochondrial (Fragment)
ko00020	Citrate cycle (TCA cycle)	P53396	*ACLY*	ATP-citrate synthase
ko00020	Citrate cycle (TCA cycle)	A7MB35	*PDHA1*	Pyruvate dehydrogenase E1 component subunit alpha, somatic form, mitochondrial
ko01524	Platinum drug resistance	P52701	*MSH6*	DNA mismatch repair protein Msh6
ko01524	Platinum drug resistance	P43247	*Msh2*	DNA mismatch repair protein Msh2
ko01524	Platinum drug resistance	Q61133	*Gstt2*	Glutathione S-transferase theta-2
ko01524	Platinum drug resistance	O42131	*TOP2B*	DNA topoisomerase 2-beta
ko01524	Platinum drug resistance	Q2KJG4	*MGST2*	Microsomal glutathione S-transferase 2
ko01524	Platinum drug resistance	Q9CPU4	*Mgst3*	Microsomal glutathione S-transferase 3
ko01524	Platinum drug resistance	P24472	*Gsta4*	Glutathione S-transferase A4
ko01524	Platinum drug resistance	Q13315	*ATM*	Serine-protein kinase ATM
ko05204	Chemical carcinogenesis	O77649	*UGT2B20*	UDP-glucuronosyltransferase 2B20
ko05204	Chemical carcinogenesis	Q61133	*Gstt2*	Glutathione S-transferase theta-2
ko05204	Chemical carcinogenesis	Q2KJG4	*MGST2*	Microsomal glutathione S-transferase 2
ko05204	Chemical carcinogenesis	Q9CPU4	*Mgst3*	Microsomal glutathione S-transferase 3
ko05204	Chemical carcinogenesis	P24472	*Gsta4*	Glutathione S-transferase A4
ko05204	Chemical carcinogenesis	Q6YP21	*CCBL2*	Kynurenine--oxoglutarate transaminase 3

Table S10. Results of Pfam enrichment analysis of up-regulated differentially expressed genes.

Pfam ID	Gene Family	Swiss-port ID	Gene Name	Gene Description
PF03131	bZIP Maf	P05412	*JUN*	Transcription factor AP-1
PF03131	bZIP Maf	O02755	*CEBPB*	CCAAT/enhancer-binding protein beta
PF03131	bZIP Maf	P51145	*Fosl2*	Fos-related antigen 2
PF03131	bZIP Maf	Q60795	*Nfe2l2*	Nuclear factor erythroid 2-related factor 2
PF03131	bZIP Maf	Q8R0S1	*Atf7*	Cyclic AMP-dependent transcription factor ATF-7
PF03131	bZIP Maf	P79145	*CREM*	cAMP-responsive element modulator
PF03131	bZIP Maf	P27925	*CREB1*	Cyclic AMP-responsive element-binding protein 1
PF03131	bZIP Maf	Q8IUR6	*CREBRF*	CREB3 regulatory factor
PF03131	bZIP Maf	O35426	*Xbp1*	X-box-binding protein 1
PF07716	bZIP 2	P05412	*JUN*	Transcription factor AP-1
PF07716	bZIP 2	O02755	*CEBPB*	CCAAT/enhancer-binding protein beta
PF07716	bZIP 2	P51145	*Fosl2*	Fos-related antigen 2
PF07716	bZIP 2	Q60795	*Nfe2l2*	Nuclear factor erythroid 2-related factor 2
PF07716	bZIP 2	Q8R0S1	*Atf7*	Cyclic AMP-dependent transcription factor ATF-7
PF07716	bZIP 2	P79145	*CREM*	cAMP-responsive element modulator
PF07716	bZIP 2	P27925	*CREB1*	Cyclic AMP-responsive element-binding protein 1
PF07716	bZIP 2	P62501	*Tsc22d1*	TSC22 domain family protein 1
PF07716	bZIP 2	Q8IUR6	*CREBRF*	CREB3 regulatory factor
PF07716	bZIP 2	O35426	*Xbp1*	X-box-binding protein 1
PF07716	bZIP 2	Q8TEY5	*CREB3L4*	Cyclic AMP-responsive element-binding protein 3-like protein 4
PF07716	bZIP 2	P35749	*MYH11*	Myosin-11
PF00170	bZIP 1	P05412	*JUN*	Transcription factor AP-1
PF00170	bZIP 1	O02755	*CEBPB*	CCAAT/enhancer-binding protein beta
PF00170	bZIP 1	P51145	*Fosl2*	Fos-related antigen 2

PF00170	bZIP 1	Q60795	*Nfe2l2*	Nuclear factor erythroid 2-related factor 2
PF00170	bZIP 1	Q8R0S1	*Atf7*	Cyclic AMP-dependent transcription factor ATF-7
PF00170	bZIP 1	P79145	*CREM*	cAMP-responsive element modulator
PF00170	bZIP 1	P27925	*CREB1*	Cyclic AMP-responsive element-binding protein 1
PF00170	bZIP 1	P62501	*Tsc22d1*	TSC22 domain family protein 1
PF00170	bZIP 1	Q8IUR6	*CREBRF*	CREB3 regulatory factor
PF00170	bZIP 1	Q5UEM7	*Creb3l4*	Cyclic AMP-responsive element-binding protein 3-like protein 4
PF00170	bZIP 1	O35426	*Xbp1*	X-box-binding protein 1
PF00170	bZIP 1	Q8TEY5	*CREB3L4*	Cyclic AMP-responsive element-binding protein 3-like protein 4
PF05773	RWD	Q9JI90	*Rnf14*	E3 ubiquitin-protein ligase RNF14
PF05773	RWD	O60140	*SPBC18H10.09*	Uncharacterized protein C18H10.09
PF05773	RWD	Q9Z255	*Ube2a*	Ubiquitin-conjugating enzyme E2 A
PF05773	RWD	Q8NEZ2	*VPS37A*	Vacuolar protein sorting-associated protein 37A
PF05773	RWD	Q29503	*UBE2R2*	Ubiquitin-conjugating enzyme E2 R2
PF05773	RWD	Q9UBS8	*RNF14*	E3 ubiquitin-protein ligase RNF14
PF05773	RWD	Q7Z7E8	*UBE2Q1*	Ubiquitin-conjugating enzyme E2 Q1
PF05773	RWD	Q96B70	*LENG9*	Leukocyte receptor cluster member 9
PF05773	RWD	Q66KB0	*ube2z*	Ubiquitin-conjugating enzyme E2 Z
PF03357	Snf7	Q6NVL7	*chmp2b*	Charged multivesicular body protein 2b
PF03357	Snf7	Q7T339	*chmp5*	Charged multivesicular body protein 5
PF03357	Snf7	Q7ZW25	*chmp2a*	Charged multivesicular body protein 2a
PF03357	Snf7	Q8CGS4	*Chmp3*	Charged multivesicular body protein 3
PF03357	Snf7	Q9HD42	*CHMP1A*	Charged multivesicular body protein 1a
PF03357	Snf7	Q9D8B3	*Chmp4b*	Charged multivesicular body protein 4b
PF03357	Snf7	Q9D7S9	*Chmp5*	Charged multivesicular body protein 5
PF03357	Snf7	Q6DF27	*chmp1b*	Charged multivesicular body protein 1b

PF13639	zf-RING 2	Q9P3U4	*dbl4*	E3 ubiquitin-protein ligase dbl4
PF13639	zf-RING 2	A8MQ27	*NEURL1B*	E3 ubiquitin-protein ligase NEURL1B
PF13639	zf-RING 2	Q9JI90	*Rnf14*	E3 ubiquitin-protein ligase RNF14
PF13639	zf-RING 2	Q6T486	*rbrA*	Probable E3 ubiquitin-protein ligase rbrA
PF13639	zf-RING 2	Q6PFJ9	*arih1*	E3 ubiquitin-protein ligase arih1
PF13639	zf-RING 2	O76050	*NEURL1*	E3 ubiquitin-protein ligase NEURL1
PF13639	zf-RING 2	Q1LV17	*irf2bp1*	Interferon regulatory factor 2-binding protein 1
PF13639	zf-RING 2	Q96FA3	*PELI1*	E3 ubiquitin-protein ligase pellino homolog 1
PF13639	zf-RING 2	Q5TC82	*RC3H1*	Roquin-1
PF13639	zf-RING 2	E1BD59	*TRIM56*	E3 ubiquitin-protein ligase TRIM56
PF13639	zf-RING 2	A9JTG5	*rbck1*	RanBP-type and C3HC4-type zinc finger-containing protein 1
PF13639	zf-RING 2	Q7Z6E9	*RBBP6*	E3 ubiquitin-protein ligase RBBP6
PF13639	zf-RING 2	O70277	*Trim3*	Tripartite motif-containing protein 3
PF13639	zf-RING 2	Q9R1A8	*Rfwd2*	E3 ubiquitin-protein ligase RFWD2
PF13639	zf-RING 2	F7H9X2	*TRIM2*	Tripartite motif-containing protein 2
PF13639	zf-RING 2	Q5SDR3	*BMI1*	Polycomb complex protein BMI-1
PF13639	zf-RING 2	Q91YT2	*Rnf185*	E3 ubiquitin-protein ligase RNF185
PF13639	zf-RING 2	Q1L5Z9	*LONRF2*	LON peptidase N-terminal domain and RING finger protein 2
PF13639	zf-RING 2	Q96PX1	*RNF157*	RING finger protein 157
PF13639	zf-RING 2	Q8N448	*LNX2*	Ligand of Numb protein X 2
PF13639	zf-RING 2	Q923S6	*Neurl1*	E3 ubiquitin-protein ligase NEURL1
PF13639	zf-RING 2	Q640V2	*rmnd5a*	Protein RMD5 homolog A
PF13639	zf-RING 2	Q6ZPS6	*Ankib1*	Ankyrin repeat and IBR domain-containing protein 1
PF14634	zf-RING 5	Q9P3U4	*dbl4*	E3 ubiquitin-protein ligase dbl4
PF14634	zf-RING 5	A8MQ27	*NEURL1B*	E3 ubiquitin-protein ligase NEURL1B
PF14634	zf-RING 5	Q9JI90	*Rnf14*	E3 ubiquitin-protein ligase RNF14

PF14634	zf-RING 5	Q6T486	*rbrA*	Probable E3 ubiquitin-protein ligase rbrA
PF14634	zf-RING 5	O76050	*NEURL1*	E3 ubiquitin-protein ligase NEURL1
PF14634	zf-RING 5	Q96FA3	*PELI1*	E3 ubiquitin-protein ligase pellino homolog 1
PF14634	zf-RING 5	Q5TC82	*RC3H1*	Roquin-1
PF14634	zf-RING 5	E1BD59	*TRIM56*	E3 ubiquitin-protein ligase TRIM56
PF14634	zf-RING 5	A9JTG5	*rbck1*	RanBP-type and C3HC4-type zinc finger-containing protein 1
PF14634	zf-RING 5	Q7Z6E9	*RBBP6*	E3 ubiquitin-protein ligase RBBP6
PF14634	zf-RING 5	O70277	*Trim3*	Tripartite motif-containing protein 3
PF14634	zf-RING 5	Q9R1A8	*Rfwd2*	E3 ubiquitin-protein ligase RFWD2
PF14634	zf-RING 5	F7H9X2	*TRIM2*	Tripartite motif-containing protein 2
PF14634	zf-RING 5	Q5SDR3	*BMI1*	Polycomb complex protein BMI-1
PF14634	zf-RING 5	Q91YT2	*Rnf185*	E3 ubiquitin-protein ligase RNF185
PF14634	zf-RING 5	Q1L5Z9	*LONRF2*	LON peptidase N-terminal domain and RING finger protein 2
PF14634	zf-RING 5	Q96PX1	*RNF157*	RING finger protein 157
PF14634	zf-RING 5	Q8N448	*LNX2*	Ligand of Numb protein X 2
PF14634	zf-RING 5	Q640V2	*rmnd5a*	Protein RMD5 homolog A
PF14634	zf-RING 5	Q6ZPS6	*Ankib1*	Ankyrin repeat and IBR domain-containing protein 1
PF05221	AdoHcyase	P51893	*ahcy-a*	Adenosylhomocysteinase A
PF13180	PDZ 2	A4FUC9	*RHPN2*	Rhophilin-2
PF13180	PDZ 2	O00560	*SDCBP*	Syntenin-1
PF13180	PDZ 2	Q9C0J8	*WDR33*	pre-mRNA 3' end processing protein WDR33
PF13180	PDZ 2	Q8TB45	*DEPTOR*	DEP domain-containing mTOR-interacting protein
PF13180	PDZ 2	O62683	*TJP3*	Tight junction protein ZO-3
PF13180	PDZ 2	Q7Z6J2	*GRASP*	General receptor for phosphoinositides 1-associated scaffold protein
PF13180	PDZ 2	Q5T5U3	*ARHGAP21*	Rho GTPase-activating protein 21
PF13180	PDZ 2	Q9JLB2	*Mpp5*	MAGUK p55 subfamily member 5

PF13180	PDZ 2	O08992	*Sdcbp*	Syntenin-1
PF13180	PDZ 2	P55196	*MLLT4*	Afadin
PF13180	PDZ 2	Q12923	*PTPN13*	Tyrosine-protein phosphatase non-receptor type 13
PF13180	PDZ 2	Q8N448	*LNX2*	Ligand of Numb protein X 2
PF02183	HALZ	O02755	*CEBPB*	CCAAT/enhancer-binding protein beta
PF02183	HALZ	P62501	*Tsc22d1*	TSC22 domain family protein 1
PF02183	HALZ	Q61371	*Ift88*	Intraflagellar transport protein 88 homolog
PF02183	HALZ	P39922		Myosin heavy chain, clone 203 (Fragment)
PF02183	HALZ	P70478	*Apc*	Adenomatous polyposis coli protein
PF02183	HALZ	Q8TEY5	*CREB3L4*	Cyclic AMP-responsive element-binding protein 3-like protein 4
PF02183	HALZ	P35749	*MYH11*	Myosin-11
PF00531	Death	Q6F3J0	*NFKB1*	Nuclear factor NF-kappa-B p105 subunit
PF00531	Death	Q9UDY8	*MALT1*	Mucosa-associated lymphoid tissue lymphoma translocation protein 1
PF00531	Death	Q6R653	*Unc5cl*	UNC5C-like protein
PF00531	Death	Q9NWZ3	*IRAK4*	Interleukin-1 receptor-associated kinase 4
PF00531	Death	Q8R9T6	*sps1*	Probable serine/threonine-protein kinase Sps1
PF00531	Death	O14964	*HGS*	Hepatocyte growth factor-regulated tyrosine kinase substrate
PF00595	PDZ	A4FUC9	*RHPN2*	Rhophilin-2
PF00595	PDZ	O00560	*SDCBP*	Syntenin-1
PF00595	PDZ	Q9C0J8	*WDR33*	pre-mRNA 3' end processing protein WDR33
PF00595	PDZ	Q8TB45	*DEPTOR*	DEP domain-containing mTOR-interacting protein
PF00595	PDZ	O62683	*TJP3*	Tight junction protein ZO-3
PF00595	PDZ	O08992	*Sdcbp*	Syntenin-1
PF00595	PDZ	Q7Z6J2	*GRASP*	General receptor for phosphoinositides 1-associated scaffold protein
PF00595	PDZ	Q5T5U3	*ARHGAP21*	Rho GTPase-activating protein 21
PF00595	PDZ	Q9JLB2	*Mpp5*	MAGUK p55 subfamily member 5

PF00595	PDZ	P55196	*MLLT4*	Afadin
PF00595	PDZ	Q12923	*PTPN13*	Tyrosine-protein phosphatase non-receptor type 13
PF00595	PDZ	Q8N448	*LNX2*	Ligand of Numb protein X 2
PF14560	Ubiquitin 2	Q63429	*Ubc*	Polyubiquitin-C
PF14560	Ubiquitin 2	Q8MKD1	*UBB*	Polyubiquitin-B
PF14560	Ubiquitin 2	P0CG68	*UBC*	Polyubiquitin-C
PF14560	Ubiquitin 2	P0CG61	*UBC*	Polyubiquitin-C
PF14560	Ubiquitin 2	P0CG47	*UBB*	Polyubiquitin-B
PF14560	Ubiquitin 2	P0CG73	*UBI1*	Polyubiquitin
PF14560	Ubiquitin 2	P0CH28	*UBC*	Polyubiquitin-C
PF14560	Ubiquitin 2	Q8C5W3	*Tbcel*	Tubulin-specific chaperone cofactor E-like protein
PF04977	DivIC	P05412	*JUN*	Transcription factor AP-1
PF04977	DivIC	O02755	*CEBPB*	CCAAT/enhancer-binding protein beta
PF04977	DivIC	P62501	*Tsc22d1*	TSC22 domain family protein 1
PF04977	DivIC	Q61371	*Ift88*	Intraflagellar transport protein 88 homolog
PF04977	DivIC	Q5RAN1	*RABGAP1*	Rab GTPase-activating protein 1
PF04977	DivIC	O35426	*Xbp1*	X-box-binding protein 1
PF04977	DivIC	Q8TEY5	*CREB3L4*	Cyclic AMP-responsive element-binding protein 3-like protein 4
PF00060	Lig chan	P19439	*KBP*	Probable glutamate receptor
PF00060	Lig chan	Q03445	*GluRIA*	Glutamate receptor 1
PF00060	Lig chan	Q10914	*glr-2*	Glutamate receptor 2
PF00060	Lig chan	P23818	*Gria1*	Glutamate receptor 1
PF00060	Lig chan	P26591		Glutamate receptor
PF00240	ubiquitin	Q63429	*Ubc*	Polyubiquitin-C
PF00240	ubiquitin	Q8MKD1	*UBB*	Polyubiquitin-B
PF00240	ubiquitin	P0CG68	*UBC*	Polyubiquitin-C

PF00240	ubiquitin	P0CG61	*UBC*	Polyubiquitin-C
PF00240	ubiquitin	P0CG47	*UBB*	Polyubiquitin-B
PF00240	ubiquitin	A9JTG5	*rbck1*	RanBP-type and C3HC4-type zinc finger-containing protein 1
PF00240	ubiquitin	P0CG73	*UBI1*	Polyubiquitin
PF00240	ubiquitin	P0CH28	*UBC*	Polyubiquitin-C
PF00240	ubiquitin	Q28DF1	*herpud2*	Homocysteine-responsive endoplasmic reticulum-resident ubiquitin-like domain member 2 protein
PF00240	ubiquitin	Q8R317	*Ubqln1*	Ubiquilin-1
PF10613	Lig chan-Glu bd	Q03445	*GluRIA*	Glutamate receptor 1
PF10613	Lig chan-Glu bd	P23818	*Gria1*	Glutamate receptor 1
PF06009	Laminin II	Q7T339	*chmp5*	Charged multivesicular body protein 5
PF06009	Laminin II	Q9D8B3	*Chmp4b*	Charged multivesicular body protein 4b
PF06009	Laminin II	P39922		Myosin heavy chain, clone 203 (Fragment)
PF06009	Laminin II	Q5RBW6	*STX12*	Syntaxin-12

Table S11. Results of Pfam enrichment analysis of down-regulated differentially expressed genes.

Pfam ID	Gene Family	Swiss-port ID	Gene Name	Gene Description
PF00076	RRM 1	Q6NWC6	*cpsf6*	Cleavage and polyadenylation specificity factor subunit 6
PF00076	RRM 1	Q8VIJ6	*Sfpq*	Splicing factor, proline- and glutamine-rich
PF00076	RRM 1	Q6IQ97	*rbm4.1*	RNA-binding protein 4.1
PF00076	RRM 1	Q8BL97	*Srsf7*	Serine/arginine-rich splicing factor 7
PF00076	RRM 1	Q6DID3	*Scaf8*	Protein SCAF8
PF00076	RRM 1	P51968		Heterogeneous nuclear ribonucleoprotein A3 homolog 1
PF00076	RRM 1	Q8C854	*Myef2*	Myelin expression factor 2
PF00076	RRM 1	P33240	*CSTF2*	Cleavage stimulation factor subunit 2
PF00076	RRM 1	Q2T9I5	*rbm26*	RNA-binding protein 26
PF00076	RRM 1	Q8VE97	*Srsf4*	Serine/arginine-rich splicing factor 4
PF00076	RRM 1	Q9UKM9	*RALY*	RNA-binding protein Raly
PF00076	RRM 1	Q98SJ2	*dazap1*	DAZ-associated protein 1
PF00076	RRM 1	Q7TMK9	*Syncrip*	Heterogeneous nuclear ribonucleoprotein Q
PF00076	RRM 1	Q8BH74	*Nup107*	Nuclear pore complex protein Nup107
PF00076	RRM 1	Q9JII5	*Dazap1*	DAZ-associated protein 1
PF00076	RRM 1	Q8VYA5	*RS2Z33*	Serine/arginine-rich splicing factor RS2Z33
PF00076	RRM 1	Q32P51	*HNRNPA1L2*	Heterogeneous nuclear ribonucleoprotein A1-like 2
PF00076	RRM 1	P31483	*TIA1*	Nucleolysin TIA-1 isoform p40
PF00076	RRM 1	Q5RBR8	*EIF4H*	Eukaryotic translation initiation factor 4H
PF00076	RRM 1	P09867	*HNRNPA1*	Heterogeneous nuclear ribonucleoprotein A1
PF00076	RRM 1	Q5ZLP8	*IGF2BP3*	Insulin-like growth factor 2 mRNA-binding protein 3
PF00076	RRM 1	P23588	*EIF4B*	Eukaryotic translation initiation factor 4B
PF00076	RRM 1	Q7ZVR8	*esrp2*	Epithelial splicing regulatory protein 2
PF00076	RRM 1	Q08473	*sqd*	RNA-binding protein squid

PF00076	RRM 1	Q8BG05	*Hnrnpa3*	Heterogeneous nuclear ribonucleoprotein A3
PF00076	RRM 1	Q99729	*HNRNPAB*	Heterogeneous nuclear ribonucleoprotein A/B
PF00076	RRM 1	Q3T106	*SRSF7*	Serine/arginine-rich splicing factor 7
PF00076	RRM 1	Q27294	*caz*	RNA-binding protein cabeza
PF00076	RRM 1	P31943	*HNRNPH1*	Heterogeneous nuclear ribonucleoprotein H
PF00076	RRM 1	Q8R081	*Hnrnpl*	Heterogeneous nuclear ribonucleoprotein L
PF00076	RRM 1	P70333	*Hnrnph2*	Heterogeneous nuclear ribonucleoprotein H2
PF00076	RRM 1	O43390	*HNRNPR*	Heterogeneous nuclear ribonucleoprotein R
PF00076	RRM 1	Q6NU14	*hnrnpdl-b*	Heterogeneous nuclear ribonucleoprotein D-like-B
PF00076	RRM 1	O60812	*HNRNPCL1*	Heterogeneous nuclear ribonucleoprotein C-like 1
PF00076	RRM 1	Q6PHZ5	*Rbm15b*	Putative RNA-binding protein 15B
PF00076	RRM 1	Q86U42	*PABPN1*	Polyadenylate-binding protein 2
PF00076	RRM 1	Q6PH90	*rbm8a-b*	RNA-binding protein 8A-B
PF00076	RRM 1	Q29RT0	*RBMX*	RNA-binding motif protein, X chromosome
PF00076	RRM 1	O76536		Hyalin (Fragment)
PF00076	RRM 1	P59708	*Sf3b6*	Splicing factor 3B subunit 6
PF02798	GST N	P0CG30	*GSTT2B*	Glutathione S-transferase theta-2B
PF02798	GST N	P57113	*Gstz1*	Maleylacetoacetate isomerase
PF02798	GST N	Q61133	*Gstt2*	Glutathione S-transferase theta-2
PF02798	GST N	O60760	*HPGDS*	Hematopoietic prostaglandin D synthase
PF02798	GST N	P24472	*Gsta4*	Glutathione S-transferase A4
PF02798	GST N	Q6PF21	*mars*	Methionine--tRNA ligase, cytoplasmic
PF16367	RRM 7	Q8VIJ6	*Sfpq*	Splicing factor, proline- and glutamine-rich
PF16367	RRM 7	Q6IQ97	*rbm4.1*	RNA-binding protein 4.1
PF16367	RRM 7	Q8BL97	*Srsf7*	Serine/arginine-rich splicing factor 7
PF16367	RRM 7	P51968		Heterogeneous nuclear ribonucleoprotein A3 homolog 1

PF16367	RRM 7	P33240	*CSTF2*	Cleavage stimulation factor subunit 2
PF16367	RRM 7	Q98SJ2	*dazap1*	DAZ-associated protein 1
PF16367	RRM 7	Q9JII5	*Dazap1*	DAZ-associated protein 1
PF16367	RRM 7	Q32P51	*HNRNPA1L2*	Heterogeneous nuclear ribonucleoprotein A1-like 2
PF16367	RRM 7	P31483	*TIA1*	Nucleolysin TIA-1 isoform p40
PF16367	RRM 7	P09867	*HNRNPA1*	Heterogeneous nuclear ribonucleoprotein A1
PF16367	RRM 7	Q08473	*sqd*	RNA-binding protein squid
PF16367	RRM 7	Q8BG05	*Hnrnpa3*	Heterogeneous nuclear ribonucleoprotein A3
PF16367	RRM 7	Q99729	*HNRNPAB*	Heterogeneous nuclear ribonucleoprotein A/B
PF16367	RRM 7	Q3T106	*SRSF7*	Serine/arginine-rich splicing factor 7
PF16367	RRM 7	Q7TMK9	*Syncrip*	Heterogeneous nuclear ribonucleoprotein Q
PF16367	RRM 7	Q6NU14	*hnrnpdl-b*	Heterogeneous nuclear ribonucleoprotein D-like-B
PF16367	RRM 7	Q6PHZ5	*Rbm15b*	Putative RNA-binding protein 15B
PF16367	RRM 7	Q29RT0	*RBMX*	RNA-binding motif protein, X chromosome
PF16367	RRM 7	O76536		Hyalin (Fragment)
PF00462	Glutaredoxin	P57113	*Gstz1*	Maleylacetoacetate isomerase
PF00462	Glutaredoxin	Q99MD6	*Txnrd3*	Thioredoxin reductase 3
PF00462	Glutaredoxin	Q5RC61	*SH3BGRL3*	SH3 domain-binding glutamic acid-rich-like protein 3
PF00462	Glutaredoxin	Q92J02	*grxC1*	Glutaredoxin-1
PF00462	Glutaredoxin	Q9H299	*SH3BGRL3*	SH3 domain-binding glutamic acid-rich-like protein 3
PF00462	Glutaredoxin	Q29RV1	*PDIA4*	Protein disulfide-isomerase A4
PF00462	Glutaredoxin	Q8TCB7	*METTL6*	Methyltransferase-like protein 6
PF00462	Glutaredoxin	Q6PBM1	*glrx5*	Glutaredoxin-related protein 5, mitochondrial
PF00462	Glutaredoxin	Q91W90	*Txndc5*	Thioredoxin domain-containing protein 5
PF00462	Glutaredoxin	Q8R4U2	*P4HB*	Protein disulfide-isomerase
PF00462	Glutaredoxin	P55822	*SH3BGR*	SH3 domain-binding glutamic acid-rich protein

PF08777	RRM 3	Q8VIJ6	*Sfpq*	Splicing factor, proline- and glutamine-rich
PF08777	RRM 3	Q8C854	*Myef2*	Myelin expression factor 2
PF08777	RRM 3	Q8VE97	*Srsf4*	Serine/arginine-rich splicing factor 4
PF08777	RRM 3	Q98SJ2	*dazap1*	DAZ-associated protein 1
PF08777	RRM 3	Q7TMK9	*Syncrip*	Heterogeneous nuclear ribonucleoprotein Q
PF08777	RRM 3	Q9JII5	*Dazap1*	DAZ-associated protein 1
PF08777	RRM 3	Q32P51	*HNRNPA1L2*	Heterogeneous nuclear ribonucleoprotein A1-like 2
PF08777	RRM 3	P09867	*HNRNPA1*	Heterogeneous nuclear ribonucleoprotein A1
PF08777	RRM 3	Q8BG05	*Hnrnpa3*	Heterogeneous nuclear ribonucleoprotein A3
PF08777	RRM 3	Q99729	*HNRNPAB*	Heterogeneous nuclear ribonucleoprotein A/B
PF08777	RRM 3	P31943	*HNRNPH1*	Heterogeneous nuclear ribonucleoprotein H
PF08777	RRM 3	Q8R081	*Hnrnpl*	Heterogeneous nuclear ribonucleoprotein L
PF08777	RRM 3	P70333	*Hnrnph2*	Heterogeneous nuclear ribonucleoprotein H2
PF08777	RRM 3	O43390	*HNRNPR*	Heterogeneous nuclear ribonucleoprotein R
PF08777	RRM 3	P51968		Heterogeneous nuclear ribonucleoprotein A3 homolog 1
PF08777	RRM 3	Q6NU14	*hnrnpdl-b*	Heterogeneous nuclear ribonucleoprotein D-like-B
PF08777	RRM 3	Q6PHZ5	*Rbm15b*	Putative RNA-binding protein 15B
PF08777	RRM 3	Q6PH90	*rbm8a-b*	RNA-binding protein 8A-B
PF08777	RRM 3	O76536		Hyalin (Fragment)
PF00091	Tubulin	P08537	*tuba*	Tubulin alpha chain
PF00091	Tubulin	P11833		Tubulin beta chain
PF00091	Tubulin	Q91060		Tubulin alpha chain
PF00091	Tubulin	P18258		Tubulin alpha-1 chain
PF00091	Tubulin	P41383	*TUB2*	Tubulin alpha-2/alpha-4 chain
PF00091	Tubulin	P68371	*TUBB4B*	Tubulin beta-4B chain
PF00091	Tubulin	P30883	*tubb4*	Tubulin beta-4 chain

PF00091	Tubulin	P61858	*betaTub85D*	Tubulin beta-2 chain
PF00013	KH 1	P61979	*Hnrnpk*	Heterogeneous nuclear ribonucleoprotein K
PF00013	KH 1	Q5VWX1	*KHDRBS2*	KH domain-containing, RNA-binding, signal transduction-associated protein 2
PF00013	KH 1	Q5ZLP8	*IGF2BP3*	Insulin-like growth factor 2 mRNA-binding protein 3
PF00013	KH 1	Q32PX7	*Fubp1*	Far upstream element-binding protein 1
PF00013	KH 1	P61978	*HNRNPK*	Heterogeneous nuclear ribonucleoprotein K
PF00013	KH 1	O19049	*HNRNPK*	Heterogeneous nuclear ribonucleoprotein K
PF00013	KH 1	Q80VL1	*Tdrkh*	Tudor and KH domain-containing protein
PF07650	KH 2	P61979	*Hnrnpk*	Heterogeneous nuclear ribonucleoprotein K
PF07650	KH 2	Q5ZLP8	*IGF2BP3*	Insulin-like growth factor 2 mRNA-binding protein 3
PF07650	KH 2	Q32PX7	*Fubp1*	Far upstream element-binding protein 1
PF07650	KH 2	P61978	*HNRNPK*	Heterogeneous nuclear ribonucleoprotein K
PF07650	KH 2	O19049	*HNRNPK*	Heterogeneous nuclear ribonucleoprotein K
PF07650	KH 2	Q80VL1	*Tdrkh*	Tudor and KH domain-containing protein
PF00578	AhpC-TSA	Q8R4U2	*P4HB*	Protein disulfide-isomerase
PF00578	AhpC-TSA	P99029	*Prdx5*	Peroxiredoxin-5, mitochondrial
PF00578	AhpC-TSA	Q29RV1	*PDIA4*	Protein disulfide-isomerase A4
PF00578	AhpC-TSA	O08807	*Prdx4*	Peroxiredoxin-4
PF00578	AhpC-TSA	Q5ZJF4	*PRDX6*	Peroxiredoxin-6
PF00578	AhpC-TSA	P38659	*Pdia4*	Protein disulfide-isomerase A4
PF00578	AhpC-TSA	Q91W90	*Txndc5*	Thioredoxin domain-containing protein 5
PF00578	AhpC-TSA	Q503L9	*nxn*	Nucleoredoxin
PF00578	AhpC-TSA	Q17770	*pdi-2*	Protein disulfide-isomerase 2
PF00578	AhpC-TSA	Q8JG64	*PDIA3*	Protein disulfide-isomerase A3
PF08534	Redoxin	P99029	*Prdx5*	Peroxiredoxin-5, mitochondrial
PF08534	Redoxin	Q29RV1	*PDIA4*	Protein disulfide-isomerase A4

PF08534	Redoxin	O08807	*Prdx4*	Peroxiredoxin-4
PF08534	Redoxin	Q5ZJF4	*PRDX6*	Peroxiredoxin-6
PF08534	Redoxin	P38659	*Pdia4*	Protein disulfide-isomerase A4
PF08534	Redoxin	Q91W90	*Txndc5*	Thioredoxin domain-containing protein 5
PF08534	Redoxin	Q8R4U2	*P4HB*	Protein disulfide-isomerase
PF08534	Redoxin	Q503L9	*nxn*	Nucleoredoxin
PF08534	Redoxin	Q8JG64	*PDIA3*	Protein disulfide-isomerase A3
PF00085	Thioredoxin	P83876	*TXNL4A*	Thioredoxin-like protein 4A
PF00085	Thioredoxin	O18883	*TXNDC9*	Thioredoxin domain-containing protein 9
PF00085	Thioredoxin	Q8R4U2	*P4HB*	Protein disulfide-isomerase
PF00085	Thioredoxin	Q29RV1	*PDIA4*	Protein disulfide-isomerase A4
PF00085	Thioredoxin	P38659	*Pdia4*	Protein disulfide-isomerase A4
PF00085	Thioredoxin	Q13454	*TUSC3*	Tumor suppressor candidate 3
PF00085	Thioredoxin	Q91W90	*Txndc5*	Thioredoxin domain-containing protein 5
PF00085	Thioredoxin	Q9CQM5	*Txndc17*	Thioredoxin domain-containing protein 17
PF00085	Thioredoxin	Q503L9	*nxn*	Nucleoredoxin
PF00085	Thioredoxin	Q17770	*pdi-2*	Protein disulfide-isomerase 2
PF00085	Thioredoxin	Q8JG64	*PDIA3*	Protein disulfide-isomerase A3
PF00085	Thioredoxin	Q8K2Q9	*Kiaa1598*	Shootin-1
PF05175	MTS	P27641	*Xrcc5*	X-ray repair cross-complementing protein 5
PF05175	MTS	Q8N6F8	*WBSCR27*	Williams-Beuren syndrome chromosomal region 27 protein
PF05175	MTS	Q86X55	*CARM1*	Histone-arginine methyltransferase CARM1
PF05175	MTS	O24144	*CCOAOMT1*	Caffeoyl-CoA O-methyltransferase 1
PF05175	MTS	A0AK43	*menG*	Demethylmenaquinone methyltransferase
PF05175	MTS	Q6AY46	*Trmt61a*	tRNA (adenine(58)-N(1))-methyltransferase catalytic subunit TRMT61A
PF05175	MTS	Q8TCB7	*METTL6*	Methyltransferase-like protein 6

PF05175	MTS	O14744	*PRMT5*	Protein arginine N-methyltransferase 5
PF05175	MTS	Q55EX9	*DDB_G0268948*	Putative methyltransferase DDB_G0268948
PF13184	KH 5	P61979	*Hnrnpk*	Heterogeneous nuclear ribonucleoprotein K
PF13184	KH 5	Q5ZLP8	*IGF2BP3*	Insulin-like growth factor 2 mRNA-binding protein 3
PF13184	KH 5	Q32PX7	*Fubp1*	Far upstream element-binding protein 1
PF13184	KH 5	P61978	*HNRNPK*	Heterogeneous nuclear ribonucleoprotein K
PF13184	KH 5	O19049	*HNRNPK*	Heterogeneous nuclear ribonucleoprotein K
PF13184	KH 5	Q80VL1	*Tdrkh*	Tudor and KH domain-containing protein
PF08241	Methyltransf 11	A6H791	*TRMT61A*	tRNA (adenine(58)-N(1))-methyltransferase catalytic subunit TRMT61A
PF08241	Methyltransf 11	Q8N6F8	*WBSCR27*	Williams-Beuren syndrome chromosomal region 27 protein
PF08241	Methyltransf 11	Q54ED7	*pks40*	Probable polyketide synthase 40
PF08241	Methyltransf 11	O24144	*CCOAOMT1*	Caffeoyl-CoA O-methyltransferase 1
PF08241	Methyltransf 11	A0AK43	*menG*	Demethylmenaquinone methyltransferase
PF08241	Methyltransf 11	Q0V9P1	*hnmt*	Histamine N-methyltransferase
PF08241	Methyltransf 11	Q8TCB7	*METTL6*	Methyltransferase-like protein 6
PF08241	Methyltransf 11	Q55E72	*stlA*	Probable polyketide synthase 1
PF08241	Methyltransf 11	Q55EX9	*DDB_G0268948*	Putative methyltransferase DDB_G0268948
PF08241	Methyltransf 11	Q9CY21	*Wbscr22*	Probable 18S rRNA (guanine-N(7))-methyltransferase
PF08241	Methyltransf 11	Q6DC37	*hnmt*	Histamine N-methyltransferase
PF08241	Methyltransf 11	Q9D0L8	*Rnmt*	mRNA cap guanine-N7 methyltransferase
PF13417	GST N 3	Q4R3I0	*MTX1*	Metaxin-1
PF13417	GST N 3	P0CG30	*GSTT2B*	Glutathione S-transferase theta-2B
PF13417	GST N 3	P57113	*Gstz1*	Maleylacetoacetate isomerase
PF13417	GST N 3	Q99MD6	*Txnrd3*	Thioredoxin reductase 3
PF13417	GST N 3	Q92J02	*grxC1*	Glutaredoxin-1
PF13417	GST N 3	P42620	*yqjG*	Glutathionyl-hydroquinone reductase YqjG

PF13417	GST N 3	Q61133	*Gstt2*	Glutathione S-transferase theta-2
PF13417	GST N 3	O60760	*HPGDS*	Hematopoietic prostaglandin D synthase

Table S12. Swiss-port annotation of genes in profile A.

Swiss-prot ID	Gene Name	Gene Description
Q8K2Y7	*Mrpl47*	39S ribosomal protein L47, mitochondrial
Q9H0Y0	*ATG10*	Ubiquitin-like-conjugating enzyme ATG10
Q16QL3	*coq2*	4-hydroxybenzoate polyprenyltransferase, mitochondrial
Q9NSN8	*SNTG1*	Gamma-1-syntrophin
Q682D3	*XRCC2*	DNA repair protein XRCC2 homolog
P35738	*Bckdhb*	2-oxoisovalerate dehydrogenase subunit beta, mitochondrial
Q6ZUK4	*TMEM26*	Transmembrane protein 26
Q2KJA6	*INTS9*	Integrator complex subunit 9
Q15020	*SART3*	Squamous cell carcinoma antigen recognized by T-cells 3
Q5RAS0	*ACADS*	Short-chain specific acyl-CoA dehydrogenase, mitochondrial
Q0V9J0	*tmem69*	Transmembrane protein 69
Q4VCS5	*AMOT*	Angiomotin
Q32KR6	*FDFT1*	Squalene synthase
Q3V188	*Endou*	Poly(U)-specific endoribonuclease
Q925Q5	*Gpha2*	Glycoprotein hormone alpha-2
Q6P829	*nosip*	Nitric oxide synthase-interacting protein
Q9JIY6	*Cml6*	Probable N-acetyltransferase CML6
Q6IQU7	*ints12*	Integrator complex subunit 12
Q6NXM2	*Rcbtb1*	RCC1 and BTB domain-containing protein 1
Q13825	*AUH*	Methylglutaconyl-CoA hydratase, mitochondrial
C9JN71	*ZNF878*	Zinc finger protein 878
Q8CC88	*Vwa8*	von Willebrand factor A domain-containing protein 8
Q9V4A7	*plexB*	Plexin-B
P23588	*EIF4B*	Eukaryotic translation initiation factor 4B

Q5R5N4	*METTL14*	N6-adenosine-methyltransferase subunit METTL14
Q9R0D8	*Wdr54*	WD repeat-containing protein 54
O75486	*SUPT3H*	Transcription initiation protein SPT3 homolog
A4FV72	*PPIE*	Peptidyl-prolyl cis-trans isomerase E
Q9UNP9	*PPIE*	Peptidyl-prolyl cis-trans isomerase E
Q6ZMW2	*ZNF782*	Zinc finger protein 782
P21675	*TAF1*	Transcription initiation factor TFIID subunit 1
Q9CPV5	*Pmf1*	Polyamine-modulated factor 1
Q2KJJ0	*PRPF6*	Pre-mRNA-processing factor 6
Q924L9	*FPGS*	Folylpolyglutamate synthase, mitochondrial
Q5RD73	*DUSP3*	Dual specificity protein phosphatase 3
Q5F408	*CARS*	Cysteine--tRNA ligase, cytoplasmic
Q9ER72	*Cars*	Cysteine--tRNA ligase, cytoplasmic
Q2TBI1	*NSMCE4A*	Non-structural maintenance of chromosomes element 4 homolog A
O08654		UPF0183 protein C16orf70 homolog
Q9ERE7	*Mesdc2*	LDLR chaperone MESD
P34439	*F54C8.1*	Probable 3-hydroxyacyl-CoA dehydrogenase F54C8.1
Q5ZI51	*ADPRHL2*	Poly(ADP-ribose) glycohydrolase ARH3
Q80VC6	*Trnau1ap*	tRNA selenocysteine 1-associated protein 1
Q9H1A4	*ANAPC1*	Anaphase-promoting complex subunit 1
Q9UBI9	*HECA*	Headcase protein homolog
Q6GPP0	*wdr70*	WD repeat-containing protein 70
Q0VD42	*TMEM136*	Transmembrane protein 136
Q9Y6A2	*CYP46A1*	Cholesterol 24-hydroxylase
Q9WVK8	*Cyp46a1*	Cholesterol 24-hydroxylase
Q8BUB6	*Ust*	Uronyl 2-sulfotransferase

O35386	*Phyh*	Phytanoyl-CoA dioxygenase, peroxisomal
Q9GZQ3	*COMMD5*	COMM domain-containing protein 5
Q9WU83	*DPM1*	Dolichol-phosphate mannosyltransferase subunit 1
P34205	*phr*	Deoxyribodipyrimidine photo-lyase
Q28811	*PHR*	Deoxyribodipyrimidine photo-lyase
Q8BKT7	*Thoc5*	THO complex subunit 5 homolog
Q7Z7A1	*CNTRL*	Centriolin
Q8WWV3	*RTN4IP1*	Reticulon-4-interacting protein 1, mitochondrial
P42669	*Pura*	Transcriptional activator protein Pur-alpha
Q8CAK3		UPF0515 protein C19orf66 homolog
Q90687	*PTPN11*	Tyrosine-protein phosphatase non-receptor type 11
Q02293	*Fntb*	Protein farnesyltransferase subunit beta
Q9EP69	*Sacm1l*	Phosphatidylinositide phosphatase SAC1
P34447	*F54F2.2/F54F2.3/F54F2.4*	Uncharacterized protein F54F2.2, isoform a
P35969	*Flt1*	Vascular endothelial growth factor receptor 1
O14522	*PTPRT*	Receptor-type tyrosine-protein phosphatase T
A2VDF0	*FUOM*	Fucose mutarotase
Q5RF24	*WDR13*	WD repeat-containing protein 13
P07872	*Acox1*	Peroxisomal acyl-coenzyme A oxidase 1
P27652		Renilla-luciferin 2-monooxygenase
Q90330	*FGFR4*	Fibroblast growth factor receptor 4
Q9WUE3	*Cyb561d2*	Cytochrome b561 domain-containing protein 2
Q2KHV4	*PARL*	Presenilins-associated rhomboid-like protein, mitochondrial
A7RM45	*v1g160387*	Probable lysosomal cobalamin transporter
O18883	*TXNDC9*	Thioredoxin domain-containing protein 9
Q8BK67	*Rcc2*	Protein RCC2

Q9H6U8	*ALG9*	Alpha-1,2-mannosyltransferase ALG9
Q6GMH0	*prpf18*	Pre-mRNA-splicing factor 18
Q4V7C1	*Fgfr1op*	FGFR1 oncogene partner
Q86TV6	*TTC7B*	Tetratricopeptide repeat protein 7B
P43234	*CTSO*	Cathepsin O
Q568N4	*timm10b*	Mitochondrial import inner membrane translocase subunit Tim10 B
Q0P5A2	*COQ5*	2-methoxy-6-polyprenyl-1,4-benzoquinol methylase, mitochondrial
Q5PQ53	*pef1*	Peflin
Q05B83	*RFC2*	Replication factor C subunit 2
Q9JKF7	*Mrpl39*	39S ribosomal protein L39, mitochondrial
O94906	*PRPF6*	Pre-mRNA-processing factor 6
P51797	*CLCN6*	Chloride transport protein 6
Q8BGV0	*Nars2*	Probable asparagine--tRNA ligase, mitochondrial
O75808	*CAPN15*	Calpain-15
Q8WND5	*IKBKAP*	Elongator complex protein 1
P70371	*Terf1*	Telomeric repeat-binding factor 1
Q924T2	*Mrps2*	28S ribosomal protein S2, mitochondrial
Q3SZI6	*RPN2*	Dolichyl-diphosphooligosaccharide--protein glycosyltransferase subunit 2
O18870	*SMN1*	Survival motor neuron protein
Q6NZ06	*ilf2*	Interleukin enhancer-binding factor 2 homolog
Q6PBF7	*emc4*	ER membrane protein complex subunit 4
Q8BGX3	*Lrtm2*	Leucine-rich repeat and transmembrane domain-containing protein 2
Q5U247	*exoc8*	Exocyst complex component 8
Q9UJ70	*NAGK*	N-acetyl-D-glucosamine kinase
B0BNM1	*Apoa1bp*	NAD(P)H-hydrate epimerase
P43247	*Msh2*	DNA mismatch repair protein Msh2

Q9C091	*GREB1L*	GREB1-like protein
Q6NYL3	*ehhadh*	Peroxisomal bifunctional enzyme
A4K526		Transmembrane protein 256 homolog
Q3SYW9	*CHTOP*	Chromatin target of PRMT1 protein
Q9VCA2	*Orct*	Organic cation transporter protein
Q66J54	*slc22a6-a*	Solute carrier family 22 member 6-A
Q8C008	*Dzank1*	Double zinc ribbon and ankyrin repeat-containing protein 1
Q9JMI1	*Aacs*	Acetoacetyl-CoA synthetase
Q0P5G1	*TONSL*	Tonsoku-like protein
Q8IVD9	*NUDCD3*	NudC domain-containing protein 3
P24472	*Gsta4*	Glutathione S-transferase A4
Q5BLE8	*retsat*	Putative all-trans-retinol 13,14-reductase
Q5E9L7	*VPS16*	Vacuolar protein sorting-associated protein 16 homolog
Q9NUL7	*DDX28*	Probable ATP-dependent RNA helicase DDX28
Q8N0W3	*FUK*	L-fucose kinase
Q9NVH2	*INTS7*	Integrator complex subunit 7
Q13439	*GOLGA4*	Golgin subfamily A member 4
P49257	*LMAN1*	Protein ERGIC-53
Q6GQI5	*ccdc93*	Coiled-coil domain-containing protein 93
Q5ZKI4	*CCDC93*	Coiled-coil domain-containing protein 93
Q6ZNB6	*NFXL1*	NF-X1-type zinc finger protein NFXL1
Q04589	*Fgfr1*	Fibroblast growth factor receptor 1
O16264	*F40A3.3*	Phosphatidylethanolamine-binding protein homolog F40A3.3
A1YER2	*HTATIP2*	Oxidoreductase HTATIP2
Q3U3N6	*Enthd2*	AP-4 complex accessory subunit tepsin
Q9D8Z1	*Ascc1*	Activating signal cointegrator 1 complex subunit 1

Q90941	*PBRM1*	Protein polybromo-1
P29054	*gtf2b*	Transcription initiation factor IIB
Q93373	*let-4*	Leucine-rich repeat-containing protein let-4
P57088	*TMEM33*	Transmembrane protein 33
P21953	*BCKDHB*	2-oxoisovalerate dehydrogenase subunit beta, mitochondrial
Q8CIG3	*Kdm1b*	Lysine-specific histone demethylase 1B
A2TLM1	*RPIA*	Ribose-5-phosphate isomerase
O75096	*LRP4*	Low-density lipoprotein receptor-related protein 4
Q15649	*ZNHIT3*	Zinc finger HIT domain-containing protein 3
A3KPL7	*tmem170a*	Transmembrane protein 170A
O88280	*Slit3*	Slit homolog 3 protein
Q8LGG0	*FKBP12*	Peptidyl-prolyl cis-trans isomerase FKBP12
O75365	*PTP4A3*	Protein tyrosine phosphatase type IVA 3
Q9NVM9	*ASUN*	Protein asunder homolog
O22190	*GH3.3*	Indole-3-acetic acid-amido synthetase GH3.3
P70541	*Eif2b3*	Translation initiation factor eIF-2B subunit gamma
A8KBL5	*slc38a11*	Putative sodium-coupled neutral amino acid transporter 11
A0JMI9	*gfm2*	Ribosome-releasing factor 2, mitochondrial
Q5ZHT1	*ACAD11*	Acyl-CoA dehydrogenase family member 11
Q80XL6	*Acad11*	Acyl-CoA dehydrogenase family member 11
Q5F485	*DDX42*	ATP-dependent RNA helicase DDX42
Q6GLQ4	*ppp1r10*	Serine/threonine-protein phosphatase 1 regulatory subunit 10
Q13042	*CDC16*	Cell division cycle protein 16 homolog
Q8CE22	*Dmtf1*	Cyclin-D-binding Myb-like transcription factor 1
Q53GQ0	*HSD17B12*	Very-long-chain 3-oxoacyl-CoA reductase
Q8AWD2	*mocs3*	Adenylyltransferase and sulfurtransferase MOCS3

Q9NWU1	*OXSM*	3-oxoacyl-[acyl-carrier-protein] synthase, mitochondrial
Q6INP0	*smim7-a*	Small integral membrane protein 7-A
Q2KIR8	*TDH*	L-threonine 3-dehydrogenase, mitochondrial
Q1JQD2	*GRWD1*	Glutamate-rich WD repeat-containing protein 1
P38652	*Pgm1*	Phosphoglucomutase-1
Q9CWZ7	*Napg*	Gamma-soluble NSF attachment protein
Q9R1P4	*Psma1*	Proteasome subunit alpha type-1
O62703	*CTNNBL1*	Beta-catenin-like protein 1
P48029	*SLC6A8*	Sodium- and chloride-dependent creatine transporter 1
P35822	*Ptprk*	Receptor-type tyrosine-protein phosphatase kappa
P08537	*tuba*	Tubulin alpha chain
Q9ET22	*Dpp7*	Dipeptidyl peptidase 2
Q3TNH5	*Fam172a*	Protein FAM172A
Q9P0K9	*FRRS1L*	DOMON domain-containing protein FRRS1L
Q64674	*Srm*	Spermidine synthase
Q6NUQ1	*RINT1*	RAD50-interacting protein 1
P22124		Ras-related protein O-RAL
O18835	*GUSB*	Beta-glucuronidase
Q6NRS1	*ibtk*	Inhibitor of Bruton tyrosine kinase
Q63ZP1	*phf10*	PHD finger protein 10
Q0MQF0	*NDUFB9*	NADH dehydrogenase [ubiquinone] 1 beta subcomplex subunit 9
Q62276	*Med22*	Mediator of RNA polymerase II transcription subunit 22
Q3SWX9	*RAD21*	Double-strand-break repair protein rad21 homolog
P48065	*SLC6A12*	Sodium- and chloride-dependent betaine transporter
O00429	*DNM1L*	Dynamin-1-like protein
Q09YI9	*MET*	Hepatocyte growth factor receptor

Q6PCI7	*mcm5-b*	DNA replication licensing factor mcm5-B
Q9V3D2	*Coprox*	Oxygen-dependent coproporphyrinogen-III oxidase
Q9CSH3	*Dis3*	Exosome complex exonuclease RRP44
Q8K1M6	*Dnm1l*	Dynamin-1-like protein
Q7SXN5	*dnm1l*	Dynamin-1-like protein
P14755	*CRYL1*	Lambda-crystallin
Q99KP3	*Cryl1*	Lambda-crystallin homolog
P68372	*Tubb4b*	Tubulin beta-4B chain
Q9HC10	*OTOF*	Otoferlin
Q5FWF5	*ESCO1*	N-acetyltransferase ESCO1
Q96MD2	*C12orf66*	UPF0536 protein C12orf66
Q66HF1	*Ndufs1*	NADH-ubiquinone oxidoreductase 75 kDa subunit, mitochondrial
P42696	*RBM34*	RNA-binding protein 34
P19440	*GGT1*	Gamma-glutamyltranspeptidase 1
Q9DGN1	*stag1*	Cohesin subunit SA-1
Q9DCZ1	*Gmpr*	GMP reductase 1
Q5JXM2	*METTL24*	Methyltransferase-like protein 24
Q21828	*R07E5.13*	Probable mitochondrial pyruvate carrier 1
Q96RQ3	*MCCC1*	Methylcrotonoyl-CoA carboxylase subunit alpha, mitochondrial
Q5ZL34	*CPSF6*	Cleavage and polyadenylation specificity factor subunit 6
Q5M8Y1	*spcs2*	Probable signal peptidase complex subunit 2
Q9D8I1	*Mzb1*	Marginal zone B- and B1-cell-specific protein
Q02853	*Mmp11*	Stromelysin-3
Q5T200	*ZC3H13*	Zinc finger CCCH domain-containing protein 13
Q91YE6	*Ipo9*	Importin-9
O70333	*Cript*	Cysteine-rich PDZ-binding protein

Q0D289	*zgc:153595*	Uncharacterized protein C16orf52 homolog
P52170	*kpna1*	Importin subunit alpha-5
Q5ZIW2	*CNOT10*	CCR4-NOT transcription complex subunit 10
P17152	*TMEM11*	Transmembrane protein 11, mitochondrial
Q6DI40	*ttc33*	Tetratricopeptide repeat protein 33
Q4V8Y6	*ergic1*	Endoplasmic reticulum-Golgi intermediate compartment protein 1
Q91Y81	Septin2	Septin-2
Q02372	*NDUFB8*	NADH dehydrogenase [ubiquinone] 1 beta subcomplex subunit 8, mitochondrial
P06596	*PLA2G1B*	Phospholipase A2
Q0MQC8	*NDUFB2*	NADH dehydrogenase [ubiquinone] 1 beta subcomplex subunit 2, mitochondrial
O75521	*ECI2*	Enoyl-CoA delta isomerase 2, mitochondrial
Q8N4V2	*SVOP*	Synaptic vesicle 2-related protein
Q8HYL8	*ACOX1*	Peroxisomal acyl-coenzyme A oxidase 1
Q6DBY0	*nup85*	Nuclear pore complex protein Nup85
Q64350	*Eif2b5*	Translation initiation factor eIF-2B subunit epsilon
Q54RF3	*eif2b5*	Translation initiation factor eIF-2B subunit epsilon
Q3ZBV8	*TARS*	Threonine--tRNA ligase, cytoplasmic
Q9NBX4	*X-element\ORF2*	Probable RNA-directed DNA polymerase from transposon X-element
Q05329	*GAD2*	Glutamate decarboxylase 2
Q9DBE0	*Csad*	Cysteine sulfinic acid decarboxylase
P70333	*Hnrnph2*	Heterogeneous nuclear ribonucleoprotein H2
P31942	*HNRNPH3*	Heterogeneous nuclear ribonucleoprotein H3
Q92963	*RIT1*	GTP-binding protein Rit1
Q60855	*Ripk1*	Receptor-interacting serine/threonine-protein kinase 1
Q0VC50	*RTN4IP1*	Reticulon-4-interacting protein 1, mitochondrial
Q05086	*UBE3A*	Ubiquitin-protein ligase E3A

O08738	*Casp6*	Caspase-6
P47863	*Aqp4*	Aquaporin-4
Q921W4	*Cryzl1*	Quinone oxidoreductase-like protein 1
A0JN27	*Gtf2h2*	General transcription factor IIH subunit 2
Q91YY4	*Atpaf2*	ATP synthase mitochondrial F1 complex assembly factor 2
Q6N021	*TET2*	Methylcytosine dioxygenase TET2
Q5ZME8	*SMU1*	WD40 repeat-containing protein SMU1
Q8IW19	*APLF*	Aprataxin and PNK-like factor
Q64L89	*MGST1*	Microsomal glutathione S-transferase 1
B2GUP8	*abcb8*	ATP-binding cassette sub-family B member 8, mitochondrial
Q00059	*TFAM*	Transcription factor A, mitochondrial
O02414		Dynein light chain LC6, flagellar outer arm
Q8WXE1	*ATRIP*	ATR-interacting protein
Q3T0V7	*EDF1*	Endothelial differentiation-related factor 1
Q99LD9	*Eif2b2*	Translation initiation factor eIF-2B subunit beta
P27641	*Xrcc5*	X-ray repair cross-complementing protein 5
Q02391	*GLG1*	Golgi apparatus protein 1
P48375	*FK506-bp2*	12 kDa FK506-binding protein
P14604	*Echs1*	Enoyl-CoA hydratase, mitochondrial
O55166	*Vps52*	Vacuolar protein sorting-associated protein 52 homolog
Q9HBH5	*RDH14*	Retinol dehydrogenase 14
Q6UWH6	*TEX261*	Protein TEX261
Q9WUK2	*Eif4h*	Eukaryotic translation initiation factor 4H
Q5ZJL9	*SAMHD1*	Deoxynucleoside triphosphate triphosphohydrolase SAMHD1
Q8VE97	*Srsf4*	Serine/arginine-rich splicing factor 4
Q3ULD5	*Mccc2*	Methylcrotonoyl-CoA carboxylase beta chain, mitochondrial

Q96PE7	*MCEE*	Methylmalonyl-CoA epimerase, mitochondrial
Q803F5	*apmap*	Adipocyte plasma membrane-associated protein
P38487		Creatinase
Q1RMR1	*ANGPTL1*	Angiopoietin-related protein 1
P49446	*Ptpre*	Receptor-type tyrosine-protein phosphatase epsilon
P56399	*Usp5*	Ubiquitin carboxyl-terminal hydrolase 5
P09215	*Prkcd*	Protein kinase C delta type
O08788	*Dctn1*	Dynactin subunit 1
Q6NWH1	*dcaf10*	DDB1- and CUL4-associated factor 10
Q2HJ33	*OLA1*	Obg-like ATPase 1
Q0V9P1	*hnmt*	Histamine N-methyltransferase
Q8R127	*Sccpdh*	Saccharopine dehydrogenase-like oxidoreductase
Q6A4L0	*Slc22a13*	Solute carrier family 22 member 13
P60901	*Psma6*	Proteasome subunit alpha type-6
O19072	*OTC*	Ornithine carbamoyltransferase, mitochondrial (Fragment)
Q2VL90	*CD163*	Scavenger receptor cysteine-rich type 1 protein M130
Q86SQ4	*GPR126*	G-protein coupled receptor 126
Q5R816	*ABHD14B*	Alpha/beta hydrolase domain-containing protein 14B
P12270	*TPR*	Nucleoprotein TPR
Q66IF1	*reep6*	Receptor expression-enhancing protein 6
Q9BXF3	*CECR2*	Cat eye syndrome critical region protein 2
Q0VC24	*WDR12*	Ribosome biogenesis protein WDR12
Q04336	*YMR196W*	Uncharacterized protein YMR196W
Q5M8F7	*cnep1r1*	Nuclear envelope phosphatase-regulatory subunit 1
Q99N23	*Ca15*	Carbonic anhydrase 15
Q80VP5		Probable peptide chain release factor C12orf65 homolog, mitochondrial

Q95SX7	*RTase*	Probable RNA-directed DNA polymerase from transposon BS
Q69YN4	*KIAA1429*	Protein virilizer homolog
Q9H5Z1	*DHX35*	Probable ATP-dependent RNA helicase DHX35
Q91YN5	*Uap1*	UDP-N-acetylhexosamine pyrophosphorylase
Q6PEI3	*phactr4b*	Phosphatase and actin regulator 4B
Q9Y4P3	*TBL2*	Transducin beta-like protein 2
P12815	*Pdcd6*	Programmed cell death protein 6
Q5F3U9	*PDS5B*	Sister chromatid cohesion protein PDS5 homolog B
Q03168	*AAEL006169*	Lysosomal aspartic protease
Q9VND8	*Rheb*	GTP-binding protein Rheb homolog
Q91W59	*Rbms1*	RNA-binding motif, single-stranded-interacting protein 1
Q9JK42	*Pdk2*	[Pyruvate dehydrogenase (acetyl-transferring)] kinase isozyme 2, mitochondrial
A9UMP7	*eno4*	Enolase-like protein ENO4
O00625	*PIR*	Pirin
Q5ZML3	*SRSF1*	Serine/arginine-rich splicing factor 1
P51688	*SGSH*	N-sulphoglucosamine sulphohydrolase
Q9D404	*Oxsm*	3-oxoacyl-[acyl-carrier-protein] synthase, mitochondrial
Q5EA75	*PCYT2*	Ethanolamine-phosphate cytidylyltransferase
P22464	*AnxB9*	Annexin B9
P57737	*CORO7*	Coronin-7
Q5R885	*UXS1*	UDP-glucuronic acid decarboxylase 1
Q8CFL8	*Zswim3*	Zinc finger SWIM domain-containing protein 3
Q5RC61	*SH3BGRL3*	SH3 domain-binding glutamic acid-rich-like protein 3
Q9CWP8	*Pold4*	DNA polymerase delta subunit 4
Q8BZX4	*Srek1*	Splicing regulatory glutamine/lysine-rich protein 1
Q9P2J5	*LARS*	Leucine--tRNA ligase, cytoplasmic

Q8BL97	*Srsf7*	Serine/arginine-rich splicing factor 7
Q3T106	*SRSF7*	Serine/arginine-rich splicing factor 7
B7ZCC9	*Adgrg4*	Adhesion G-protein coupled receptor G4
C6KFA3	*gpr126*	G-protein coupled receptor 126
A7Z035	*CLINT1*	Clathrin interactor 1
Q9W6B4	*TIMP3*	Metalloproteinase inhibitor 3
Q28GH3	*uba2*	SUMO-activating enzyme subunit 2
P54985	*CYPA*	Peptidyl-prolyl cis-trans isomerase
Q8VIJ6	*Sfpq*	Splicing factor, proline- and glutamine-rich
Q6GR10	*maea*	Macrophage erythroblast attacher
B2RZ17	*Fbxw2*	F-box/WD repeat-containing protein 2
Q497B8	*Kdm8*	Lysine-specific demethylase 8
D2GVP7	*STAU1*	Double-stranded RNA-binding protein Staufen homolog 1
Q7YQM0	*GDI1*	Rab GDP dissociation inhibitor alpha
Q7TPQ3	*Shprh*	E3 ubiquitin-protein ligase SHPRH
Q7XJJ7	*FAAH*	Fatty acid amide hydrolase
Q8BH60	*Gopc*	Golgi-associated PDZ and coiled-coil motif-containing protein
Q9P0U1	*TOMM7*	Mitochondrial import receptor subunit TOM7 homolog
Q6GMK8	*gmppaa*	Mannose-1-phosphate guanyltransferase alpha-A
Q4R627	*LARP7*	La-related protein 7
Q9DBU0	*Tm9sf1*	Transmembrane 9 superfamily member 1
Q9DCJ9	*Npl*	N-acetylneuraminate lyase
O77649	*UGT2B20*	UDP-glucuronosyltransferase 2B20
B4F6U4	*prdm10*	PR domain zinc finger protein 10
A6BM72	*MEGF11*	Multiple epidermal growth factor-like domains protein 11
Q8IYB7	*DIS3L2*	DIS3-like exonuclease 2

O13022	*dpysl3-a*	Dihydropyrimidinase-related protein 3-A
Q9T0A0	*LACS4*	Long chain acyl-CoA synthetase 4
O75140	*DEPDC5*	DEP domain-containing protein 5
P02605		Myosin light chain 3, skeletal muscle isoform
Q4R591	*GPI*	Glucose-6-phosphate isomerase
P99029	*Prdx5*	Peroxiredoxin-5, mitochondrial
Q02556	*IRF8*	Interferon regulatory factor 8
P04040	*CAT*	Catalase
P42167	*TMPO*	Lamina-associated polypeptide 2, isoforms beta/gamma
P00172	*CYB5A*	Cytochrome b5
Q5VWX1	*KHDRBS2*	KH domain-containing, RNA-binding, signal transduction-associated protein 2
Q8CHT3	*Ints5*	Integrator complex subunit 5
P35659	*DEK*	Protein DEK
Q9VGY6	*Skeletor*	Protein Skeletor, isoforms B/C
P70699	*Gaa*	Lysosomal alpha-glucosidase
Q8AWW5	*CRIM1*	Cysteine-rich motor neuron 1 protein
Q9JLL0	*Crim1*	Cysteine-rich motor neuron 1 protein
P80017		Globin D, coelomic
F1N9Y5	*SYK*	Tyrosine-protein kinase SYK
Q78P75	*Dynll2*	Dynein light chain 2, cytoplasmic
Q0VFD8	*bloc1s1*	Biogenesis of lysosome-related organelles complex 1 subunit 1
P68907	*Pdzrn3*	E3 ubiquitin-protein ligase PDZRN3
Q9Y5K3	*PCYT1B*	Choline-phosphate cytidylyltransferase B
Q7ZX22	*wdr24*	WD repeat-containing protein 24
Q61704	*Itih3*	Inter-alpha-trypsin inhibitor heavy chain H3
P24367	*PPIB*	Peptidyl-prolyl cis-trans isomerase B

Q13315	*ATM*	Serine-protein kinase ATM
O54921	*Exoc2*	Exocyst complex component 2
Q9WU19	*Hao1*	Hydroxyacid oxidase 1
O35250	*Exoc7*	Exocyst complex component 7
Q80W68	*Kirrel*	Kin of IRRE-like protein 1
O02485	*ZK1073.1*	Uncharacterized protein ZK1073.1
O75323	*GBAS*	Protein NipSnap homolog 2
P0C1J8	*pin1*	Peptidyl-prolyl cis-trans isomerase pin1
Q4KMQ2	*ANO6*	Anoctamin-6
Q27128		Bifunctional 3'-phosphoadenosine 5'-phosphosulfate synthase
P26440	*IVD*	Isovaleryl-CoA dehydrogenase, mitochondrial
O08795	*Prkcsh*	Glucosidase 2 subunit beta
Q99643	*SDHC*	Succinate dehydrogenase cytochrome b560 subunit, mitochondrial
Q9BYP7	*WNK3*	Serine/threonine-protein kinase WNK3
Q5R7G6	*NPEPL1*	Probable aminopeptidase NPEPL1
Q91060		Tubulin alpha chain
P31483	*TIA1*	Nucleolysin TIA-1 isoform p40
F6RQL9	*MTAP*	S-methyl-5'-thioadenosine phosphorylase
Q8BY79	*Slc35g1*	Solute carrier family 35 member G1
Q7ZVR8	*esrp2*	Epithelial splicing regulatory protein 2
Q3KRG3	*tsr2*	Pre-rRNA-processing protein TSR2 homolog
P97819	*Pla2g6*	85/88 kDa calcium-independent phospholipase A2
P00349	*PGD*	6-phosphogluconate dehydrogenase, decarboxylating
Q12873	*CHD3*	Chromodomain-helicase-DNA-binding protein 3
Q6ZJ05	*URH1*	Probable uridine nucleosidase 1
P55864	*pax6*	Paired box protein Pax-6

O97159	*Mi-2*	Chromodomain-helicase-DNA-binding protein Mi-2 homolog
Q8C854	*Myef2*	Myelin expression factor 2
P41562	*Idh1*	Isocitrate dehydrogenase [NADP] cytoplasmic
Q6XUZ5	*IDH1*	Isocitrate dehydrogenase [NADP] cytoplasmic
Q9WV52	*Plek2*	Pleckstrin-2
P29361	*YWHAZ*	14-3-3 protein zeta/delta
Q5ZLY5	*PLEKHF2*	Pleckstrin homology domain-containing family F member 2
Q5VYX0	*RNLS*	Renalase
P61979	*Hnrnpk*	Heterogeneous nuclear ribonucleoprotein K
Q2TBV1	*RFC3*	Replication factor C subunit 3
Q9JII5	*Dazap1*	DAZ-associated protein 1
Q3UUJ4	*Strada*	STE20-related kinase adapter protein alpha
Q63186	*Eif2b4*	Translation initiation factor eIF-2B subunit delta
P05094	*ACTN1*	Alpha-actinin-1
Q5ZMW5	*ARHGAP26*	Rho GTPase-activating protein 26
P41252	*IARS*	Isoleucine--tRNA ligase, cytoplasmic
Q9NQR9	*G6PC2*	Glucose-6-phosphatase 2
Q96K80	*ZC3H10*	Zinc finger CCCH domain-containing protein 10
Q4KLN6	*Rrm2*	Ribonucleoside-diphosphate reductase subunit M2
P41216	*Acsl1*	Long-chain-fatty-acid--CoA ligase 1
A2TIL1	*GSR*	Glutathione reductase, mitochondrial
Q7T3H1	*dctn2*	Dynactin subunit 2
Q9WTP6	*Ak2*	Adenylate kinase 2, mitochondrial
Q8BXB6	*Slco2b1*	Solute carrier organic anion transporter family member 2B1
B3P6T8	*bai*	Transmembrane emp24 domain-containing protein bai
P57103	*SLC8A3*	Sodium/calcium exchanger 3

Q9CPU4	*Mgst3*	Microsomal glutathione S-transferase 3
O35737	*Hnrnph1*	Heterogeneous nuclear ribonucleoprotein H
A1XD93	*TFIP11*	Tuftelin-interacting protein 11
Q6EAS5	*GBE1*	1,4-alpha-glucan-branching enzyme
Q8MJY8	*HPGD*	15-hydroxyprostaglandin dehydrogenase [NAD(+)]
Q8BMA6	*Srp68*	Signal recognition particle subunit SRP68
P40122	*CAP*	Adenylyl cyclase-associated protein
P49907	*SEPP1*	Selenoprotein P
P0DMQ6	*SORD*	Sorbitol dehydrogenase
P60983	*GMFB*	Glia maturation factor beta
P60984	*GMFB*	Glia maturation factor beta
Q9Z127	*Slc7a5*	Large neutral amino acids transporter small subunit 1
Q25117		ATP synthase subunit beta, mitochondrial
P13437	*Acaa2*	3-ketoacyl-CoA thiolase, mitochondrial
Q6PGA0	*Rcor3*	REST corepressor 3
Q96SZ4	*ZSCAN10*	Zinc finger and SCAN domain-containing protein 10
Q9NFZ4		Tropomyosin
P07511	*SHMT1*	Serine hydroxymethyltransferase, cytosolic
B3MI37	*GF11110*	Succinate dehydrogenase assembly factor 2-A, mitochondrial
Q61133	*Gstt2*	Glutathione S-transferase theta-2
P60174	*TPI1*	Triosephosphate isomerase
Q9DBB9	*Cpn2*	Carboxypeptidase N subunit 2
Q8R081	*Hnrnpl*	Heterogeneous nuclear ribonucleoprotein L
Q29RT0	*RBMX*	RNA-binding motif protein, X chromosome
Q0IIY2	*wscd1*	WSC domain-containing protein 1
Q7SZR5	*sumo1*	Small ubiquitin-related modifier 1

Q8C3F2	*Fam120c*	Constitutive coactivator of PPAR-gamma-like protein 2
Q6B855	*TKT*	Transketolase
P11833		Tubulin beta chain
Q9YHC3		Tubulin beta-1 chain
A2VD92	*ddx1*	ATP-dependent RNA helicase DDX1
Q09028	*RBBP4*	Histone-binding protein RBBP4
Q8K4S1	*Plce1*	1-phosphatidylinositol 4,5-bisphosphate phosphodiesterase epsilon-1
P25007	*Cyp1*	Peptidyl-prolyl cis-trans isomerase
Q29RU1	*MRPS12*	28S ribosomal protein S12, mitochondrial
Q80X72	*Lrrc15*	Leucine-rich repeat-containing protein 15
Q7PKQ5	*Actn*	Alpha-actinin, sarcomeric
Q86UF1	*TSPAN33*	Tetraspanin-33
P08575	*PTPRC*	Receptor-type tyrosine-protein phosphatase C
P78417	*GSTO1*	Glutathione S-transferase omega-1
P16264		Egg peptide speract receptor
P97852	*Hsd17b4*	Peroxisomal multifunctional enzyme type 2
Q96BM0	*IFI27L1*	Interferon alpha-inducible protein 27-like protein 1
Q9D0K0	*Tbc1d7*	TBC1 domain family member 7
Q96PN7	*TRERF1*	Transcriptional-regulating factor 1
Q95LL3	*TBC1D22B*	TBC1 domain family member 22B
Q15417	*CNN3*	Calponin-3
P57113	*Gstz1*	Maleylacetoacetate isomerase
P19615		Major yolk protein
P02640	*VIL1*	Villin-1
Q5XI73	*Arhgdia*	Rho GDP-dissociation inhibitor 1
P35346	*SSTR5*	Somatostatin receptor type 5

P10079	*EGF1*	Fibropellin-1
P50475	*Aars*	Alanine--tRNA ligase, cytoplasmic
Q9W0T1	*E(bx)*	Nucleosome-remodeling factor subunit NURF301
Q5ZME2	*MDH1*	Malate dehydrogenase, cytoplasmic
Q55EX9	*DDB_G0268948*	Putative methyltransferase DDB_G0268948
Q0MQI5	*NDUFV1*	NADH dehydrogenase [ubiquinone] flavoprotein 1, mitochondrial
Q8VDM6	*Hnrnpul1*	Heterogeneous nuclear ribonucleoprotein U-like protein 1
P05065	*Aldoa*	Fructose-bisphosphate aldolase A
P48430	*SOX2*	Transcription factor SOX-2
Q5U4Y8	*seh1l*	Nucleoporin seh1
Q28521	*HNRNPA1*	Heterogeneous nuclear ribonucleoprotein A1
Q9Y6X9	*MORC2*	MORC family CW-type zinc finger protein 2
Q9UPN3	*MACF1*	Microtubule-actin cross-linking factor 1, isoforms 1/2/3/5
Q8CGC6	*Rbm28*	RNA-binding protein 28
P52899	*T05H10.6*	Probable pyruvate dehydrogenase E1 component subunit alpha, mitochondrial
Q9UM22	*EPDR1*	Mammalian ependymin-related protein 1
Q8MPM1	*gelsolin*	Gelsolin-like protein 2
A8E657	*AASS*	Alpha-aminoadipic semialdehyde synthase, mitochondrial
P12024	*chp*	Chaoptin
Q9NRD9	*DUOX1*	Dual oxidase 1
P34943	*NDUFA9*	NADH dehydrogenase [ubiquinone] 1 alpha subcomplex subunit 9, mitochondrial
O75347	*TBCA*	Tubulin-specific chaperone A
Q32PX7	*Fubp1*	Far upstream element-binding protein 1
Q32P51	*HNRNPA1L2*	Heterogeneous nuclear ribonucleoprotein A1-like 2
Q6NXG1	*ESRP1*	Epithelial splicing regulatory protein 1
P02545	*LMNA*	Prelamin-A/C

Q4UMH6	*RF_0381*	Putative ankyrin repeat protein RF_0381
P23229	*ITGA6*	Integrin alpha-6
P18258		Tubulin alpha-1 chain
P22271	*PROA*	Profilin-A
O17482	*tim*	Protein timeless
Q1HG43	*DUOXA1*	Dual oxidase maturation factor 1
Q24439	*Oscp*	ATP synthase subunit O, mitochondrial
O93277	*WDR1*	WD repeat-containing protein 1
Q8HXP3	*SOD2*	Superoxide dismutase [Mn], mitochondrial
Q27294	*caz*	RNA-binding protein cabeza
Q03173	*Enah*	Protein enabled homolog
O76536		Hyalin (Fragment)
Q9NS73	*MBIP*	MAP3K12-binding inhibitory protein 1
O57590	*surf4*	Surfeit locus protein 4
Q64428	*Hadha*	Trifunctional enzyme subunit alpha, mitochondrial
Q29554	*HADHA*	Trifunctional enzyme subunit alpha, mitochondrial
Q7TMK9	*Syncrip*	Heterogeneous nuclear ribonucleoprotein Q
Q98SJ2	*dazap1*	DAZ-associated protein 1
Q5ZLF0	*ST13*	Hsc70-interacting protein
C3YWU0	*BRAFLDRAFT_56888*	Alpha-L-fucosidase
Q96EB6	*SIRT1*	NAD-dependent protein deacetylase sirtuin-1
Q561L1	*adssl1-a*	Adenylosuccinate synthetase isozyme 1 A
Q9UNH7	*SNX6*	Sorting nexin-6
Q0MQB9	*NDUFA11*	NADH dehydrogenase [ubiquinone] 1 alpha subcomplex subunit 11
Q5EA65	*DPAGT1*	UDP-N-acetylglucosamine--dolichyl-phosphate N-acetylglucosaminephosphotransferase
P38024	*AIRC*	Multifunctional protein ADE2

Q16740	*CLPP*	ATP-dependent Clp protease proteolytic subunit, mitochondrial
P14314	*PRKCSH*	Glucosidase 2 subunit beta
Q96Q15	*SMG1*	Serine/threonine-protein kinase SMG1
Q3ZBG9	*PLSCR2*	Phospholipid scramblase 2
P07686	*HEXB*	Beta-hexosaminidase subunit beta
Q96PZ7	*CSMD1*	CUB and sushi domain-containing protein 1
P07207	*N*	Neurogenic locus Notch protein
Q9YGD2	*SUCLG1*	Succinyl-CoA ligase subunit alpha, mitochondrial (Fragment)
P28491	*CALR*	Calreticulin
O93336	*hs2st1*	Heparan sulfate 2-O-sulfotransferase 1
Q01205	*Dlst*	Dihydrolipoyllysine-residue succinyltransferase component of 2-oxoglutarate dehydrogenase complex, mitochondrial
P21956	*Mfge8*	Lactadherin
Q9VT65	*CalpB*	Calpain-B
Q9UGT4	*SUSD2*	Sushi domain-containing protein 2
P42765	*ACAA2*	3-ketoacyl-CoA thiolase, mitochondrial
A7RNG8	*v1g180167*	Coiled-coil domain-containing protein 22 homolog
Q5ZJ73	*CREG1*	Protein CREG1
Q99M80	*Ptprt*	Receptor-type tyrosine-protein phosphatase T
Q9CZ28	*Snf8*	Vacuolar-sorting protein SNF8
Q8TAQ2	*SMARCC2*	SWI/SNF complex subunit SMARCC2
O75131	*CPNE3*	Copine-3
P48450	*Lss*	Lanosterol synthase
P25788	*PSMA3*	Proteasome subunit alpha type-3
O54694	*SPTLC2*	Serine palmitoyltransferase 2
Q9V496	*Rfabg*	Apolipophorins
O08747	*Unc5c*	Netrin receptor UNC5C

Q8VCH0	*Acaa1b*	3-ketoacyl-CoA thiolase B, peroxisomal
Q8IZF6	*ADGRG4*	Adhesion G-protein coupled receptor G4
Q9D0L8	*Rnmt*	mRNA cap guanine-N7 methyltransferase
Q50JE5	*Ace*	Angiotensin-converting enzyme
Q13685	*AAMP*	Angio-associated migratory cell protein
O35449	*Prrt1*	Proline-rich transmembrane protein 1
Q63ZQ1	*sept2-b*	Septin-2B
Q8BGC0	*Htatsf1*	HIV Tat-specific factor 1 homolog
Q58D34	*PI16*	Peptidase inhibitor 16
Q5RBR7	*LYPLA1*	Acyl-protein thioesterase 1
Q802T2	*ALG6*	Dolichyl pyrophosphate Man9GlcNAc2 alpha-1,3-glucosyltransferase
Q9Y244	*POMP*	Proteasome maturation protein
Q92616	*GCN1L1*	Translational activator GCN1
Q9CWK8	*Snx2*	Sorting nexin-2
Q8BYB9	*Poglut1*	Protein O-glucosyltransferase 1
P54996	*g6pd*	Glucose-6-phosphate 1-dehydrogenase
O73791	*tie2*	Tyrosine-protein kinase receptor Tie-2
Q96T23	*RSF1*	Remodeling and spacing factor 1
P05370	*G6pdx*	Glucose-6-phosphate 1-dehydrogenase
A7SG73	*qprt*	Nicotinate-nucleotide pyrophosphorylase [carboxylating] (Fragment)
O31463	*ybgG*	Homocysteine S-methyltransferase YbgG
P0C6B8	*Svep1*	Sushi, von Willebrand factor type A, EGF and pentraxin domain-containing protein 1
Q6MG48	*Prrc2a*	Protein PRRC2A
P31943	*HNRNPH1*	Heterogeneous nuclear ribonucleoprotein H
Q12872	*SFSWAP*	Splicing factor, suppressor of white-apricot homolog
Q9HC24	*TMBIM4*	Protein lifeguard 4

Q6NV83	*U2surp*	U2 snRNP-associated SURP motif-containing protein
Q5ZL77	*RIC8A*	Synembryn-A
Q26613	*EMAP*	77 kDa echinoderm microtubule-associated protein
O49923	*ADK*	Adenosine kinase
Q8HY31	*UROD*	Uroporphyrinogen decarboxylase
O61577	*KATNA1*	Katanin p60 ATPase-containing subunit A1
Q14114	*LRP8*	Low-density lipoprotein receptor-related protein 8
Q5REX3	*ZFR*	Zinc finger RNA-binding protein
Q9UBF6	*RNF7*	RING-box protein 2
Q5ZMG1	*UFL1*	E3 UFM1-protein ligase 1
Q60756	*Tcf15*	Transcription factor 15
P14408	*Fh*	Fumarate hydratase, mitochondrial
Q9D4H9	*Phf14*	PHD finger protein 14
Q8BGF6	*Elmod2*	ELMO domain-containing protein 2
Q9V0D5	*mdh*	Malate dehydrogenase
P46721	*SLCO1A2*	Solute carrier organic anion transporter family member 1A2
Q5R6R5	*CMAS*	N-acylneuraminate cytidylyltransferase
Q9Z1Z2	*Strap*	Serine-threonine kinase receptor-associated protein
Q5FYB1	*ARSI*	Arylsulfatase I
Q6DHN0	*tmem53*	Transmembrane protein 53
P31696	*AGRN*	Agrin
Q3T134	*SPCS1*	Signal peptidase complex subunit 1
Q8NBN7	*RDH13*	Retinol dehydrogenase 13
Q8HXG5	*NDUFB11*	NADH dehydrogenase [ubiquinone] 1 beta subcomplex subunit 11, mitochondrial
O15439	*ABCC4*	Multidrug resistance-associated protein 4
Q8N565	*MREG*	Melanoregulin

Q8BMF4	*Dlat*	Dihydrolipoyllysine-residue acetyltransferase component of pyruvate dehydrogenase complex, mitochondrial
Q00277	*GPX1*	Glutathione peroxidase
Q2VLG6	*CD163*	Scavenger receptor cysteine-rich type 1 protein M130
P85521	*CD163*	Scavenger receptor cysteine-rich type 1 protein M130
Q9NR16	*CD163L1*	Scavenger receptor cysteine-rich type 1 protein M160
Q2KJ81	*AP1M1*	AP-1 complex subunit mu-1
P55250	*FUMR*	Fumarate hydratase, mitochondrial
Q9PRL8	*DBI*	Acyl-CoA-binding protein
Q9ULI0	*ATAD2B*	ATPase family AAA domain-containing protein 2B
P19070	*Cr2*	Complement receptor type 2
Q9HI69	*Ta1476*	Glutamate formimidoyltransferase
Q6Q0C0	*TRAF7*	E3 ubiquitin-protein ligase TRAF7
Q0P5G4	*CCBL2*	Kynurenine--oxoglutarate transaminase 3
P28827	*PTPRM*	Receptor-type tyrosine-protein phosphatase mu
P84169	*PSMD13*	26S proteasome non-ATPase regulatory subunit 13
Q9NYT0	*PLEK2*	Pleckstrin-2
Q9UNK0	*STX8*	Syntaxin-8
Q28BZ2	*hint3*	Histidine triad nucleotide-binding protein 3
Q4SBY6	*hnmt*	Histamine N-methyltransferase
Q67ER4	*RNF113A*	RING finger protein 113A
P11024	*NNT*	NAD(P) transhydrogenase, mitochondrial
Q9UNX4	*WDR3*	WD repeat-containing protein 3
Q9QZU7	*Bbox1*	Gamma-butyrobetaine dioxygenase
P34896	*SHMT1*	Serine hydroxymethyltransferase, cytosolic
P68943	*Med28*	Mediator of RNA polymerase II transcription subunit 28
Q99L13	*Hibadh*	3-hydroxyisobutyrate dehydrogenase, mitochondrial

Q9Y0Y7	*CG2061*	LanC-like protein 3 homolog
A2AR02	*Ppig*	Peptidyl-prolyl cis-trans isomerase G
P29400	*COL4A5*	Collagen alpha-5(IV) chain
P00488	*F13A1*	Coagulation factor XIII A chain
P40123	*CAP2*	Adenylyl cyclase-associated protein 2
A5D989	*EEF1D*	Elongation factor 1-delta
P42620	*yqjG*	Glutathionyl-hydroquinone reductase YqjG
Q9R050	*Ssbp3*	Single-stranded DNA-binding protein 3
Q24524	*sn*	Protein singed
O75828	*CBR3*	Carbonyl reductase [NADPH] 3
Q7TMS5	*Abcg2*	ATP-binding cassette sub-family G member 2
P52297	*kpnb1*	Importin subunit beta
Q6Q7P4	*EPB41*	Protein 4.1
Q9NWY4	*C4orf27*	UPF0609 protein C4orf27
Q6AXS5	*Serbp1*	Plasminogen activator inhibitor 1 RNA-binding protein
P61008	*SPCS3*	Signal peptidase complex subunit 3
Q64516	*Gk*	Glycerol kinase
P17955	*Nup62*	Nuclear pore glycoprotein p62
A4IG62	*supv3l1*	ATP-dependent RNA helicase SUPV3L1, mitochondrial
Q58DA6	*SLC35A2*	UDP-galactose translocator
Q63150	*Dpys*	Dihydropyrimidinase
A2ALW5	*Dnajc25*	DnaJ homolog subfamily C member 25
Q28XE2	*Anp32a*	Acidic leucine-rich nuclear phosphoprotein 32 family member A
A6H791	*TRMT61A*	tRNA (adenine(58)-N(1))-methyltransferase catalytic subunit TRMT61A
P61759	*Vbp1*	Prefoldin subunit 3
Q69ZL1	*Fgd6*	FYVE, RhoGEF and PH domain-containing protein 6

Q59E55	*nab*	NGFI-A-binding protein homolog
Q91XC8	*Dap*	Death-associated protein 1
Q7ZXB1	*mcm7-b*	DNA replication licensing factor mcm7-B
P28828	*Ptprm*	Receptor-type tyrosine-protein phosphatase mu
Q7Z2Q7	*LRRC70*	Leucine-rich repeat-containing protein 70
O14562	*UBFD1*	Ubiquitin domain-containing protein UBFD1
O57478	*fech*	Ferrochelatase, mitochondrial
Q8CFX8	*Aars*	Alanine--tRNA ligase, cytoplasmic
Q5XK83	*mcm4-a*	DNA replication licensing factor mcm4-A
Q8NBI5	*SLC43A3*	Solute carrier family 43 member 3
P16930	*FAH*	Fumarylacetoacetase
P00637	*FBP1*	Fructose-1,6-bisphosphatase 1
B5FXE5	*AIFM2*	Apoptosis-inducing factor 2
Q9Y210	*TRPC6*	Short transient receptor potential channel 6
Q96NU1	*SAMD11*	Sterile alpha motif domain-containing protein 11
Q05187		Hemocyte protein-glutamine gamma-glutamyltransferase
Q6DCU7		Protein Hikeshi
O00370		LINE-1 retrotransposable element ORF2 protein
Q6NWD4	*timm50*	Mitochondrial import inner membrane translocase subunit TIM50
Q9BTT6	*LRRC1*	Leucine-rich repeat-containing protein 1
Q9Y3Z3	*SAMHD1*	Deoxynucleoside triphosphate triphosphohydrolase SAMHD1
Q7ZY08	*ube2t*	Ubiquitin-conjugating enzyme E2 T
Q4KMD7	*tut1*	Speckle targeted PIP5K1A-regulated poly(A) polymerase
O19069	*SUCLG1*	Succinyl-CoA ligase [ADP/GDP-forming] subunit alpha, mitochondrial
P52757	*CHN2*	Beta-chimaerin
Q5PNP1	*snx14*	Sorting nexin-14

P51968		Heterogeneous nuclear ribonucleoprotein A3 homolog 1
P0CG30	*GSTT2B*	Glutathione S-transferase theta-2B
O46629	*HADHB*	Trifunctional enzyme subunit beta, mitochondrial
P56518	*HDAC1*	Histone deacetylase 1
P07687	*Ephx1*	Epoxide hydrolase 1
Q9S850	*SOX*	Sulfite oxidase
Q3SZA9	*MRPL35*	39S ribosomal protein L35, mitochondrial
P53767	*Flt1*	Vascular endothelial growth factor receptor 1
Q6IQ97	*rbm4.1*	RNA-binding protein 4.1
Q32KH5	*GALNS*	N-acetylgalactosamine-6-sulfatase
P0C227		Globin
P37879	*KARS*	Lysine--tRNA ligase
O60524	*NEMF*	Nuclear export mediator factor NEMF
Q80YF0	*MAD1L1*	Mitotic spindle assembly checkpoint protein MAD1
P22027	*ATP5S*	ATP synthase subunit s, mitochondrial
Q28BP9	*faf2*	FAS-associated factor 2
Q8CGE9	*Rgs12*	Regulator of G-protein signaling 12
Q9BQI6	*ANKRD32*	Ankyrin repeat domain-containing protein 32
Q15637	*SF1*	Splicing factor 1
Q80XH4	*Wscd1*	WSC domain-containing protein 1
P06180		Histone-binding protein N1/N2
Q6AY04		Uncharacterized protein C2orf47 homolog, mitochondrial
Q5I7G2	*RXR*	Retinoic acid receptor RXR
Q8INK6	*PGRP-LB*	Peptidoglycan-recognition protein LB
P50533	*smc2*	Structural maintenance of chromosomes protein 2
P82675	*MRPS5*	28S ribosomal protein S5, mitochondrial

Q920B9	*Supt16h*	FACT complex subunit SPT16
Q99NA9	*Pcgf6*	Polycomb group RING finger protein 6
Q9HAR2	*LPHN3*	Latrophilin-3
Q96FV9	*THOC1*	THO complex subunit 1
Q2KJ84	*TMED5*	Transmembrane emp24 domain-containing protein 5
Q1RMV0	*PEX5*	Peroxisomal targeting signal 1 receptor
Q29073	*PTGR1*	Prostaglandin reductase 1
Q1L987	*atpaf1*	ATP synthase mitochondrial F1 complex assembly factor 1
Q99M87	*Dnaja3*	DnaJ homolog subfamily A member 3, mitochondrial
O08618	*Prpsap2*	Phosphoribosyl pyrophosphate synthase-associated protein 2
Q86X55	*CARM1*	Histone-arginine methyltransferase CARM1
Q8WMD0	*COMMD1*	COMM domain-containing protein 1
Q7ZUH1	*zgc:56576*	UPF0568 protein C14orf166 homolog
Q96CD0	*FBXL8*	F-box/LRR-repeat protein 8
O75165	*DNAJC13*	DnaJ homolog subfamily C member 13
Q8BMS1	*Hadha*	Trifunctional enzyme subunit alpha, mitochondrial
Q01406	*CTTN1*	Src substrate protein p85
Q8NBP0	*TTC13*	Tetratricopeptide repeat protein 13
Q9Y296	*TRAPPC4*	Trafficking protein particle complex subunit 4
Q3TXT3	*Inip*	SOSS complex subunit C
Q8WZ42	*TTN*	Titin
Q9VQ79	*AIF*	Putative apoptosis-inducing factor 1, mitochondrial
Q6AY20	*M6pr*	Cation-dependent mannose-6-phosphate receptor
P53590	*SUCLG2*	Succinyl-CoA ligase [GDP-forming] subunit beta, mitochondrial (Fragment)
F1NW29	*TDP2*	Tyrosyl-DNA phosphodiesterase 2
Q8IZP0	*ABI1*	Abl interactor 1

Q6NYT1	*med8*	Mediator of RNA polymerase II transcription subunit 8
Q9DBC0	*Selo*	Selenoprotein O
Q8K194	*Snrnp27*	U4/U6.U5 small nuclear ribonucleoprotein 27 kDa protein
Q7ZY18	*zmcm6-b*	Zygotic DNA replication licensing factor mcm6-B
P13154	*ldh*	Leucine dehydrogenase
Q7TT51	*Lgsn*	Lengsin
Q12830	*BPTF*	Nucleosome-remodeling factor subunit BPTF
B1H369	*hdac8*	Histone deacetylase 8
Q5ZMD2	*ANKMY2*	Ankyrin repeat and MYND domain-containing protein 2
Q6DE55	*hmha1*	Minor histocompatibility protein HA-1
Q75J93	*cpras1*	Circularly permutated Ras protein 1
Q504Y2	*PKDCC*	Extracellular tyrosine-protein kinase PKDCC
Q63955	*Pou4f3*	POU domain, class 4, transcription factor 3
Q6P2B1	*Tnpo3*	Transportin-3
O88851	*Rbbp9*	Putative hydrolase RBBP9
Q5RBJ7	*TM6SF1*	Transmembrane 6 superfamily member 1
Q05752	*NDUFA7*	NADH dehydrogenase [ubiquinone] 1 alpha subcomplex subunit 7
B0R0T1	*si:dkey-18l1.1*	von Willebrand factor A domain-containing protein 8
Q14318	*FKBP8*	Peptidyl-prolyl cis-trans isomerase FKBP8
Q8CIC2	*Nupl2*	Nucleoporin-like protein 2
P63171	*DYNLT1*	Dynein light chain Tctex-type 1
Q06033	*ITIH3*	Inter-alpha-trypsin inhibitor heavy chain H3
A0JMW6	*tmem214-a*	Transmembrane protein 214-A
Q5E980	*CYP20A1*	Cytochrome P450 20A1
P51913	*ENO1*	Alpha-enolase
O35454	*Clcn6*	Chloride transport protein 6

Q2M3R5	*SLC35G1*	Solute carrier family 35 member G1
Q96KP4	*CNDP2*	Cytosolic non-specific dipeptidase
Q9DAY5		UPF0669 protein C6orf120 homolog
O14972	*DSCR3*	Down syndrome critical region protein 3
B0UXP9	*impdh2*	Inosine-5'-monophosphate dehydrogenase 2
Q28ID3	*glrx3*	Glutaredoxin-3
O43772	*SLC25A20*	Mitochondrial carnitine/acylcarnitine carrier protein
O35435	*Dhodh*	Dihydroorotate dehydrogenase (quinone), mitochondrial
P79888	*amd1*	S-adenosylmethionine decarboxylase proenzyme
Q502K2	*samhd1*	Deoxynucleoside triphosphate triphosphohydrolase SAMHD1
C3ZDX5	*BRAFLDRAFT_275690*	Ubiquitin-fold modifier-conjugating enzyme 1
Q9BRT8	*CBWD1*	COBW domain-containing protein 1
Q6DF57	*gpatch11*	G patch domain-containing protein 11
A3QK15	*aacs*	Acetoacetyl-CoA synthetase
A2VDP4	*ZNF567*	Zinc finger protein 567
Q8R0F8	*Fahd1*	Acylpyruvase FAHD1, mitochondrial
Q810B6	*Ankfy1*	Rabankyrin-5
Q3T0V9	*DERA*	Deoxyribose-phosphate aldolase
O09173	*Hgd*	Homogentisate 1,2-dioxygenase
Q9FNE2	*GRXC2*	Glutaredoxin-C2
Q58WW2	*DCAF6*	DDB1- and CUL4-associated factor 6
Q75QI0	*CFDP1*	Craniofacial development protein 1
Q9R1A8	*Rfwd2*	E3 ubiquitin-protein ligase RFWD2
Q8MQH7	*dri*	Protein dead ringer homolog
P23606	*Tgm1*	Protein-glutamine gamma-glutamyltransferase K
Q5ZKS6	*BECN1*	Beclin-1

Q17QC5	*AP2S1*	AP-2 complex subunit sigma
Q05B30	*wdr91*	WD repeat-containing protein 91
Q1MX18	*INSC*	Protein inscuteable homolog
Q9UJA9	*ENPP5*	Ectonucleotide pyrophosphatase/phosphodiesterase family member 5
A7MB78	*GYS1*	Glycogen [starch] synthase, muscle
Q9Z2U1	*Psma5*	Proteasome subunit alpha type-5
Q9JI03	*Col5a1*	Collagen alpha-1(V) chain
Q5VUM1	*SDHAF4*	Succinate dehydrogenase assembly factor 4, mitochondrial
P0C1J6	*FKBP4*	FK506-binding protein 4
Q923B0	*Ggact*	Gamma-glutamylaminecyclotransferase
Q8HXX8	*GCDH*	Glutaryl-CoA dehydrogenase, mitochondrial
Q6P2U9	*cops3*	COP9 signalosome complex subunit 3
Q10656	*egl-15*	Myoblast growth factor receptor egl-15
Q96EQ0	*SGTB*	Small glutamine-rich tetratricopeptide repeat-containing protein beta
Q9UKZ1	*CNOT11*	CCR4-NOT transcription complex subunit 11
P29702	*FNTA*	Protein farnesyltransferase/geranylgeranyltransferase type-1 subunit alpha
Q96Q05	*TRAPPC9*	Trafficking protein particle complex subunit 9
Q4WQS0	*AFUA_4G13800*	Exo-alpha-sialidase
P49119	*CDK2AP1*	Cyclin-dependent kinase 2-associated protein 1
A0AVT1	*UBA6*	Ubiquitin-like modifier-activating enzyme 6
O14735	*CDIPT*	CDP-diacylglycerol--inositol 3-phosphatidyltransferase
O43264	*ZW10*	Centromere/kinetochore protein zw10 homolog
Q0MQG6	*NDUFS3*	NADH dehydrogenase [ubiquinone] iron-sulfur protein 3, mitochondrial
P35269	*GTF2F1*	General transcription factor IIF subunit 1
P21872	*GART*	Trifunctional purine biosynthetic protein adenosine-3
Q7PD79	*G-s-alpha-60A*	Guanine nucleotide-binding protein G(s) subunit alpha

Q9WYG0	*TM_0325*	Uncharacterized oxidoreductase TM_0325
Q13542	*EIF4EBP2*	Eukaryotic translation initiation factor 4E-binding protein 2
Q9DBW3	*Natd1*	Protein NATD1
Q8R431	*Mgll*	Monoglyceride lipase
Q96PQ7	*KLHL5*	Kelch-like protein 5
Q8K4Q6	*Neil1*	Endonuclease 8-like 1
Q08D86	*GK5*	Putative glycerol kinase 5
P61085	*UBE2K*	Ubiquitin-conjugating enzyme E2 K
Q502G5	*zgc:112255*	Uncharacterized protein C1orf50 homolog
P35546	*Ret*	Proto-oncogene tyrosine-protein kinase receptor Ret
Q6AZT4	*brms1l*	Breast cancer metastasis-suppressor 1-like protein
Q803V5	*mlst8*	Target of rapamycin complex subunit lst8
Q9NW38	*FANCL*	E3 ubiquitin-protein ligase FANCL
Q42891	*GLX1*	Lactoylglutathione lyase
Q6PF93	*Pik3c3*	Phosphatidylinositol 3-kinase catalytic subunit type 3
Q6AZN6	*pik3c3*	Phosphatidylinositol 3-kinase catalytic subunit type 3
Q9BUN5	*CCDC28B*	Coiled-coil domain-containing protein 28B
Q502L2	*pgam5*	Serine/threonine-protein phosphatase PGAM5, mitochondrial
Q5VIR6	*VPS53*	Vacuolar protein sorting-associated protein 53 homolog
Q5E9K3	*PNPO*	Pyridoxine-5'-phosphate oxidase
Q9D6J6	*Ndufv2*	NADH dehydrogenase [ubiquinone] flavoprotein 2, mitochondrial
Q2HJF4	*PBLD*	Phenazine biosynthesis-like domain-containing protein
Q9R216	*Fzd9*	Frizzled-9
Q02645	*hts*	Protein hu-li tai shao
Q5R9E1	*HMGCL*	Hydroxymethylglutaryl-CoA lyase, mitochondrial
P35915	*HMGCL*	Hydroxymethylglutaryl-CoA lyase, mitochondrial

Q4R3I0	*MTX1*	Metaxin-1
Q29RU9	*PIPOX*	Peroxisomal sarcosine oxidase
Q6NWF4	*vps25*	Vacuolar protein-sorting-associated protein 25
Q3UGP8	*Alg10b*	Putative Dol-P-Glc:Glc(2)Man(9)GlcNAc(2)-PP-Dol alpha-1,2-glucosyltransferase
Q9UJM8	*HAO1*	Hydroxyacid oxidase 1
Q6XPS3	*TPTE2*	Phosphatidylinositol 3,4,5-trisphosphate 3-phosphatase TPTE2
Q8CHC4	*Synj1*	Synaptojanin-1
Q66KD0	*btbd17*	BTB/POZ domain-containing protein 17
Q9QZP2	*Prdm4*	PR domain zinc finger protein 4
Q8BFR5	*Tufm*	Elongation factor Tu, mitochondrial
Q8CJ11	*Gpr64*	G-protein coupled receptor 64
P59015	*vps18*	Vacuolar protein sorting-associated protein 18 homolog
O61585	*KATNB1*	Katanin p80 WD40 repeat-containing subunit B1
Q2TBL6	*TALDO1*	Transaldolase
Q5EAN7	*Terf2ip*	Telomeric repeat-binding factor 2-interacting protein 1
A7RRJ0	*FEN1*	Flap endonuclease 1
O54749	*Cyp2j5*	Cytochrome P450 2J5
Q6DIZ0	*haao*	3-hydroxyanthranilate 3,4-dioxygenase
O73787	*tubgcp3*	Gamma-tubulin complex component 3 homolog
Q6P6S9	*Entpd5*	Ectonucleoside triphosphate diphosphohydrolase 5
O70422	*Gtf2h4*	General transcription factor IIH subunit 4
Q6GLM9	*cops5*	COP9 signalosome complex subunit 5
Q8BH74	*Nup107*	Nuclear pore complex protein Nup107
Q28J24	*taf8*	Transcription initiation factor TFIID subunit 8
P28039	*AOAH*	Acyloxyacyl hydrolase
P35290	*Rab24*	Ras-related protein Rab-24

Q503L9	*nxn*	Nucleoredoxin
P11915	*Scp2*	Non-specific lipid-transfer protein
Q16401	*PSMD5*	26S proteasome non-ATPase regulatory subunit 5
P57080	*Usp25*	Ubiquitin carboxyl-terminal hydrolase 25
A2T737	*EHF*	ETS homologous factor
Q9D0W5	*Ppil1*	Peptidyl-prolyl cis-trans isomerase-like 1
Q99JW2	*Acy1*	Aminoacylase-1
P53602	*MVD*	Diphosphomevalonate decarboxylase
Q8N6S4	*ANKRD13C*	Ankyrin repeat domain-containing protein 13C
Q6PD74	*AAGAB*	Alpha- and gamma-adaptin-binding protein p34
P15206		G2/mitotic-specific cyclin-B
Q28IB1	*nudcd1*	NudC domain-containing protein 1
Q96RS6	*NUDCD1*	NudC domain-containing protein 1
P49739	*mmcm3*	Maternal DNA replication licensing factor mcm3
O00233	*PSMD9*	26S proteasome non-ATPase regulatory subunit 9
Q1T7B7	*CENPP*	Centromere protein P
Q9D273	*Mmab*	Cob(I)yrinic acid a,c-diamide adenosyltransferase, mitochondrial
Q0VBZ0	*CSK*	Tyrosine-protein kinase CSK
Q5RDS0	*PNPLA6*	Neuropathy target esterase
B4JLX2	*sws*	Neuropathy target esterase sws
A0MTA1	*apex1*	DNA-(apurinic or apyrimidinic site) lyase
Q6PBE2	*bcas2*	Pre-mRNA-splicing factor SPF27
Q5XM24	*aprA*	Autocrine proliferation repressor protein A
Q3SXM5	*HSDL1*	Inactive hydroxysteroid dehydrogenase-like protein 1
Q6P1S2	*C3orf33*	Protein C3orf33
P0CB79	*NDUFA2*	NADH dehydrogenase [ubiquinone] 1 alpha subcomplex subunit 2

Q3SZV6	*TIMM21*	Mitochondrial import inner membrane translocase subunit Tim21
Q9EQN5	*Ighmbp2*	DNA-binding protein SMUBP-2
Q673U1	*Hs3st2*	Heparan sulfate glucosamine 3-O-sulfotransferase 2
Q6AI39	*GLTSCR1L*	GLTSCR1-like protein
Q29448	*HMGCL*	Hydroxymethylglutaryl-CoA lyase, mitochondrial
Q8C9H6	*Strip2*	Striatin-interacting proteins 2
Q6DRB1	*gle1*	Nucleoporin GLE1
Q5RC70	*DNAJC11*	DnaJ homolog subfamily C member 11
Q8IY57	*YAF2*	YY1-associated factor 2
Q90X38	*gpkow*	G patch domain and KOW motifs-containing protein
Q9YGN6	*smug1*	Single-strand selective monofunctional uracil DNA glycosylase
Q8IWL3	*HSCB*	Iron-sulfur cluster co-chaperone protein HscB, mitochondrial
Q32PH2	*TMEM143*	Transmembrane protein 143
O14802	*POLR3A*	DNA-directed RNA polymerase III subunit RPC1
Q96GX1	*TCTN2*	Tectonic-2
P50532	*smc4*	Structural maintenance of chromosomes protein 4
Q8N6F8	*WBSCR27*	Williams-Beuren syndrome chromosomal region 27 protein
A0LMC1	*phnX*	Phosphonoacetaldehyde hydrolase
O88907	*Pias1*	E3 SUMO-protein ligase PIAS1
P26882	*PPID*	Peptidyl-prolyl cis-trans isomerase D
Q9UJ83	*HACL1*	2-hydroxyacyl-CoA lyase 1
Q6DHJ1	*tyw3*	tRNA wybutosine-synthesizing protein 3 homolog
Q5BJX1	*Mrpl41*	39S ribosomal protein L41, mitochondrial
Q2NKS2	*COX16*	Cytochrome c oxidase assembly protein COX16 homolog, mitochondrial
Q07553	*Gyc32E*	Guanylate cyclase 32E
Q24214	*CanB2*	Calcineurin subunit B type 2

O08759	*Ube3a*	Ubiquitin-protein ligase E3A
O43808	*SLC25A17*	Peroxisomal membrane protein PMP34
Q7ZYD5	*slc25a25*	Calcium-binding mitochondrial carrier protein SCaMC-2
Q99J08	*Sec14l2*	SEC14-like protein 2
P53007	*SLC25A1*	Tricarboxylate transport protein, mitochondrial
A4IG72	*tgfbrap1*	Transforming growth factor-beta receptor-associated protein 1 homolog
Q66GV0	*mpv17*	Protein Mpv17
Q14320	*FAM50A*	Protein FAM50A
Q8BK06	*Fbxo9*	F-box only protein 9
Q8BGZ4	*Cdc23*	Cell division cycle protein 23 homolog
F6Y9J3	*fbxo9*	F-box only protein 9
P49815	*TSC2*	Tuberin
B0S8I0	*gtpbp8*	GTP-binding protein 8
O08836	*Igbp1*	Immunoglobulin-binding protein 1
O95848	*NUDT14*	Uridine diphosphate glucose pyrophosphatase
Q9UHG3	*PCYOX1*	Prenylcysteine oxidase 1
Q95KC9	*PCYOX1*	Prenylcysteine oxidase
D3ZZL9	*Gcc2*	GRIP and coiled-coil domain-containing protein 2
Q6P6B7	*ANKRD16*	Ankyrin repeat domain-containing protein 16
P58742	*Aaas*	Aladin
P82861	*fdxr*	NADPH:adrenodoxin oxidoreductase, mitochondrial
Q6ZT98	*TTLL7*	Tubulin polyglutamylase TTLL7
Q6NRL2	*snx33*	Sorting nexin-33
Q4V8X4	*acbd6*	Acyl-CoA-binding domain-containing protein 6
Q32KM1	*TUBG2*	Tubulin gamma-2 chain
Q13472	*TOP3A*	DNA topoisomerase 3-alpha

Q6DCF2	*mms19*	MMS19 nucleotide excision repair protein homolog
Q9H1I8	*ASCC2*	Activating signal cointegrator 1 complex subunit 2
Q9NQT4	*EXOSC5*	Exosome complex component RRP46
B3DJT0	*sart3*	Squamous cell carcinoma antigen recognized by T-cells 3
Q5REG1	*SART3*	Squamous cell carcinoma antigen recognized by T-cells 3
O75771	*RAD51D*	DNA repair protein RAD51 homolog 4
O75575	*CRCP*	DNA-directed RNA polymerase III subunit RPC9
Q5ZIH0	*CPSF3L*	Integrator complex subunit 11
Q1RMS6	*INTS7*	Integrator complex subunit 7
Q5U3Y0	*Klhdc10*	Kelch domain-containing protein 10
Q7ZWY2	*pih1d1*	PIH1 domain-containing protein 1
Q66KX4	*med29*	Mediator of RNA polymerase II transcription subunit 29
Q8VCX6	*Kptn*	Kaptin
Q8N3U4	*STAG2*	Cohesin subunit SA-2
Q28CZ9	*cyb5r4*	Cytochrome b5 reductase 4
Q96GC5	*MRPL48*	39S ribosomal protein L48, mitochondrial
Q9QYZ8	*Dkk2*	Dickkopf-related protein 2
Q9JJT9	*Phax*	Phosphorylated adapter RNA export protein
Q96LU5	*IMMP1L*	Mitochondrial inner membrane protease subunit 1
Q9GKZ4	*TRAM1*	Translocating chain-associated membrane protein 1
Q96LD8	*SENP8*	Sentrin-specific protease 8
Q9BUB7	*TMEM70*	Transmembrane protein 70, mitochondrial
Q5CZL8	*cybrd1*	Cytochrome b reductase 1
Q9BM98	*janA*	Sex-regulated protein janus-A
Q9NHD5	*san*	Probable N-acetyltransferase san
Q7RTY0	*SLC16A13*	Monocarboxylate transporter 13

Q64244	*Cd38*	ADP-ribosyl cyclase/cyclic ADP-ribose hydrolase 1
A1A4Q2	*PRORSD1*	Prolyl-tRNA synthetase associated domain-containing protein 1
Q53H12	*AGK*	Acylglycerol kinase, mitochondrial
P05165	*PCCA*	Propionyl-CoA carboxylase alpha chain, mitochondrial
P23475	*Xrcc6*	X-ray repair cross-complementing protein 6
Q7ZTL7	*bcs1l*	Mitochondrial chaperone BCS1
Q5JTJ3	*COA6*	Cytochrome c oxidase assembly factor 6 homolog
Q5ZLD7	*VPS53*	Vacuolar protein sorting-associated protein 53 homolog
Q12769	*NUP160*	Nuclear pore complex protein Nup160
Q4R6F3	*NGLY1*	Peptide-N(4)-(N-acetyl-beta-glucosaminyl)asparagine amidase
P14735	*IDE*	Insulin-degrading enzyme
Q925E0	*Sntg2*	Gamma-2-syntrophin
Q4VBS4	*metap1d*	Methionine aminopeptidase 1D, mitochondrial

Table S13. Swiss-port annotation of genes in profile B.

Swiss-prot ID	Gene Name	Description
Q2LC84	*Numb*	Protein numb homolog
P51798	*CLCN7*	H(+)/Cl(-) exchange transporter 7
O96530		Hyalin (Fragment)
O18638	*oaf*	Out at first protein
Q9H2G9	*BLZF1*	Golgin-45
Q9JMD3	*Stard10*	PCTP-like protein
Q9Y5S8	*NOX1*	NADPH oxidase 1
Q967D7	*tutl*	Protein turtle
Q96N64	*PWWP2A*	PWWP domain-containing protein 2A
Q2T9T9	*FBXW9*	F-box/WD repeat-containing protein 9
Q2T9T9	*FBXW9*	F-box/WD repeat-containing protein 9
Q21281	*mup-4*	Transmembrane matrix receptor MUP-4
P24523	*GADD45A*	Growth arrest and DNA damage-inducible protein GADD45 alpha
O15530	*PDPK1*	3-phosphoinositide-dependent protein kinase 1
A6NFN3	*RBFOX3*	RNA binding protein fox-1 homolog 3
Q2KJ63	*KLKB1*	Plasma kallikrein
Q80U62	*Kiaa0226*	Run domain Beclin-1 interacting and cysteine-rich containing protein
Q9D7S9	*Chmp5*	Charged multivesicular body protein 5
Q60773	*Cdkn2d*	Cyclin-dependent kinase 4 inhibitor D
O14964	*HGS*	Hepatocyte growth factor-regulated tyrosine kinase substrate
Q6NT16	*SLC18B1*	MFS-type transporter SLC18B1
Q99PI5	*Lpin2*	Phosphatidate phosphatase LPIN2
Q6PBT5	*pyroxd1*	Pyridine nucleotide-disulfide oxidoreductase domain-containing protein 1
Q6DDL7	*unc93a*	Protein unc-93 homolog A

P35289	*Rab15*	Ras-related protein Rab-15
Q04656	*ATP7A*	Copper-transporting ATPase 1
Q75J93	*cpras1*	Circularly permutated Ras protein 1
P04634	*Lipf*	Gastric triacylglycerol lipase
Q8BYH7	*Tbc1d17*	TBC1 domain family member 17
A8MQ27	*NEURL1B*	E3 ubiquitin-protein ligase NEURL1B
O76050	*NEURL1*	E3 ubiquitin-protein ligase NEURL1
O75122	*CLASP2*	CLIP-associating protein 2
Q5RFR6	*KIAA0319L*	Dyslexia-associated protein KIAA0319-like protein
Q9V7Y2	*Sply*	Sphingosine-1-phosphate lyase
Q5XIS7	*Ubap1*	Ubiquitin-associated protein 1
Q9UL36	*ZNF236*	Zinc finger protein 236
Q96N67	*DOCK7*	Dedicator of cytokinesis protein 7
Q8NHE4	*ATP6V0E2*	V-type proton ATPase subunit e 2
Q8CBY3	*Leng8*	Leukocyte receptor cluster member 8 homolog
P13686	*ACP5*	Tartrate-resistant acid phosphatase type 5
Q9Z1J8	*Sec14l3*	SEC14-like protein 3
O42563	*cyp3a27*	Cytochrome P450 3A27
Q6T486	*rbrA*	Probable E3 ubiquitin-protein ligase rbrA
Q9UA35	*SLC28A3*	Solute carrier family 28 member 3
Q924T7	*Rnf31*	E3 ubiquitin-protein ligase RNF31
Q9UBS8	*RNF14*	E3 ubiquitin-protein ligase RNF14
Q9CYU6	*Dph7*	Diphthine methyltransferase
Q3U3V8	*Xrra1*	X-ray radiation resistance-associated protein 1
Q75J93	*cpras1*	Circularly permutated Ras protein 1
P78312	*FAM193A*	Protein FAM193A

Q9H2I8	*C10orf11*	Leucine-rich repeat-containing protein C10orf11
Q9H2I8	*C10orf11*	Leucine-rich repeat-containing protein C10orf11
Q13772	*NCOA4*	Nuclear receptor coactivator 4
Q1LV17	*irf2bp1*	Interferon regulatory factor 2-binding protein 1
Q96QT6	*PHF12*	PHD finger protein 12
Q9D600	*Gins2*	DNA replication complex GINS protein PSF2
Q9IBG7	*kcp*	Kielin/chordin-like protein
P35624	*TIMP1*	Metalloproteinase inhibitor 1
Q9JLV1	*Bag3*	BAG family molecular chaperone regulator 3
P0CT32	*eef1a2*	Elongation factor 1-alpha
Q9H0E9	*BRD8*	Bromodomain-containing protein 8
Q8BG21	*Cacfd1*	Calcium channel flower homolog
Q3B7L5	*FLCN*	Folliculin
Q3B7L5	*FLCN*	Folliculin
Q8N511	*TMEM199*	Transmembrane protein 199
A7YWP4	*HAL*	Histidine ammonia-lyase
Q16739	*UGCG*	Ceramide glucosyltransferase
Q8IVE3	*PLEKHH2*	Pleckstrin homology domain-containing family H member 2
O15027	*SEC16A*	Protein transport protein Sec16A
Q3T904	*ATG9A*	Autophagy-related protein 9A
Q8VEK0	*Tmem30a*	Cell cycle control protein 50A
Q9WU60	*Atrn*	Attractin
Q6A051	*Atrnl1*	Attractin-like protein 1
P60924	*Dele*	Death ligand signal enhancer
A8K2U0	*A2ML1*	Alpha-2-macroglobulin-like protein 1
Q6P5L8	*hsdl2*	Hydroxysteroid dehydrogenase-like protein 2

Q9UBI4	*STOML1*	Stomatin-like protein 1
Q3T178	*VPS28*	Vacuolar protein sorting-associated protein 28 homolog
O54981	*STIP1*	Stress-induced-phosphoprotein 1
Q8BH75	*Rnf41*	E3 ubiquitin-protein ligase NRDP1
Q9JI90	*Rnf14*	E3 ubiquitin-protein ligase RNF14
Q16543	*CDC37*	Hsp90 co-chaperone Cdc37
Q96P53	*WDFY2*	WD repeat and FYVE domain-containing protein 2
Q9CY45	*N6amt2*	Protein-lysine N-methyltransferase N6amt2
Q9NX94	*WBP1L*	WW domain binding protein 1-like
Q66JZ4	*TCAIM*	T-cell activation inhibitor, mitochondrial
Q9D9G3	*Chic2*	Cysteine-rich hydrophobic domain-containing protein 2
Q7Z2Z2	*EFTUD1*	Elongation factor Tu GTP-binding domain-containing protein 1
Q8C0D5	*Eftud1*	Elongation factor Tu GTP-binding domain-containing protein 1
Q9BY84	*DUSP16*	Dual specificity protein phosphatase 16
Q28178	*THBS1*	Thrombospondin-1
Q28178	*THBS1*	Thrombospondin-1
Q0P4S0	*zfyve28*	Lateral signaling target protein 2 homolog
Q80U96	*Xpo1*	Exportin-1
O95436	*SLC34A2*	Sodium-dependent phosphate transport protein 2B
Q5ZJX5	*TLDC1*	TLD domain-containing protein 1
Q6PGU2	*maf1*	Repressor of RNA polymerase III transcription MAF1 homolog
Q4QQW8	*Plbd2*	Putative phospholipase B-like 2
Q4R4A2	*ZUFSP*	Zinc finger with UFM1-specific peptidase domain protein
Q5VT06	*CEP350*	Centrosome-associated protein 350
Q9Z1Z1	*Eif2ak3*	Eukaryotic translation initiation factor 2-alpha kinase 3
Q9Z2B5	*Eif2ak3*	Eukaryotic translation initiation factor 2-alpha kinase 3

Q6F3F9	*Gpr126*	G-protein coupled receptor 126
P62999	*RAC1*	Ras-related C3 botulinum toxin substrate 1
P62999	*RAC1*	Ras-related C3 botulinum toxin substrate 1
P62998	*RAC1*	Ras-related C3 botulinum toxin substrate 1
P62999	*RAC1*	Ras-related C3 botulinum toxin substrate 1
Q80U96	*Xpo1*	Exportin-1
Q7L945	*ZNF627*	Zinc finger protein 627
Q32NT4	*lrrc58*	Leucine-rich repeat-containing protein 58
Q86TD4	*SRL*	Sarcalumenin
Q499B3	*kansl3*	KAT8 regulatory NSL complex subunit 3
Q9XSW3	*MBL2*	Mannose-binding protein C
Q96RV3	*PCNX*	Pecanex-like protein 1
P48820	*RANBP2*	E3 SUMO-protein ligase RanBP2 (Fragment)
Q5F480	*ITPK1*	Inositol-tetrakisphosphate 1-kinase
Q5F480	*ITPK1*	Inositol-tetrakisphosphate 1-kinase
P01105	*GAG*	p135Gag-Myb-Ets-transforming protein (Fragment)
Q8IZJ1	*UNC5B*	Netrin receptor UNC5B
A6BM72	*MEGF11*	Multiple epidermal growth factor-like domains protein 11
Q29466	*ATP6V0A1*	V-type proton ATPase 116 kDa subunit a isoform 1
Q9Z1G4	*Atp6v0a1*	V-type proton ATPase 116 kDa subunit a isoform 1
Q03001	*DST*	Dystonin
Q8CG19	*Ltbp1*	Latent-transforming growth factor beta-binding protein 1
Q6DF27	*chmp1b*	Charged multivesicular body protein 1b
P14921	*ETS1*	Protein C-ets-1
P51784	*USP11*	Ubiquitin carboxyl-terminal hydrolase 11
P35125	*USP6*	Ubiquitin carboxyl-terminal hydrolase 6

Q5HYM0	*ZC3H12B*	Probable ribonuclease ZC3H12B
O14976	*GAK*	Cyclin-G-associated kinase
Q6VUC1	*DPH3*	DPH3 homolog
Q9UBS8	*RNF14*	E3 ubiquitin-protein ligase RNF14
Q6NX45	*ZNF774*	Zinc finger protein 774
Q7TPM9	*Mtmr10*	Myotubularin-related protein 10
Q14781	*CBX2*	Chromobox protein homolog 2
Q9HBF4	*ZFYVE1*	Zinc finger FYVE domain-containing protein 1
Q6PFJ9	*arih1*	E3 ubiquitin-protein ligase arih1
Q9UBS8	*RNF14*	E3 ubiquitin-protein ligase RNF14
P48067	*SLC6A9*	Sodium- and chloride-dependent glycine transporter 1
P37892	*cpe*	Carboxypeptidase E
A6H7I3	*ANGEL2*	Protein angel homolog 2
Q9EQT3	*Rhou*	Rho-related GTP-binding protein RhoU
Q8WXF0	*SRSF12*	Serine/arginine-rich splicing factor 12
Q8BQR4	*Kansl2*	KAT8 regulatory NSL complex subunit 2
Q24306	*Diap1*	Death-associated inhibitor of apoptosis 1
P23927	*Cryab*	Alpha-crystallin B chain
Q91X58	*Zfand2b*	AN1-type zinc finger protein 2B
Q91V37	*Atp6v0b*	V-type proton ATPase 21 kDa proteolipid subunit
Q13905	*RAPGEF1*	Rap guanine nucleotide exchange factor 1
Q6F3J0	*NFKB1*	Nuclear factor NF-kappa-B p105 subunit
Q27433	*mec-2*	Mechanosensory protein 2
Q27433	*mec-2*	Mechanosensory protein 2
Q8QZY6	*Tspan14*	Tetraspanin-14
O09015	*Mxi1*	Max-interacting protein 1

O09015	*Mxi1*	Max-interacting protein 1
P50540	*Mxi1*	Max-interacting protein 1
O54715	*Atp6ap1*	V-type proton ATPase subunit S1
Q9BRS2	*RIOK1*	Serine/threonine-protein kinase RIO1
Q9Y2H1	*STK38L*	Serine/threonine-protein kinase 38-like
Q5WR10	*DDX39B*	Spliceosome RNA helicase DDX39B
P36993	*Ppm1b*	Protein phosphatase 1B
Q9VUL9	*FucTA*	Glycoprotein 3-alpha-L-fucosyltransferase A
O97758	*TJP1*	Tight junction protein ZO-1
A0JM12	*megf10*	Multiple epidermal growth factor-like domains protein 10
Q8R519	*Acmsd*	2-amino-3-carboxymuconate-6-semialdehyde decarboxylase
P50428	*Arsa*	Arylsulfatase A
Q5ZJY3	*GARNL3*	GTPase-activating Rap/Ran-GAP domain-like protein 3
Q80T91	*Megf11*	Multiple epidermal growth factor-like domains protein 11
Q9JJC9	*Herpud2*	Homocysteine-responsive endoplasmic reticulum-resident ubiquitin-like domain member 2 protein
Q08170	*SRSF4*	Serine/arginine-rich splicing factor 4
A5D6U8	*papl*	Iron/zinc purple acid phosphatase-like protein
Q8BUM9	*Usp43*	Ubiquitin carboxyl-terminal hydrolase 43
O95433	*AHSA1*	Activator of 90 kDa heat shock protein ATPase homolog 1
Q80SU7	*Gvin1*	Interferon-induced very large GTPase 1
C6KFA3	*gpr126*	G-protein coupled receptor 126
F1N9Y5	*SYK*	Tyrosine-protein kinase SYK
Q99946	*PRRT1*	Proline-rich transmembrane protein 1
O75074	*LRP3*	Low-density lipoprotein receptor-related protein 3
Q969T9	*WBP2*	WW domain-binding protein 2
Q9HBG4	*ATP6V0A4*	V-type proton ATPase 116 kDa subunit a isoform 4

O35598	*Adam10*	Disintegrin and metalloproteinase domain-containing protein 10
P37234	*pparg*	Peroxisome proliferator-activated receptor gamma
Q7ZVV1	*ercc3*	TFIIH basal transcription factor complex helicase XPB subunit
Q2KJ36	*DUSP6*	Dual specificity protein phosphatase 6
Q90YY5	*med26*	Mediator of RNA polymerase II transcription subunit 26
Q9WU78	*Pdcd6ip*	Programmed cell death 6-interacting protein
Q92783	*STAM*	Signal transducing adapter molecule 1
O76536		Hyalin (Fragment)
O76536		Hyalin (Fragment)
B6RSP1	*plekha7*	Pleckstrin homology domain-containing family A member 7
Q9D8B3	*Chmp4b*	Charged multivesicular body protein 4b
A1XQU5	*RPL27*	60S ribosomal protein L27
Q32P51	*HNRNPA1L2*	Heterogeneous nuclear ribonucleoprotein A1-like 2
Q8AY29	*ugcg-a*	Ceramide glucosyltransferase-A
Q9H3S7	*PTPN23*	Tyrosine-protein phosphatase non-receptor type 23
Q969S3	*ZNF622*	Zinc finger protein 622
Q90Y35	*ZNF622*	Zinc finger protein 622
Q9NJ15	*PC6*	Proprotein convertase subtilisin/kexin type 5
Q9NJ15	*PC6*	Proprotein convertase subtilisin/kexin type 5
Q66KU2	*sec61g*	Protein transport protein Sec61 subunit gamma
Q5PQT2	*Mtmr3*	Myotubularin-related protein 3
Q3SZ68	*LAMTOR5*	Ragulator complex protein LAMTOR5
Q27433	*mec-2*	Mechanosensory protein 2
Q9JI90	*Rnf14*	E3 ubiquitin-protein ligase RNF14
P49137	*MAPKAPK2*	MAP kinase-activated protein kinase 2
Q9VGY6	*Skeletor*	Protein Skeletor, isoforms B/C

O95786	*DDX58*	Probable ATP-dependent RNA helicase DDX58
P84246	*H3F3A*	Histone H3.3
A5A6J2	*DDX5*	Probable ATP-dependent RNA helicase DDX5
Q9UJF2	*RASAL2*	Ras GTPase-activating protein nGAP
A9JTG5	*rbck1*	RanBP-type and C3HC4-type zinc finger-containing protein 1
A4IHK6	*slc43a2*	Large neutral amino acids transporter small subunit 4
Q8N370	*SLC43A2*	Large neutral amino acids transporter small subunit 4
Q6J6J0	*BRCA1*	Breast cancer type 1 susceptibility protein homolog
Q4R6M5	*DDX5*	Probable ATP-dependent RNA helicase DDX5
Q9Z255	*Ube2a*	Ubiquitin-conjugating enzyme E2 A
Q9CQR2	*Rps21*	40S ribosomal protein S21
Q75WF2		Plancitoxin-1
Q75J93	*cpras1*	Circularly permutated Ras protein 1
Q75J93	*cpras1*	Circularly permutated Ras protein 1
Q6P2D8	*XRRA1*	X-ray radiation resistance-associated protein 1
P08962	*CD63*	CD63 antigen
C0HJP3	*COL1A1*	Collagen alpha-1(I) chain (Fragments)
Q9UDY8	*MALT1*	Mucosa-associated lymphoid tissue lymphoma translocation protein 1
Q80XI3	*Eif4g3*	Eukaryotic translation initiation factor 4 gamma 3
Q9H4G4	*GLIPR2*	Golgi-associated plant pathogenesis-related protein 1
Q24322	*Sema-1a*	Semaphorin-1A
Q4R596	*AHCY*	Adenosylhomocysteinase
Q5R416	*CTNNA2*	Catenin alpha-2
O60140	*SPBC18H10.09*	Uncharacterized protein C18H10.09
Q8VEM8	*Slc25a3*	Phosphate carrier protein, mitochondrial
Q6TEM9	*mylipa*	E3 ubiquitin-protein ligase MYLIP-A

Q63429	*Ubc*	Polyubiquitin-C
Q63429	*Ubc*	Polyubiquitin-C
Q8MKD1	*UBB*	Polyubiquitin-B
Q15025	*TNIP1*	TNFAIP3-interacting protein 1
Q63429	*Ubc*	Polyubiquitin-C
Q9DCT5	*Sdf2*	Stromal cell-derived factor 2
P97434	*Mprip*	Myosin phosphatase Rho-interacting protein
O93477	*ahcy-b*	Adenosylhomocysteinase B
O93477	*ahcy-b*	Adenosylhomocysteinase B
O54910	*Nfkbie*	NF-kappa-B inhibitor epsilon
O60637	*TSPAN3*	Tetraspanin-3
Q07352	*ZFP36L1*	Zinc finger protein 36, C3H1 type-like 1
P0CG60	*UBB*	Polyubiquitin-B
Q8MKD1	*UBB*	Polyubiquitin-B
Q3T0B7	*RPS27L*	40S ribosomal protein S27-like
Q32L83	*BRI3*	Brain protein I3
Q00004	*SRP68*	Signal recognition particle subunit SRP68
Q8MKD1	*UBB*	Polyubiquitin-B
P51810	*GPR143*	G-protein coupled receptor 143
Q684M2	*ATG4D*	Cysteine protease ATG4D
Q56K03	*RPL27A*	60S ribosomal protein L27a
P85838		Ferritin, heavy subunit
P0CG68	*UBC*	Polyubiquitin-C
Q9GP16	*RpL31*	60S ribosomal protein L31
O16110	*AAEL000291*	V-type proton ATPase 16 kDa proteolipid subunit
Q8CGS4	*Chmp3*	Charged multivesicular body protein 3

Q60520	*Sin3a*	Paired amphipathic helix protein Sin3a
P51968		Heterogeneous nuclear ribonucleoprotein A3 homolog 1
Q502F0	*creb3l3a*	Cyclic AMP-responsive element-binding protein 3-like protein 3-A
Q8CGY8	*Ogt*	UDP-N-acetylglucosamine--peptide N-acetylglucosaminyltransferase 110 kDa subunit
A6BM72	*MEGF11*	Multiple epidermal growth factor-like domains protein 11
Q94738	*HSP110*	97 kDa heat shock protein
Q920R6	*Atp6v0a4*	V-type proton ATPase 116 kDa subunit a isoform 4
Q03141	*Mark3*	MAP/microtubule affinity-regulating kinase 3
Q9BYP7	*WNK3*	Serine/threonine-protein kinase WNK3
P21531	*Rpl3*	60S ribosomal protein L3
Q3MHV9	*SERINC1*	Serine incorporator 1
P29691	*eef-2*	Elongation factor 2
P08775	*Polr2a*	DNA-directed RNA polymerase II subunit RPB1
O02755	*CEBPB*	CCAAT/enhancer-binding protein beta
A4D0T7	*LINC00998*	Putative transmembrane protein LINC00998
Q90YH9	*TES*	Testin
Q9NDJ2	*dom*	Helicase domino
P17507	*eef1ao*	Elongation factor 1-alpha, oocyte form
P15870		Histone H1-delta
Q49SH1	*slc24a5*	Sodium/potassium/calcium exchanger 5
Q8K4K3	*Trib2*	Tribbles homolog 2
Q90YT7	*rpl31*	60S ribosomal protein L31
P50615	*Btg3*	Protein BTG3
E1BD59	*TRIM56*	E3 ubiquitin-protein ligase TRIM56
Q9HBI1	*PARVB*	Beta-parvin
O76757	*Ahcy13*	Adenosylhomocysteinase

P13060	*EF2*	Elongation factor 2
Q5M8Z0	*bhmt*	Betaine--homocysteine S-methyltransferase 1
Q90694	*CDC42*	Cell division control protein 42 homolog
Q6RUV5	*Rac1*	Ras-related C3 botulinum toxin substrate 1
P98156	*Vldlr*	Very low-density lipoprotein receptor
P27604	*ahcy-1*	Adenosylhomocysteinase
P42577		Soma ferritin
Q66KU4	*rpl36*	60S ribosomal protein L36
P42263	*GRIA3*	Glutamate receptor 3
Q3T0X6	*RPS16*	40S ribosomal protein S16
Q63429	*Ubc*	Polyubiquitin-C
P42577		Soma ferritin
Q9BZ67	*FRMD8*	FERM domain-containing protein 8
Q71U00	*skp1*	S-phase kinase-associated protein 1
P13060	*EF2*	Elongation factor 2
P36241	*RpL19*	60S ribosomal protein L19
A2BFP5	*slc12a9*	Solute carrier family 12 member 9
Q13002	*GRIK2*	Glutamate receptor ionotropic, kainate 2
A5A6K9	*HSP90AA1*	Heat shock protein HSP 90-alpha
Q969Q5	*RAB24*	Ras-related protein Rab-24
O15943	*CadN*	Neural-cadherin
Q9DFQ7	*rpl24*	60S ribosomal protein L24
Q8IX03	*WWC1*	Protein KIBRA
Q8M893	*MT-ND1*	NADH-ubiquinone oxidoreductase chain 1
Q61466	*Smarcd1*	SWI/SNF-related matrix-associated actin-dependent regulator of chromatin subfamily D member 1
Q6DIB5	*Megf10*	Multiple epidermal growth factor-like domains protein 10

Q99M80	*Ptprt*	Receptor-type tyrosine-protein phosphatase T
P0CT31	*eef1a1*	Elongation factor 1-alpha
Q9NB33	*RpL44*	60S ribosomal protein L44
A5A6P2	*ASAH1*	Acid ceramidase
Q9VGY6	*Skeletor*	Protein Skeletor, isoforms B/C
Q4LAY1	*RpS29*	40S ribosomal protein S29
Q29451	*MAN2B1*	Lysosomal alpha-mannosidase
Q6P431	*mknk2*	MAP kinase-interacting serine/threonine-protein kinase 2
Q52KE7	*Ccnl1*	Cyclin-L1
Q7ZVX0	*ccnl1*	Cyclin-L1
Q9BXK1	*KLF16*	Krueppel-like factor 16
Q96L91	*EP400*	E1A-binding protein p400
Q8CFE5	*Btbd7*	BTB/POZ domain-containing protein 7
P24928	*POLR2A*	DNA-directed RNA polymerase II subunit RPB1
Q9WTP3	*Spdef*	SAM pointed domain-containing Ets transcription factor
Q9C0J8	*WDR33*	pre-mRNA 3' end processing protein WDR33
C0HJP3	*COL1A1*	Collagen alpha-1(I) chain (Fragments)
P23403	*rps20*	40S ribosomal protein S20
Q2TAD9	*cyhr1-b*	Cysteine and histidine-rich protein 1-B
P70066	*rps15*	40S ribosomal protein S15
Q9JI90	*Rnf14*	E3 ubiquitin-protein ligase RNF14
Q9BU79	*TMEM243*	Transmembrane protein 243
F1MKX4	*PSME4*	Proteasome activator complex subunit 4
O35119	*Trpc4*	Short transient receptor potential channel 4
Q00449	*Mdr49*	Multidrug resistance protein homolog 49
Q15386	*UBE3C*	Ubiquitin-protein ligase E3C

Q6DJ95	*cars2*	Probable cysteine--tRNA ligase, mitochondrial
Q9DBT9	*Dmgdh*	Dimethylglycine dehydrogenase, mitochondrial
Q9HC52	*CBX8*	Chromobox protein homolog 8
Q80TR4	*Slit1*	Slit homolog 1 protein
F7H9X2	*TRIM2*	Tripartite motif-containing protein 2
Q14094	*CCNI*	Cyclin-I
Q60795	*Nfe2l2*	Nuclear factor erythroid 2-related factor 2
Q9V576	*wun*	Putative phosphatidate phosphatase
Q5NVI9	*DNAJA1*	DnaJ homolog subfamily A member 1
Q80U16	*Fam65b*	Protein FAM65B
Q10743	*Adam10*	Disintegrin and metalloproteinase domain-containing protein 10 (Fragment)
O77656	*MMP13*	Collagenase 3
O77656	*MMP13*	Collagenase 3
A0JMD2	*zfyve28*	Lateral signaling target protein 2 homolog
Q6P5S7	*RNASEK*	Ribonuclease kappa
Q91ZW3	*Smarca5*	SWI/SNF-related matrix-associated actin-dependent regulator of chromatin subfamily A member 5
Q61139	*Pcsk7*	Proprotein convertase subtilisin/kexin type 7
Q5ZJ54	*CCT6*	T-complex protein 1 subunit zeta
Q5F489	*TAF3*	Transcription initiation factor TFIID subunit 3
Q96PE6	*ZIM3*	Zinc finger imprinted 3
Q920R6	*Atp6v0a4*	V-type proton ATPase 116 kDa subunit a isoform 4
Q6QLW4		Cytochrome c
Q7Z7E8	*UBE2Q1*	Ubiquitin-conjugating enzyme E2 Q1
O00560	*SDCBP*	Syntenin-1
Q9BQ16	*SPOCK3*	Testican-3
P79145	*CREM*	cAMP-responsive element modulator

Q27433	*mec-2*	Mechanosensory protein 2
Q8JGR4	*rpl24*	60S ribosomal protein L24
Q3T0D7	*SAR1A*	GTP-binding protein SAR1a
Q16825	*PTPN21*	Tyrosine-protein phosphatase non-receptor type 21
Q963B6	*RpL10A*	60S ribosomal protein L10a
P70459	*Erf*	ETS domain-containing transcription factor ERF
Q97IC2	*CA_C1728*	Probable serine/threonine-protein kinase CA_C1728
Q4R5H7	*DAZAP2*	DAZ-associated protein 2
Q6DHR3	*rasgef1ba*	Ras-GEF domain-containing family member 1B-A
O01727	*RPS6*	40S ribosomal protein S6
Q9NWZ3	*IRAK4*	Interleukin-1 receptor-associated kinase 4
O62683	*TJP3*	Tight junction protein ZO-3
Q8TB96	*ITFG1*	T-cell immunomodulatory protein
Q8K0Q5	*Arhgap18*	Rho GTPase-activating protein 18
O43312	*MTSS1*	Metastasis suppressor protein 1
P49013	*EGF3*	Fibropellin-3
Q62384	*Zpr1*	Zinc finger protein ZPR1
A0JPH4	*scap*	Sterol regulatory element-binding protein cleavage-activating protein
O35657	*Neu1*	Sialidase-1
Q2T9X2	*CCT4*	T-complex protein 1 subunit delta
Q3UDK1	*Trafd1*	TRAF-type zinc finger domain-containing protein 1
O08623	*Sqstm1*	Sequestosome-1
Q14596	*NBR1*	Next to BRCA1 gene 1 protein
Q6T486	*rbrA*	Probable E3 ubiquitin-protein ligase rbrA
Q5EAU3	*tmem55a*	Type II phosphatidylinositol 4,5-bisphosphate 4-phosphatase
Q8CIN9	*Rffl*	E3 ubiquitin-protein ligase rififylin

O60293	*ZFC3H1*	Zinc finger C3H1 domain-containing protein
Q5SWA1	*PPP1R15B*	Protein phosphatase 1 regulatory subunit 15B
Q5E9Z2	*HABP2*	Hyaluronan-binding protein 2
Q8R1A4	*Dock7*	Dedicator of cytokinesis protein 7
Q9Y485	*DMXL1*	DmX-like protein 1
P27465	*PISD*	Phosphatidylserine decarboxylase proenzyme
Q8CDG3	*Vcpip1*	Deubiquitinating protein VCIP135
P36241	*RpL19*	60S ribosomal protein L19
Q9W4J5	*CG3527*	Ribosomal RNA small subunit methyltransferase NEP1
Q96CD0	*FBXL8*	F-box/LRR-repeat protein 8
O76536		Hyalin (Fragment)
P25155	*F10*	Coagulation factor X
Q8MSF5	*CG40127*	Ribonuclease kappa
Q80U95	*Ube3c*	Ubiquitin-protein ligase E3C
Q9XZE5	*pho2a*	Serine/threonine-protein phosphatase 2A catalytic subunit A
Q5VT06	*CEP350*	Centrosome-associated protein 350
A4FUC9	*RHPN2*	Rhophilin-2
Q95XZ6	*laat-1*	Lysosomal amino acid transporter 1
Q96FA3	*PELI1*	E3 ubiquitin-protein ligase pellino homolog 1
Q6IQ85	*yipf6*	Protein YIPF6
Q6XPZ3	*PLA2G15*	Group XV phospholipase A2
Q3UVU3	*Slc30a10*	Zinc transporter 10
Q6IQ55	*TTBK2*	Tau-tubulin kinase 2
Q7M456		Ribonuclease Oy
Q7ZX21	*otud5-a*	OTU domain-containing protein 5-A
Q9Y3P9	*RABGAP1*	Rab GTPase-activating protein 1

Q6BHV8	*RPL39*	60S ribosomal protein L39
Q99NH0	*Ankrd17*	Ankyrin repeat domain-containing protein 17
Q5R7S6	*ZFAND6*	AN1-type zinc finger protein 6
Q66HG8	*Ik*	Protein Red
Q15652	*JMJD1C*	Probable JmjC domain-containing histone demethylation protein 2C
Q5ZIX8	*KDM3A*	Lysine-specific demethylase 3A
Q923S6	*Neurl1*	E3 ubiquitin-protein ligase NEURL1
P27465	*PISD*	Phosphatidylserine decarboxylase proenzyme
P52827	*MRPL12*	39S ribosomal protein L12, mitochondrial
Q8BG09	*Tmem184b*	Transmembrane protein 184B
Q9P2E3	*ZNFX1*	NFX1-type zinc finger-containing protein 1
Q8K363	*Ddx18*	ATP-dependent RNA helicase DDX18
P46109	*CRKL*	Crk-like protein
P46530	*notch1a*	Neurogenic locus notch homolog protein 1
Q8IU60	*DCP2*	m7GpppN-mRNA hydrolase
O76536		Hyalin (Fragment)
Q9HB90	*RRAGC*	Ras-related GTP-binding protein C
O95405	*ZFYVE9*	Zinc finger FYVE domain-containing protein 9
Q32PF3	*PCNP*	PEST proteolytic signal-containing nuclear protein
Q92551	*IP6K1*	Inositol hexakisphosphate kinase 1
P0CG49	*Ubb*	Polyubiquitin-B
Q9P278	*FNIP2*	Folliculin-interacting protein 2
Q2TAD9	*cyhr1-b*	Cysteine and histidine-rich protein 1-B
Q5SSF7	*Fam46c*	Protein FAM46C
Q3T1J8	*Rtfdc1*	Protein RTF2 homolog
P22270	*Oct-TyrR*	Tyramine/octopamine receptor

A8WG21	*snx10b*	Sorting nexin-10B
Q7T339	*chmp5*	Charged multivesicular body protein 5
O88902	*Ptpn23*	Tyrosine-protein phosphatase non-receptor type 23 (Fragment)
Q13501	*SQSTM1*	Sequestosome-1
Q56A36	*hsh2d*	Hematopoietic SH2 domain-containing protein homolog
Q64092	*Tfe3*	Transcription factor E3
Q9UKP5	*ADAMTS6*	A disintegrin and metalloproteinase with thrombospondin motifs 6
Q9Y6F1	*PARP3*	Poly [ADP-ribose] polymerase 3
Q28G05	*syf2*	Pre-mRNA-splicing factor syf2
Q6R653	*Unc5cl*	UNC5C-like protein
Q9UN76	*SLC6A14*	Sodium- and chloride-dependent neutral and basic amino acid transporter B(0+)
Q8K1N1	*Pnpla8*	Calcium-independent phospholipase A2-gamma
Q5XTS1	*PNPLA8*	Calcium-independent phospholipase A2-gamma
Q16TM5	*AAEL010189*	Band 7 protein AAEL010189
Q99719	*43713*	Septin-5
Q95SX7	*RTase*	Probable RNA-directed DNA polymerase from transposon BS
Q99K70	*Rragc*	Ras-related GTP-binding protein C
P54254	*Atxn1*	Ataxin-1
Q8BP74	*Pstk*	L-seryl-tRNA(Sec) kinase
Q13163	*MAP2K5*	Dual specificity mitogen-activated protein kinase kinase 5
P13255	*Gnmt*	Glycine N-methyltransferase
Q9Y6R7	*FCGBP*	IgGFc-binding protein
Q96JH7	*VCPIP1*	Deubiquitinating protein VCIP135
B3EWY9		Mucin-like protein (Fragment)
Q9Y6V7	*DDX49*	Probable ATP-dependent RNA helicase DDX49
Q8IZ41	*RASEF*	Ras and EF-hand domain-containing protein

Q9NUS5	*AP5S1*	AP-5 complex subunit sigma-1
Q3SZY9	*MED6*	Mediator of RNA polymerase II transcription subunit 6
Q96NW4	*ANKRD27*	Ankyrin repeat domain-containing protein 27
Q5RA95	*NDRG3*	Protein NDRG3
P10760	*Ahcy*	Adenosylhomocysteinase
Q32N90	*mbtd1*	MBT domain-containing protein 1
Q09575	*K02A2.6*	Uncharacterized protein K02A2.6
Q0PV50	*TLR3*	Toll-like receptor 3
Q08DJ7	*AAR2*	Protein AAR2 homolog
O43166	*SIPA1L1*	Signal-induced proliferation-associated 1-like protein 1
Q5RBW6	*STX12*	Syntaxin-12
Q5R8L2	*BMI1*	Polycomb complex protein BMI-1
P41971	*Elk3*	ETS domain-containing protein Elk-3
Q12829	*RAB40B*	Ras-related protein Rab-40B
Q3UDK1	*Trafd1*	TRAF-type zinc finger domain-containing protein 1
P47934	*Crat*	Carnitine O-acetyltransferase
Q96JN8	*NEURL4*	Neuralized-like protein 4
Q5ZJH7	*CNPPD1*	Protein CNPPD1
Q9GKW8	*ANKDD1A*	Ankyrin repeat and death domain-containing protein 1A (Fragment)
P25425	*Pou2f1*	POU domain, class 2, transcription factor 1
Q13501	*SQSTM1*	Sequestosome-1
Q8NEZ2	*VPS37A*	Vacuolar protein sorting-associated protein 37A
P49916	*LIG3*	DNA ligase 3
O08727	*Tnfrsf11b*	Tumor necrosis factor receptor superfamily member 11B
Q6NVL7	*chmp2b*	Charged multivesicular body protein 2b
P20107	*ZRC1*	Zinc/cadmium resistance protein

Q9JI90	*Rnf14*	E3 ubiquitin-protein ligase RNF14
Q14596	*NBR1*	Next to BRCA1 gene 1 protein
Q5XI06	*Kat8*	Histone acetyltransferase KAT8
Q4V8K5	*Brox*	BRO1 domain-containing protein BROX
P01119	*RAS1*	Ras-like protein 1
Q1L5Z9	*LONRF2*	LON peptidase N-terminal domain and RING finger protein 2
Q6P0D1	*slc30a6*	Zinc transporter 6
Q09225	*nrf-6*	Nose resistant to fluoxetine protein 6
Q1LV22	*fam214a*	Protein FAM214A
P17713	*STK*	Tyrosine-protein kinase STK
Q6GR25	*pptc7*	Protein phosphatase PTC7 homolog
Q9H3F6	*KCTD10*	BTB/POZ domain-containing adapter for CUL3-mediated RhoA degradation protein 3
Q13829	*TNFAIP1*	BTB/POZ domain-containing adapter for CUL3-mediated RhoA degradation protein 2
Q8C0D5	*Eftud1*	Elongation factor Tu GTP-binding domain-containing protein 1
Q02883	*FMP30*	N-acyl-phosphatidylethanolamine-hydrolyzing phospholipase D, mitochondrial
Q6PGV1	*atp6v0d1*	V-type proton ATPase subunit d 1
Q4R8V8	*CCDC37*	Coiled-coil domain-containing protein 37
Q9EPQ0	*Slc24a3*	Sodium/potassium/calcium exchanger 3 (Fragment)
Q5SSH7	*Zzef1*	Zinc finger ZZ-type and EF-hand domain-containing protein 1
Q5EAJ7	*MVP*	Major vault protein
Q9P3U4	*dbl4*	E3 ubiquitin-protein ligase dbl4
Q08509	*Eps8*	Epidermal growth factor receptor kinase substrate 8
Q96JH7	*VCPIP1*	Deubiquitinating protein VCIP135
Q9GLV6	*DDX58*	Probable ATP-dependent RNA helicase DDX58
Q9R1D7	*GBF1*	Golgi-specific brefeldin A-resistance guanine nucleotide exchange factor 1
Q06248	*HSP70IV*	Heat shock 70 kDa protein IV

P40918	*HSP70*	Heat shock 70 kDa protein
Q06248	*HSP70IV*	Heat shock 70 kDa protein IV
Q6KAU4	*Mvb12b*	Multivesicular body subunit 12B
Q9HD42	*CHMP1A*	Charged multivesicular body protein 1a
Q7ZW25	*chmp2a*	Charged multivesicular body protein 2a
P14198	*AAC4*	AAC-rich mRNA clone AAC4 protein
Q06248	*HSP70IV*	Heat shock 70 kDa protein IV
O14727	*APAF1*	Apoptotic protease-activating factor 1
O94913	*PCF11*	Pre-mRNA cleavage complex 2 protein Pcf11
Q9QUK4	*Naip2*	Baculoviral IAP repeat-containing protein 1b
Q5ZL54	*RCJMB04_7l9*	UPF0469 protein KIAA0907 homolog
Q8R0S1	*Atf7*	Cyclic AMP-dependent transcription factor ATF-7
O76536		Hyalin (Fragment)
D9IQ16		Galaxin
D9IQ16		Galaxin
Q7T0V2	*lamtor3-a*	Ragulator complex protein LAMTOR3-A
Q7T0V2	*lamtor3-a*	Ragulator complex protein LAMTOR3-A
Q803H0	*zgc:55781*	UPF0415 protein C7orf25 homolog
Q9P959	*alxA*	Alternative oxidase, mitochondrial
Q6VAB6	*KSR2*	Kinase suppressor of Ras 2
Q6PBT5	*pyroxd1*	Pyridine nucleotide-disulfide oxidoreductase domain-containing protein 1
Q92805	*GOLGA1*	Golgin subfamily A member 1
Q80UU1	*Ankzf1*	Ankyrin repeat and zinc finger domain-containing protein 1
Q05922	*Dusp2*	Dual specificity protein phosphatase 2
P42839	*VNX1*	Low affinity vacuolar monovalent cation/H(+) antiporter
Q3U5F4	*Yrdc*	YrdC domain-containing protein, mitochondrial

Q6IE24	*Usp54*	Inactive ubiquitin carboxyl-terminal hydrolase 54
Q99758	*ABCA3*	ATP-binding cassette sub-family A member 3
Q9H2G9	*BLZF1*	Golgin-45
Q99719	*43713*	Septin-5
P55196	*MLLT4*	Afadin
Q8IYT8	*ULK2*	Serine/threonine-protein kinase ULK2
Q1HPK6	*tef2*	Translation elongation factor 2
Q12923	*PTPN13*	Tyrosine-protein phosphatase non-receptor type 13
Q8R420	*Abca3*	ATP-binding cassette sub-family A member 3
Q5REY2	*RPL27A*	60S ribosomal protein L27a
Q5VT06	*CEP350*	Centrosome-associated protein 350
Q9UPW6	*SATB2*	DNA-binding protein SATB2
Q9NQR8	*Ndufaf4*	NADH dehydrogenase [ubiquinone] 1 alpha subcomplex assembly factor 4
Q4VGL6	*Rc3h1*	Roquin-1
Q99KY4	*Gak*	Cyclin-G-associated kinase
A2RSY1	*Kansl3*	KAT8 regulatory NSL complex subunit 3
Q8NCJ5	*SPRYD3*	SPRY domain-containing protein 3
Q6Q899	*Ddx58*	Probable ATP-dependent RNA helicase DDX58
O95786	*DDX58*	Probable ATP-dependent RNA helicase DDX58
Q8BG99	*Pknox2*	Homeobox protein PKNOX2
Q4V7H1	*stil*	SCL-interrupting locus protein homolog
Q9C5D2	*FBL4*	F-box/LRR-repeat protein 4
Q941Q3	*HUA1*	Zinc finger CCCH domain-containing protein 37
Q80U95	*Ube3c*	Ubiquitin-protein ligase E3C
Q15386	*UBE3C*	Ubiquitin-protein ligase E3C
Q15386	*UBE3C*	Ubiquitin-protein ligase E3C

Q03468	*ERCC6*	DNA excision repair protein ERCC-6
Q99758	*ABCA3*	ATP-binding cassette sub-family A member 3
Q8R420	*Abca3*	ATP-binding cassette sub-family A member 3
Q8IWB9	*TEX2*	Testis-expressed sequence 2 protein
Q6PGY5	*dnajc21*	DnaJ homolog subfamily C member 21
Q64194	*Lipa*	Lysosomal acid lipase/cholesteryl ester hydrolase
O43592	*XPOT*	Exportin-T
P10385		Glutenin, low molecular weight subunit
Q92636	*NSMAF*	Protein FAN
Q4V8I4	*Def8*	Differentially expressed in FDCP 8 homolog
Q6PE18	*pi4k2a*	Phosphatidylinositol 4-kinase type 2-alpha
Q28G26	*pi4k2b*	Phosphatidylinositol 4-kinase type 2-beta
Q96HW7	*INTS4*	Integrator complex subunit 4
A4IIC5	*slc39a3*	Zinc transporter ZIP3
Q6PC69	*rpl10a*	60S ribosomal protein L10a

Table S14. Swiss-port annotation of genes in profile C.

Swiss-prot ID	Gene Name	Description
Q4V7C1	*Fgfr1op*	FGFR1 oncogene partner
A2AQ19	*Rtf1*	RNA polymerase-associated protein RTF1 homolog
P04757	*Chrna3*	Neuronal acetylcholine receptor subunit alpha-3
P62895	*CYCS*	Cytochrome c
Q8IYW5	*RNF168*	E3 ubiquitin-protein ligase RNF168
P58283	*Rnf216*	E3 ubiquitin-protein ligase RNF216
P33992	*MCM5*	DNA replication licensing factor MCM5
Q5PQ53	*pef1*	Peflin
O18883	*TXNDC9*	Thioredoxin domain-containing protein 9
Q8WUR0		Protein C19orf12 homolog
Q7ZVE3	*tigarb*	Fructose-2,6-bisphosphatase TIGAR B
P0DMQ6	*SORD*	Sorbitol dehydrogenase
Q8WPA2	*AR*	Allatostatin-A receptor
P08761	*Eip71CD*	Peptide methionine sulfoxide reductase
Q9Y5R5	*DMRT2*	Doublesex- and mab-3-related transcription factor 2
Q6DBY0	*nup85*	Nuclear pore complex protein Nup85
O93336	*hs2st1*	Heparan sulfate 2-O-sulfotransferase 1
Q9VUV9	*l(3)72Ab*	Putative U5 small nuclear ribonucleoprotein 200 kDa helicase
A7RR04	*v1g236547*	Elongation factor G, mitochondrial
Q5JXM2	*METTL24*	Methyltransferase-like protein 24
Q2KIA5	*DNM1L*	Dynamin-1-like protein
Q08BI9	*mcu*	Calcium uniporter protein, mitochondrial
Q9HAV4	*XPO5*	Exportin-5
O94903	*PROSC*	Proline synthase co-transcribed bacterial homolog protein

Q99LD9	*Eif2b2*	Translation initiation factor eIF-2B subunit beta
Q8BU03	*Pwp2*	Periodic tryptophan protein 2 homolog
Q5RC70	*DNAJC11*	DnaJ homolog subfamily C member 11
Q28HC7	*commd6*	COMM domain-containing protein 6
Q8TE02	*ELP5*	Elongator complex protein 5
A5PKG7	*KCTD5*	BTB/POZ domain-containing protein KCTD5
P13437	*Acaa2*	3-ketoacyl-CoA thiolase, mitochondrial
Q2TBR0	*PCCB*	Propionyl-CoA carboxylase beta chain, mitochondrial
Q6P8D9	*ube2m*	NEDD8-conjugating enzyme Ubc12
Q8N5C7	*DTWD1*	DTW domain-containing protein 1
P09110	*ACAA1*	3-ketoacyl-CoA thiolase, peroxisomal
Q6P6Z0	*ddb1*	DNA damage-binding protein 1
P05625	*RAF1*	RAF proto-oncogene serine/threonine-protein kinase
Q58DV5	*MRPL30*	39S ribosomal protein L30, mitochondrial
Q925Q5	*Gpha2*	Glycoprotein hormone alpha-2
Q32KX5	*LRRC28*	Leucine-rich repeat-containing protein 28
Q96EQ9	*Prdm9*	Histone-lysine N-methyltransferase PRDM9
Q9UBC2	*EPS15L1*	Epidermal growth factor receptor substrate 15-like 1
P28491	*CALR*	Calreticulin
Q9D720	*Nsmce1*	Non-structural maintenance of chromosomes element 1 homolog
Q6DE14	*e2f4*	Transcription factor E2F4
Q8N972	*ZNF709*	Zinc finger protein 709
B7Q290	*ISCW010018*	Uncharacterized protein NCBP2-AS2 homolog
Q04462	*Vars*	Valine--tRNA ligase
P23610	*F8A1*	Factor VIII intron 22 protein
Q6PBE2	*bcas2*	Pre-mRNA-splicing factor SPF27

Q5ZJH5	*WDR61*	WD repeat-containing protein 61
Q6P2U9	*cops3*	COP9 signalosome complex subunit 3
Q5FWF5	*ESCO1*	N-acetyltransferase ESCO1
Q96B26	*EXOSC8*	Exosome complex component RRP43
Q7ZYD5	*slc25a25*	Calcium-binding mitochondrial carrier protein SCaMC-2
Q5RBL1	*GARS*	Glycine--tRNA ligase
Q96PN7	*TRERF1*	Transcriptional-regulating factor 1
A8WH18	*narfl*	Cytosolic Fe-S cluster assembly factor narfl
P48449	*LSS*	Lanosterol synthase
Q8BLN5	*Lss*	Lanosterol synthase
Q96G46	*DUS3L*	tRNA-dihydrouridine(47) synthase [NAD(P)(+)]-like
O75096	*LRP4*	Low-density lipoprotein receptor-related protein 4
Q99497	*PARK7*	Protein deglycase DJ-1
P05556	*ITGB1*	Integrin beta-1
B2RYZ5	*eny2*	Transcription and mRNA export factor ENY2
Q6AY46	*Trmt61a*	tRNA (adenine(58)-N(1))-methyltransferase catalytic subunit TRMT61A
O43772	*SLC25A20*	Mitochondrial carnitine/acylcarnitine carrier protein
P81127	*NAPG*	Gamma-soluble NSF attachment protein
Q28CZ9	*cyb5r4*	Cytochrome b5 reductase 4
Q9JLZ3	*Auh*	Methylglutaconyl-CoA hydratase, mitochondrial
Q5XIC0	*Eci2*	Enoyl-CoA delta isomerase 2, mitochondrial
Q9UJW0	*DCTN4*	Dynactin subunit 4
Q9ULI0	*ATAD2B*	ATPase family AAA domain-containing protein 2B
Q6GNI6	*usp22-a*	Ubiquitin carboxyl-terminal hydrolase 22-A
Q5BLE8	*retsat*	Putative all-trans-retinol 13,14-reductase
A4FUF0	*GLYR1*	Putative oxidoreductase GLYR1

O02213	*ser-2*	Tyramine receptor Ser-2
Q8BRF7	*Scfd1*	Sec1 family domain-containing protein 1
Q5E9L7	*VPS16*	Vacuolar protein sorting-associated protein 16 homolog
P07872	*Acox1*	Peroxisomal acyl-coenzyme A oxidase 1
Q3T0Q3	*POLR2C*	DNA-directed RNA polymerase II subunit RPB3
Q5F359	*TRAPPC5*	Trafficking protein particle complex subunit 5
Q6PA06	*Atl2*	Atlastin-2
O75716	*STK16*	Serine/threonine-protein kinase 16
O55071	*Cyp2b19*	Cytochrome P450 2B19
Q8VBT0	*Tmx1*	Thioredoxin-related transmembrane protein 1
Q8N9H8	*EXD3*	Exonuclease mut-7 homolog
A6PW82	*CXorf30*	Putative uncharacterized protein CXorf30
O75486	*SUPT3H*	Transcription initiation protein SPT3 homolog
O01761	*unc-89*	Muscle M-line assembly protein unc-89
Q91VH2	*Snx9*	Sorting nexin-9
Q5FVV3	*chchd6*	MICOS complex subunit mic25
Q8NDG6	*TDRD9*	Putative ATP-dependent RNA helicase TDRD9
P60881	*Snap25*	Synaptosomal-associated protein 25
Q9YHT2	*SDHB*	Succinate dehydrogenase [ubiquinone] iron-sulfur subunit, mitochondrial
Q3B8J8	*sdhb*	Succinate dehydrogenase [ubiquinone] iron-sulfur subunit, mitochondrial
P50473		Arylsulfatase
Q05752	*NDUFA7*	NADH dehydrogenase [ubiquinone] 1 alpha subcomplex subunit 7
Q56W64	*At5g61540*	Probable isoaspartyl peptidase/L-asparaginase 3
Q9Y6I9	*TEX264*	Testis-expressed sequence 264 protein
A3KPL7	*tmem170a*	Transmembrane protein 170A
Q21697	*R04B3.2*	Putative N(4)-(beta-N-acetylglucosaminyl)-L-asparaginase

Q92122	*pkm*	Pyruvate kinase PKM
Q28GP7	*snx33*	Sorting nexin-33
Q4VAA7	*Snx33*	Sorting nexin-33
Q5U4V2	*hnmt-a*	Histamine N-methyltransferase A
Q9JIY6	*Cml6*	Probable N-acetyltransferase CML6
Q5T200	*ZC3H13*	Zinc finger CCCH domain-containing protein 13
Q5RFD6	*CPM*	Carboxypeptidase M
Q6P819	*rqcd1*	Cell differentiation protein RCD1 homolog
A7RM45	*v1g160387*	Probable lysosomal cobalamin transporter
Q9NW38	*FANCL*	E3 ubiquitin-protein ligase FANCL
Q9CZR2	*Naalad2*	N-acetylated-alpha-linked acidic dipeptidase 2
Q6AZW2	*akr1a1a*	Alcohol dehydrogenase [NADP(+)] A
Q8BY79	*Slc35g1*	Solute carrier family 35 member G1
Q64674	*Srm*	Spermidine synthase
Q9QZU7	*Bbox1*	Gamma-butyrobetaine dioxygenase
Q96FV9	*THOC1*	THO complex subunit 1
Q14117	*DPYS*	Dihydropyrimidinase
Q8TBZ3	*WDR20*	WD repeat-containing protein 20
Q6DGV1	*celf4*	CUGBP Elav-like family member 4
Q5RBK3	*HACD2*	Very-long-chain (3R)-3-hydroxyacyl-CoA dehydratase 2
Q9X248	*fabG*	3-oxoacyl-[acyl-carrier-protein] reductase FabG
Q9CXW2	*Mrps22*	28S ribosomal protein S22, mitochondrial
P23606	*Tgm1*	Protein-glutamine gamma-glutamyltransferase K
P41252	*IARS*	Isoleucine--tRNA ligase, cytoplasmic
Q6ZNE5	*ATG14*	Beclin 1-associated autophagy-related key regulator
Q8TDX6	*CSGALNACT1*	Chondroitin sulfate N-acetylgalactosaminyltransferase 1

O08912	*Galnt1*	Polypeptide N-acetylgalactosaminyltransferase 1
O57579	*ANPEP*	Aminopeptidase N
Q58CW6	*CARF*	Calcium-responsive transcription factor
Q8N264	*ARHGAP24*	Rho GTPase-activating protein 24
O60306	*AQR*	Intron-binding protein aquarius
Q497B8	*Kdm8*	Lysine-specific demethylase 8
P53590	*SUCLG2*	Succinyl-CoA ligase [GDP-forming] subunit beta, mitochondrial (Fragment)
O97725	*NDUFA12*	NADH dehydrogenase [ubiquinone] 1 alpha subcomplex subunit 12
B3DJT0	*sart3*	Squamous cell carcinoma antigen recognized by T-cells 3
O43181	*NDUFS4*	NADH dehydrogenase [ubiquinone] iron-sulfur protein 4, mitochondrial
Q9NWU1	*OXSM*	3-oxoacyl-[acyl-carrier-protein] synthase, mitochondrial
Q9P0U1	*TOMM7*	Mitochondrial import receptor subunit TOM7 homolog
Q0MQB9	*NDUFA11*	NADH dehydrogenase [ubiquinone] 1 alpha subcomplex subunit 11
Q9D404	*Oxsm*	3-oxoacyl-[acyl-carrier-protein] synthase, mitochondrial
Q5ZMG1	*UFL1*	E3 UFM1-protein ligase 1
Q9Y0Y7	*CG2061*	LanC-like protein 3 homolog
B1H3B1	*fundc1*	FUN14 domain-containing protein 1
Q5M8Z6	*ass1*	Argininosuccinate synthase
Q99570	*PIK3R4*	Phosphoinositide 3-kinase regulatory subunit 4
Q1JPX3	*farsa*	Phenylalanine--tRNA ligase alpha subunit
Q9WTR6	*Slc7a11*	Cystine/glutamate transporter
Q8K2I3	*Fmo2*	Dimethylaniline monooxygenase [N-oxide-forming] 2
P32929	*CTH*	Cystathionine gamma-lyase
P47820	*Ace*	Angiotensin-converting enzyme
Q9FZ87	*GH3.17*	Indole-3-acetic acid-amido synthetase GH3.17
P48147	*PREP*	Prolyl endopeptidase

Q92621	*NUP205*	Nuclear pore complex protein Nup205
Q96KF7	*SMIM8*	Small integral membrane protein 8
A1K5T6	*azo1574*	UPF0061 protein azo1574
Q80YF0	*MAD1L1*	Mitotic spindle assembly checkpoint protein MAD1
Q9WVK8	*Cyp46a1*	Cholesterol 24-hydroxylase
P29451	*TXN*	Thioredoxin
Q5W5U4	*DDX4*	Probable ATP-dependent RNA helicase DDX4
Q9BTT6	*LRRC1*	Leucine-rich repeat-containing protein 1
P35623	*SHMT1*	Serine hydroxymethyltransferase, cytosolic
Q61133	*Gstt2*	Glutathione S-transferase theta-2
Q6IRP4	*abhd13*	Alpha/beta hydrolase domain-containing protein 13
P51553	*IDH3G*	Isocitrate dehydrogenase [NAD] subunit gamma, mitochondrial
P86179		L-rhamnose-binding lectin CSL3
Q5ZM16	*RBM22*	Pre-mRNA-splicing factor RBM22
Q91YN5	*Uap1*	UDP-N-acetylhexosamine pyrophosphorylase
P40426	*PBX3*	Pre-B-cell leukemia transcription factor 3
Q86PM4	*FGFR*	Fibroblast growth factor receptor
O42602	*crhr1*	Corticotropin-releasing factor receptor 1
O18835	*GUSB*	Beta-glucuronidase
Q8IWX8	*CHERP*	Calcium homeostasis endoplasmic reticulum protein
A1L244	*sacm1la*	Phosphatidylinositide phosphatase SAC1-A
E7EY42	*ptdss2*	Phosphatidylserine synthase 2
A0AK43	*menG*	Demethylmenaquinone methyltransferase
Q8BG05	*Hnrnpa3*	Heterogeneous nuclear ribonucleoprotein A3
O02414		Dynein light chain LC6, flagellar outer arm
Q9P0L0	*VAPA*	Vesicle-associated membrane protein-associated protein A

O77750	*AQP4*	Aquaporin-4
O75317	*USP12*	Ubiquitin carboxyl-terminal hydrolase 12
Q8BHN3	*Ganab*	Neutral alpha-glucosidase AB
O60760	*HPGDS*	Hematopoietic prostaglandin D synthase
Q9W0T1	*E(bx)*	Nucleosome-remodeling factor subunit NURF301
Q92734	*TFG*	Protein TFG
O70422	*Gtf2h4*	General transcription factor IIH subunit 4
Q9Y385	*UBE2J1*	Ubiquitin-conjugating enzyme E2 J1
Q13126	*MTAP*	S-methyl-5'-thioadenosine phosphorylase
Q8K5B3	*Mcfd2*	Multiple coagulation factor deficiency protein 2 homolog
Q6IQX7	*Chpf*	Chondroitin sulfate synthase 2
A5D989	*EEF1D*	Elongation factor 1-delta
P29693	*eef1d*	Elongation factor 1-delta
P60984	*GMFB*	Glia maturation factor beta
Q684R7	*Frem1*	FRAS1-related extracellular matrix protein 1
Q95LA2	*FMO1*	Dimethylaniline monooxygenase [N-oxide-forming] 1
Q28XE2	*Anp32a*	Acidic leucine-rich nuclear phosphoprotein 32 family member A
Q6AZN6	*pik3c3*	Phosphatidylinositol 3-kinase catalytic subunit type 3
Q6PF93	*Pik3c3*	Phosphatidylinositol 3-kinase catalytic subunit type 3
Q7SYJ9	*spag7*	Sperm-associated antigen 7 homolog
P18708	*NSF*	Vesicle-fusing ATPase
P28570	*Slc6a8*	Sodium- and chloride-dependent creatine transporter 1
Q9DBW3	*Natd1*	Protein NATD1
Q3T114	*HRSP12*	Ribonuclease UK114
Q6GMH0	*prpf18*	Pre-mRNA-splicing factor 18
Q28BP9	*faf2*	FAS-associated factor 2

P13497	*BMP1*	Bone morphogenetic protein 1
P98198	*ATP8B2*	Phospholipid-transporting ATPase ID
Q96NG8	*ZNF582*	Zinc finger protein 582
Q58DQ5	*MRPS9*	28S ribosomal protein S9, mitochondrial
Q924A2	*Cic*	Protein capicua homolog
Q96RK0	*CIC*	Protein capicua homolog
Q9BWU0	*SLC4A1AP*	Kanadaptin
P29241		ADP-ribosyl cyclase/cyclic ADP-ribose hydrolase
P24802	*PLOD1*	Procollagen-lysine,2-oxoglutarate 5-dioxygenase 1
Q5XI31	*Pigs*	GPI transamidase component PIG-S
A6QM00	*GADL1*	Acidic amino acid decarboxylase GADL1
Q80WP8	*Gadl1*	Acidic amino acid decarboxylase GADL1
Q8MJY8	*HPGD*	15-hydroxyprostaglandin dehydrogenase [NAD(+)]
Q08D62	*mael*	Protein maelstrom homolog
Q2T9W6	*LRRFIP2*	Leucine-rich repeat flightless-interacting protein 2
Q5M8G4	*lrrc40*	Leucine-rich repeat-containing protein 40
O75165	*DNAJC13*	DnaJ homolog subfamily C member 13
Q566I1	*ino80d*	INO80 complex subunit D
Q9Z2F5	*Ctbp1*	C-terminal-binding protein 1
Q6P0E5	*ddrgk1*	DDRGK domain-containing protein 1
Q6NU94	*mettl1*	tRNA (guanine-N(7)-)-methyltransferase
P14618	*PKM*	Pyruvate kinase PKM
Q9DCF9	*Ssr3*	Translocon-associated protein subunit gamma
P61218	*POLR2F*	DNA-directed RNA polymerases I, II, and III subunit RPABC2
Q8K354	*Cbr3*	Carbonyl reductase [NADPH] 3
Q5R8B0	*BRD7*	Bromodomain-containing protein 7

Q6DCU7		Protein Hikeshi
Q15155	*NOMO1*	Nodal modulator 1
Q5ZLY5	*PLEKHF2*	Pleckstrin homology domain-containing family F member 2
Q9GKZ4	*TRAM1*	Translocating chain-associated membrane protein 1
A7MBJ5	*CAND1*	Cullin-associated NEDD8-dissociated protein 1
Q9V998	*ubl*	Ubiquitin-like protein 5
Q8AVH7	*egfl6*	Epidermal growth factor-like protein 6
Q91233	*hsp70*	Heat shock 70 kDa protein
Q08BN9	*nxpe3*	NXPE family member 3
Q9CY21	*Wbscr22*	Probable 18S rRNA (guanine-N(7))-methyltransferase
P22892	*Ap1g1*	AP-1 complex subunit gamma-1
Q00004	*SRP68*	Signal recognition particle subunit SRP68
Q3SZA2		UPF0562 protein C7orf55 homolog
Q5EA33	*ANKRD49*	Ankyrin repeat domain-containing protein 49
O75828	*CBR3*	Carbonyl reductase [NADPH] 3
Q4V8D1	*Cyp2u1*	Cytochrome P450 2U1
P12939	*Cyp2d10*	Cytochrome P450 2D10
Q9CX98	*Cyp2u1*	Cytochrome P450 2U1
P00185	*Cyp1a1*	Cytochrome P450 1A1
E1B7L7	*UBN2*	Ubinuclein-2
Q6PC30	*cops5*	COP9 signalosome complex subunit 5
Q6P635	*cops5*	COP9 signalosome complex subunit 5
O88968	*Tcn2*	Transcobalamin-2
Q9Z2D0	*Mtmr9*	Myotubularin-related protein 9
Q9NUP9	*LIN7C*	Protein lin-7 homolog C
Q9CU62	*Smc1a*	Structural maintenance of chromosomes protein 1A

O95985	*TOP3B*	DNA topoisomerase 3-beta-1
P51452	*DUSP3*	Dual specificity protein phosphatase 3
Q5SPC5	*otof*	Otoferlin
Q561P5	*mcm5*	DNA replication licensing factor mcm5
Q6P9I7	*smc6*	Structural maintenance of chromosomes protein 6
O54750	*Cyp2j6*	Cytochrome P450 2J6
Q14671	*PUM1*	Pumilio homolog 1
P48679	*Lmna*	Prelamin-A/C
P50994	*ANXA4*	Annexin A4
Q9Z1Z2	*Strap*	Serine-threonine kinase receptor-associated protein
Q9QXN5	*Miox*	Inositol oxygenase
Q13614	*MTMR2*	Myotubularin-related protein 2
P39021	*meox2*	Homeobox protein MOX-2
Q9DCJ9	*Npl*	N-acetylneuraminate lyase
P62310	*LSM3*	U6 snRNA-associated Sm-like protein LSm3
Q6GLC5	*emc6*	ER membrane protein complex subunit 6
Q28ID3	*glrx3*	Glutaredoxin-3
Q0V9P1	*hnmt*	Histamine N-methyltransferase
P23588	*EIF4B*	Eukaryotic translation initiation factor 4B
Q921M4	*Golga2*	Golgin subfamily A member 2
Q6PH90	*rbm8a-b*	RNA-binding protein 8A-B
Q9IB83	*psmb1-B*	Proteasome subunit beta type-1-B
Q9IB84	*psmb1-A*	Proteasome subunit beta type-1-A
Q78P75	*Dynll2*	Dynein light chain 2, cytoplasmic
O73672	*psma2*	Proteasome subunit alpha type-2
Q96253	*At1g51650*	ATP synthase subunit epsilon, mitochondrial

Q8R4U2	*P4HB*	Protein disulfide-isomerase
Q8LGG0	*FKBP12*	Peptidyl-prolyl cis-trans isomerase FKBP12
Q9EQ28	*Pold3*	DNA polymerase delta subunit 3
Q9FUN0	*HMT-1*	Homocysteine S-methyltransferase 1
Q8BYB9	*Poglut1*	Protein O-glucosyltransferase 1
Q6DFT6	*sptssa*	Serine palmitoyltransferase small subunit A
P42573	*ced-3*	Cell death protein 3
Q98943	*CASP2*	Caspase-2
A5GFY4	*NELFCD*	Negative elongation factor D
Q2KJ81	*AP1M1*	AP-1 complex subunit mu-1
D4G3R4	*wapA*	tRNA(Glu)-specific nuclease WapA
Q08705	*CTCF*	Transcriptional repressor CTCF
Q9VCA2	*Orct*	Organic cation transporter protein
Q6ZUK4	*TMEM26*	Transmembrane protein 26
Q63159	*Coq3*	Ubiquinone biosynthesis O-methyltransferase, mitochondrial
Q8WUW1	*BRK1*	Protein BRICK1
Q5RAX9	*PRDM10*	PR domain zinc finger protein 10
Q9D8U7	*Dtwd1*	DTW domain-containing protein 1
Q8N567	*ZCCHC9*	Zinc finger CCHC domain-containing protein 9
Q9Y2U5	*MAP3K2*	Mitogen-activated protein kinase kinase kinase 2
Q5RAG7	*SLC7A11*	Cystine/glutamate transporter
P35749	*MYH11*	Myosin-11
Q96RW7	*HMCN1*	Hemicentin-1
Q5ZM20	*RMI2*	RecQ-mediated genome instability protein 2
Q04759	*PRKCQ*	Protein kinase C theta type
Q8IW19	*APLF*	Aprataxin and PNK-like factor

C1C4R8	*bdh2*	3-hydroxybutyrate dehydrogenase type 2
Q12718	*LCC2*	Laccase-2
Q6TUD4	*Yipf3*	Protein YIPF3
Q6AXS5	*Serbp1*	Plasminogen activator inhibitor 1 RNA-binding protein
Q9DBR3	*Armc8*	Armadillo repeat-containing protein 8
Q2KJG4	*MGST2*	Microsomal glutathione S-transferase 2
Q2VL90	*CD163*	Scavenger receptor cysteine-rich type 1 protein M130
Q95218	*Dmbt1*	Deleted in malignant brain tumors 1 protein
Q7ZUG0	*snrpe*	Small nuclear ribonucleoprotein E
P07228	*ITGB1*	Integrin beta-1
Q6NXG1	*ESRP1*	Epithelial splicing regulatory protein 1
Q8TCJ2	*STT3B*	Dolichyl-diphosphooligosaccharide--protein glycosyltransferase subunit STT3B
Q96GM5	*SMARCD1*	SWI/SNF-related matrix-associated actin-dependent regulator of chromatin subfamily D member 1
Q8JG64	*PDIA3*	Protein disulfide-isomerase A3
Q3SZL5	*PIGS*	GPI transamidase component PIG-S
A4IH82	*sh3bp5l*	SH3 domain-binding protein 5-like
P22124		Ras-related protein O-RAL
Q54ED7	*pks40*	Probable polyketide synthase 40
Q9TUL9	*TIMP3*	Metalloproteinase inhibitor 3
Q0D289	*zgc:153595*	Uncharacterized protein C16orf52 homolog
Q5G269	*PRSS12*	Neurotrypsin
P21328	*pol*	RNA-directed DNA polymerase from mobile element jockey
Q4V8C3	*Eml1*	Echinoderm microtubule-associated protein-like 1
Q5M9I6	*Mmtag2*	Multiple myeloma tumor-associated protein 2 homolog
Q8VE97	*Srsf4*	Serine/arginine-rich splicing factor 4
P49907	*SEPP1*	Selenoprotein P

Q9Y6C9	*MTCH2*	Mitochondrial carrier homolog 2
Q3T073	*SERP2*	Stress-associated endoplasmic reticulum protein 2
Q9WUE3	*Cyb561d2*	Cytochrome b561 domain-containing protein 2
Q5RF00	*ALDH2*	Aldehyde dehydrogenase, mitochondrial
O76536		Hyalin (Fragment)
Q9XSK2	*CD63*	CD63 antigen
Q28GH3	*uba2*	SUMO-activating enzyme subunit 2
Q5VT52	*RPRD2*	Regulation of nuclear pre-mRNA domain-containing protein 2
Q96MY1	*NOL4L*	Nucleolar protein 4-like
P26443	*Glud1*	Glutamate dehydrogenase 1, mitochondrial
Q80X72	*Lrrc15*	Leucine-rich repeat-containing protein 15
B1AT66	*Slc16a6*	Monocarboxylate transporter 7
P34059	*GALNS*	N-acetylgalactosamine-6-sulfatase
P68371	*TUBB4B*	Tubulin beta-4B chain
Q8VDL4	*Adpgk*	ADP-dependent glucokinase
Q8WPD0		Alpha-N-acetylgalactosamine-specific lectin
Q8BH74	*Nup107*	Nuclear pore complex protein Nup107
O08742	*Gp5*	Platelet glycoprotein V
Q9QXG4	*Acss2*	Acetyl-coenzyme A synthetase, cytoplasmic
Q8HXX4	*HADHB*	Trifunctional enzyme subunit beta, mitochondrial
O46629	*HADHB*	Trifunctional enzyme subunit beta, mitochondrial
Q5BKQ4	*Pnliprp1*	Inactive pancreatic lipase-related protein 1
Q6UB98	*ANKRD12*	Ankyrin repeat domain-containing protein 12
Q8AWB6	*SLC35B1*	Solute carrier family 35 member B1
Q5ZMP6	*AP2M1*	AP-2 complex subunit mu
Q8K3X2	*TSTA3*	GDP-L-fucose synthase

Q62280	*Ss18*	Protein SSXT
A2APV2	*Fmnl2*	Formin-like protein 2
Q803G5	*samm50a*	Sorting and assembly machinery component 50 homolog A
Q9P2J5	*LARS*	Leucine--tRNA ligase, cytoplasmic
Q8JFW4	*adat3*	Probable inactive tRNA-specific adenosine deaminase-like protein 3
P30676		Guanine nucleotide-binding protein G(i) subunit alpha
Q4LDE5	*SVEP1*	Sushi, von Willebrand factor type A, EGF and pentraxin domain-containing protein 1
O96530		Hyalin (Fragment)
Q8HZK3	*DUOX1*	Dual oxidase 1
E9QAM5	*Helz2*	Helicase with zinc finger domain 2
O88487	*Dync1i2*	Cytoplasmic dynein 1 intermediate chain 2
Q0MQG2	*NDUFS1*	NADH-ubiquinone oxidoreductase 75 kDa subunit, mitochondrial
Q66HF1	*Ndufs1*	NADH-ubiquinone oxidoreductase 75 kDa subunit, mitochondrial
Q62599	*Mta1*	Metastasis-associated protein MTA1
Q6P8X1	*Snx6*	Sorting nexin-6
Q9UNH7	*SNX6*	Sorting nexin-6
Q5U249	*ahctf1*	Protein ELYS
Q803T2	*strip1*	Striatin-interacting protein 1 homolog
A6H730	*ACPP*	Prostatic acid phosphatase
Q6NYL5	*gpt2l*	Alanine aminotransferase 2-like
P07511	*SHMT1*	Serine hydroxymethyltransferase, cytosolic
Q6DK99	*cnfn-b*	Cornifelin homolog B
P53396	*ACLY*	ATP-citrate synthase
Q3SZD7	*CBR1*	Carbonyl reductase [NADPH] 1
Q8NEZ4	*KMT2C*	Histone-lysine N-methyltransferase 2C
Q9TT99	*ATOX1*	Copper transport protein ATOX1

Q32PX7	*Fubp1*	Far upstream element-binding protein 1
P45985	*MAP2K4*	Dual specificity mitogen-activated protein kinase kinase 4
Q5G266	*PRSS12*	Neurotrypsin
Q69YN4	*KIAA1429*	Protein virilizer homolog
Q5REX3	*ZFR*	Zinc finger RNA-binding protein
Q0VFD6	*mzt1*	Mitotic-spindle organizing protein 1
B2RYI0	*Wdr91*	WD repeat-containing protein 91
P06180		Histone-binding protein N1/N2
Q8CJ11	*Gpr64*	G-protein coupled receptor 64
O35161	*Celsr1*	Cadherin EGF LAG seven-pass G-type receptor 1
Q3KNW5	*SLC10A6*	Solute carrier family 10 member 6
Q0III3	*DYNC1I2*	Cytoplasmic dynein 1 intermediate chain 2
P99029	*Prdx5*	Peroxiredoxin-5, mitochondrial
Q24560	*betaTub56D*	Tubulin beta-1 chain
Q6P5I8	*yipf5*	Protein YIPF5
Q9UJZ1	*STOML2*	Stomatin-like protein 2, mitochondrial
Q155U0	*vps51*	Vacuolar protein sorting-associated protein 51 homolog
Q6P963	*hagh*	Hydroxyacylglutathione hydrolase, mitochondrial
P35556	*FBN2*	Fibrillin-2
Q12873	*CHD3*	Chromodomain-helicase-DNA-binding protein 3
F1N9Y5	*SYK*	Tyrosine-protein kinase SYK
O00425	*IGF2BP3*	Insulin-like growth factor 2 mRNA-binding protein 3
C4A0D9	*BAP1*	Ubiquitin carboxyl-terminal hydrolase BAP1
Q9NYQ2	*Hao2*	Hydroxyacid oxidase 2
G5E8K5	*Ank3*	Ankyrin-3
A6QLR3	*FAM175B*	BRISC complex subunit Abro1

O17320		Actin
Q9Y296	*TRAPPC4*	Trafficking protein particle complex subunit 4
P18258		Tubulin alpha-1 chain
P06027		Echinoidin
A7MB35	*PDHA1*	Pyruvate dehydrogenase E1 component subunit alpha, somatic form, mitochondrial
O94826	*TOMM70A*	Mitochondrial import receptor subunit TOM70
Q8VEK0	*Tmem30a*	Cell cycle control protein 50A
O08747	*Unc5c*	Netrin receptor UNC5C
P78314	*SH3BP2*	SH3 domain-binding protein 2
Q90744	*NAGA*	Alpha-N-acetylgalactosaminidase
Q69ZL1	*Fgd6*	FYVE, RhoGEF and PH domain-containing protein 6
Q9WV92	*Epb41l3*	Band 4.1-like protein 3
O43491	*EPB41L2*	Band 4.1-like protein 2
P41383	*TUB2*	Tubulin alpha-2/alpha-4 chain
Q8VDM6	*Hnrnpul1*	Heterogeneous nuclear ribonucleoprotein U-like protein 1
Q24439	*Oscp*	ATP synthase subunit O, mitochondrial
Q0GA40	*LGSN*	Lengsin
O89079	*Cope*	Coatomer subunit epsilon
Q2RQU6	*tuf2*	Elongation factor Tu 2
Q6PCJ1	*dctn1*	Dynactin subunit 1
P9WIT3	*Rv1771*	L-gulono-1,4-lactone dehydrogenase
O19049	*HNRNPK*	Heterogeneous nuclear ribonucleoprotein K
P52846	*SULT1A1*	Sulfotransferase 1A1
O96952	*THIO*	Thioredoxin
Q9YHC3		Tubulin beta-1 chain
P31943	*HNRNPH1*	Heterogeneous nuclear ribonucleoprotein H

O08795	*Prkcsh*	Glucosidase 2 subunit beta
Q9BYK8	*HELZ2*	Helicase with zinc finger domain 2
O08810	*Eftud2*	116 kDa U5 small nuclear ribonucleoprotein component
Q4R6W9	*SNAPC1*	snRNA-activating protein complex subunit 1
Q5I7G2	*RXR*	Retinoic acid receptor RXR
Q9EQU5	*Set*	Protein SET
P21872	*GART*	Trifunctional purine biosynthetic protein adenosine-3
Q3ULD5	*Mccc2*	Methylcrotonoyl-CoA carboxylase beta chain, mitochondrial
P00349	*PGD*	6-phosphogluconate dehydrogenase, decarboxylating
Q14247	*CTTN*	Src substrate cortactin
P68365	*TUBA1C*	Tubulin alpha-1C chain
Q1HG43	*DUOXA1*	Dual oxidase maturation factor 1
Q4V7C7	*Actr3*	Actin-related protein 3
Q9NRD9	*DUOX1*	Dual oxidase 1
Q9P2R3	*ANKFY1*	Rabankyrin-5
Q61233	*Lcp1*	Plastin-2
Q5RAJ6	*DNAJB11*	DnaJ homolog subfamily B member 11
Q5SQX6	*Cyfip2*	Cytoplasmic FMR1-interacting protein 2
Q9UJ70	*NAGK*	N-acetyl-D-glucosamine kinase
Q16932		Syntaxin
Q5REK0	*FMO2*	Dimethylaniline monooxygenase [N-oxide-forming] 2
Q5PRD0	*ywhaba*	14-3-3 protein beta/alpha-A
O73932	*igf2bp3-a*	Insulin-like growth factor 2 mRNA-binding protein 3-A
P24798	*ATP1A3*	Sodium/potassium-transporting ATPase subunit alpha-3
P04040	*CAT*	Catalase
Q8VHI5	*Vit*	Vitrin

Q92673	*SORL1*	Sortilin-related receptor
Q9Y2M2	*SSUH2*	Protein SSUH2 homolog
P60900	*PSMA6*	Proteasome subunit alpha type-6
Q8AWF2	*naca*	Nascent polypeptide-associated complex subunit alpha
P09867	*HNRNPA1*	Heterogeneous nuclear ribonucleoprotein A1
Q9HC10	*OTOF*	Otoferlin
Q02650	*Pax5*	Paired box protein Pax-5
Q5RAP9	*ATP5G2*	ATP synthase F(0) complex subunit C2, mitochondrial
P17605	*ATP5G1*	ATP synthase F(0) complex subunit C1, mitochondrial
P21127	*CDK11B*	Cyclin-dependent kinase 11B
O15072	*ADAMTS3*	A disintegrin and metalloproteinase with thrombospondin motifs 3
P68373	*Tuba1c*	Tubulin alpha-1C chain
Q01205	*Dlst*	Dihydrolipoyllysine-residue succinyltransferase component of 2-oxoglutarate dehydrogenase complex, mitochondrial
Q9VSS2	*Srp68*	Signal recognition particle subunit SRP68
Q13454	*TUSC3*	Tumor suppressor candidate 3
Q66S17		Natterin-3
Q58DT8	*WDR55*	WD repeat-containing protein 55
Q91060		Tubulin alpha chain
A8E7I5	*ttc36*	Tetratricopeptide repeat protein 36
P07207	*N*	Neurogenic locus Notch protein
Q6NU14	*hnrnpdl-b*	Heterogeneous nuclear ribonucleoprotein D-like-B
Q98SJ2	*dazap1*	DAZ-associated protein 1
A2A9C3	*Szt2*	Protein SZT2
Q9CQ86	*Mien1*	Migration and invasion enhancer 1
B5XGE7	*trappc2l*	Trafficking protein particle complex subunit 2-like protein
P08133	*ANXA6*	Annexin A6

P61858	*betaTub85D*	Tubulin beta-2 chain
Q7ZVR8	*esrp2*	Epithelial splicing regulatory protein 2
Q9JIQ8	*Tmprss2*	Transmembrane protease serine 2
Q99MS4	*Prss29*	Serine protease 29
P00745	*PROC*	Vitamin K-dependent protein C (Fragment)
Q7XJJ7	*FAAH*	Fatty acid amide hydrolase
P06603	*alphaTub84B*	Tubulin alpha-1 chain
P52273		Tubulin alpha chain
Q8CIY2	*Duox1*	Dual oxidase 1
O55035	*Ppig*	Peptidyl-prolyl cis-trans isomerase G
Q69CJ9	*RPL35*	60S ribosomal protein L35
P01023	*A2M*	Alpha-2-macroglobulin
P08049	*MME*	Neprilysin
Q6P4Z2	*col2a1*	Collagen alpha-1(II) chain
Q5E956	*TPI1*	Triosephosphate isomerase
Q9UHR5	*SAP30BP*	SAP30-binding protein
Q1JP79	*ARPC1A*	Actin-related protein 2/3 complex subunit 1A
Q00685	*C3*	Complement C3 (Fragment)
Q04832	*HEXBP*	DNA-binding protein HEXBP
P22271	*PROA*	Profilin-A
Q7YQM0	*GDI1*	Rab GDP dissociation inhibitor alpha
Q99729	*HNRNPAB*	Heterogeneous nuclear ribonucleoprotein A/B
Q7LFX5	*CHST15*	Carbohydrate sulfotransferase 15
Q0MQG6	*NDUFS3*	NADH dehydrogenase [ubiquinone] iron-sulfur protein 3, mitochondrial
Q32P51	*HNRNPA1L2*	Heterogeneous nuclear ribonucleoprotein A1-like 2
P02461	*COL3A1*	Collagen alpha-1(III) chain

Q9H4G4	*GLIPR2*	Golgi-associated plant pathogenesis-related protein 1
P57113	*Gstz1*	Maleylacetoacetate isomerase
P51968		Heterogeneous nuclear ribonucleoprotein A3 homolog 1
P21675	*TAF1*	Transcription initiation factor TFIID subunit 1
Q9UGM3	*DMBT1*	Deleted in malignant brain tumors 1 protein
Q8C3F2	*Fam120c*	Constitutive coactivator of PPAR-gamma-like protein 2
P92176	*ACT2*	Actin-2
Q7T3S3	*chst11*	Carbohydrate sulfotransferase 11
O88947	*F10*	Coagulation factor X
G4WAW9	*SelM*	Selenoprotein M
Q29RT0	*RBMX*	RNA-binding motif protein, X chromosome
Q5EA36	*RBM14*	RNA-binding protein 14
P61210	*ARF1*	ADP-ribosylation factor 1
Q6CQ22	*COF1*	Cofilin
P31483	*TIA1*	Nucleolysin TIA-1 isoform p40
P08121	*Col3a1*	Collagen alpha-1(III) chain
B4F6K2	*hagh*	Hydroxyacylglutathione hydrolase, mitochondrial
P14315	*CAPZB*	F-actin-capping protein subunit beta isoforms 1 and 2
Q27294	*caz*	RNA-binding protein cabeza
P06865	*HEXA*	Beta-hexosaminidase subunit alpha
Q9UVX4	*ACT1*	Actin
O18373	*SelD*	Selenide, water dikinase
P02453	*COL1A1*	Collagen alpha-1(I) chain
Q7SZR5	*sumo1*	Small ubiquitin-related modifier 1
Q0MQI4	*NDUFV1*	NADH dehydrogenase [ubiquinone] flavoprotein 1, mitochondrial
P56518	*HDAC1*	Histone deacetylase 1

O76554	*des-2*	Acetylcholine receptor subunit alpha-type des-2
Q91W90	*Txndc5*	Thioredoxin domain-containing protein 5
Q8MPM1	*gelsolin*	Gelsolin-like protein 2
P35748	*MYH11*	Myosin-11
Q9VXG4	*AnxB11*	Annexin B11
Q9BXJ5	*C1QTNF2*	Complement C1q tumor necrosis factor-related protein 2
P05496	*ATP5G1*	ATP synthase F(0) complex subunit C1, mitochondrial
Q9JID1	*Pdcd4*	Programmed cell death protein 4
P12716		Actin, cytoplasmic
P17126		Actin, non-muscle 6.2
Q96K80	*ZC3H10*	Zinc finger CCCH domain-containing protein 10
Q8JG30	*SULT1B1*	Sulfotransferase family cytosolic 1B member 1
Q9VT65	*CalpB*	Calpain-B
Q8VCC9	*Spon1*	Spondin-1
Q5XJR6	*ormdl3*	ORM1-like protein 3
Q08473	*sqd*	RNA-binding protein squid
Q9JK42	*Pdk2*	[Pyruvate dehydrogenase (acetyl-transferring)] kinase isozyme 2, mitochondrial
Q5RBP1	*NPNT*	Nephronectin
Q148N0	*OGDH*	2-oxoglutarate dehydrogenase, mitochondrial
P53464		Actin, cytoskeletal
P02576	*ARDA*	Actin, plasmodial isoform
P02578		Actin-1
Q00215		Actin, cytoplasmic
P29361	*YWHAZ*	14-3-3 protein zeta/delta
P23818	*Gria1*	Glutamate receptor 1
Q9U505		ATP synthase lipid-binding protein, mitochondrial

Q4UMH6	*RF_0381*	Putative ankyrin repeat protein RF_0381
Q15417	*CNN3*	Calponin-3
Q14789	*GOLGB1*	Golgin subfamily B member 1
P13941	*Col3a1*	Collagen alpha-1(III) chain
Q15291	*RBBP5*	Retinoblastoma-binding protein 5
P91791		Peptidyl-prolyl cis-trans isomerase
Q00174	*LanA*	Laminin subunit alpha
Q7SYH5	*slc5a8*	Sodium-coupled monocarboxylate transporter 1
P18484	*Ap2a2*	AP-2 complex subunit alpha-2
Q9Z0M6	*Cd97*	CD97 antigen
O54879	*Hmgb3*	High mobility group protein B3
Q9XSJ0	*UCHL5*	Ubiquitin carboxyl-terminal hydrolase isozyme L5
Q9DBB9	*Cpn2*	Carboxypeptidase N subunit 2
P11833		Tubulin beta chain
P30883	*tubb4*	Tubulin beta-4 chain
P36221	*tubb1*	Tubulin beta-1 chain
P30156	*TUBB5*	Tubulin beta-5 chain
Q29RV1	*PDIA4*	Protein disulfide-isomerase A4
O08807	*Prdx4*	Peroxiredoxin-4
O45040	*GBETA1*	Guanine nucleotide-binding protein G(I)/G(S)/G(T) subunit beta-1
O61213	*bli-3*	Dual oxidase 1
Q5RBR8	*EIF4H*	Eukaryotic translation initiation factor 4H
P02640	*VIL1*	Villin-1
A1A4L0	*SNX4*	Sorting nexin-4
Q93129		Actin, cytoplasmic
Q02081	*LCC4*	Laccase-4

P35285	*Rab22a*	Ras-related protein Rab-22A
A2ARA8	*Itga8*	Integrin alpha-8
Q3MHL3	*RBBP4*	Histone-binding protein RBBP4
P60710	*Actb*	Actin, cytoplasmic 1
Q3B8G7	*ier3ip1*	Immediate early response 3-interacting protein 1
Q80UG5	*43717*	Septin-9
Q9JMH7	*Neu3*	Sialidase-3
Q7SXW6	*actr2a*	Actin-related protein 2-A
Q5R7A8	*MYCBP*	C-Myc-binding protein
Q8N8S7	*ENAH*	Protein enabled homolog
Q6PIL6	*KCNIP4*	Kv channel-interacting protein 4
Q7TMK9	*Syncrip*	Heterogeneous nuclear ribonucleoprotein Q
P61979	*Hnrnpk*	Heterogeneous nuclear ribonucleoprotein K
O00338	*SULT1C2*	Sulfotransferase 1C2
P52845	*Ste2*	Estrogen sulfotransferase, isoform 2
P61978	*HNRNPK*	Heterogeneous nuclear ribonucleoprotein K
P49739	*mmcm3*	Maternal DNA replication licensing factor mcm3
P21956	*Mfge8*	Lactadherin
Q9D0R2	*Tars*	Threonine--tRNA ligase, cytoplasmic
Q55E72	*stlA*	Probable polyketide synthase 1
O93428	*ctsd*	Cathepsin D
P54654	*cap*	Adenylyl cyclase-associated protein
O97490	*Mfi2*	Melanotransferrin
Q92113	*CYP17A1*	Steroid 17-alpha-hydroxylase/17,20 lyase
Q9Y222	*DMTF1*	Cyclin-D-binding Myb-like transcription factor 1
P0CY46	*Egfr*	Epidermal growth factor receptor

Q04609	*FOLH1*	Glutamate carboxypeptidase 2
P35659	*DEK*	Protein DEK
Q27U48		Tubulin beta-1 chain
P53708	*ITGA8*	Integrin alpha-8
Q9ERS2	*Ndufa13*	NADH dehydrogenase [ubiquinone] 1 alpha subcomplex subunit 13
P21279	*Gnaq*	Guanine nucleotide-binding protein G(q) subunit alpha
Q3ZBK6	*LSM4*	U6 snRNA-associated Sm-like protein LSm4
P98068	*SPAN*	Protein SpAN
Q2KJC9	*ALDH7A1*	Alpha-aminoadipic semialdehyde dehydrogenase
Q6AQK1	*gatA*	Glutamyl-tRNA(Gln) amidotransferase subunit A
Q6UR05	*ABCC1*	Multidrug resistance-associated protein 1
F1MS15	*PLEKHA8*	Pleckstrin homology domain-containing family A member 8
Q9EQX4	*Aif1l*	Allograft inflammatory factor 1-like
Q9BDK2	*AIF1*	Allograft inflammatory factor 1
P35479		Leukocyte cysteine proteinase inhibitor 1
P46721	*SLCO1A2*	Solute carrier organic anion transporter family member 1A2
Q9HAP2	*MLXIP*	MLX-interacting protein
Q17770	*pdi-2*	Protein disulfide-isomerase 2
O95793	*STAU1*	Double-stranded RNA-binding protein Staufen homolog 1
P09670	*SOD1*	Superoxide dismutase [Cu-Zn]
P46531	*NOTCH1*	Neurogenic locus notch homolog protein 1
P26652	*TIMP3*	Metalloproteinase inhibitor 3
Q25117		ATP synthase subunit beta, mitochondrial
Q8HXY4	*BCKDHA*	2-oxoisovalerate dehydrogenase subunit alpha, mitochondrial
P49432	*Pdhb*	Pyruvate dehydrogenase E1 component subunit beta, mitochondrial
P49137	*MAPKAPK2*	MAP kinase-activated protein kinase 2

Q29471	*ANXA13*	Annexin A13
Q5R5I4	*UBE2L3*	Ubiquitin-conjugating enzyme E2 L3
Q6AZT4	*brms1l*	Breast cancer metastasis-suppressor 1-like protein
A9YUB5	*URM1*	Ubiquitin-related modifier 1
O97580	*SUCLA2*	Succinyl-CoA ligase [ADP-forming] subunit beta, mitochondrial (Fragment)
Q91731	*sox11-a*	Transcription factor Sox-11-A
Q9D8Z1	*Ascc1*	Activating signal cointegrator 1 complex subunit 1
P06623	*CNP*	2',3'-cyclic-nucleotide 3'-phosphodiesterase
Q03348	*Ptpra*	Receptor-type tyrosine-protein phosphatase alpha
Q26613	*EMAP*	77 kDa echinoderm microtubule-associated protein
P18241		Tubulin beta-1 chain
P04975	*CLTB*	Clathrin light chain B
Q01433	*AMPD2*	AMP deaminase 2
P61315	*Gal3st3*	Galactose-3-O-sulfotransferase 3
P32882		Tubulin beta-2 chain
Q5ZMW5	*ARHGAP26*	Rho GTPase-activating protein 26
Q5ZKH0	*GTF2H5*	General transcription factor IIH subunit 5
Q91Y81	*43710*	Septin-2
Q6QBQ4	*Plscr3*	Phospholipid scramblase 3
Q9NYQ6	*CELSR1*	Cadherin EGF LAG seven-pass G-type receptor 1
A2VDP4	*ZNF567*	Zinc finger protein 567
Q8BUP8	*Fam43a*	Protein FAM43A
Q5E9C0	*RSU1*	Ras suppressor protein 1
Q8BL97	*Srsf7*	Serine/arginine-rich splicing factor 7
Q3T106	*SRSF7*	Serine/arginine-rich splicing factor 7
Q8N6F8	*WBSCR27*	Williams-Beuren syndrome chromosomal region 27 protein

Q13685	*AAMP*	Angio-associated migratory cell protein
Q28178	*THBS1*	Thrombospondin-1
P62958	*HINT1*	Histidine triad nucleotide-binding protein 1
Q66IF1	*reep6*	Receptor expression-enhancing protein 6
Q9D0K0	*Tbc1d7*	TBC1 domain family member 7
C3YWU0	*BRAFLDRAFT_56888*	Alpha-L-fucosidase
Q29496	*CYP3A24*	Cytochrome P450 3A24
P56101	*dnajc5*	DnaJ homolog subfamily C member 5
Q9BXJ3	*C1QTNF4*	Complement C1q tumor necrosis factor-related protein 4
P08537	*tuba*	Tubulin alpha chain
P37879	*KARS*	Lysine--tRNA ligase
Q9EQS0	*Taldo1*	Transaldolase
Q14185	*DOCK1*	Dedicator of cytokinesis protein 1
Q921Y4	*Mfsd5*	Molybdate-anion transporter
Q9H8Y8	*GORASP2*	Golgi reassembly-stacking protein 2
Q4R5B3	*TUBB2A*	Tubulin beta-2A chain
Q5ZJ41	*43529*	E3 ubiquitin-protein ligase MARCH5
Q66HG5	*Tm9sf2*	Transmembrane 9 superfamily member 2
P27216	*ANXA13*	Annexin A13
P17152	*TMEM11*	Transmembrane protein 11, mitochondrial
P41216	*Acsl1*	Long-chain-fatty-acid--CoA ligase 1
O55043	*Arhgef7*	Rho guanine nucleotide exchange factor 7
Q3SZ47	*MRPL33*	39S ribosomal protein L33, mitochondrial
Q8TXI4	*rad50*	DNA double-strand break repair Rad50 ATPase
P34416	*F42H10.3*	LIM and SH3 domain protein F42H10.3
Q7JQD3	*AM*	Gelsolin-like protein 1

P0C6B8	*Svep1*	Sushi, von Willebrand factor type A, EGF and pentraxin domain-containing protein 1
P82205		Superoxide dismutase [Cu-Zn]
Q90941	*PBRM1*	Protein polybromo-1
Q3SZA9	*MRPL35*	39S ribosomal protein L35, mitochondrial
Q9NDS0	*TPM*	Tropomyosin
P78412	*IRX6*	Iroquois-class homeodomain protein IRX-6
Q9GV77	*ECM3*	Extracellular matrix protein 3
P47708	*Rph3a*	Rabphilin-3A
Q5R7G6	*NPEPL1*	Probable aminopeptidase NPEPL1
O60494	*CUBN*	Cubilin
B0BLT0	*znf593*	Zinc finger protein 593
Q9BUN8	*DERL1*	Derlin-1
Q5RCI4	*CCDC47*	Coiled-coil domain-containing protein 47
Q9Z1J3	*Nfs1*	Cysteine desulfurase, mitochondrial
P80928	*MIF*	Macrophage migration inhibitory factor
Q8R322	*Gle1*	Nucleoporin GLE1
Q64560	*Tpp2*	Tripeptidyl-peptidase 2
A0JPJ7	*Ola1*	Obg-like ATPase 1
Q2YDE7	*ZBTB8OS*	Protein archease
P26446	*PARP1*	Poly [ADP-ribose] polymerase 1
Q8WWY8	*LIPH*	Lipase member H
Q1LZ71	*FAIM2*	Protein lifeguard 2
Q32L53	*GRINA*	Protein lifeguard 1
P10768	*ESD*	S-formylglutathione hydrolase
Q58CS8	*GNPTG*	N-acetylglucosamine-1-phosphotransferase subunit gamma
Q69ZX6	*Morc2a*	MORC family CW-type zinc finger protein 2A

Q0P4P2	*fibcd1*	Fibrinogen C domain-containing protein 1
P31696	*AGRN*	Agrin
Q99PL5	*Rrbp1*	Ribosome-binding protein 1
P18075	*BMP7*	Bone morphogenetic protein 7
P84103	*SRSF3*	Serine/arginine-rich splicing factor 3
Q5G267	*PRSS12*	Neurotrypsin
Q6IRI9	*Fmo2*	Dimethylaniline monooxygenase [N-oxide-forming] 2
Q8AWW5	*CRIM1*	Cysteine-rich motor neuron 1 protein
Q5XGB9	*lap3*	Cytosol aminopeptidase
Q6UXV4	*APOOL*	MICOS complex subunit MIC27
O08738	*Casp6*	Caspase-6
O54941	*Smarce1*	SWI/SNF-related matrix-associated actin-dependent regulator of chromatin subfamily E member 1
Q9VFC8	*GlyS*	Glycogen [starch] synthase
P41367	*ACADM*	Medium-chain specific acyl-CoA dehydrogenase, mitochondrial
Q2KHU0	*PSPH*	Phosphoserine phosphatase
P18288		Tubulin alpha chain, testis-specific
Q91WD5	*Ndufs2*	NADH dehydrogenase [ubiquinone] iron-sulfur protein 2, mitochondrial
Q8WXR4	*MYO3B*	Myosin-IIIb
Q3SZB4	*ACADM*	Medium-chain specific acyl-CoA dehydrogenase, mitochondrial
O55044	*G6PD*	Glucose-6-phosphate 1-dehydrogenase
P05370	*G6pdx*	Glucose-6-phosphate 1-dehydrogenase
O93327	*H2AFY*	Core histone macro-H2A.1
Q5R930	*MSRB3*	Methionine-R-sulfoxide reductase B3
P52297	*kpnb1*	Importin subunit beta
P41732	*TSPAN7*	Tetraspanin-7
Q90330	*FGFR4*	Fibroblast growth factor receptor 4

Q06645	*Atp5g1*	ATP synthase F(0) complex subunit C1, mitochondrial
P13280	*GYG1*	Glycogenin-1
Q58DT4	*PYCR1*	Pyrroline-5-carboxylate reductase 1, mitochondrial
Q9UGT4	*SUSD2*	Sushi domain-containing protein 2
Q05519	*SRSF11*	Serine/arginine-rich splicing factor 11
P42683	*LCK*	Proto-oncogene tyrosine-protein kinase LCK
Q9BWD1	*ACAT2*	Acetyl-CoA acetyltransferase, cytosolic
O95394	*PGM3*	Phosphoacetylglucosamine mutase
P47866	*Crhr2*	Corticotropin-releasing factor receptor 2
Q7SXG4	*uba2*	SUMO-activating enzyme subunit 2
Q3V384	*Lace1*	Lactation elevated protein 1
Q504A5	*tpmt*	Probable thiopurine S-methyltransferase
Q32PH2	*TMEM143*	Transmembrane protein 143
Q54GX7	*act10*	Actin-10
Q9XSC3	*WDR44*	WD repeat-containing protein 44
Q9DCS9	*Ndufb10*	NADH dehydrogenase [ubiquinone] 1 beta subcomplex subunit 10
Q29492	*G6PD*	Glucose-6-phosphate 1-dehydrogenase
Q9IBG7	*kcp*	Kielin/chordin-like protein
Q0P5A6	*PSMD5*	26S proteasome non-ATPase regulatory subunit 5
Q2TBL6	*TALDO1*	Transaldolase
Q32P85	*DYNLRB2*	Dynein light chain roadblock-type 2
Q0MQD0	*NDUFB3*	NADH dehydrogenase [ubiquinone] 1 beta subcomplex subunit 3
Q95SX7	*RTase*	Probable RNA-directed DNA polymerase from transposon BS
P32192		Elongation factor 1-delta
Q9VE46	*CG7708*	High-affinity choline transporter 1
Q8BY02	*Nkrf*	NF-kappa-B-repressing factor

Q5RD03	*ARMC6*	Armadillo repeat-containing protein 6
A1A5V9	*elp5*	Elongator complex protein 5
Q9CQM5	*Txndc17*	Thioredoxin domain-containing protein 17
Q2KI56	*COPS7B*	COP9 signalosome complex subunit 7b
Q5VWX1	*KHDRBS2*	KH domain-containing, RNA-binding, signal transduction-associated protein 2
P42346	*Mtor*	Serine/threonine-protein kinase mTOR
P22083	*FUT4*	Alpha-(1,3)-fucosyltransferase 4
P83851	*NSNH*	Inosine-uridine preferring nucleoside hydrolase
Q02368	*NDUFB7*	NADH dehydrogenase [ubiquinone] 1 beta subcomplex subunit 7
Q99MR0	*Actl6b*	Actin-like protein 6B
P50464	*unc-97*	LIM domain-containing protein unc-97
Q5F408	*CARS*	Cysteine--tRNA ligase, cytoplasmic
Q6PDQ2	*Chd4*	Chromodomain-helicase-DNA-binding protein 4
Q2VLH6	*Cd163*	Scavenger receptor cysteine-rich type 1 protein M130
Q9NUV7	*SPTLC3*	Serine palmitoyltransferase 3
Q5ZKF4	*HMG20A*	High mobility group protein 20A
O08651	*Phgdh*	D-3-phosphoglycerate dehydrogenase
P26885	*FKBP2*	Peptidyl-prolyl cis-trans isomerase FKBP2
A2TLM1	*RPIA*	Ribose-5-phosphate isomerase
Q9SHF2	*AGO3*	Protein argonaute 3
P98073	*TMPRSS15*	Enteropeptidase
Q8N3X1	*FNBP4*	Formin-binding protein 4
Q64350	*Eif2b5*	Translation initiation factor eIF-2B subunit epsilon
Q9QYP0	*Megf8*	Multiple epidermal growth factor-like domains protein 8
Q63829	*Commd3*	COMM domain-containing protein 3
Q9TU53	*CUBN*	Cubilin

Q9N126	*RDH8*	Retinol dehydrogenase 8
Q5F495	*MOB4*	MOB-like protein phocein
Q2KJH9	*ALDH9A1*	4-trimethylaminobutyraldehyde dehydrogenase
Q802W2	*aldh9a1b*	Aldehyde dehydrogenase family 9 member A1-B
Q5R8A4	*ALDH9A1*	4-trimethylaminobutyraldehyde dehydrogenase
A1A5S1	*Prpf6*	Pre-mRNA-processing factor 6
O14744	*PRMT5*	Protein arginine N-methyltransferase 5
Q15435	*PPP1R7*	Protein phosphatase 1 regulatory subunit 7
Q6ZMN7	*PDZRN4*	PDZ domain-containing RING finger protein 4
Q22235	*enpl-1*	Endoplasmin homolog
Q5ZKN2	*PGRMC1*	Membrane-associated progesterone receptor component 1
Q8CGE9	*Rgs12*	Regulator of G-protein signaling 12
Q96K76	*USP47*	Ubiquitin carboxyl-terminal hydrolase 47
Q6ZMW2	*ZNF782*	Zinc finger protein 782
Q28852	*ATP5L*	ATP synthase subunit g, mitochondrial
Q8QZY9	*Sf3b4*	Splicing factor 3B subunit 4
Q6PBF7	*emc4*	ER membrane protein complex subunit 4
P26262	*Klkb1*	Plasma kallikrein
Q5U4Y8	*seh1l*	Nucleoporin seh1
O75844	*ZMPSTE24*	CAAX prenyl protease 1 homolog
P31695	*Notch4*	Neurogenic locus notch homolog protein 4
A0JMV4	*rbm5-a*	RNA-binding protein 5-A
Q10567	*AP1B1*	AP-1 complex subunit beta-1
Q96EW2	*HSPBAP1*	HSPB1-associated protein 1
Q6DKP5	*WDR13*	WD repeat-containing protein 13
P97694	*Cyth1*	Cytohesin-1

Q14697	*GANAB*	Neutral alpha-glucosidase AB
Q94502	*modA*	Neutral alpha-glucosidase AB
Q9DB27	*Mcts1*	Malignant T-cell-amplified sequence 1
Q9H7F0	*ATP13A3*	Probable cation-transporting ATPase 13A3
Q9DBC0	*Selo*	Selenoprotein O
A5A6L0	*PDHA1*	Pyruvate dehydrogenase E1 component subunit alpha, somatic form, mitochondrial
P52899	*T05H10.6*	Probable pyruvate dehydrogenase E1 component subunit alpha, mitochondrial
F7J220	*SRCR1*	Scavenger receptor cysteine-rich domain superfamily protein
Q92J02	*grxC1*	Glutaredoxin-1
Q8N9C0	*IGSF22*	Immunoglobulin superfamily member 22
Q10475	*tif471*	Eukaryotic translation initiation factor 4 gamma
Q9D0L8	*Rnmt*	mRNA cap guanine-N7 methyltransferase
Q24524	*sn*	Protein singed
P11182	*DBT*	Lipoamide acyltransferase component of branched-chain alpha-keto acid dehydrogenase complex, mitochondrial
P53395	*Dbt*	Lipoamide acyltransferase component of branched-chain alpha-keto acid dehydrogenase complex, mitochondrial
P50426	*GNS*	N-acetylglucosamine-6-sulfatase
Q00277	*GPX1*	Glutathione peroxidase
P07898	*ACAN*	Aggrecan core protein
Q6IQ97	*rbm4.1*	RNA-binding protein 4.1
C3ZDX5	*BRAFLDRAFT_275690*	Ubiquitin-fold modifier-conjugating enzyme 1
Q148G7	*DCTN6*	Dynactin subunit 6
P70193	*Lrig1*	Leucine-rich repeats and immunoglobulin-like domains protein 1
Q9WU63	*Hebp2*	Heme-binding protein 2
Q6P2B1	*Tnpo3*	Transportin-3
Q5R941	*FKBP14*	Peptidyl-prolyl cis-trans isomerase FKBP14
O61577	*KATNA1*	Katanin p60 ATPase-containing subunit A1

Q6AZA0	*acat1*	Acetyl-CoA acetyltransferase, mitochondrial
Q8TFG4	*SPAPB18E9.04c*	Uncharacterized protein PB18E9.04c
Q16891	*IMMT*	MICOS complex subunit MIC60
Q5ZLD7	*VPS53*	Vacuolar protein sorting-associated protein 53 homolog
E1BB03	*ZRANB3*	DNA annealing helicase and endonuclease ZRANB3
Q9YHT1	*SDHA*	Succinate dehydrogenase [ubiquinone] flavoprotein subunit, mitochondrial
Q8N6L1	*KRTCAP2*	Keratinocyte-associated protein 2
Q5Y4N8	*Adgre1*	Adhesion G protein-coupled receptor E2
Q6YP21	*CCBL2*	Kynurenine--oxoglutarate transaminase 3
P54363	*Aprt*	Adenine phosphoribosyltransferase
O14562	*UBFD1*	Ubiquitin domain-containing protein UBFD1
Q3UUJ4	*Strada*	STE20-related kinase adapter protein alpha
Q9CWK8	*Snx2*	Sorting nexin-2
Q5H8C1	*FREM1*	FRAS1-related extracellular matrix protein 1
Q9D8I1	*Mzb1*	Marginal zone B- and B1-cell-specific protein
F1QEG2	*klhl41b*	Kelch-like protein 41b
Q9Y5X2	*SNX8*	Sorting nexin-8
Q0V9J0	*tmem69*	Transmembrane protein 69
Q27128		Bifunctional 3'-phosphoadenosine 5'-phosphosulfate synthase
Q0U2T3	*MDV1*	Mitochondrial division protein 1
O43678	*NDUFA2*	NADH dehydrogenase [ubiquinone] 1 alpha subcomplex subunit 2
Q29073	*PTGR1*	Prostaglandin reductase 1
Q6PBY7	*trappc13*	Trafficking protein particle complex subunit 13
Q12769	*NUP160*	Nuclear pore complex protein Nup160
Q8BMF4	*Dlat*	Dihydrolipoyllysine-residue acetyltransferase component of pyruvate dehydrogenase complex, mitochondrial
Q61122	*Nab1*	NGFI-A-binding protein 1

P54357	*Mlc-c*	Myosin-2 essential light chain
Q6GM78	*asrgl1*	Isoaspartyl peptidase/L-asparaginase
Q5XPI3	*Rnf123*	E3 ubiquitin-protein ligase RNF123
D4PHA7	*Wscd2*	WSC domain-containing protein 2
Q8BJ25	*Thap3*	THAP domain-containing protein 3
Q7TMR0	*Prcp*	Lysosomal Pro-X carboxypeptidase
B4H303	*GL13432*	NFU1 iron-sulfur cluster scaffold homolog, mitochondrial
Q01414	*ERG*	Transcriptional regulator ERG homolog (Fragment)
O61585	*KATNB1*	Katanin p80 WD40 repeat-containing subunit B1
Q9HD67	*MYO10*	Unconventional myosin-X
Q8R081	*Hnrnpl*	Heterogeneous nuclear ribonucleoprotein L
Q64270	*Eif2b1*	Translation initiation factor eIF-2B subunit alpha
Q0IIF2	*EIF2B1*	Translation initiation factor eIF-2B subunit alpha
Q6NWC6	*cpsf6*	Cleavage and polyadenylation specificity factor subunit 6
Q9Y2V7	*COG6*	Conserved oligomeric Golgi complex subunit 6
P98153	*DGCR2*	Integral membrane protein DGCR2/IDD
Q8NBN3	*TMEM87A*	Transmembrane protein 87A
Q803I2	*ergic3*	Endoplasmic reticulum-Golgi intermediate compartment protein 3
Q9QZ88	*Vps29*	Vacuolar protein sorting-associated protein 29
P46872	*KRP85*	Kinesin-II 85 kDa subunit
Q8WVK2	*SNRNP27*	U4/U6.U5 small nuclear ribonucleoprotein 27 kDa protein
Q2TBV1	*RFC3*	Replication factor C subunit 3
Q9TZH6	*pcbd-1*	Putative pterin-4-alpha-carbinolamine dehydratase
Q8WW62	*TMED6*	Transmembrane emp24 domain-containing protein 6
Q5RBA8	*PRPSAP2*	Phosphoribosyl pyrophosphate synthase-associated protein 2
Q5MJS3	*Fam20c*	Extracellular serine/threonine protein kinase FAM20C

Q13797	*ITGA9*	Integrin alpha-9
Q6UWH6	*TEX261*	Protein TEX261
Q7RTY0	*SLC16A13*	Monocarboxylate transporter 13
O55166	*Vps52*	Vacuolar protein sorting-associated protein 52 homolog
Q5TJF0	*VPS52*	Vacuolar protein sorting-associated protein 52 homolog
Q8N1B4	*VPS52*	Vacuolar protein sorting-associated protein 52 homolog
Q93725	*ned-8*	NEDD8
Q5G270	*PRSS12*	Neurotrypsin
P25155	*F10*	Coagulation factor X
P10079	*EGF1*	Fibropellin-1
O35077	*Gpd1*	Glycerol-3-phosphate dehydrogenase [NAD(+)], cytoplasmic
Q6Q473	*Clca4a*	Calcium-activated chloride channel regulator 4A
Q63850	*Nup62*	Nuclear pore glycoprotein p62
Q9BVL2	*NUPL1*	Nucleoporin p58/p45
P91660	*l(2)03659*	Probable multidrug resistance-associated protein lethal(2)03659
O15439	*ABCC4*	Multidrug resistance-associated protein 4
Q8CHI9	*Chst15*	Carbohydrate sulfotransferase 15
Q8K1M6	*Dnm1l*	Dynamin-1-like protein
Q969T3	*SNX21*	Sorting nexin-21
Q9Z186	*G6pc2*	Glucose-6-phosphatase 2
P38659	*Pdia4*	Protein disulfide-isomerase A4
Q8NBX0	*SCCPDH*	Saccharopine dehydrogenase-like oxidoreductase
Q60756	*Tcf15*	Transcription factor 15
O57478	*fech*	Ferrochelatase, mitochondrial
Q91619	*nodal*	Nodal homolog
Q9UPU7	*TBC1D2B*	TBC1 domain family member 2B

Q7TNU6	*Znf250*	Zinc finger protein 250
Q0MVN8	*CRYZ*	Quinone oxidoreductase
Q86KD0	*DDB_G0274169*	BolA-like protein DDB_G0274169
Q55EX9	*DDB_G0268948*	Putative methyltransferase DDB_G0268948
O93277	*WDR1*	WD repeat-containing protein 1
Q9JHL4	*Dbnl*	Drebrin-like protein
Q5ZLP8	*IGF2BP3*	Insulin-like growth factor 2 mRNA-binding protein 3
Q8BGG7	*Ubash3b*	Ubiquitin-associated and SH3 domain-containing protein B
Q5F4A9	*ARL6IP4*	ADP-ribosylation factor-like protein 6-interacting protein 4
Q8BUL6	*Plekha1*	Pleckstrin homology domain-containing family A member 1
O57590	*surf4*	Surfeit locus protein 4
P12270	*TPR*	Nucleoprotein TPR
Q8R242	*Ctbs*	Di-N-acetylchitobiase
Q9Y6G5	*COMMD10*	COMM domain-containing protein 10
O00370		LINE-1 retrotransposable element ORF2 protein
Q9YGD2	*SUCLG1*	Succinyl-CoA ligase subunit alpha, mitochondrial (Fragment)
Q9WUM5	*Suclg1*	Succinyl-CoA ligase [ADP/GDP-forming] subunit alpha, mitochondrial
P33731	*SRP72*	Signal recognition particle subunit SRP72
Q67UP9	*Os06g0651600*	Probable protein phosphatase 2C 58
Q14AT5	*Ano7*	Anoctamin-7
Q6MG48	*Prrc2a*	Protein PRRC2A
Q2TBH5	*UBXN8*	UBX domain-containing protein 8
Q90687	*PTPN11*	Tyrosine-protein phosphatase non-receptor type 11
Q28J34	*erlin2*	Erlin-2
Q06805	*TIE1*	Tyrosine-protein kinase receptor Tie-1
Q9ULZ9	*MMP17*	Matrix metalloproteinase-17

Q80SU7	*Gvin1*	Interferon-induced very large GTPase 1
A7SMW7	*v1g172254*	L-2-hydroxyglutarate dehydrogenase, mitochondrial
P17276	*Hn*	Protein henna
Q02380	*NDUFB5*	NADH dehydrogenase [ubiquinone] 1 beta subcomplex subunit 5, mitochondrial
O75521	*ECI2*	Enoyl-CoA delta isomerase 2, mitochondrial
Q80UJ7	*Rab3gap1*	Rab3 GTPase-activating protein catalytic subunit
Q9UJM8	*HAO1*	Hydroxyacid oxidase 1
Q64244	*Cd38*	ADP-ribosyl cyclase/cyclic ADP-ribose hydrolase 1
Q9UK45	*LSM7*	U6 snRNA-associated Sm-like protein LSm7
Q9DCD2	*Xab2*	Pre-mRNA-splicing factor SYF1
Q24K02	*IDE*	Insulin-degrading enzyme
Q25378	*PKC1*	Protein kinase C
Q5REY7	*UBXN7*	UBX domain-containing protein 7
Q96DM3	*C18orf8*	Uncharacterized protein C18orf8
Q8N539	*FIBCD1*	Fibrinogen C domain-containing protein 1
Q9BUN5	*CCDC28B*	Coiled-coil domain-containing protein 28B
Q6NS19	*ergic1*	Endoplasmic reticulum-Golgi intermediate compartment protein 1
O93257	*XRCC6*	X-ray repair cross-complementing protein 5
Q14008	*CKAP5*	Cytoskeleton-associated protein 5
Q6IQU7	*ints12*	Integrator complex subunit 12
Q9CRC6		UPF0693 protein C10orf32 homolog
Q7TQC7	*Gpatch2*	G patch domain-containing protein 2
Q6P4S8	*Ints1*	Integrator complex subunit 1
A4IF63	*TRIM2*	Tripartite motif-containing protein 2
Q9NTN3	*SLC35D1*	UDP-glucuronic acid/UDP-N-acetylgalactosamine transporter
Q3TLI0	*Trappc10*	Trafficking protein particle complex subunit 10

P52430	*Pon1*	Serum paraoxonase/arylesterase 1
Q4SBY6	*hnmt*	Histamine N-methyltransferase
A2CEI6	*piwil2*	Piwi-like protein 2
A6QLU1	*GPD2*	Glycerol-3-phosphate dehydrogenase, mitochondrial
Q497B0	*Nit2*	Omega-amidase NIT2
Q91XL3	*Uxs1*	UDP-glucuronic acid decarboxylase 1
Q8NBP0	*TTC13*	Tetratricopeptide repeat protein 13
Q98931	*LRP8*	Low-density lipoprotein receptor-related protein 8
Q3ZBL5	*PTRH2*	Peptidyl-tRNA hydrolase 2, mitochondrial
Q0MQB0	*NDUFA8*	NADH dehydrogenase [ubiquinone] 1 alpha subcomplex subunit 8
P11029	*ACAC*	Acetyl-CoA carboxylase
Q5U2Y6	*Tfip11*	Tuftelin-interacting protein 11
Q9NRL2	*BAZ1A*	Bromodomain adjacent to zinc finger domain protein 1A
A8E0R9	*grip2*	Glutamate receptor-interacting protein 2
Q06AB3	*UCHL3*	Ubiquitin carboxyl-terminal hydrolase isozyme L3
O75323	*GBAS*	Protein NipSnap homolog 2
Q66J54	*slc22a6-a*	Solute carrier family 22 member 6-A
Q9BUE0	*MED18*	Mediator of RNA polymerase II transcription subunit 18
Q5R440	*CANX*	Calnexin
Q96PE7	*MCEE*	Methylmalonyl-CoA epimerase, mitochondrial
Q9WTV0	*Preb*	Prolactin regulatory element-binding protein
Q503L9	*nxn*	Nucleoredoxin
Q6DKJ4	*NXN*	Nucleoredoxin
P18519	*NGFR*	Tumor necrosis factor receptor superfamily member 16
P70106	*GUCY2C*	Heat-stable enterotoxin receptor
P63155	*Crnkl1*	Crooked neck-like protein 1

Q9BZJ0	*CRNKL1*	Crooked neck-like protein 1
Q9HBH1	*PDF*	Peptide deformylase, mitochondrial
Q24488	*Ror*	Tyrosine-protein kinase transmembrane receptor Ror
Q8BRH0	*Tmtc3*	Transmembrane and TPR repeat-containing protein 3
Q6ZXV5	*TMTC3*	Transmembrane and TPR repeat-containing protein 3
Q96IG2	*FBXL20*	F-box/LRR-repeat protein 20
Q5R6R5	*CMAS*	N-acylneuraminate cytidylyltransferase
Q8BUB6	*Ust*	Uronyl 2-sulfotransferase
Q5RF24	*WDR13*	WD repeat-containing protein 13
A7Z035	*CLINT1*	Clathrin interactor 1
P83299	*ca1*	Carbonic anhydrase 1
O60733	*PLA2G6*	85/88 kDa calcium-independent phospholipase A2
A4FV72	*PPIE*	Peptidyl-prolyl cis-trans isomerase E
Q99LB6	*Mat2b*	Methionine adenosyltransferase 2 subunit beta
Q6P4X5	*cpt2*	Carnitine O-palmitoyltransferase 2, mitochondrial
Q9R112	*Sqrdl*	Sulfide:quinone oxidoreductase, mitochondrial
Q5R8G6	*MCM3*	DNA replication licensing factor MCM3
Q3T1L0	*Aldh16a1*	Aldehyde dehydrogenase family 16 member A1
Q9Y6D6	*ARFGEF1*	Brefeldin A-inhibited guanine nucleotide-exchange protein 1
Q6DEB1	*etnppl*	Ethanolamine-phosphate phospho-lyase
Q8CAK3		UPF0515 protein C19orf66 homolog
P20735	*GGT1*	Gamma-glutamyltranspeptidase 1
P51688	*SGSH*	N-sulphoglucosamine sulphohydrolase
Q6P3A8	*Bckdhb*	2-oxoisovalerate dehydrogenase subunit beta, mitochondrial
O55023	*Impa1*	Inositol monophosphatase 1
P54985	*CYPA*	Peptidyl-prolyl cis-trans isomerase

Q5U3K5	*Rabl6*	Rab-like protein 6
Q4H4B6	*scrib*	Protein scribble homolog
C3YEM5	*BRAFLDRAFT_84221*	Elongation factor Ts, mitochondrial
Q803V3	*ppp2r3c*	Serine/threonine-protein phosphatase 2A regulatory subunit B" subunit gamma
Q8HXP3	*SOD2*	Superoxide dismutase [Mn], mitochondrial
Q86TI2	*DPP9*	Dipeptidyl peptidase 9
A6QQU6	*TMEM144*	Transmembrane protein 144
P70170	*Abcc9*	ATP-binding cassette sub-family C member 9
Q6W2J9	*BCOR*	BCL-6 corepressor
Q569T7	*naglt1*	Sodium-dependent glucose transporter 1
P82915	*MRPS16*	28S ribosomal protein S16, mitochondrial
P33124	*Acsl6*	Long-chain-fatty-acid--CoA ligase 6
Q06646	*Atp5g2*	ATP synthase F(0) complex subunit C2, mitochondrial
Q10656	*egl-15*	Myoblast growth factor receptor egl-15
Q4V893	*Gpalpp1*	GPALPP motifs-containing protein 1
Q6PBM1	*glrx5*	Glutaredoxin-related protein 5, mitochondrial
Q5ZLG0	*AACS*	Acetoacetyl-CoA synthetase
Q9QZP2	*Prdm4*	PR domain zinc finger protein 4
Q5RDW1	*MTG2*	Mitochondrial ribosome-associated GTPase 2
Q5ZJM3	*NGLY1*	Peptide-N(4)-(N-acetyl-beta-glucosaminyl)asparagine amidase
P24472	*Gsta4*	Glutathione S-transferase A4
Q16772	*GSTA3*	Glutathione S-transferase A3
Q5DTY9	*Kctd16*	BTB/POZ domain-containing protein KCTD16
F8S0Z7		Snake venom 5'-nucleotidase
Q0VC50	*RTN4IP1*	Reticulon-4-interacting protein 1, mitochondrial
Q8NFH4	*NUP37*	Nucleoporin Nup37

P57080	*Usp25*	Ubiquitin carboxyl-terminal hydrolase 25
Q8CHP6	*Phc3*	Polyhomeotic-like protein 3
P35290	*Rab24*	Ras-related protein Rab-24
Q3T0C2	*HPGD*	15-hydroxyprostaglandin dehydrogenase [NAD(+)]
P35269	*GTF2F1*	General transcription factor IIF subunit 1
Q05913	*TfIIFalpha*	General transcription factor IIF subunit 1
A3KFU9	*Ptchd2*	Patched domain-containing protein 2
P20933	*AGA*	N(4)-(beta-N-acetylglucosaminyl)-L-asparaginase
Q9Z1G4	*Atp6v0a1*	V-type proton ATPase 116 kDa subunit a isoform 1
Q55BM1	*DDB_G0270444*	Probable inactive protein kinase DDB_G0270444
P42166	*TMPO*	Lamina-associated polypeptide 2, isoform alpha
P68401	*PAFAH1B2*	Platelet-activating factor acetylhydrolase IB subunit beta
Q8HXG5	*NDUFB11*	NADH dehydrogenase [ubiquinone] 1 beta subcomplex subunit 11, mitochondrial
Q9DGN1	*stag1*	Cohesin subunit SA-1
Q8N3U4	*STAG2*	Cohesin subunit SA-2
P97519	*Hmgcl*	Hydroxymethylglutaryl-CoA lyase, mitochondrial
Q5R7R6	*COG4*	Conserved oligomeric Golgi complex subunit 4
Q3UX61	*Naa11*	N-alpha-acetyltransferase 11
Q8TCB7	*METTL6*	Methyltransferase-like protein 6
Q148M7	*TAF13*	Transcription initiation factor TFIID subunit 13
A7RNG8	*v1g180167*	Coiled-coil domain-containing protein 22 homolog
Q95RN0	*CG10038*	UPF0528 protein CG10038
Q9WUA2	*Farsb*	Phenylalanine--tRNA ligase beta subunit
Q6TH01	*si:dkey-29f10.1*	Protein C10
A0JN27	*Gtf2h2*	General transcription factor IIH subunit 2
P38117	*ETFB*	Electron transfer flavoprotein subunit beta

P61087	*Ube2k*	Ubiquitin-conjugating enzyme E2 K
Q7ZYD9	*ankrd13c-b*	Ankyrin repeat domain-containing protein 13C-B
Q32KM1	*TUBG2*	Tubulin gamma-2 chain
Q4V9P0	*slc25a26*	S-adenosylmethionine mitochondrial carrier protein
Q96K78	*ADGRG7*	Adhesion G-protein coupled receptor G7
Q5F339	*HCCS*	Cytochrome c-type heme lyase
Q96DB5	*RMDN1*	Regulator of microtubule dynamics protein 1
Q9Z1W9	*Stk39*	STE20/SPS1-related proline-alanine-rich protein kinase
Q6AYJ3	*Spata6l*	Spermatogenesis associated 6-like protein
Q8WU76	*SCFD2*	Sec1 family domain-containing protein 2
Q3UMM4	*Cdk10*	Cyclin-dependent kinase 10
Q2HJD0	*FAF2*	FAS-associated factor 2
Q9BZF1	*OSBPL8*	Oxysterol-binding protein-related protein 8
Q80VC6	*Trnau1ap*	tRNA selenocysteine 1-associated protein 1
P22412	*DPEP1*	Dipeptidase 1
O17482	*tim*	Protein timeless
Q7ZWY2	*pih1d1*	PIH1 domain-containing protein 1
Q92963	*RIT1*	GTP-binding protein Rit1
Q6GMK8	*gmppaa*	Mannose-1-phosphate guanyltransferase alpha-A
Q8AWD2	*mocs3*	Adenylyltransferase and sulfurtransferase MOCS3
Q5RAS0	*ACADS*	Short-chain specific acyl-CoA dehydrogenase, mitochondrial
Q5FVA9	*rae1*	mRNA export factor
Q9H2M9	*RAB3GAP2*	Rab3 GTPase-activating protein non-catalytic subunit
P07949	*RET*	Proto-oncogene tyrosine-protein kinase receptor Ret
Q6NTW6	*nae1*	NEDD8-activating enzyme E1 regulatory subunit
Q8VDI9	*Alg9*	Alpha-1,2-mannosyltransferase ALG9

Q9H6U8	*ALG9*	Alpha-1,2-mannosyltransferase ALG9
A4YGN0	*Msed_1424*	Succinate-semialdehyde dehydrogenase (acetylating)
Q9D8U8	*Snx5*	Sorting nexin-5
P36200	*lhx3*	LIM/homeobox protein Lhx3
A7RK30	*v1g238856*	Quinone oxidoreductase-like protein 2 homolog
Q5ZIJ9	*MIB2*	E3 ubiquitin-protein ligase MIB2
Q5RGJ6	*ccdc53*	WASH complex subunit CCDC53
Q6IR80		UPF0536 protein C12orf66 homolog
O76071	*CIAO1*	Probable cytosolic iron-sulfur protein assembly protein CIAO1
Q9H1I8	*ASCC2*	Activating signal cointegrator 1 complex subunit 2
Q5R839	*SLC41A1*	Solute carrier family 41 member 1
E9PZZ1	*Prdm13*	PR domain zinc finger protein 13
Q9D1I6	*Mrpl14*	39S ribosomal protein L14, mitochondrial
Q3KRG3	*tsr2*	Pre-rRNA-processing protein TSR2 homolog
Q63ZM9	*znf830*	Zinc finger protein 830
Q96GX1	*TCTN2*	Tectonic-2
Q08D86	*GK5*	Putative glycerol kinase 5
O43451	*MGAM*	Maltase-glucoamylase, intestinal
Q6A4L0	*Slc22a13*	Solute carrier family 22 member 13
Q91YP3	*Dera*	Deoxyribose-phosphate aldolase
Q8CI04	*Cog3*	Conserved oligomeric Golgi complex subunit 3
P35428	*Hes1*	Transcription factor HES-1
Q6NVC5	*rhot1a*	Mitochondrial Rho GTPase 1-A
Q8K2Y7	*Mrpl47*	39S ribosomal protein L47, mitochondrial
B1AS42	*Cyb5rl*	NADH-cytochrome b5 reductase-like
Q9JMJ2	*Fbxw4*	F-box/WD repeat-containing protein 4

Q5I0C3	*Mccc1*	Methylcrotonoyl-CoA carboxylase subunit alpha, mitochondrial
P47897	*QARS*	Glutamine--tRNA ligase
Q29RS4	*INO80E*	INO80 complex subunit E
Q6PBU7	*uqcc2*	Ubiquinol-cytochrome-c reductase complex assembly factor 2
Q6DCF2	*mms19*	MMS19 nucleotide excision repair protein homolog
P41236	*PPP1R2*	Protein phosphatase inhibitor 2
Q5PQN7	*Lzic*	Protein LZIC
Q6TGZ5	*hpd*	4-hydroxyphenylpyruvate dioxygenase
Q9NVM9	*ASUN*	Protein asunder homolog
Q8HXD5	*RNF220*	E3 ubiquitin-protein ligase RNF220
A7MBP4	*ift46*	Intraflagellar transport protein 46 homolog
Q805E5	*chst14*	Carbohydrate sulfotransferase 14
O35658	*C1qbp*	Complement component 1 Q subcomponent-binding protein, mitochondrial
A7RWC9	*v1g163483*	Inosine triphosphate pyrophosphatase
Q6GR10	*maea*	Macrophage erythroblast attacher
Q3ZBF3	*MRPL38*	39S ribosomal protein L38, mitochondrial
Q6VVD7	*sox8*	Transcription factor Sox-8
Q9NY43	*BARHL2*	BarH-like 2 homeobox protein
Q6NUQ1	*RINT1*	RAD50-interacting protein 1
Q9R1K9	*Cetn2*	Centrin-2
Q5U3Y0	*Klhdc10*	Kelch domain-containing protein 10
Q8NHY2	*RFWD2*	E3 ubiquitin-protein ligase RFWD2
Q9Y226	*SLC22A13*	Solute carrier family 22 member 13
Q9CZD3	*Gars*	Glycine--tRNA ligase
Q9UBB5	*MBD2*	Methyl-CpG-binding domain protein 2
P70333	*Hnrnph2*	Heterogeneous nuclear ribonucleoprotein H2

P23687	*PREP*	Prolyl endopeptidase
Q5ZIW2	*CNOT10*	CCR4-NOT transcription complex subunit 10
Q9VUL9	*FucTA*	Glycoprotein 3-alpha-L-fucosyltransferase A
Q6GMD3		UPF0515 protein C19orf66 homolog
Q66IJ0	*nup35*	Nucleoporin NUP53
Q2HZX7	*fuz*	Protein fuzzy homolog
P28482	*MAPK1*	Mitogen-activated protein kinase 1
Q925E0	*Sntg2*	Gamma-2-syntrophin

Table S15. Swiss-port annotation of genes in profile D.

Swiss-prot ID	Gene Name	Description
Q5ZLD3	*KLHL13*	Kelch-like protein 13
Q8N2E2	*VWDE*	von Willebrand factor D and EGF domain-containing protein
Q8TB45	*DEPTOR*	DEP domain-containing mTOR-interacting protein
Q6DFK2	*slc25a40*	Solute carrier family 25 member 40
P10995	*act2*	Actin, alpha skeletal muscle 2
Q8K2K6	*Agfg1*	Arf-GAP domain and FG repeat-containing protein 1
Q8QHJ8	*xpo6*	Exportin-6
A1A4J7	*SMG8*	Protein SMG8
P51798	*CLCN7*	H(+)/Cl(-) exchange transporter 7
P98175	*RBM10*	RNA-binding protein 10
Q9BQS8	*FYCO1*	FYVE and coiled-coil domain-containing protein 1
Q8BG99	*Pknox2*	Homeobox protein PKNOX2
Q5PR73	*Diras2*	GTP-binding protein Di-Ras2
Q6INP9	*bcar3*	Breast cancer anti-estrogen resistance protein 3 homolog
P28661	*Septin4*	Septin-4
Q7ZX37	*jmjd6-b*	Bifunctional arginine demethylase and lysyl-hydroxylase JMJD6-B
Q5ZMK5	*JMJD6*	Bifunctional arginine demethylase and lysyl-hydroxylase JMJD6
Q8NE35	*CPEB3*	Cytoplasmic polyadenylation element-binding protein 3
Q5VUA4	*ZNF318*	Zinc finger protein 318
Q9VHU1	*CG9630*	Probable ATP-dependent RNA helicase DDX55 homolog
P20722	*Bmp6*	Bone morphogenetic protein 6
Q6P791	*Lamtor1*	Ragulator complex protein LAMTOR1
Q9QX29	*Trpc5*	Short transient receptor potential channel 5
Q4FZZ1	*Pxk*	PX domain-containing protein kinase-like protein

Q4R495	*MFSD11*	UNC93-like protein MFSD11
Q70IA6	*MOB2*	MOB kinase activator 2
O08759	*Ube3a*	Ubiquitin-protein ligase E3A
Q5ZL54	*RCJMB04_7l9*	UPF0469 protein KIAA0907 homolog
Q5I0I9	*Coq10b*	Coenzyme Q-binding protein COQ10 homolog B, mitochondrial
O75976	*CPD*	Carboxypeptidase D
P52848	*NDST1*	Bifunctional heparan sulfate N-deacetylase/N-sulfotransferase 1
A2ARA8	*Itga8*	Integrin alpha-8
Q9NX57	*RAB20*	Ras-related protein Rab-20
Q3UHG7	*Lchn*	Protein LCHN
Q3V0J4	*Ankrd53*	Ankyrin repeat domain-containing protein 53
Q8AVQ6	*syf2*	Pre-mRNA-splicing factor syf2
P27039	*acvr2a*	Activin receptor type-2A
Q6DG88	*atg4b*	Cysteine protease ATG4B
Q5SP50	*zcrb1*	Zinc finger CCHC-type and RNA-binding motif-containing protein 1
Q6AY70	*Kansl2*	KAT8 regulatory NSL complex subunit 2
Q8TEK3	*DOT1L*	Histone-lysine N-methyltransferase, H3 lysine-79 specific
Q99KY4	*Gak*	Cyclin-G-associated kinase
Q9VC27	*nct*	Nicastrin
Q8VHY0	*Cspg4*	Chondroitin sulfate proteoglycan 4
Q8TDM6	*DLG5*	Disks large homolog 5
P25210	*NFYB*	Nuclear transcription factor Y subunit beta
Q14511	*NEDD9*	Enhancer of filamentation 1
P26009	*ITGA8*	Integrin alpha-8
Q2NL67	*PARP6*	Poly [ADP-ribose] polymerase 6
P35289	*Rab15*	Ras-related protein Rab-15

Q14671	*PUM1*	Pumilio homolog 1
Q9UL36	*ZNF236*	Zinc finger protein 236
O60293	*ZFC3H1*	Zinc finger C3H1 domain-containing protein
Q6INE8	*vmp1*	Vacuole membrane protein 1
Q62896	*Bet1*	BET1 homolog
P27465	*PISD*	Phosphatidylserine decarboxylase proenzyme
P48317	*Gadd45a*	Growth arrest and DNA damage-inducible protein GADD45 alpha
Q3UES3	*Tnks2*	Tankyrase-2
Q95SX7	*RTase*	Probable RNA-directed DNA polymerase from transposon BS
Q9UA35	*SLC28A3*	Solute carrier family 28 member 3
Q04656	*ATP7A*	Copper-transporting ATPase 1
P16671	*CD36*	Platelet glycoprotein 4
O94868	*FCHSD2*	F-BAR and double SH3 domains protein 2
Q9NRA2	*SLC17A5*	Sialin
Q5VT25	*CDC42BPA*	Serine/threonine-protein kinase MRCK alpha
Q6PD21	*Shb*	SH2 domain-containing adapter protein B
Q8HZV4	*TUBD1*	Tubulin delta chain
P15550	*ND3*	NADH-ubiquinone oxidoreductase chain 3
Q9CR57	*Rpl14*	60S ribosomal protein L14
Q8N2K1	*UBE2J2*	Ubiquitin-conjugating enzyme E2 J2
E1BMN8	*NLK*	Serine/threonine-protein kinase NLK
P17130	*hnrnpa1*	Heterogeneous nuclear ribonucleoproteins A1 homolog
F1R2X6	*psme4b*	Proteasome activator complex subunit 4B
P25723	*tld*	Dorsal-ventral patterning protein tolloid
P56677	*St14*	Suppressor of tumorigenicity 14 protein homolog
Q96J88	*EPSTI1*	Epithelial-stromal interaction protein 1

Q9Y4A5	*TRRAP*	Transformation/transcription domain-associated protein
Q9XZ08	*botv*	Exostosin-3
O95436	*SLC34A2*	Sodium-dependent phosphate transport protein 2B
Q9BXS9	*SLC26A6*	Solute carrier family 26 member 6
Q9R155	*Slc26a4*	Pendrin
Q6FPI4	*RAD18*	Postreplication repair E3 ubiquitin-protein ligase RAD18
Q5ZLM8	*FAM133*	Protein FAM133
Q8R3V6	*Cuedc1*	CUE domain-containing protein 1
Q6Q899	*Ddx58*	Probable ATP-dependent RNA helicase DDX58
Q3T0K9	*DAZAP2*	DAZ-associated protein 2
O88843	*Cradd*	Death domain-containing protein CRADD
Q9JLB2	*Mpp5*	MAGUK p55 subfamily member 5
Q9UHW9	*SLC12A6*	Solute carrier family 12 member 6
O95405	*ZFYVE9*	Zinc finger FYVE domain-containing protein 9
Q5ZIP3	*DOHH*	Deoxyhypusine hydroxylase
Q6AZN8	*slc30a6-a*	Zinc transporter 6-A
Q8BIE6	*Frmd4a*	FERM domain-containing protein 4A
Q7JK25	*B3GALT1*	Beta-1,3-galactosyltransferase 1
P61514	*Rpl37a*	60S ribosomal protein L37a
Q29J90	*moody*	G-protein coupled receptor moody
Q9H4B6	*SAV1*	Protein salvador homolog 1
Q2T9T9	*FBXW9*	F-box/WD repeat-containing protein 9
Q12894	*IFRD2*	Interferon-related developmental regulator 2
Q86W33	*TPRA1*	Transmembrane protein adipocyte-associated 1
P12270	*TPR*	Nucleoprotein TPR
Q75WF2		Plancitoxin-1

Q5FVG2	*Epb41l5*	Band 4.1-like protein 5
Q96PE6	*ZIM3*	Zinc finger imprinted 3
P17980	*PSMC3*	26S protease regulatory subunit 6A
Q9Y6F1	*PARP3*	Poly [ADP-ribose] polymerase 3
P49540	*ycf45*	Uncharacterized protein ycf45
O15530	*PDPK1*	3-phosphoinositide-dependent protein kinase 1
Q8TAE6	*PPP1R14C*	Protein phosphatase 1 regulatory subunit 14C
P22897	*MRC1*	Macrophage mannose receptor 1
Q9HBY0	*NOX3*	NADPH oxidase 3
Q96JH7	*VCPIP1*	Deubiquitinating protein VCIP135
Q92797	*SYMPK*	Symplekin
Q9Y6V7	*DDX49*	Probable ATP-dependent RNA helicase DDX49
O02703	*BAX*	Apoptosis regulator BAX
Q96PV6	*LENG8*	Leukocyte receptor cluster member 8
Q9QUI0	*Rhoa*	Transforming protein RhoA
Q9DB05	*Napa*	Alpha-soluble NSF attachment protein
Q3T0D7	*SAR1A*	GTP-binding protein SAR1a
Q9BRS2	*RIOK1*	Serine/threonine-protein kinase RIO1
P52742	*ZNF135*	Zinc finger protein 135
Q9JJA7	*Ccnl2*	Cyclin-L2
Q7ZVX0	*ccnl1*	Cyclin-L1
Q5E9R2	*AGPAT4*	1-acyl-sn-glycerol-3-phosphate acyltransferase delta
Q92636	*NSMAF*	Protein FAN
Q8BFW3	*Ppp1r15b*	Protein phosphatase 1 regulatory subunit 15B
A6QNK1	*GAL3ST1*	Galactosylceramide sulfotransferase
Q9CY45	*N6amt2*	Protein-lysine N-methyltransferase N6amt2

Q7T2P6	*timm23*	Mitochondrial import inner membrane translocase subunit Tim23
O76757	*Ahcy13*	Adenosylhomocysteinase
O75061	*DNAJC6*	Putative tyrosine-protein phosphatase auxilin
P79244	*RPL37*	60S ribosomal protein L37
Q9C0H5	*ARHGAP39*	Rho GTPase-activating protein 39
Q9HAP2	*MLXIP*	MLX-interacting protein
Q7ZW25	*chmp2a*	Charged multivesicular body protein 2a
Q91YT2	*Rnf185*	E3 ubiquitin-protein ligase RNF185
Q71U34	*HSPA8*	Heat shock cognate 71 kDa protein
Q5ZJY3	*GARNL3*	GTPase-activating Rap/Ran-GAP domain-like protein 3
B7ZCC9	*Adgrg4*	Adhesion G-protein coupled receptor G4
Q8R1A4	*Dock7*	Dedicator of cytokinesis protein 7
Q96N67	*DOCK7*	Dedicator of cytokinesis protein 7
O14544	*SOCS6*	Suppressor of cytokine signaling 6
Q9JI90	*Rnf14*	E3 ubiquitin-protein ligase RNF14
Q49SH1	*slc24a5*	Sodium/potassium/calcium exchanger 5
Q923Q2	*Stard13*	StAR-related lipid transfer protein 13
Q8IUR6	*CREBRF*	CREB3 regulatory factor
Q61147	*Cp*	Ceruloplasmin
Q9P281	*BAHCC1*	BAH and coiled-coil domain-containing protein 1
Q8AXU9	*SH3GL3*	Endophilin-A3
Q5RBW6	*STX12*	Syntaxin-12
F1N9Y5	*SYK*	Tyrosine-protein kinase SYK
Q66KB0	*ube2z*	Ubiquitin-conjugating enzyme E2 Z
P49908	*SEPP1*	Selenoprotein P
Q9CQV4	*Fam134c*	Protein FAM134C

Q5R6Q7	*SNX16*	Sorting nexin-16
Q5TC82	*RC3H1*	Roquin-1
Q9NJ15	*PC6*	Proprotein convertase subtilisin/kexin type 5
Q969S3	*ZNF622*	Zinc finger protein 622
Q90Y35	*ZNF622*	Zinc finger protein 622
Q969Q5	*RAB24*	Ras-related protein Rab-24
Q8WXF0	*SRSF12*	Serine/arginine-rich splicing factor 12
P70298	*Cux2*	Homeobox protein cut-like 2
Q7L945	*ZNF627*	Zinc finger protein 627
Q9HBF4	*ZFYVE1*	Zinc finger FYVE domain-containing protein 1
P27604	*ahcy-1*	Adenosylhomocysteinase
Q0VFV6	*vimp*	Selenoprotein S
Q5RES1	*MMP14*	Matrix metalloproteinase-14
Q9H9E1	*ANKRA2*	Ankyrin repeat family A protein 2
Q9UBS8	*RNF14*	E3 ubiquitin-protein ligase RNF14
P10072	*HKR1*	Krueppel-related zinc finger protein 1
Q7ZUX6	*dohh*	Deoxyhypusine hydroxylase
Q5REK1	*ZNF136*	Zinc finger protein 136
P50747	*HLCS*	Biotin--protein ligase
Q6PDK8	*Dtx4*	E3 ubiquitin-protein ligase DTX4
Q99946	*PRRT1*	Proline-rich transmembrane protein 1
Q9D1F4	*Akt1s1*	Proline-rich AKT1 substrate 1
Q9R1A8	*Rfwd2*	E3 ubiquitin-protein ligase RFWD2
Q8N448	*LNX2*	Ligand of Numb protein X 2
Q76MZ3	*Ppp2r1a*	Serine/threonine-protein phosphatase 2A 65 kDa regulatory subunit A alpha isoform
O76932	*Pp4-19C*	Serine/threonine-protein phosphatase 4 catalytic subunit

O43312	*MTSS1*	Metastasis suppressor protein 1
Q95J75	*SLC25A32*	Mitochondrial folate transporter/carrier
Q9R1D7	*GBF1*	Golgi-specific brefeldin A-resistance guanine nucleotide exchange factor 1
O76536		Hyalin (Fragment)
Q5XIS7	*Ubap1*	Ubiquitin-associated protein 1
Q99816	*TSG101*	Tumor susceptibility gene 101 protein
Q7Z401	*DENND4A*	C-myc promoter-binding protein
Q96EP0	*RNF31*	E3 ubiquitin-protein ligase RNF31
Q9BWT3	*PAPOLG*	Poly(A) polymerase gamma
Q2KIA4	*SCD5*	Stearoyl-CoA desaturase 5
P27987	*ITPKB*	Inositol-trisphosphate 3-kinase B
Q14094	*CCNI*	Cyclin-I
Q9D9G3	*Chic2*	Cysteine-rich hydrophobic domain-containing protein 2
D9IQ16		Galaxin
P97798	*Neo1*	Neogenin
Q924W7	*St5*	Suppression of tumorigenicity 5 protein
Q8BHT7	*Rgp1*	RAB6A-GEF complex partner protein 2
Q8K458	*Prokr2*	Prokineticin receptor 2
A6QPN6	*IFI30*	Gamma-interferon-inducible lysosomal thiol reductase
Q6AZJ9	*rps21*	40S ribosomal protein S21
P14734	*fkh*	Protein fork head
P79401	*CYP3A29*	Cytochrome P450 3A29
Q32NT4	*lrrc58*	Leucine-rich repeat-containing protein 58
Q09225	*nrf-6*	Nose resistant to fluoxetine protein 6
P84103	*SRSF3*	Serine/arginine-rich splicing factor 3
Q5VT06	*CEP350*	Centrosome-associated protein 350

Q7Z6E9	*RBBP6*	E3 ubiquitin-protein ligase RBBP6
Q92539	*LPIN2*	Phosphatidate phosphatase LPIN2
Q6R327	*RICTOR*	Rapamycin-insensitive companion of mTOR
O14901	*KLF11*	Krueppel-like factor 11
Q9JMD3	*Stard10*	PCTP-like protein
P27117	*ODC1*	Ornithine decarboxylase
Q5SSF7	*Fam46c*	Protein FAM46C
Q9P2E3	*ZNFX1*	NFX1-type zinc finger-containing protein 1
O75899	*GABBR2*	Gamma-aminobutyric acid type B receptor subunit 2
Q8NEZ2	*VPS37A*	Vacuolar protein sorting-associated protein 37A
Q9NZS9	*BFAR*	Bifunctional apoptosis regulator
Q7Z3B3	*KANSL1*	KAT8 regulatory NSL complex subunit 1
P26794	*Pim1*	Serine/threonine-protein kinase pim-1
P31483	*TIA1*	Nucleolysin TIA-1 isoform p40
O95786	*DDX58*	Probable ATP-dependent RNA helicase DDX58
Q0V8S0	*HGS*	Hepatocyte growth factor-regulated tyrosine kinase substrate
Q8R4D1	*Slc9a8*	Sodium/hydrogen exchanger 8
Q13772	*NCOA4*	Nuclear receptor coactivator 4
Q5RD69	*SPOCK3*	Testican-3
P97675	*Enpp3*	Ectonucleotide pyrophosphatase/phosphodiesterase family member 3
O14545	*TRAFD1*	TRAF-type zinc finger domain-containing protein 1
Q9Z2A5	*Ate1*	Arginyl-tRNA--protein transferase 1
A5D6U8	*papl*	Iron/zinc purple acid phosphatase-like protein
Q9WTP3	*Spdef*	SAM pointed domain-containing Ets transcription factor
Q80U96	*Xpo1*	Exportin-1
Q8TC07	*TBC1D15*	TBC1 domain family member 15

Q3T178	*VPS28*	Vacuolar protein sorting-associated protein 28 homolog
Q9V359	*Vps28*	Vacuolar protein sorting-associated protein 28 homolog
Q9D1C8	*Vps28*	Vacuolar protein sorting-associated protein 28 homolog
Q96FA3	*PELI1*	E3 ubiquitin-protein ligase pellino homolog 1
P79145	*CREM*	cAMP-responsive element modulator
Q0KL02	*Trio*	Triple functional domain protein
B3STU3	*Chac1*	Glutathione-specific gamma-glutamylcyclotransferase 1
P16393	*HMGCR*	3-hydroxy-3-methylglutaryl-coenzyme A reductase
Q32N90	*mbtd1*	MBT domain-containing protein 1
Q4L208	*Slc9a8*	Sodium/hydrogen exchanger 8
Q80UG2	*Plxna4*	Plexin-A4
Q8VE97	*Srsf4*	Serine/arginine-rich splicing factor 4
Q1RMZ1	*C7orf60*	Probable methyltransferase BTM2 homolog
P46109	*CRKL*	Crk-like protein
Q99K85	*Psat1*	Phosphoserine aminotransferase
O15040	*TECPR2*	Tectonin beta-propeller repeat-containing protein 2
Q29466	*ATP6V0A1*	V-type proton ATPase 116 kDa subunit a isoform 1
Q5RAM8	*EAF1*	ELL-associated factor 1
P62501	*Tsc22d1*	TSC22 domain family protein 1
Q10651	*apl-1*	Beta-amyloid-like protein
P52827	*MRPL12*	39S ribosomal protein L12, mitochondrial
P13255	*Gnmt*	Glycine N-methyltransferase
P37892	*cpe*	Carboxypeptidase E
O95989	*NUDT3*	Diphosphoinositol polyphosphate phosphohydrolase 1
Q9Z1G4	*Atp6v0a1*	V-type proton ATPase 116 kDa subunit a isoform 1
Q6P3P1	*tax1bp1*	Tax1-binding protein 1 homolog

P70705	*Atp7a*	Copper-transporting ATPase 1
Q90YT3	*rpl35a*	60S ribosomal protein L35a
Q03445	*GluRIA*	Glutamate receptor 1
A2ALS5	*Rap1gap*	Rap1 GTPase-activating protein 1
Q5R8Q4	*PATL1*	Protein PAT1 homolog 1
Q9WUU8	*Tnip1*	TNFAIP3-interacting protein 1
Q9UJF2	*RASAL2*	Ras GTPase-activating protein nGAP
Q27433	*mec-2*	Mechanosensory protein 2
Q5ZHZ0	*DDX39B*	Spliceosome RNA helicase DDX39B
P62752	*Rpl23a*	60S ribosomal protein L23a
P62280	*RPS11*	40S ribosomal protein S11
O08992	*Sdcbp*	Syntenin-1
Q5RDU4	*PARP6*	Poly [ADP-ribose] polymerase 6
Q92113	*CYP17A1*	Steroid 17-alpha-hydroxylase/17,20 lyase
Q6T486	*rbrA*	Probable E3 ubiquitin-protein ligase rbrA
Q9W6Y1	*hsc70*	Heat shock cognate 71 kDa protein
P14198	*AAC4*	AAC-rich mRNA clone AAC4 protein
Q9EQT3	*Rhou*	Rho-related GTP-binding protein RhoU
Q9Y6W6	*DUSP10*	Dual specificity protein phosphatase 10
P81134	*ATP6AP2*	Renin receptor
Q04118	*PRB3*	Basic salivary proline-rich protein 3
Q9H3F6	*KCTD10*	BTB/POZ domain-containing adapter for CUL3-mediated RhoA degradation protein 3
Q8BYH7	*Tbc1d17*	TBC1 domain family member 17
Q03468	*ERCC6*	DNA excision repair protein ERCC-6
P61964	*WDR5*	WD repeat-containing protein 5
Q9Y3C5	*RNF11*	RING finger protein 11

Q99LI8	*Hgs*	Hepatocyte growth factor-regulated tyrosine kinase substrate
O35598	*Adam10*	Disintegrin and metalloproteinase domain-containing protein 10
O00560	*SDCBP*	Syntenin-1
Q5RK28	*Nmes1*	Normal mucosa of esophagus-specific gene 1 protein
Q9VZS3	*CG17737*	Protein translation factor SUI1 homolog
Q5RAN1	*RABGAP1*	Rab GTPase-activating protein 1
Q8QZY6	*Tspan14*	Tetraspanin-14
Q92736	*RYR2*	Ryanodine receptor 2
Q8WXU2	*DYX1C1*	Dyslexia susceptibility 1 candidate gene 1 protein
Q9BXK1	*KLF16*	Krueppel-like factor 16
Q6ZV89	*SH2D5*	SH2 domain-containing protein 5
Q66T02	*Plekhg5*	Pleckstrin homology domain-containing family G member 5
Q4QQW8	*Plbd2*	Putative phospholipase B-like 2
Q9UJT1	*TUBD1*	Tubulin delta chain
Q4LDE5	*SVEP1*	Sushi, von Willebrand factor type A, EGF and pentraxin domain-containing protein 1
Q90YR4	*rps10*	40S ribosomal protein S10
Q26636		Cathepsin L
Q5JWR5	*DOPEY1*	Protein dopey-1
Q14596	*NBR1*	Next to BRCA1 gene 1 protein
Q13905	*RAPGEF1*	Rap guanine nucleotide exchange factor 1
Q6PGV1	*atp6v0d1*	V-type proton ATPase subunit d 1
Q9UDY8	*MALT1*	Mucosa-associated lymphoid tissue lymphoma translocation protein 1
Q5PQT2	*Mtmr3*	Myotubularin-related protein 3
Q1LV17	*irf2bp1*	Interferon regulatory factor 2-binding protein 1
Q5RE11	*TSPAN3*	Tetraspanin-3
P05932		Calmodulin-beta (Fragment)

P49760	*CLK2*	Dual specificity protein kinase CLK2
Q5SDR3	*BMI1*	Polycomb complex protein BMI-1
Q9VGY6	*Skeletor*	Protein Skeletor, isoforms B/C
Q8BH48	*Ubap1*	Ubiquitin-associated protein 1
Q03141	*Mark3*	MAP/microtubule affinity-regulating kinase 3
Q90889	*MAFG*	Transcription factor MafG
Q5E938	*EIF1*	Eukaryotic translation initiation factor 1
Q1L5Z9	*LONRF2*	LON peptidase N-terminal domain and RING finger protein 2
O14964	*HGS*	Hepatocyte growth factor-regulated tyrosine kinase substrate
Q00651	*Itga4*	Integrin alpha-4
Q6P0V6	*rpl8*	60S ribosomal protein L8
O93477	*ahcy-b*	Adenosylhomocysteinase B
Q9Y0H3	*rps19*	40S ribosomal protein S19
Q8BSM7	*Slc43a1*	Large neutral amino acids transporter small subunit 3
Q80XI3	*Eif4g3*	Eukaryotic translation initiation factor 4 gamma 3
Q80U16	*Fam65b*	Protein FAM65B
Q9H3R0	*KDM4C*	Lysine-specific demethylase 4C
Q9PSX7	*RHOC*	Rho-related GTP-binding protein RhoC
P52198	*RND2*	Rho-related GTP-binding protein RhoN
P41567	*EIF1*	Eukaryotic translation initiation factor 1
Q9Y2G3	*ATP11B*	Probable phospholipid-transporting ATPase IF
P0CG68	*UBC*	Polyubiquitin-C
Q9NRJ4	*TULP4*	Tubby-related protein 4
Q9V3G1	*RpL8*	60S ribosomal protein L8
O08727	*Tnfrsf11b*	Tumor necrosis factor receptor superfamily member 11B
P46530	*notch1a*	Neurogenic locus notch homolog protein 1

F7H9X2	*TRIM2*	Tripartite motif-containing protein 2
Q60795	*Nfe2l2*	Nuclear factor erythroid 2-related factor 2
Q9XZE5	*pho2a*	Serine/threonine-protein phosphatase 2A catalytic subunit A
O62806	*MMP13*	Collagenase 3
O77656	*MMP13*	Collagenase 3
O00418	*EEF2K*	Eukaryotic elongation factor 2 kinase
P23526	*AHCY*	Adenosylhomocysteinase
Q9HB90	*RRAGC*	Ras-related GTP-binding protein C
Q6NW85	*arih1l*	E3 ubiquitin-protein ligase arih1l
Q96N64	*PWWP2A*	PWWP domain-containing protein 2A
P08775	*Polr2a*	DNA-directed RNA polymerase II subunit RPB1
Q9HCM2	*PLXNA4*	Plexin-A4
Q8MKD1	*UBB*	Polyubiquitin-B
Q4R5Q0	*RPL3*	60S ribosomal protein L3
P10760	*Ahcy*	Adenosylhomocysteinase
P51893	*ahcy-a*	Adenosylhomocysteinase A
P42839	*VNX1*	Low affinity vacuolar monovalent cation/H(+) antiporter
Q8MUF6		Paramyosin
A9JTG5	*rbck1*	RanBP-type and C3HC4-type zinc finger-containing protein 1
Q6ZN04	*MEX3B*	RNA-binding protein MEX3B
Q96K80	*ZC3H10*	Zinc finger CCCH domain-containing protein 10
Q1HPK6	*tef2*	Translation elongation factor 2
Q9BE45	*NFKBIZ*	NF-kappa-B inhibitor zeta
A1L3F4	*mex3b*	RNA-binding protein MEX3B
P25001	*COI*	Cytochrome c oxidase subunit 1
P21531	*Rpl3*	60S ribosomal protein L3

P29691	*eef-2*	Elongation factor 2
Q49AM3	*TTC31*	Tetratricopeptide repeat protein 31
P0CG73	*UBI1*	Polyubiquitin
Q63429	*Ubc*	Polyubiquitin-C
O16797	*RpL3*	60S ribosomal protein L3
Q9ULZ9	*MMP17*	Matrix metalloproteinase-17
Q640V2	*rmnd5a*	Protein RMD5 homolog A
Q9ULT8	*HECTD1*	E3 ubiquitin-protein ligase HECTD1
P10288	*CDH2*	Cadherin-2
F1P065	*FARP1*	FERM, RhoGEF and pleckstrin domain-containing protein 1
O62683	*TJP3*	Tight junction protein ZO-3
P05412	*JUN*	Transcription factor AP-1
P51968		Heterogeneous nuclear ribonucleoprotein A3 homolog 1
P02595		Calmodulin
Q6DFV5	*Helz*	Probable helicase with zinc finger domain
P11707	*CYP3A6*	Cytochrome P450 3A6
B1AZP2	*Dlgap4*	Disks large-associated protein 4
Q6PB44	*Ptpn23*	Tyrosine-protein phosphatase non-receptor type 23
Q505F5	*Lrrc47*	Leucine-rich repeat-containing protein 47
Q8IX03	*WWC1*	Protein KIBRA
Q1JQA2	*SMAD1*	Mothers against decapentaplegic homolog 1
Q99NH0	*Ankrd17*	Ankyrin repeat domain-containing protein 17
Q27294	*caz*	RNA-binding protein cabeza
Q8NCJ5	*SPRYD3*	SPRY domain-containing protein 3
O75581	*LRP6*	Low-density lipoprotein receptor-related protein 6
O14727	*APAF1*	Apoptotic protease-activating factor 1

Q5SPF7	*unc93a*	Protein unc-93 homolog A
Q05469	*LIPE*	Hormone-sensitive lipase
P39023	*RPL3*	60S ribosomal protein L3
Q9NWZ3	*IRAK4*	Interleukin-1 receptor-associated kinase 4
P50540	*Mxi1*	Max-interacting protein 1
Q7Z7A4	*PXK*	PX domain-containing protein kinase-like protein
Q5ZJ56	*RPL7*	60S ribosomal protein L7
Q00325	*SLC25A3*	Phosphate carrier protein, mitochondrial
Q9BYP7	*WNK3*	Serine/threonine-protein kinase WNK3
Q28709	*CD63*	CD63 antigen
P01122	*RHO*	Ras-like GTP-binding protein RHO
P42678	*AGAP006459*	Protein translation factor SUI1 homolog
Q32L83	*BRI3*	Brain protein I3
Q8K4K3	*Trib2*	Tribbles homolog 2
Q5RF58	*SLC43A2*	Large neutral amino acids transporter small subunit 4
Q90YY5	*med26*	Mediator of RNA polymerase II transcription subunit 26
E7F9T0	*mical1*	Protein-methionine sulfoxide oxidase mical1
Q9HC52	*CBX8*	Chromobox protein homolog 8
Q9UPX0	*IGSF9B*	Protein turtle homolog B
Q8CFI0	*Nedd4l*	E3 ubiquitin-protein ligase NEDD4-like
Q95029	*Cp1*	Cathepsin L
Q9Y2M2	*SSUH2*	Protein SSUH2 homolog
Q99758	*ABCA3*	ATP-binding cassette sub-family A member 3
O60140	*SPBC18H10.09*	Uncharacterized protein C18H10.09
Q5ZMM5	*ZNF706*	Zinc finger protein 706
Q8CFE5	*Btbd7*	BTB/POZ domain-containing protein 7

P35816	*PDP1*	[Pyruvate dehydrogenase [acetyl-transferring]]-phosphatase 1, mitochondrial
P62906	*RPL10A*	60S ribosomal protein L10a
P20749	*BCL3*	B-cell lymphoma 3 protein
Q0P4S0	*zfyve28*	Lateral signaling target protein 2 homolog
Q9P278	*FNIP2*	Folliculin-interacting protein 2
Q8CBY1	*Samd4a*	Protein Smaug homolog 1
P31503	*Pou2f1*	POU domain, class 2, transcription factor 1 (Fragment)
O16110	*AAEL000291*	V-type proton ATPase 16 kDa proteolipid subunit
Q2HJ58	*PRPS1*	Ribose-phosphate pyrophosphokinase 1
Q922Q2	*Riok1*	Serine/threonine-protein kinase RIO1
Q10914	*glr-2*	Glutamate receptor 2
Q7T339	*chmp5*	Charged multivesicular body protein 5
Q9D7S9	*Chmp5*	Charged multivesicular body protein 5
Q9BY84	*DUSP16*	Dual specificity protein phosphatase 16
P21448	*ABCB1*	Multidrug resistance protein 1
Q14151	*SAFB2*	Scaffold attachment factor B2
Q9NTZ6	*RBM12*	RNA-binding protein 12
P53473	*CYIB*	Actin, cytoskeletal 1B
O43752	*STX6*	Syntaxin-6
P41971	*Elk3*	ETS domain-containing protein Elk-3
Q9BGQ3	*Septin5*	Septin-5
Q08AW4	*plekhm3*	Pleckstrin homology domain-containing family M member 3
Q9BSY9	*DESI2*	Desumoylating isopeptidase 2
Q6NYT3	*ier5l*	Immediate early response gene 5-like protein
O96081		Calmodulin-B
P0C2F0	*FAU*	40S ribosomal protein S30

P19120	*HSPA8*	Heat shock cognate 71 kDa protein
Q61371	*Ift88*	Intraflagellar transport protein 88 homolog
A4IIW7	*cdk14*	Cyclin-dependent kinase 14
Q92841	*DDX17*	Probable ATP-dependent RNA helicase DDX17
Q8R420	*Abca3*	ATP-binding cassette sub-family A member 3
P39922		Myosin heavy chain, clone 203 (Fragment)
Q8K0Q5	*Arhgap18*	Rho GTPase-activating protein 18
Q9CS74	*Ecd*	Protein ecdysoneless homolog
P84245	*H3f3b*	Histone H3.3
Q8R0S1	*Atf7*	Cyclic AMP-dependent transcription factor ATF-7
P19439	*KBP*	Probable glutamate receptor
Q7YRV6	*SOCS2*	Suppressor of cytokine signaling 2
Q6PFD9	*Nup98*	Nuclear pore complex protein Nup98-Nup96
Q6P5S7	*RNASEK*	Ribonuclease kappa
Q99L45	*Eif2s2*	Eukaryotic translation initiation factor 2 subunit 2
Q6V4S5	*Sdk2*	Protein sidekick-2
Q9Z1M8	*Ik*	Protein Red
Q64512	*Ptpn13*	Tyrosine-protein phosphatase non-receptor type 13
Q24133	*DnaJ-1*	DnaJ protein homolog 1
Q9D722	*Oser1*	Oxidative stress-responsive serine-rich protein 1
Q8BQQ1	*Zdhhc14*	Probable palmitoyltransferase ZDHHC14
Q5R924	*RPS20*	40S ribosomal protein S20
Q6PB03	*pld3*	Phospholipase D3
Q29503	*UBE2R2*	Ubiquitin-conjugating enzyme E2 R2
Q8TAC1	*RFESD*	Rieske domain-containing protein
P97260	*SCAP*	Sterol regulatory element-binding protein cleavage-activating protein

Q9WV30	*Nfat5*	Nuclear factor of activated T-cells 5
Q5RC80	*RBM39*	RNA-binding protein 39
Q92622	*KIAA0226*	Run domain Beclin-1 interacting and cysteine-rich containing protein
Q9NDJ2	*dom*	Helicase domino
Q96L91	*EP400*	E1A-binding protein p400
P50615	*Btg3*	Protein BTG3
P70110	*CD36*	Platelet glycoprotein 4
Q96PX1	*RNF157*	RING finger protein 157
Q6PBT5	*pyroxd1*	Pyridine nucleotide-disulfide oxidoreductase domain-containing protein 1
O14976	*GAK*	Cyclin-G-associated kinase
Q9NSC7	*ST6GALNAC1*	Alpha-N-acetylgalactosaminide alpha-2,6-sialyltransferase 1
Q1L908	*msto1*	Protein misato homolog 1
Q6XPZ3	*PLA2G15*	Group XV phospholipase A2
O02755	*CEBPB*	CCAAT/enhancer-binding protein beta
Q5HYM0	*ZC3H12B*	Probable ribonuclease ZC3H12B
Q8TB96	*ITFG1*	T-cell immunomodulatory protein
Q9NX94	*WBP1L*	WW domain binding protein 1-like
P35220	*alpha-Cat*	Catenin alpha
P25155	*F10*	Coagulation factor X
Q8R1X6	*Spg20*	Spartin
Q6NVL7	*chmp2b*	Charged multivesicular body protein 2b
A0JPH4	*scap*	Sterol regulatory element-binding protein cleavage-activating protein
Q99JD4	*Clasp2*	CLIP-associating protein 2
P23403	*rps20*	40S ribosomal protein S20
Q6JHU9	*Crebbp*	CREB-binding protein
Q56K04	*CRIP1*	Cysteine-rich protein 1

P24798	*ATP1A3*	Sodium/potassium-transporting ATPase subunit alpha-3
Q9VSF3	*Ubc12*	Nedd8-conjugating enzyme Ubc12
Q14AM7		UPF0472 protein C16orf72 homolog
P10385		Glutenin, low molecular weight subunit
Q96P53	*WDFY2*	WD repeat and FYVE domain-containing protein 2
Q5E9E6	*RPL10A*	60S ribosomal protein L10a
Q8VEK0	*Tmem30a*	Cell cycle control protein 50A
Q6P2D8	*XRRA1*	X-ray radiation resistance-associated protein 1
Q6VAB6	*KSR2*	Kinase suppressor of Ras 2
P97434	*Mprip*	Myosin phosphatase Rho-interacting protein
Q8BGQ6	*Efcab14*	EF-hand calcium-binding domain-containing protein 14
Q9D1P2	*Kat8*	Histone acetyltransferase KAT8
Q90705	*EEF2*	Elongation factor 2
Q5R959	*SPRY2*	Protein sprouty homolog 2
Q5ZJA4	*ACTR5*	Actin-related protein 5
Q9D8B3	*Chmp4b*	Charged multivesicular body protein 4b
Q9XVF7	*rpl-8*	60S ribosomal protein L8
Q05975	*RAB2*	Ras-related protein Rab-2
Q99LN9	*Dohh*	Deoxyhypusine hydroxylase
O88322	*Nid2*	Nidogen-2
O60264	*SMARCA5*	SWI/SNF-related matrix-associated actin-dependent regulator of chromatin subfamily A member 5
Q9Z255	*Ube2a*	Ubiquitin-conjugating enzyme E2 A
Q6ZU64	*CCDC108*	Coiled-coil domain-containing protein 108
Q9D9B4		Leucine-rich repeat-containing protein C10orf11 homolog
P47934	*Crat*	Carnitine O-acetyltransferase
Q6WKZ8	*Ubr2*	E3 ubiquitin-protein ligase UBR2

P62999	*RAC1*	Ras-related C3 botulinum toxin substrate 1
P63001	*Rac1*	Ras-related C3 botulinum toxin substrate 1
P62998	*RAC1*	Ras-related C3 botulinum toxin substrate 1
P60763	*RAC3*	Ras-related C3 botulinum toxin substrate 3
Q6RUV5	*Rac1*	Ras-related C3 botulinum toxin substrate 1
P60764	*Rac3*	Ras-related C3 botulinum toxin substrate 3
Q11011	*Npepps*	Puromycin-sensitive aminopeptidase
A6H7I3	*ANGEL2*	Protein angel homolog 2
Q8BUM9	*Usp43*	Ubiquitin carboxyl-terminal hydrolase 43
Q9Y485	*DMXL1*	DmX-like protein 1
Q6DF27	*chmp1b*	Charged multivesicular body protein 1b
P35624	*TIMP1*	Metalloproteinase inhibitor 1
Q7ZVM1	*zgc:55908*	WASH complex subunit strumpellin
Q2KJ36	*DUSP6*	Dual specificity protein phosphatase 6
Q76FK4	*NOL8*	Nucleolar protein 8
Q9DCT5	*Sdf2*	Stromal cell-derived factor 2
Q9Y5W9	*SNX11*	Sorting nexin-11
P63017	*Hspa8*	Heat shock cognate 71 kDa protein
P11142	*HSPA8*	Heat shock cognate 71 kDa protein
Q9BVA6	*FICD*	Adenosine monophosphate-protein transferase FICD
Q5ZJH7	*CNPPD1*	Protein CNPPD1
Q9Z1Z1	*Eif2ak3*	Eukaryotic translation initiation factor 2-alpha kinase 3
Q9QXN0	*Shroom3*	Protein Shroom3
Q60855	*Ripk1*	Receptor-interacting serine/threonine-protein kinase 1
Q9HD42	*CHMP1A*	Charged multivesicular body protein 1a
P17124	*HRH2*	Histamine H2 receptor

Q96RW7	*HMCN1*	Hemicentin-1
O15550	*KDM6A*	Lysine-specific demethylase 6A
Q8TC21	*ZNF596*	Zinc finger protein 596
Q99N01	*Slco4a1*	Solute carrier organic anion transporter family member 4A1
Q54LN4	*gghA*	Gamma-glutamyl hydrolase A
Q920R6	*Atp6v0a4*	V-type proton ATPase 116 kDa subunit a isoform 4
O43909	*EXTL3*	Exostosin-like 3
P97887	*Psen1*	Presenilin-1
Q5XIP1	*Pelo*	Protein pelota homolog
P48612	*pelo*	Protein pelota
Q91WM3	*Rrp9*	U3 small nucleolar RNA-interacting protein 2
Q5F480	*ITPK1*	Inositol-tetrakisphosphate 1-kinase
P81128	*Arhgap35*	Rho GTPase-activating protein 35
A4IIC5	*slc39a3*	Zinc transporter ZIP3
Q92551	*IP6K1*	Inositol hexakisphosphate kinase 1
Q68FL4	*Ahcyl2*	Putative adenosylhomocysteinase 3
Q96NW4	*ANKRD27*	Ankyrin repeat domain-containing protein 27
Q9P2E2	*KIF17*	Kinesin-like protein KIF17
Q9Y2H1	*STK38L*	Serine/threonine-protein kinase 38-like
Q3U5F4	*Yrdc*	YrdC domain-containing protein, mitochondrial
Q9VUL9	*FucTA*	Glycoprotein 3-alpha-L-fucosyltransferase A
Q3UVC0	*Ksr2*	Kinase suppressor of Ras 2
Q4GXP3	*RpS21*	40S ribosomal protein S21
Q15311	*RALBP1*	RalA-binding protein 1
Q3T904	*ATG9A*	Autophagy-related protein 9A
O18973	*RABGEF1*	Rab5 GDP/GTP exchange factor

Q7ZUA6	*LMBR1*	Limb region 1 protein homolog
Q1L8P7	*thbs3b*	Thrombospondin-3b
Q8NHE4	*ATP6V0E2*	V-type proton ATPase subunit e 2
Q6NUZ2	*ttyh2l*	Protein tweety homolog 2-like
Q0V9V9	*ttyh1*	Protein tweety homolog 1
P46094	*XCR1*	Chemokine XC receptor 1
Q9BU79	*TMEM243*	Transmembrane protein 243
P49137	*MAPKAPK2*	MAP kinase-activated protein kinase 2
Q8IU60	*DCP2*	m7GpppN-mRNA hydrolase
O95628	*CNOT4*	CCR4-NOT transcription complex subunit 4
Q2LC84	*Numb*	Protein numb homolog
Q66II3	*grb2*	Growth factor receptor-bound protein 2
Q9NP78	*ABCB9*	ATP-binding cassette sub-family B member 9
B1AY10	*Nfx1*	Transcriptional repressor NF-X1
Q499B3	*kansl3*	KAT8 regulatory NSL complex subunit 3
O76050	*NEURL1*	E3 ubiquitin-protein ligase NEURL1
Q08D99	*TMEM41A*	Transmembrane protein 41A
Q29451	*MAN2B1*	Lysosomal alpha-mannosidase
Q96MC6	*HIAT1*	Hippocampus abundant transcript 1 protein
P53708	*ITGA8*	Integrin alpha-8
Q27580	*Ahcy13*	Adenosylhomocysteinase
Q8CFY5	*Cox10*	Protoheme IX farnesyltransferase, mitochondrial
P97433	*Arhgef28*	Rho guanine nucleotide exchange factor 28
Q6IQ26	*DENND5A*	DENN domain-containing protein 5A
O08653	*Tep1*	Telomerase protein component 1
Q62210	*Birc2*	Baculoviral IAP repeat-containing protein 2

O43639	*NCK2*	Cytoplasmic protein NCK2
P61357	*RPL27*	60S ribosomal protein L27
E1BD59	*TRIM56*	E3 ubiquitin-protein ligase TRIM56
Q90577	*SRL*	Sarcalumenin
Q6GR25	*pptc7*	Protein phosphatase PTC7 homolog
Q13163	*MAP2K5*	Dual specificity mitogen-activated protein kinase kinase 5
Q8C2A2	*Tsen54*	tRNA-splicing endonuclease subunit Sen54
G3MWR8	*MICAL3*	Protein-methionine sulfoxide oxidase MICAL3
Q91ZZ5	*Rxfp2*	Relaxin receptor 2
Q9Z0M5	*Lipa*	Lysosomal acid lipase/cholesteryl ester hydrolase
P04634	*Lipf*	Gastric triacylglycerol lipase
Q0II91	*DNAJC21*	DnaJ homolog subfamily C member 21
Q91081		Alpha-2 adrenergic receptor
P70459	*Erf*	ETS domain-containing transcription factor ERF
O14807	*MRAS*	Ras-related protein M-Ras
Q9UKP5	*ADAMTS6*	A disintegrin and metalloproteinase with thrombospondin motifs 6
O94966	*USP19*	Ubiquitin carboxyl-terminal hydrolase 19
Q07352	*ZFP36L1*	Zinc finger protein 36, C3H1 type-like 1
O94985	*CLSTN1*	Calsyntenin-1
Q16739	*UGCG*	Ceramide glucosyltransferase
Q8R9T6	*sps1*	Probable serine/threonine-protein kinase Sps1
Q15032	*R3HDM1*	R3H domain-containing protein 1
Q5T5U3	*ARHGAP21*	Rho GTPase-activating protein 21
P42338	*PIK3CB*	Phosphatidylinositol 4,5-bisphosphate 3-kinase catalytic subunit beta isoform
Q74FW6	*tdcB*	L-threonine ammonia-lyase
A2CG63	*Arid4b*	AT-rich interactive domain-containing protein 4B

Q7ZV79	*uck2b*	Uridine-cytidine kinase 2-B
Q96CN9	*GCC1*	GRIP and coiled-coil domain-containing protein 1
Q28FH2	*scyl1*	N-terminal kinase-like protein
Q8N961	*ABTB2*	Ankyrin repeat and BTB/POZ domain-containing protein 2
Q6KAU4	*Mvb12b*	Multivesicular body subunit 12B
Q86TD4	*SRL*	Sarcalumenin
Q14644	*RASA3*	Ras GTPase-activating protein 3
Q5F2E8	*Taok1*	Serine/threonine-protein kinase TAO1
Q9R0M5	*Tpk1*	Thiamin pyrophosphokinase 1
Q6PC19	*ssu72*	RNA polymerase II subunit A C-terminal domain phosphatase SSU72
Q60773	*Cdkn2d*	Cyclin-dependent kinase 4 inhibitor D
O00222	*GRM8*	Metabotropic glutamate receptor 8
P22792	*CPN2*	Carboxypeptidase N subunit 2
Q0PV50	*TLR3*	Toll-like receptor 3
P82252	*Slc7a9*	b(0,+)-type amino acid transporter 1
Q9H8Y5	*ANKZF1*	Ankyrin repeat and zinc finger domain-containing protein 1
Q6R653	*Unc5cl*	UNC5C-like protein
Q9VRP9	*Bre1*	E3 ubiquitin-protein ligase Bre1
A2A4P0	*Dhx8*	ATP-dependent RNA helicase DHX8
Q923S6	*Neurl1*	E3 ubiquitin-protein ligase NEURL1
Q9BYG0	*B3GNT5*	Lactosylceramide 1,3-N-acetyl-beta-D-glucosaminyltransferase
Q6AYQ1	*Golga7*	Golgin subfamily A member 7
Q8IV50	*LYSMD2*	LysM and putative peptidoglycan-binding domain-containing protein 2
Q80X82	*Sympk*	Symplekin
Q8K1N1	*Pnpla8*	Calcium-independent phospholipase A2-gamma
Q5XTS1	*PNPLA8*	Calcium-independent phospholipase A2-gamma

Q5BLD8	*mettl21a*	Protein N-lysine methyltransferase METTL21A
Q05922	*Dusp2*	Dual specificity protein phosphatase 2
Q7ZVW9	*arglu1b*	Arginine and glutamate-rich protein 1-B
Q96KC2	*ARL5B*	ADP-ribosylation factor-like protein 5B
Q6IQ16	*SPOPL*	Speckle-type POZ protein-like
Q69566	*U88*	Uncharacterized protein U88
Q6NRZ4	*col4a3bp*	Collagen type IV alpha-3-binding protein
Q8CF97	*Vcpip1*	Deubiquitinating protein VCIP135
Q7Z6J2	*GRASP*	General receptor for phosphoinositides 1-associated scaffold protein
O95251	*KAT7*	Histone acetyltransferase KAT7
O43166	*SIPA1L1*	Signal-induced proliferation-associated 1-like protein 1
Q8TBB1	*LNX1*	E3 ubiquitin-protein ligase LNX
Q9VVX5	*nes*	Lysophospholipid acyltransferase 5
Q6P1A2	*LPCAT3*	Lysophospholipid acyltransferase 5
Q91V01	*Lpcat3*	Lysophospholipid acyltransferase 5
P25291	*GP2*	Pancreatic secretory granule membrane major glycoprotein GP2
Q9VY77	*jub*	LIM domain-containing protein jub
Q5TYQ1	*zyg11*	Protein zyg-11 homolog
Q17QU4	*RAB39B*	Ras-related protein Rab-39B
Q28CY9	*xpr1*	Xenotropic and polytropic retrovirus receptor 1 homolog
Q9UBH6	*XPR1*	Xenotropic and polytropic retrovirus receptor 1
O13016	*PTPN1*	Tyrosine-protein phosphatase non-receptor type 1
A1L0Y2	*neurl4*	Neuralized-like protein 4
Q6PFK1	*znf598*	Zinc finger protein 598
E7FAM5	*trim71*	E3 ubiquitin-protein ligase TRIM71
Q9ESK9	*Rb1cc1*	RB1-inducible coiled-coil protein 1

Q8LB33	*At3g58530*	F-box protein At3g58530
Q7Q6S8	*MED14*	Mediator of RNA polymerase II transcription subunit 14
Q9H2G9	*BLZF1*	Golgin-45
Q25C79	*Akirin2*	Akirin-2
Q7T3P8	*fem1c*	Protein fem-1 homolog C
Q62720	*Slc30a1*	Zinc transporter 1
Q16611	*BAK1*	Bcl-2 homologous antagonist/killer
Q7Z698	*SPRED2*	Sprouty-related, EVH1 domain-containing protein 2
Q803H0	*zgc:55781*	UPF0415 protein C7orf25 homolog
P54923	*Adprh*	[Protein ADP-ribosylarginine] hydrolase
Q1JQB2	*BUB3*	Mitotic checkpoint protein BUB3
Q61070	*Ei24*	Etoposide-induced protein 2.4
O75592	*MYCBP2*	E3 ubiquitin-protein ligase MYCBP2
Q9WU60	*Atrn*	Attractin
O60343	*TBC1D4*	TBC1 domain family member 4
Q9UM11	*FZR1*	Fizzy-related protein homolog
Q68F70	*ints4*	Integrator complex subunit 4
Q5I0J5	*Mitd1*	MIT domain-containing protein 1
P01119	*RAS1*	Ras-like protein 1
P04052	*RpII215*	DNA-directed RNA polymerase II subunit RPB1
O08522	*GOSR1*	Golgi SNAP receptor complex member 1
A2RSY1	*Kansl3*	KAT8 regulatory NSL complex subunit 3
Q5SSH7	*Zzef1*	Zinc finger ZZ-type and EF-hand domain-containing protein 1
Q8N3A8	*PARP8*	Poly [ADP-ribose] polymerase 8
Q7Z2Z2	*EFTUD1*	Elongation factor Tu GTP-binding domain-containing protein 1
Q5RJI4	*Pkdcc*	Extracellular tyrosine-protein kinase PKDCC

Q56R14	*trim33*	E3 ubiquitin-protein ligase TRIM33
Q9NZ56	*FMN2*	Formin-2
O96017	*CHEK2*	Serine/threonine-protein kinase Chk2
A3KPW9	*Bag6*	Large proline-rich protein BAG6
Q8K385	*FRRS1*	Ferric-chelate reductase 1
P26591		Glutamate receptor
Q92783	*STAM*	Signal transducing adapter molecule 1
Q3KTM2	*GDPD5*	Glycerophosphodiester phosphodiesterase domain-containing protein 5
Q8IUQ4	*SIAH1*	E3 ubiquitin-protein ligase SIAH1
Q96LT4	*SAMD8*	Sphingomyelin synthase-related protein 1
Q5ZKK5	*ODF2*	Outer dense fiber protein 2
Q6NRS2	*pqlc1*	PQ-loop repeat-containing protein 1
E1BBQ2	*GPR158*	Probable G-protein coupled receptor 158
Q9H3S7	*PTPN23*	Tyrosine-protein phosphatase non-receptor type 23
P32780	*GTF2H1*	General transcription factor IIH subunit 1
Q2GSX8	*PAB1*	Polyadenylate-binding protein, cytoplasmic and nuclear
